# Mitigating Hidden Climate Change Impacts of Timber Cities Critically Depends on Proactive Forest and Waste Management

**DOI:** 10.1021/acs.est.5c12250

**Published:** 2026-05-14

**Authors:** Alperen Yayla, Augustin Danneaux, Estelle Schurer, Meng Gao, Cagatay Demirci, Colin Rose, Stijn van Ewijk, Rupert J. Myers

**Affiliations:** † Department of Civil and Environmental Engineering, 4615 Imperial College London, Skempton Building, South Kensington Campus, London SW7 2AZ, United Kingdom; ‡ Université Paris-Saclay, AgroParisTech, CNRS, ENPC, Institut Polytechnique de Paris, CIRAD, EHESS, UMR Cired, 94130 Nogent-sur-Marne, France; § Gensler Europe, Thomas More Square, London E1W 1YW, United Kingdom; ∥ B&K Hybrid Solutions, Haslams Lane, Alfreton Road, Derby DE21 4TS, United Kingdom; ⊥ Department of Civil, Environmental and Geomatic Engineering, 4919 University College London, London WC1E 6BT, United Kingdom; # The Bartlett School of Sustainable Construction, University College London, London WC1E 7HB, United Kingdom; E.u.r.o.Tec GmbH, Unter dem Hofe 5, 58099 Hagen, Germany

**Keywords:** Climate impacts, engineered timber, concrete, low-carbon cement, forests, cities, dynamic life cycle assessment

## Abstract

Decarbonization options for buildings include using low-carbon cement and engineered timber. However, the long-term cumulative effects of urbanization, destinations of building materials at end-of-life, CO_2_ uptake from cement carbonation, and biogenic sequestration from biomass regrowth on their climate change impacts remain unclear. Here, we assess the climate change impacts of these dynamic factors on future urban buildings for urban growth between 2025 and 2100, using dynamic life cycle assessment across 14 pathways under various short- and long-term scenarios. Construction of urban buildings using timber (‘timber cities’) can lead to a global temperature increase that is up to 0.023 K lower by 2100 than that caused by their construction using reinforced concrete (‘reinforced concrete cities’). After 2100, timber cities can lead to a temperature increase similar to or higher than reinforced concrete cities if there is poor forest regrowth, high landfill gas release, and incineration. If timber recycling leads to forest aging or deforestation due to reduced motivation for forest regrowth, global temperature can significantly rise compared to a scenario in which timber is recycled while simultaneously maintaining the forest carbon sink, which is the most climate-friendly option. Important global actions to minimize the climate impacts of future cities are (1) to support rapid and large-scale implementation of timber buildings in response to current high urbanization; (2) to proactively develop land, forest, and waste policies that limit future temperature increases caused by poor forest regrowth, landfill gas release, and wood incineration; and (3) to adopt dynamic life cycle assessment and related indicators such as absolute global warming potential in the built environment for climate-related policymaking, rather than using only global warming potential.

## Graphical abstract

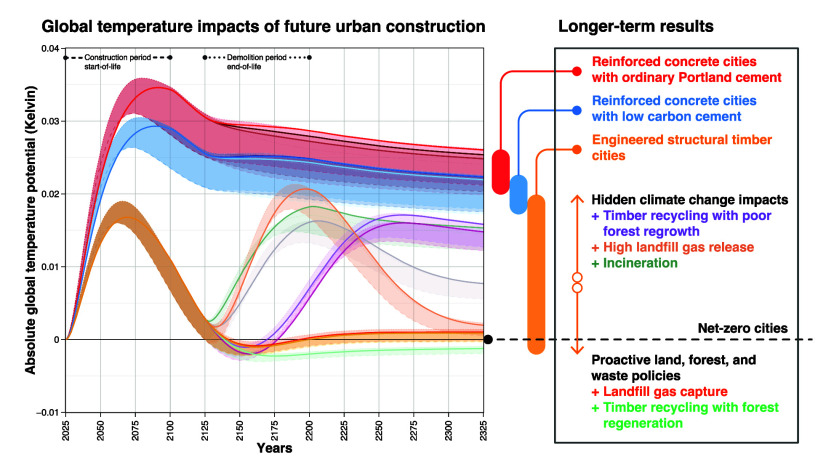

## 1 Introduction

The construction industry depends on the production of infrastructure materials (e.g., steel and cement) that are responsible for ∼13% (4.5 gigatonnes carbon dioxide equivalents; Gt CO_2_-eq.) of annual greenhouse gas (GHG) emissions globally.^1^ Cement production and implementation of concrete structures are the largest contributor to construction emissions, accounting for 7% of global CO_2_-eq. emissions.^2^ To achieve decarbonization aims such as the Paris Agreement, there is a pressing need to decouple climate impacts from construction materials. This need requires a major shift from higher environmental footprint materials (e.g., ordinary Portland cement; OPC) to lower carbon (e.g., low-carbon cement) and biobased materials (e.g., engineered timber).

Many building life cycle assessment (LCA) studies have shown that engineered timber-framed buildings (e.g., residential, commercial, mixed-use, etc.) have lower global warming potential (GWP) over a 100-year time horizon than concrete and steel-frame buildings.^3,4^ Concerns over the climate change impacts of cement production have also accelerated the development of many alternative production methods.^5,6^ A primary method is clinker substitution with supplementary cementitious materials (SCMs) since clinker is the main contributor to the carbon footprints of cement and concrete. For example, limestone calcined clay cement (LC^3^) is a recently developed cement composed of clinker, calcined clay, limestone, and gypsum,^6^ which enables 50% clinker substitution. LC^3^ with 50% clinker substitution has up to 40% lower CO_2_-eq. emissions per mass of cement produced compared to OPC.^7^

Timber, as a biobased material, contributes to climate change mitigation by sequestering CO_2_ during growth. It has been reported that constructing new residential and commercial buildings with engineered timber structural frames to satisfy the demand for 90% of the urban population can store up to 28.1 Gt of CO_2_ from 2020 to 2050 ^8^ (buildings with a 30 square meter (m^2^) per capita average floor area). Concrete also sequesters carbon since cement naturally carbonates in air.^9^ This carbon-sink effect was also calculated to be able to cumulatively store up to 117.2 Gt CO_2_ from 2015 to 2100, offsetting 30% of the global cement cycle emissions (business as usual scenario) over the same period.^9^

However, previous studies that estimate emissions for future use of construction materials oversimplify or do not consider the cumulative effects of dynamic factors in the life cycles of buildings. These dynamic factors are urbanization rate (i.e., proportion of urban population), destinations of building materials at end-of-life, CO_2_ uptake from cement carbonation, and biogenic sequestration from biomass regrowth. For example, current global timber city transition studies^8,10,11^ estimate the benefits of timber cities based on emission factors for materials and the assumption of increased new urban building construction toward 2100. This approach may be beneficial to identify how increased timber demand will be met without damaging forest resources.^10,11^ Yet, current shared socioeconomic pathways (SSPs, which are scenarios used to model future climate impacts based on societal, economic, and environmental developments) project that urban population growth will slow down toward 2100.^12^ This implies that new urban building construction will also decline by 2100 and that far fewer timber buildings may be needed in the future than what is currently modeled.

End-of-life processing of construction materials can be a significant source of climate change impacts, especially for biobased materials.^13^ Decomposition of timber in landfills can lead to methane (CH_4_) and CO_2_ emissions.^14^ Timber recycling can reduce the demand for new wood harvesting, leading to forest aging or deforestation. For example, it has been reported that the forest carbon sink in Japan may have decreased significantly in the 2010s due to the aging of forests planted in the 1960s.^15,16^ Currently, carbon sink strength declines in temperate regions due to forest aging,^17^ whereas in tropical regions, it declines primarily due to deforestation.^16^ There is a lack of consensus about how to allocate the burdens and benefits of these end-of-life issues. Although various end-of-life allocation procedures have been developed,^18,19^ their application to building life cycles remains an active area of research and debate.

Accounting for biogenic carbon and concrete carbonation in LCA is also complex and often controversially implemented. Most LCA studies of buildings either do not account for biogenic carbon or assume that biogenic CO_2_ emissions offset CO_2_ uptake during biomass growth.^20,21^ Sustainably managed forests are often assumed to be carbon-neutral.^22,23^ Yet, the time lag between biogenic emission and sequestration^24,25^ and life cycle (e.g., harvesting, transport, end-of-life) emissions including land use change effects can lead to increased GWP.^26^

In traditional LCA, the GWP metric converts various GHG emissions into kilograms of CO_2_-eq. over a period of atmospheric decay, typically a century (GWP100). The static GWP calculations assume all emissions happen at the same time and do not consider the temporal distribution of GHG emissions throughout the life cycle of products and buildings.^27^ To address these temporal distributions, dynamic GWP calculations have emerged.^28,29^ However, both static and dynamic GWP distort the impact of short-lived (e.g., CH_4_) and long-lived (e.g., nitrous oxide, N_2_O) gases by focusing on specific timeframes (e.g., 20, 100, and 500 year time horizons). This distortion due to the fixed time horizon and neglecting temperature change over time limits the ability of these GWP indicators to compare short- and long-term climate change impacts since they measure the cumulative rather than immediate effects of GHGs.

Due to these limitations of the GWP, there is a need for using alternative LCA indicators in built environment studies, such as the absolute global temperature potential (AGTP).^30^ AGTP quantifies the change in the surface temperature at a specified future time point. Therefore, it enables the tracking of the dynamic climate change impacts of construction materials. This can help (1) LCA practitioners to quantify and compare climate change impacts at a higher level of detail, (2) companies to produce more nuanced roadmaps and targets, and (3) policymakers to make and implement more effective policies for the built environment.

In summary, past studies have not thoroughly investigated the climate change impacts of future urban buildings, especially those that are caused by dynamic factors, compromising the reliabilities of their results. Thorough quantification of the future climate change impacts induced by urban buildings requires investigation of (1) how a declining annual urban population growth affects demand for and emissions from construction materials; (2) the environmental performance of low-carbon cement concrete buildings compared to functionally equivalent engineered timber buildings, (3) different waste treatments (e.g., recycling, incineration, disposal) for these materials; (4) how GHG emissions and sequestration over building life cycles affect atmospheric GHG concentrations and temperature change dynamically; and (5) the use of alternative indicators other than GWP, such as AGTP, that can offer additional insight regarding climate change impacts.

Here, we assess the climate change impacts of constructing urban buildings for the new urban population between 2025 and 2100 via 14 pathways that vary the use of cement-based and timber products in buildings. We model this urban population growth to be satisfied through 4-storey multifamily residential buildings constructed with (1) reinforced OPC concrete (‘BAU’ scenarios), (2) reinforced LC^3^ concrete (‘LC^3^’ scenarios), and (3) engineered structural timber (‘EST’ scenarios). These start-of-life scenarios are accompanied by end-of-life material utilization scenarios. We also classified all scenarios based on the levels of change, relative to the current situation, in material use (for start-of-life scenarios) and forest and waste practices (for end-of-life scenarios) (see Methods, Figure 1).

**Figure 1:**
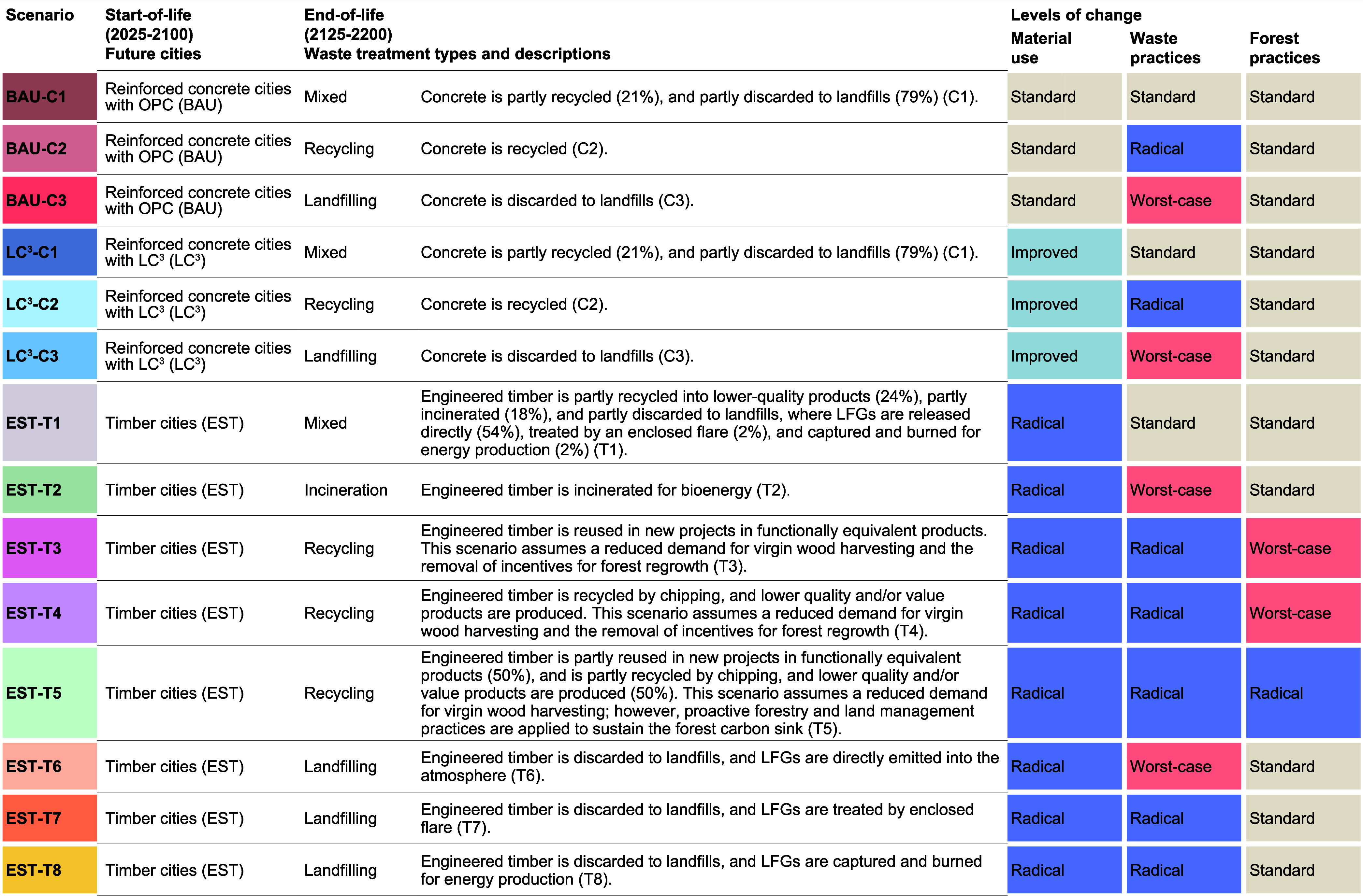
**The urban building scenarios for future cities modeled in this study**. Abbreviations: BAU, business as usual; OPC, ordinary Portland cement; LC^3^, limestone calcined clay cement; LFGs, landfill gases.

This study considers the dynamic effects of both urbanization and emissions/removals through detailed modeling of the urbanization rate, end-of-life waste management, and CO_2_ uptake from both biomass regrowth and carbonation of concrete in future urban buildings. To achieve this, we implemented our dynamic LCA methodology in Python. Construction of buildings is modeled annually for the period 2025–2100 (i.e., start-of-life) according to urban population growth of countries based on SSPs (i.e., SSP1, SSP2, and SSP5 in this study). These buildings are demolished between 2125 and 2200 (i.e., end-of-life) when they reach their 100-year lifespans. Over the full 300 year (2025–2325) period analyzed, we track temperature changes and atmospheric GHG concentrations induced by the construction and demolition of these new urban buildings globally, at country-scale resolution. We also calculated AGTP as well as dynamic and static GWP impact indicators. Global, regional, and country-scale results are provided in a database (https://doi.org/10.5281/zenodo.13886867) (see ref. 31) to facilitate future research for cities and construction materials.

## 2 Methods

### 2.1 Goal and Scope

The main goal of this study is to quantify climate change impacts including GHG emissions induced by future midrise residential and commercial urban buildings under different start-of-life and end-of-life scenarios. We only consider construction of new urban buildings between 2025 and 2100 based on the urban growth between these years, neglecting existing building stock and future construction beyond 2100 in line with previous large-scale urban transition studies^8,10,11^ (the terms ‘timber cities’ and ‘reinforced concrete cities’ represent these new urban buildings in this study). The substitution credits (see Section 2.2 Scenarios) applied in end-of-life scenarios partially address this future construction issue, especially in recycling scenarios, where reused concrete and timber offset the production of virgin materials. To avoid additional complexity and highly uncertain assumptions related to existing building stock, and further construction and demolition phases, we did not extend the model to include these elements.

Our dynamic LCA study assessed the life cycle impacts of buildings from cradle to grave, including both biogenic carbon and concrete carbonation. The case study building has a 1,970 m^2^ gross internal area with four storeys and a lifespan of 100 years. Although traditional LCA studies use a lifespan of ∼50 years, recent studies propose the use of an average lifespan of 100 years.^29,32^ This is because new modern buildings should be long-lasting for both economic and environmental reasons (e.g., cost and climate change impacts of demolishing and rebuilding). For example, one of the recent housing design standards published by the Mayor of London aims to achieve net-zero emission houses that are designed to last at least 200 years^33^ (see Supporting Information S1, Section S1.1.4 for a sensitivity analysis on the building lifespan parameter for values of 50, 100, 150 years).

We presented a novel dynamic LCA model, which simulates the behavior of the elementary flows during and after the lifespan of the buildings. We used a series of Python codes to model and simulate the behavior of GHGs in nature. Atmospheric GHG concentration changes and life cycle impact assessment (LCIA) indicators (i.e., GWPs and AGTP) were also calculated in this code (see Section 2.4 Dynamic LCA).

### 2.2 Scenarios

We developed a comprehensive multistage scenario analysis to achieve our goal (Figure 1). There are two scenario types: (1) start-of-life and (2) end-of-life. Start-of-life scenarios characterize future cities based on structural frame materials of buildings (i.e., reinforced concrete with OPC and LC^3^, or engineered timber), and end-of-life scenarios vary based on the structural frame and characterize end-of-life processing options (e.g., recycling, landfilling). End-of-life use of concrete and timber may also affect other systems such as raw material extraction (i.e., aggregate production, and wood harvesting) and the energy system (i.e., bioenergy and fossil fuel supply). We modeled these effects as end-of-life substitution credits in our dynamic LCA (see Supporting Information S1, Section S4.2 for detailed scenario explanations).

### 2.3 Life Cycle Inventory

Inventory analysis was modeled independently for start-of-life and end-of-life scenarios based on shared socioeconomic pathways (SSPs, i.e., SSP1, SSP2, and SSP5 here). For this, we used the Activity Browser software^34^ and implemented integrated assessment models (IAM)-based life cycle inventories using the ‘*premise*’ (PRospective EnvironMental Impact asSEment) framework by Sacchi et al.^35^ Regionalized Model of Investment and Development (REMIND) IAM scenarios, namely REMIND-SSP1-Base, REMIND-SSP2-Base, and REMIND-SSP5-Base, were used to create life cycle inventories. We used the REMIND-SSPs-Base scenarios since they represent current climate change mitigation policy trajectories and allow a more transparent comparison of construction pathways without assuming additional future mitigation efforts (see Supporting Information S1, Section S1.1.3 for sensitivity analyses based on various REMIND scenarios).

For start-of-life emissions, we modeled the life cycle inventory for 2025 using the ecoinvent^36^ database (v3.10.1, cutoff system model) and for 2050 and 2100 using IAM-integrated data sets generated from the same ecoinvent database through *premise*. For intermediate years, start-of-life emissions were projected using a decreasing exponential interpolation function, since emission trajectories in the REMIND scenarios used in this study mainly follow a rapid early decrease, followed by a progressively slower reduction over time^37^ (see Supporting Information Figure S4 and Supporting Information S1, Section S4.4.2 for details).

Since no IAM projections are available beyond 2100, end-of-life emissions occurring after this year were modeled using the IAM-integrated inventories for 2100, assuming that further emissions beyond 2100 are constant. Then, we performed inventory analysis using this life-cycle inventory as input in our dynamic LCA model.

### 2.4 Dynamic LCA

#### 2.4.1 Model

We modeled the construction of buildings annually for the period 2025–2100 according to the annual urban population growth of the countries based on SSP1, SSP2, and SSP5. To find the total annual new urban building construction area, we assume that all of the new urban population will live in buildings with a 30 m^2^ per capita average floor area.^10^ We performed a sensitivity analysis based on varying space needs per capita, using values^38^ of 9.2, 30, and 79.1 m^2^ per capita, which represent compact, average, and high space-demand urban development patterns and building typologies (see Supporting Information S1, Section S1.1.5). Then, we calculated the number of buildings to be constructed per year by dividing the total annual new urban building construction area by the case study building area. This procedure was performed for each of the countries. The buildings are demolished between 2125 and 2200 according to the order of their construction when their 100-year lifespans expire.

The start-of-life and end-of-life emissions were found by inventory analysis. Then, start-of-life emissions enter the model as pulse emissions (i.e., <1 year duration) based on the construction year of buildings (2025–2100). After 100 years (2125–2200), the end-of-life emissions also enter the model as pulse emissions based on the demolition year of buildings. Biogenic carbon sequestration and carbonation were modeled as a time-dependent CO_2_ sink starting from the start year of the analysis (2025) onward, following the previously introduced methodology from Cherubini et al.^28^ We also calculated the atmospheric decay of emissions based on characteristics of each GHG. This modeling tracks the atmospheric CO_2_ concentration changes over time for 300 years (2025–2325) and reveals when each emission and uptake occurs. We, therefore, took into account the dynamic effects of both urbanization and GHG emissions.

#### 2.4.2 Biogenic Carbon Sequestration

For carbon sequestration through biomass growth, we applied the forward-looking approach, assuming that atmospheric carbon starts to be sequestered during biomass regrowth after the wood harvest. We think that this approach is more appropriate since climate change is an urgent topic and we have a limited carbon budget. This approach emphasizes the importance of replantation after harvest as a practice to maximize climate benefits and captures the advantages of using tree species with shorter rotation periods.^39^ Biomass growth is modeled as a Gaussian distribution, expressed as a function of its rotation period. The choice of rotation period depends on many factors such as tree species, land productivity, and geographical climate, as well as the purpose of the forest.^40^ We assumed a rotation period of 100 years, based on an average of currently recommended rotation lengths in Nordic, Continental, and Mediterranean climates^40^ (see Supporting Information S1, Section S4.5.1 for detailed calculations). We also performed a sensitivity analysis on this forest rotation period parameter for values of 40 (mostly plantation forests, and softwood species), 100, and 160 (mostly hardwood species) years (see Supporting Information S1, Section S1.1.6).

#### 2.4.3 Degradable Organic Carbon in Landfills

For the end-of-life scenarios of timber cities which include landfilling options (T1, T6, T7, and T8), we assumed that an average of 10.5% of the discarded wood is subject to decay in landfills in line with ref.^41^ Since there is a lack of consensus about what percentage of wood is subject to decay, we also provided sensitivity analysis for various values based on different studies^41^ (0.9%, 10.5%, and 23.2% wood decay rates) and the IPCC (Intergovernmental Panel on Climate Change) (50% wood decay rate) (see Supporting Information S1, Section S1.1.7).

Degradable organic carbon has three possible fates for LFGs resulting in CH_4_ and CO_2_ emissions. Here, we used the values by Head et al.:^42^ The share of emitted carbon was modeled as (1) 10% CO_2_ and 90% CH_4_ for landfills with the direct release of LFGs (T1, T6); (2) 99.7% CO_2_ and 0.3% CH_4_ for landfills treated by enclosed flares (T7); and (3) 99.995% CO_2_ and 0.005% CH_4_ for landfills treated through energy production (T8). The degradation of carbon in landfills is modeled using the first-order decay method outlined by the IPCC (Intergovernmental Panel on Climate Change)^27^ (see Supporting Information S1, Section S4.5.2 for detailed calculations).

#### 2.4.4 Carbonation

CO_2_ uptake and storage via carbonation of concrete occur both in the use and end-of-life stages of a building. This was modeled separately for concrete with OPC and LC^3^ since they have different material properties (e.g., calcium oxide content, pore structure, etc.^43^). For both types of concrete, we used the calculation method by Lagerblad^44^ considering appropriate parameters (e.g., supplementary cementitious material coefficient). This method quantifies the amount of CO_2_ uptake in the concrete by measuring the progression of a one-dimensional carbonation front from the concrete surface.^44^ The time-dependent uptake of CO_2_ in concrete is represented using a dynamic formulation, which captures the gradual nature of the carbonation process (see Supporting Information S1, Section S4.5.3 for detailed calculations).

#### 2.4.5 Atmospheric Decay of Emissions

We modeled future GHG changes in the atmosphere based on the decay of GHGs in the air, over 300 years, starting from the start of the building life. This decay reflects wider planetary flows of carbon, which are shared and transferred between the atmosphere, the ocean, and the biosphere. When emissions occur in any given year, a fraction of the GHG released is quickly stored in the ocean’s upper layer. The transport of this GHG to deep ocean layers is slower. When the rate of GHG uptake by oceanic sinks increases, and oceanic GHG concentrations exceed atmospheric levels, some of the GHG initially stored in the ocean’s upper layer is gradually released back into the atmosphere, a process known as outgassing, leading to a re-equilibration between ocean and atmosphere. In the long term, an equilibrium is found between any atmospheric source and sink^45^ (see Supporting Information S1, Section S4.5.4 for detailed calculations).

#### 2.4.6 Life Cycle Impact Assessment

We calculated seven different indicators to provide a comprehensive result in our dynamic LCA: GWP20_static_, GWP20_dynamic_, GWP100_static_, GWP100_dynamic_, GWP200_static_, GWP200_dynamic_, and AGTP (see Supporting Information S1, Section S4.5.5 for detailed life cycle impact assessment calculations).

Static GWP calculations were computed based on IPCC guidelines, assuming all emissions happen at the same time (the start year of the analysis, year 0, 2025 in this study).^27^ To assess progressive removals, the cumulative mass of GHG sequestered at the end of the observation period was considered as a negative emission in the start year of the analysis.

The current and common version of the dynamic GWP^46^ could lead to an overestimation of the possible benefits of temporary carbon storage. Here, we used the expression of dynamic GWP developed by Ventura et al.,^47^ which takes into account the totality of the flows and their impact according to the time horizon of the impact (THI) chosen. The THI is used to calculate the GWP and must be the same for all GHGs contributing to the climate change impact category. Whenever a substance is emitted, its characterization factor must be calculated for the totality of the THI. However, as with all horizon-based metrics, climate effects of early emissions occurring beyond the selected THI are not captured. This limitation should be considered when interpreting the longer-term results.

AGTP measures the change in global mean surface temperature resulting from a unit emission of a particular GHG at a given time and has a unit of Kelvin (K). It provides a direct link between emissions and temperature changes. AGTP is calculated based on the instantaneous radiative forcing (RF), which is the perturbation of the Earth’s energy balance at the top of the atmosphere by a climate change mechanism. This is directly proportional to the change in atmospheric GHG concentration.

## 3 Results

### 3.1 Shorter-Term Effects

#### 3.1.1 Timber Cities Can Reduce the Global Temperature by 2100 Compared to Concrete Cities

The radical level of change in material use toward timber city scenarios consistently shows lower climate change impacts compared to the reinforced concrete scenarios. If the world follows SSP2, the EST scenarios (0.011 K) can reduce global AGTP at 2100 by 0.023 K compared to BAU (0.034 K) and 0.018 K compared to LC^3^ (0.029 K) scenarios, respectively (Figure 2). Timber cities can also cumulatively store 36.3 Gt of CO_2_ in buildings with extensive use of timber (e.g., for structural frame) and reduce atmospheric CO_2_ by up to 9.7 Gt by 2100 through CO_2_ uptake in the global forest sink (Figure 3). Concrete cities can reduce atmospheric CO_2_ by up to 0.8 Gt by 2100 through carbonation. At later times, the superior climate benefits of the timber city scenarios greatly vary depending on how end-of-life timber is utilized (see section 3.2 Longer-Term Effects).

**Figure 2:**
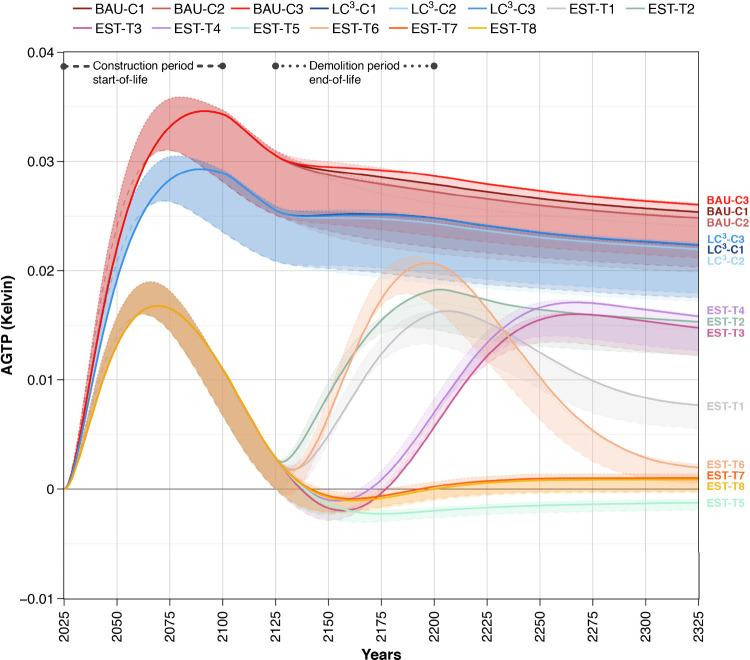
**Absolute global temperature potential (AGTP) of future cities constructed worldwide between 2025 and 2325 based on start-of-life and end-of-life urban building scenarios**. For each scenario, the solid line (SSP2), the dashed line (SSP1), and the dotted line (SSP5) represent shared socioeconomic pathways (SSPs), with the shaded area between these lines quantifying uncertainty. Start-of-life scenarios for future urban buildings cover the construction period between 2025 and 2100. End-of-life scenarios for future urban buildings cover the demolition period between 2125 and 2200.

**Figure 3:**
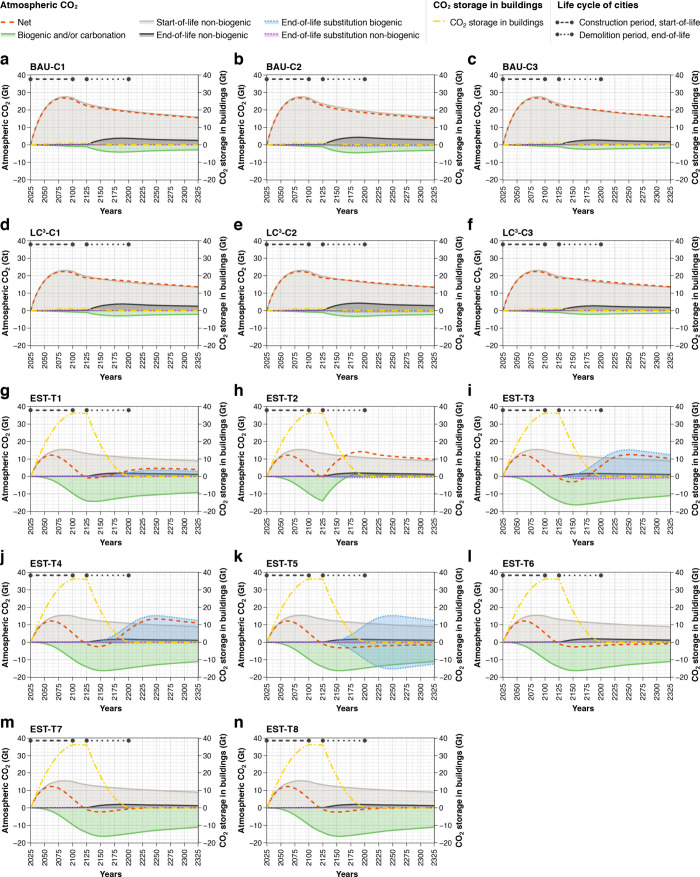
**Atmospheric CO**_**2**_ **concentrations and CO**_**2**_ **storage in buildings between 2025 and 2325 based on scenarios for future timber and reinforced concrete cities constructed worldwide**. **a** BAU-C1, **b** BAU-C2, **c** BAU-C3, **d** LC^3^-C1, **e** LC^3^-C2, **f** LC^3^-C3, **g** EST-T1, **h** EST-T2, **i** EST-T3, **j** EST-T4, **k** EST-T5, **l** EST-T6, **m** EST-T7, **n** EST-T8. The results are shown for SSP2. Start-of-life scenarios for future urban buildings cover the construction period between 2025 and 2100. End-of-life scenarios for future urban buildings cover the demolition period between 2125 and 2200.

Although the climate benefits of timber cities until 2100 apply to all countries, some contribute much more to the global temperature increase than others. India (20%), Nigeria (12%), United States of America (5%), Pakistan (5%), Ethiopia (3%), the Democratic Republic of Congo (DR Congo) (3%), Uganda (3%), and Tanzania (3%) account for 54% of the total global AGTP (0.011 K) in 2100 for all timber city scenarios (Figure 4). This is due to current population increases and/or intensive urbanization in these countries. Large-scale transitions to timber cities in these countries can thus significantly reduce future climate change impacts from construction materials globally by 2100 (Figure 4 and see Supplementary Figure S1). For example, timber city scenarios in India can reduce its AGTP at 2100 by 0.005 K compared to business as usual reinforced concrete city scenarios (0.002 vs 0.007 K) (Figure 4) (see Supporting Information S1, Section S1.3 for country-level detailed results).

**Figure 4:**
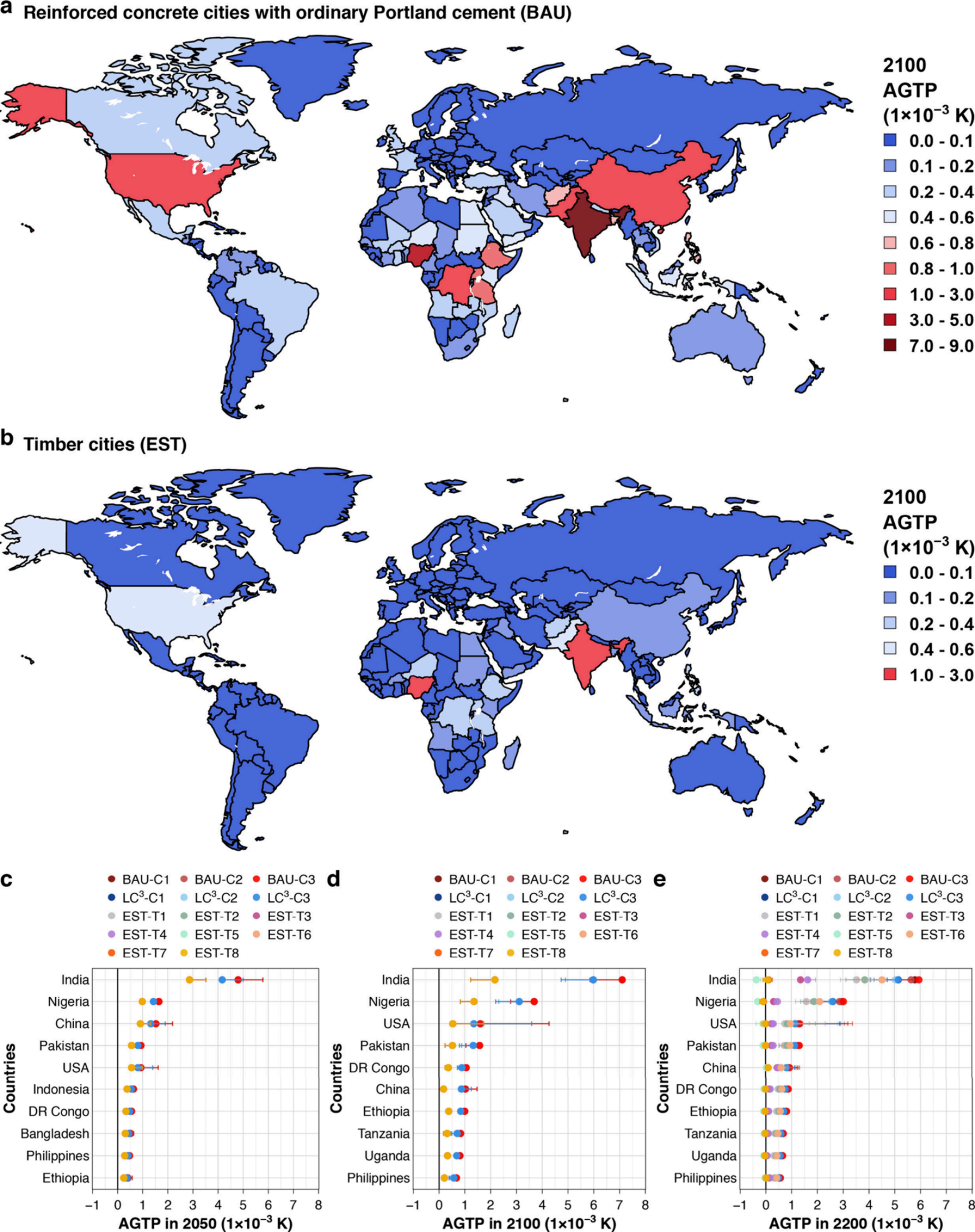
**Geographic breakdown of absolute global temperature potential (AGTP) of future cities in 2050, 2100, and 2100 based on start-of-life and end-of-life urban building scenarios**. **a** AGTP ranges in 2100 at a country-scale resolution for the BAU scenarios, **b** AGTP ranges in 2100 at a country-scale resolution for the EST scenarios, **c** The ten countries with the highest AGTP in 2050, **d** The ten countries with the highest AGTP in 2100, **e** The ten countries with the highest AGTP in 2200. Uncertainties are based on shared socioeconomic pathways (SSPs). For the world maps in a and b, results are shown for SSP2. For graphs in c, d, and e, large dots use SSP2, and error bars quantify uncertainty based on SSP1 and SSP5. Start-of-life scenarios for future urban buildings cover the construction period between 2025 and 2100. End-of-life scenarios for future urban buildings cover the demolition period between 2125 and 2200.

However, it is unclear how the increasing demand for wood will be met in the case of large-scale transitions to timber cities in these countries. Secondary forests (forests that show signs of human intervention and control, i.e., mature wood resources suitable for sustainable forestry) and plantation forest areas are limited in many of these countries. For example, the total industrial roundwood harvest in India, Nigeria, Pakistan, the DR Congo, and Ethiopia accounted for only 3% of the global production volume in 2022.^48^ This implies that there is a substantial deforestation risk associated with the expansion of wood production by increasing harvesting in these countries.^49^ As a priority, these regions need to develop alternative ways to increase timber supply for construction (e.g., shifting wood fuel to industrial use,^8,11^ timber importing,^11^ and circular use of wood^50^) that are quicker and avoid loss of forest ecosystems and biodiversity.^51^

### 3.2 Longer-Term Effects

#### 3.2.1 End-of-Life Use of Timber Affects the Benefits of Timber Cities

For timber cities, atmospheric GHG concentration changes, AGTP, and GWP values at end-of-life (after 2125) vary greatly depending on how timber is utilized at end-of-life and much more so than the corresponding values for reinforced concrete buildings (Figure 3g-n, and see Supporting Information Figure S3g-n).

Incineration of timber waste (EST-T1 and T2, Figure 3g,h) and low forest regrowth (EST-T3 and T4, Figure 3i,j) increase net atmospheric CO_2_ concentrations. Direct release of LFGs increases net atmospheric CH_4_ concentrations (EST-T1,T6, and see Supporting Information Figure S3g,l). Timber recycling with sustaining forest regrowth (EST-T5, Figure 3k) and capturing LFGs lead to reduced net atmospheric GHG concentrations (EST-T7,T8, Figure 3m,n).

These peaks and troughs in GHG concentrations show that end-of-life options for timber have very different climate performance, and this also results in large AGTP and GWP ranges throughout the life cycle of timber cities. For example, timber city scenarios have AGTP ranges between −0.002 (EST-T5) and 0.021 (EST-T6) K at around year 2200 and have AGTP ranges between −0.001 (EST-T5) and 0.016 (EST-T2,T4) at around year 2300. For reinforced concrete city scenarios, the AGTP ranges are smaller, varying between 0.022 K (LC^3^-C2) and 0.029 K (BAU-C3) over the time period from 2200 to 2300.

Timber city scenarios have GWP100_dynamic_ ranges between 10.2 (EST-T5) and 31.6 (EST-T6) Gt CO_2_-eq. and GWP100_static_ ranges between 1.3 (EST-T2) and 41.4 (EST-T6) Gt CO_2_-eq. (Figure 5). For reinforced concrete city scenarios, GWP values vary in a narrower range than timber city scenarios: GWP100_dynamic_ ranges between 54.0 (LC^3^-C2) and 64.3 (BAU-C3) Gt CO_2_-eq., and GWP100_static_ ranges between 55.7 (LC^3^-C2) and 65.2 (BAU-C3) Gt CO_2_-eq. (Figure 5) (see Supporting Information Table S1 for building level GWP results per m^2^ floor space).

**Figure 5:**
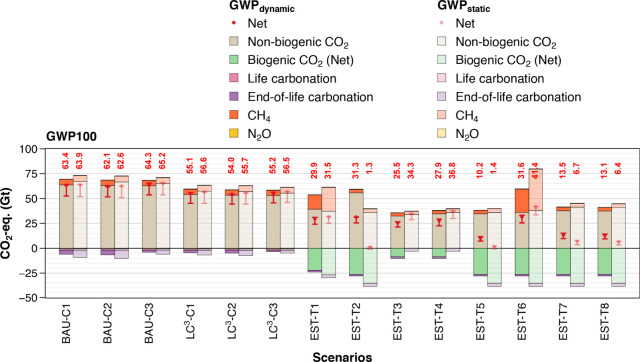
**Dynamic and static global warming potential (GWP) results of future cities based on start-of-life and end-of-life urban building scenarios for the time horizon impact of 100 years (GWP100**_**dynamic**_ **and GWP100**_**static**_ **)**. Uncertainties are based on shared socioeconomic pathways (SSPs). The contribution analysis (i.e., heights of bars for each variable), red dots, and texts for each scenario represent SSP2. Red error bars quantify uncertainty based on SSP1 and SSP5. Start-of-life scenarios for future urban buildings cover the construction period between 2025 and 2100. End-of-life scenarios for future urban buildings cover the demolition period between 2125 and 2200.

Our dynamic GWP calculations consider the instantaneous emission changes by putting more weight on events happening earlier or over a longer period of time (see Methods). This weighting is important to prevent under- or overestimations arising from static GWP calculations, especially in timber city scenarios. Therefore, our dynamic GWP results account for the longer decay of CO_2_ from timber incineration (EST-T1,T2) and the faster decay of CH_4_ from LFG emissions (EST-T1,T6) (Figure 5).

#### 3.2.2 Landfill Gases, Incineration, and Lack of Forest Regrowth Can Significantly Affect Climate Change Impacts

End-of-life emissions of engineered timber such as LFGs and incineration emissions and lack of forest regrowth can cause timber cities to have similar or temporarily higher AGTP compared to reinforced concrete cities.

The type of landfill significantly affects the climate impacts of end-of-life timber. In the EST-T6 scenario which models direct release of LFGs, timber decomposition leads to a peak in atmospheric CH_4_ and AGTP in 2185 that does not appear when LFGs are captured (EST-T7,T8) (Figure 2, and see Supplementary Figure S3l). The AGTP value also depends highly on the end-of-life wood decay rate in landfills: if 10% of landfilled waste wood decays then EST-T6 has similar AGTP to the LC^3^ scenarios at around year 2200 (Figure 2). Since there is a lack of consensus about what percentage of wood is subject to decay, we also performed a sensitivity analysis on this parameter (see Methods). For example, when we assume that 23% of wood is subject to decay in landfills, EST-T6 and EST-T1 can have temporarily higher AGTP at around year 2200 (between 2170 and 2260, for ∼100 years) compared to all reinforced concrete city scenarios (see Supplementary Figures S13, S14).

The worst long-term environmental performance for timber cities is modeled when (1) recycled timber substitutes wood from active harvesting for new engineered timber, which is when there is limited market demand for timber (EST-T3,T4), and (2) end-of-life timber waste is incinerated (EST-T2). Utilization of engineered timber waste may affect resource use in the same (e.g., wood) and different sectors (e.g., energy), making the benefits of timber buildings dependent on those changes. The EST-T4 scenario has the highest AGTP (0.017 K in 2260) among the timber city scenarios after year 2230 (Figure 2). Consequences caused by reducing active harvesting include forest aging and deforestation, both of which reduce the carbon-sink capacity of forests^16,17^ (Figure 3i,j).

Forest regrowth provides a continuous CO_2_ sink except in the modeled incineration scenario (EST-T2). In this scenario, accumulated and stored CO_2_ in buildings that have been demolished starts to be emitted into the atmosphere after year 2125 (Figure 3h). Timber wastes substitute other energy sources when they are incinerated for bioenergy, which reduces nonbiogenic GHG emissions (Figure 3h). Despite this effect, the EST-T2 (0.016 K in 2260) scenario has the second highest AGTP after 2260 due to high biogenic emissions (Figure 2, Figure 3h).

#### 3.2.3 Strong Forest and Waste Management Help Timber Cities Approach Net Zero CO_2_-eq. Emissions

The best long-term environmental performance for timber cities is modeled when CO_2_ is intentionally stored in forests or landfills with LFGs capture. The two scenarios corresponding to this situation are (1) recycling timber waste and sustaining the forest carbon sink through forest regrowth (EST-T5) and (2) discarding timber waste to landfills that both store a portion of the carbon in timber (i.e., as solid waste) and capture and burn LFGs from decomposed timber for energy production (EST-T8). The EST-T5 and EST-T8 scenarios have the two lowest AGTP and GWP among the timber city scenarios (Figure 6).

**Figure 6:**
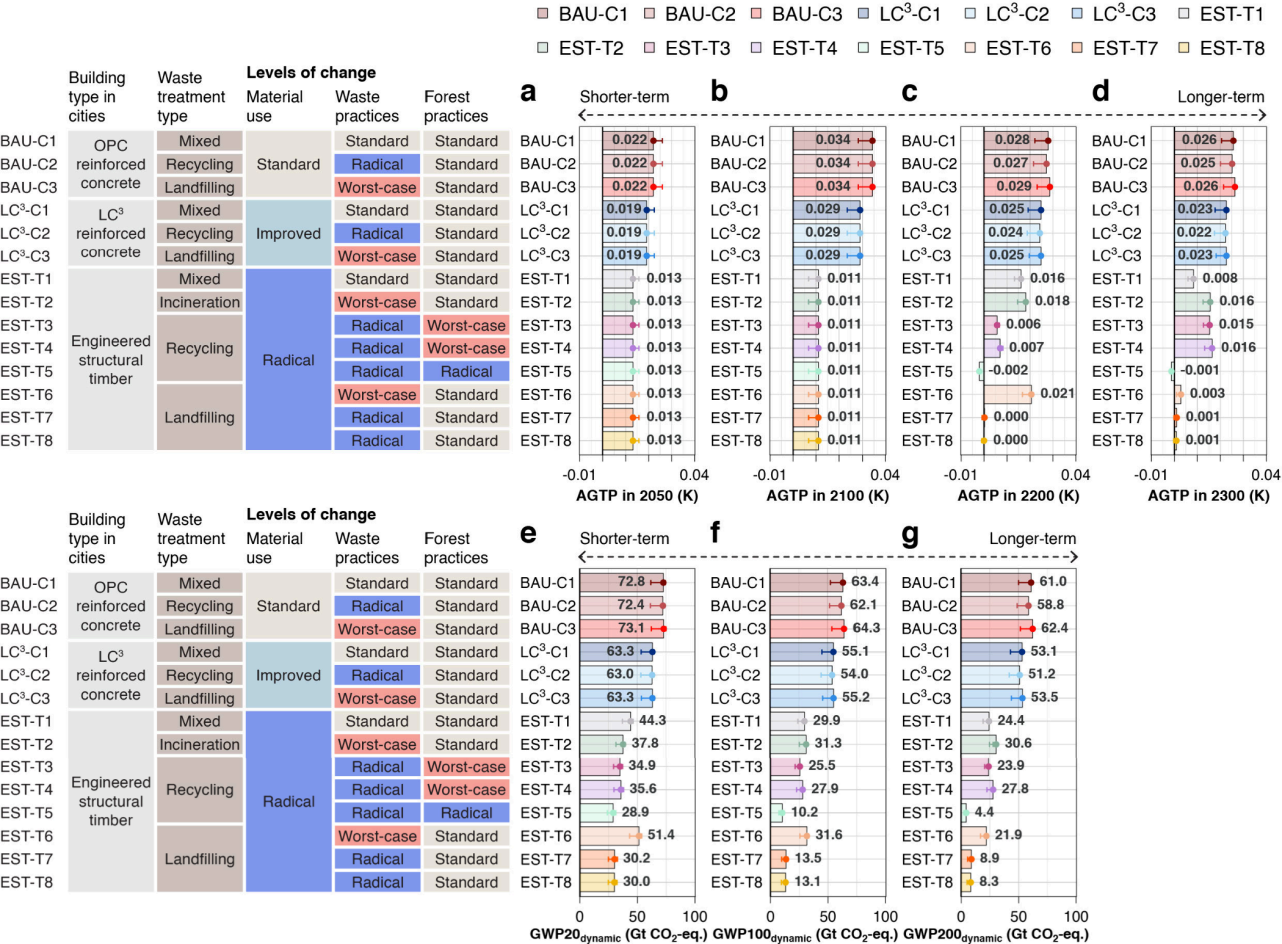
**The drivers of future climate change based on levels of change in material use, waste practices, and forest practices**. **a** AGTP in 2050, **b** AGTP in 2100, **c** AGTP in 2200, **d** AGTP in 2300, **e** GWP20_dynamic_, **f** GWP100_dynamic_, **g** GWP200_dynamic_. The large dots use SSP2, and error bars quantify uncertainty based on SSP1 and SSP5. Start-of-life scenarios for future urban buildings cover the construction period between 2025 and 2100. End-of-life scenarios for future urban buildings cover the demolition period between 2125 and 2200.

Maintaining forests and landfills also has land-use implications. Forests require dedicated land for regrowth, and landfills occupy land that could otherwise be used for other purposes. Identification of potential trade-offs,^52,53^ including effects on biodiversity, soil, and competing land uses, requires local investigations.

Our results show that poor forest management (i.e., lack of forest regrowth) can lead to high climate change performance for future timber cities, despite radical changes to improve waste management (recycling for EST-T3 and T4) (Figure 6). Poor waste practices (incineration, EST-T2; LFGs release, EST-T1,T6) with standard forest management also lead to high climate change impacts (Figure 6).

Timber recycling can reduce virgin wood demand and accelerate timber cities, but radical improvements to sustain longer-term forest regrowth are required to maintain the climate benefits of timber use and achieve future net-zero cities (EST-T5) (Figure 6). Radical improvements in landfilling (EST-T7,T8) can help timber cities approach net-zero with standard forest management since landfilling has a lesser effect on changes in future timber demand and forestry (i.e., for aging or deforestation) (Figure 6).

Our results show that maintaining demand for new timber construction is key to increasing the CO_2_ sink in the built environment, maintaining the CO_2_ sink in forests, and for climate-change mitigation. Long-term slowdowns in population growth and urbanization could reduce demand for new construction and the motivation for plantation forestry regrowth. If so, sustaining a forest carbon sink would require increasing demand for wood products in sectors other than construction or an additional effort such as preventing existing and possible future deforestation in associated areas and encouraging regeneration of natural forest. These efforts can also be opportunities for habitat creation, biodiversity increase, and soil quality improvement.

## 4 Discussion

Existing global timber city transition studies overestimate the climate benefits of timber cities. There are two reasons for this: (1) linear^8,11^ or increasing^10^ demand modeling of annual building construction trends and (2) oversimplification of the dynamic effects of emissions or removals (e.g., end-of-life emissions and time-dependent storage from forest regrowth and carbonation). For example, the timber city scenario with the lowest climate change impact (EST-T5) has an average annual GHG emissions reduction of approximately half of the CO_2_ emissions reduction calculated by Mishra et al.^10^ (1.32 CO_2_ y^–1^ in ref.^10^ vs 0.72 Gt CO_2_-eq. y^–1^ calculated here using GWP100_dynamic_). If the world follows SSP1–5, annual urban population growth and new building construction are projected to decline until 2100 (see Supplementary Figure S15), meaning that it is important for countries to act sooner rather than later to decarbonize the construction sector, when demand is greatest. For example, although Indonesia has the sixth highest AGTP value for all scenarios in 2050, it is not among the top ten countries in 2100 and 2200 (Figure 4). This is because the SSP2 scenario for Indonesia foresees negligible urban population growth after 2065.^12^

Global efforts to mitigate climate change impacts via the transition to timber cities before 2100 should be focused on countries with higher urban population growth during this period, such as India, Nigeria, USA, Pakistan, Ethiopia, DR Congo, Uganda, Tanzania, Indonesia, Bangladesh, and Brazil. Declining urban population growth toward 2100 will increase the importance of extending the life of and repurposing buildings (e.g., industrial to commercial/residential use) to accommodate these future population dynamics instead of constructing new buildings.^54^

The rapid transition to timber cities requires worldwide changes to policymaking to better support timber design and construction, which is currently limited to a few countries/municipalities. Public procurement of timber construction can drive demand and scaling of local mass timber production. For example, France mandated in 2022 that all new public buildings must comprise 50% biobased materials by weight, such as timber, hemp, or straw,^55^ including buildings for the 2024 Paris Olympics. The city of Hamburg implemented financial and regulatory incentives to accelerate mass timber construction, granting €0.3 kg^–1^ wood used in residential buildings and €0.8 kg^–1^ wood used in nonresidential buildings.^55^ Large-scale timber projects, such as Wood City in Finland and Sweden^56^ and Woven City in Japan,^57^ can be promoted in developing countries with high population growth to drive demand for mass timber.

Updating national building codes based on structural innovations may further accelerate adoption. For example, in 2025, Türkiye has published its first timber building design code^58^ (i.e., seismic resilience, material properties, fire safety, and insulation standards^59,60^ ) as mandatory guidelines. In 2025, Ontario raised the height limit for timber buildings from 12 to 18 storeys^61^ (see Supporting Information S1, Section S2 for more details). Better fire engineering knowledge and clearer risk understanding among insurers will improve timber construction feasibility. Adoption also requires speedy upskilling of engineers, architects, and contractors to increase confidence in the ability to deliver timber construction and to prevent costly design and installation mistakes that impact industry or society perception.

Future timber use requires its careful end-of-life management^13,14^ for long-term climate benefits. Direct release of LFGs can cause the environmental impacts of timber buildings to outweigh the environmental benefits that accrue during their operational life. Our sensitivity analysis shows that high landfill decay rates, such as the IPCC-recommended 50%, produce a pronounced CH_4_-driven temperature peak (Supporting Information Figures S13 and S14). The existing literature^41,62^ suggests that the 50% decay rate may overestimate actual wood decomposition in landfills. Policies for timber landfilling should account for uncertainty in decay rates, particularly in landfills with direct LFG release. Measures should prioritize strategies such as LFGs capture that prevent large and rapid methane emissions.

Although methane flaring or incineration can mitigate adverse CH_4_ peak effects, cost-effectiveness and practical implementation aspects of controlled landfilling need to be considered (e.g., high excavation volumes and soil disposal requirements, leachate management, and gas collection system complexity).^63^ For example, based on the waste database of Kaza et al.,^64^ we estimate that 91% of landfills (including open dumps) where wood is discarded directly release LFGs, globally. Investments in timber city transitions, therefore, should also proactively incorporate landfill improvements.

The lack of forest regrowth due to recycling and incineration of timber may raise environmental concerns. Incineration of timber is not carbon neutral due to life cycle (e.g., harvesting, transport) and land use emissions and the time lag between biogenic sequestrations and emissions.^24,25^ Our results show that timber cities have the lowest long-term climate benefits if timber recycling prevents tree regrowth and new plantations for further harvesting. This is because timber demand drives wood harvesting, and if timber demand is met through recycling, then it can imply a reduced need for wood harvesting, forest regrowth, and new plantations. However, if timber recycling reduces wood harvesting but the regrowth of forests is sustained, and so too their carbon sinks, then there can be significant environmental benefits: reduced waste disposal, lower building life cycle climate change impacts, increased forest carbon sink capacity, richer and more biodiverse forests, and better soil quality over the long-term.

To achieve these environmental benefits, proactive forestry practices are required before demand for timber reduces in the future and leads to deforestation and/or forest aging. Current sustainable forestry practices focus on reducing the negative effects of current high wood demand (e.g., overharvesting). For example, selective logging (harvesting only certain trees based on size, species, etc., instead of clear-cutting to preserve forest structure) is globally applied, contributing 15% of global timber needs.^65^ In Sweden, it is reported that at least two new trees are planted for every felled tree.^66^ However, if the demand for virgin wood decreases, logging and replanting may also decrease. Plantation forests are mostly owned by private individuals and companies (e.g., 78% of Swedish forests^67^), and lower wood demand implies lower income and less motivation for replantation. In such cases, deforestation can be prevented by proactively encouraging the regeneration of natural forests or adapting the management of plantation forests, whether that is still for wood production or something else: agriculture, renewable energy generation, CO_2_ storage, etc.

Our results indicate that landfilling of waste timber may be a viable option for sustaining carbon pools in forests under the scenario assumptions of our model, especially in cases where high timber reuse reduces the incentive for forest regeneration. This finding can support decision-makers in evaluating end-of-life management strategies, including the reconsideration of potential bans on timber landfilling. A recently proposed method of burying wood logs in vaults^68^ can also offer a solution to support forest regrowth in areas with suitable terrain (i.e., transport distances, soil type for excavation, etc.). Ultimately, local or regional analyses should be undertaken to determine the desirable options for each specific context.

The GWP indicator can misrepresent climate change impacts of timber buildings, because it is used to reflect climate impacts that vary greatly over decades and centuries in a single number. For example, our GWP20 results for EST-T1 and EST-T6 scenarios show higher or similar climate impacts than reinforced concrete city scenarios (which consider the entire life cycle of buildings) (see Supplementary Figure S2). However, all timber city scenarios show reduced net atmospheric GHG concentrations and GWP100 values than reinforced concrete city scenarios by 2100 (Figures 3–5). Alternative indicators such as AGTP are clearly needed to avoid these possible misinterpretations.

The AGTP indicator enables the climate impacts of buildings to be tracked dynamically. Scenarios with lower AGTP are favored to mitigate climate change impact. Trade-offs between shorter-term and longer-term warming effects can be identified using AGTP. For example, the AGTP shows that timber buildings built in 2025 with lifetimes of 100 years have lower climate change impacts than reinforced concrete buildings by 2100, since the climate change impacts of end-of-life utilization of timber will not yet be apparent. After 2100, AGTP enables the identification of land and waste management strategies that have lower climate change impact. The benefit of the AGTP metric is important for non-CO_2_ gases (e.g., CH_4_) whose measure is highly dependent on the time horizon of the GWP calculation. Here, the AGTP indicator has shown that major drivers of temperature increase (i.e., lack of forest regrowth, landfill gas release, incineration) appear after demolition, meaning that for timber building construction there is a time window of a building lifetime (∼100 years) to prevent these adverse effects. Evidence for developing policies for large-scale transitions in the built environment should utilize dynamic LCA methods and indicators such as the AGTP (see Supporting Information S1, Section S2 for more details).

Our modeling framework assumes a single case study building for all regions and globally. Although existing parameters such as floor area per capita (which is the major driver of building design, material intensity, etc.) vary across regions, this is mainly due to existing socioeconomic inequalities. For example, low-income countries currently tend to have a lower floor area per capita primarily due to economic constraints rather than cultural or architectural preferences. This often results in overcrowded living conditions that fall below minimum space requirements for a comfortable standard of living.^69^ Existing data show that infrastructure material intensities in some countries/regions are even below dignified living standards.^70^ Since our study focuses on newly constructed buildings in future urban developments, we assume that all individuals in modern urban environments should have access to equal baseline living conditions, including floor area per capita, rather than reflecting current inequalities. Although most of the current national design guides do not specify minimum living space per person, we propose that they should incorporate this requirement to establish baseline living standards (e.g., Mayor of London Housing Design Standards^71^) (see Supporting Information S1, Section S3 for more details).

To investigate global long-term climate change impacts of future urban developments, our model uses average parameters (e.g., building lifespan and forest rotation periods). Our scenarios also represent idealized and worst-case conditions to explore different management strategies and policy contexts. This idealization includes an instantaneous global shift to low-carbon cement and/or engineered timber construction (i.e., after 2025). Our sensitivity analyses indicate that variations in the modeling parameters do not alter the main conclusions, and more detailed local investigations could also be performed using our model. For example, various building design, floor space per capita, building lifespan, and forest rotation periods can be used for a specific region. Slow, intermediate, and fast transitions to timber cities could also be adopted by region and would be expected to yield GWP and AGTP values lying between business as usual (BAU) and engineered structural timber (EST) scenarios we modeled. To preserve the readability of the study, we avoided adding additional scenario complexity. A more detailed investigation of afforestation and land management strategies would require the application of independent land-use IAMs (e.g., ref.^72^).

Our life cycle inventory and main results are based on current climate change mitigation policy trajectories (i.e., REMIND-SSPs-Base). Under more ambitious climate policy scenarios, including pathways aligned with countries’ nationally determined contributions, and ∼2 °C and ∼1.5 °C targets, timber-based urban developments can provide a meaningful net cooling effect over the longer-term (Supplementary Figures S5, S6). Achieving such outcomes for sustainable and climate-effective urbanization depends on countries fulfilling their climate pledges and implementing effective climate policies to reduce emissions from material production.

Our results indicate that the most important global actions to minimize the environmental impacts of future cities are both (1) to support rapid and large-scale implementation of timber buildings in response to current high urbanization rates and (2) to proactively develop land, forest, and waste management policies for limiting temperature increases caused by more GHG emissive end-of-life timber utilization options. We also suggest using dynamic LCA and related indicators like the AGTP to reveal the true climate change impacts of construction materials, especially for biobased materials, and using those results to develop more effective mitigation measures.

## Supplementary Material



## Data Availability

All input and output data sets, the dynamic life cycle assessment model codebase, and data tables for the figures in the manuscript are stored in the data repository hosted on Zenodo (https://doi.org/10.5281/zenodo.13886867).^31^ A selection of numerical results is provided with this manuscript (including data presented in the Supporting Information S1). For life cycle inventory analysis, data from the ecoinvent^36^ database (v3.10.1 cutoff) and integrated assessment models (IAM)-based life cycle inventories using the ‘*premise*’ (PRospective EnvironMental Impact asSEment) framework by Sacchi et al.^35^ were used. To calculate global and regional demand for midrise residential and commercial buildings, population and urban population share data were used from the shared socioeconomic pathways (SSPs) scenario database for SSP1, SSP2, and SSP5 projections published by the International Institute for Applied Systems Analysis (IIASA).^12^ For future CO_2_ concentration based on SSPs, we used the data for SSP1-1.9, SSP2-2.6, and SSP5-8.5 projections calculated by Meinshausen et al.^73^ End-of-life waste treatment shares for reinforced concrete and timber were calculated based on the World Bank waste database by Kaza et al.^64^ The dynamic life cycle assessment model was implemented in Python (v3.9.16). The codebase produced in this study is publicly available at https://github.com/ADanneaux/dynamic-LCA and in the data repository hosted on Zenodo (https://doi.org/10.5281/zenodo.13886867).^31^

## Notes

Supplementary Results, Discussions, Limitations, and Methods; Supplementary Tables S1–S4; Supplementary Figures S1–S47; and Supplementary Information S1 References (PDF)
