# Supplementary material for: Mitigating Hidden Climate Change Impacts of Timber Cities Critically Depends on Proactive Forest and Waste Management
Source: Environ Sci Technol. 2026 May 14;60(22):15731–44. doi: 10.1021/acs.est.5c12250 (PMC13261874; doi:10.1021/acs.est.5c12250)
Supplement: Supplementary file 1 [file es5c12250_si_001.pdf]

Supplementary Information S1

Mitigating hidden climate change impacts of timber cities critically depends on proactive forest and waste management

Alperen Yayla [1,a]; Augustin Danneaux [1,2,b]; Estelle Schurer [1,3,c]; Meng Gao [1,d]; Cagatay Demirci [4,e]; Colin Rose [5,f]; Stijn van Ewijk [5,g]; Rupert J. Myers [1,h*]

[1] Department of Civil and Environmental Engineering, Imperial College London, Skempton Building, South Kensington Campus, London, SW7 2AZ, United Kingdom.

[2] Université Paris-Saclay, AgroParisTech, CNRS, ENPC, Institut Polytechnique de Paris, CIRAD, EHESS, UMR Cired, Nogent-surMarne, 94130, France.

[3] Gensler Europe, Thomas More Square, London, E1W 1YW, United Kingdom.

[4] B&K Hybrid Solutions, Haslams Lane, Alfreton Road, Derby, DE21 4TS, United Kingdom.

[5] Department of Civil, Environmental & Geomatic Engineering, University College London, London, WC1E 6BT, United Kingdom.

[a] a.yayla22@imperial.ac.uk, [b] augustin.danneaux@enpc.fr, [c] e.schurer@hotmail.co.uk, [d] m.gao20@imperial.ac.uk, [e] c.demirci14@alumni.imperial.ac.uk, [f] colin.rose@ucl.ac.uk, [g] s.vanewijk@ucl.ac.uk, [h] r.myers@imperial.ac.uk

* Corresponding author, Rupert J. Myers, E-mail address: r.myers@imperial.ac.uk.

This Supplementary Information S1 file includes:

Pages S1 to S114
Supplementary Results, Discussions, Limitations, and Methods
Supplementary Tables S1 to S4
Supplementary Figures S1 to S47
Supplementary Information S1 References

Other supporting materials for this manuscript include the following:

Data repository, Supplementary Information S2

Table of contents

Mitigating hidden climate change impacts of timber cities critically depends on proactive forest and waste management S1
Table of contents S2
List of tables S4
List of figures S5
S1. Supplementary results S20
S1.1. Global level S20
S1.1.1. Global warming potential S20
S1.1.2. Atmospheric CH_4 concentrations S24
S1.1.3. Sensitivity analysis based on Regionalized Model of Investment and Development (REMIND) integrated assessment model (IAM) scenarios S26
S1.1.3.1. Start-of-life emission projections by 2100 ..... S26
S1.1.3.2. Absolute global temperature potential S28
S1.1.3.3. Global potential warming potential S30
S1.1.4. Sensitivity analysis based on building lifespan S32
S1.1.4.1. Absolute global temperature potential S32
S1.1.4.2. Global warming potential S34
S1.1.5. Sensitivity analysis based on floor area per capita S36
S1.1.5.1. Absolute global temperature potential S37
S1.1.5.2. Global warming potential S39
S1.1.6. Sensitivity analysis based on forest rotation period S41
S1.1.6.1. Absolute global temperature potential S41
S1.1.6.2. Global warming potential S43
S1.1.7. Sensitivity analysis based on wood decay rate in landfills S45
S1.1.7.1. Absolute global temperature potential S45
S1.1.7.2. Global warming potential S47
S1.1.8. Future urban population growth and building area S49
S1.2. Regional level S50
S1.2.1. Absolute global temperature potential by region S50
S1.2.2. Global warming potential by region S51
S1.2.3. Southern Asia S52
S1.2.3.1. Absolute global temperature potential S52
S1.2.3.2. Global warming potential S53
S1.2.4. Western Africa S55
S1.2.4.1. Absolute global temperature potential S55
S1.2.4.2. Global warming potential S56
S1.2.5. Eastern Africa S58
S1.2.5.1. Absolute global temperature potential S58
S1.2.5.2. Global warming potential S59
S1.3. Country level S61
S1.3.1. Absolute global temperature potential by country S61
S1.3.2. Global warming potential by country S62
S1.3.3. India S63
S1.3.3.1. Absolute global temperature potential S63

S1.3.3.2. Global warming potential S64
S1.3.4. Nigeria S66
S1.3.4.1. Absolute global temperature potential S66
S1.3.4.2. Global warming potential S67
S1.3.5. United States of America S69
S1.3.5.1. Absolute global temperature potential S69
S1.3.5.2. Global warming potential S70
S1.3.6. Pakistan S72
S1.3.6.1. Absolute global temperature potential S72
S1.3.6.2. Global warming potential S73
S1.3.7. Democratic Republic of the Congo S75
S1.3.7.1. Absolute global temperature potential S75
S1.3.7.2. Global warming potential S76
S1.3.8. China S78
S1.3.8.1. Absolute global temperature potential S78
S1.3.8.2. Global warming potential S79
S1.3.9. Ethiopia S81
S1.3.9.1. Absolute global temperature potential S81
S1.3.9.2. Global warming potential S82
S1.4. Building level S84
S1.4.1. Atmospheric greenhouse gas concentration changes S84
S1.4.2. Absolute global temperature potential S87
S1.4.3. Global warming potential S89
S1.4.4. Carbonation sensitivity S92
S2. Supplementary discussion S93
S3. Supplementary limitations S94
S4. Supplementary methods S95
S4.1. Goal and scope S95
S4.2. Scenarios S95
S4.2.1. Start-of-life scenarios S96
S4.2.2. End-of-life scenarios S96
S4.2.3. End-of-life substitution credits S96
S4.3. Case study building S99
S4.4. Life cycle inventory S104
S4.4.1. Material quantities S104
S4.4.2. Start-of-life and end-of-life emissions S104
S4.5. Dynamic life cycle assessment S106
S4.5.1. Biogenic carbon sequestration S106
S4.5.2. Degradable organic carbon in landfills S106
S4.5.3. Carbonation S106
S4.5.4. Atmospheric decay of emissions S108
S4.5.5. Life cycle impact assessment S109
S4.5.5.1. Static global warming potential S109
S4.5.5.2. Dynamic global warming potential S109
S4.5.5.3. Absolute global temperature potential S110
References in this Supplementary Information S1 file S111

List of tables

Supplementary Table S1 | $GWP100_{dynamic}$ and $GWP100_{static}$ results per square meter of floor space for the case study building constructed in 2025 based on start-of-life and end-of-life urban building scenarios. The case study building has a 1,970 m^2 gross internal area with four storeys, and a lifespan of 100 years. The results are shown for SSP2. .... S89

Supplementary Table S2 | The urban building end-of-life scenarios and substitution credits modelled in this study. S98

Supplementary Table S3 | Insulation materials for the reinforced concrete structural design of the case study building, and U-values of building envelope. S102

Supplementary Table S4 | Insulation materials for the engineered timber structural design of the case study building, and U-values of building envelope. S103

List of figures

Supplementary Fig. S1 | Geographic breakdown of dynamic global warming potential (GWP) results of future cities based on start-of-life and end-of-life urban building scenarios for time horizon impacts of 20, 100, and 200 years. a $GWP100_{dynamic}$ ranges at a country-scale resolution for the BAU-C3 scenario, b $GWP100_{dynamic}$ ranges at a country-scale resolution for the EST-T6 scenario, c The ten countries with the highest $GWP20_{dynamic}$ results, d The ten countries with the highest $GWP100_{dynamic}$ results, e The ten countries with the highest $GWP200_{dynamic}$ results. Uncertainties are based on shared socioeconomic pathways (SSPs). For the world maps in a and b, results are shown for SSP2. For graphs in c, d, and e, large dots use SSP2, and error bars quantify uncertainty based on SSP1 and SSP5. Start-of-life scenarios for future urban buildings cover the construction period between 2025 and 2100: BAU, reinforced OPC concrete cities; LC^3, reinforced LC^3 concrete cities; EST, timber cities. End-of-life scenarios for future urban buildings cover the demolition period between 2125 and 2200: C1, concrete is partly recycled, and partly discarded to landfills; C2, concrete is recycled; C3, concrete is discarded to landfills; T1, engineered timber is partly recycled into lower-quality products, partly incinerated, and partly discarded to landfills; T2, engineered timber is incinerated for bioenergy; T3, engineered timber is reused as functionally equivalent products, and this removes the incentive for forest regrowth; T4, engineered timber is recycled into lower quality and/or value products, and this removes the incentive for forest regrowth; T5, engineered timber is partly reused, partly recycled into lower quality/value products, and forestry and land management practices are applied to sustain the forest regrowth; T6, engineered timber is discarded to landfills with direct LFGs release; T7, engineered timber is discarded to landfills, and LFGs are treated by enclosed flare; T8, engineered timber is discarded to landfills, and LFGs are captured and burned for energy production. S20

Supplementary Fig. S2 | Dynamic and static global warming potential (GWP) results of future cities based on start-of-life and end-of-life urban building scenarios for time horizon impacts of 20, 100 and 200 years. a $GWP20_{dynamic}$ and $GWP20_{static}$, b $GWP100_{dynamic}$ and $GWP100_{static}$, c $GWP200_{dynamic}$ and $GWP200_{static}$. Uncertainties are based on shared socioeconomic pathways (SSPs). The contribution analysis (i.e., heights of bars for each variable), red dots and texts for each scenario represent SSP2. Red error bars quantify uncertainty based on SSP1 and SSP5. Start-of-life scenarios for future urban buildings cover the construction period between 2025 and 2100: BAU, reinforced OPC concrete cities; LC^3, reinforced LC^3 concrete cities; EST, timber cities. End-of-life scenarios for future urban buildings cover the demolition period between 2125 and 2200: C1, concrete is partly recycled, and partly discarded to landfills; C2, concrete is recycled; C3, concrete is discarded to landfills; T1, engineered timber is partly recycled into lower-quality products, partly incinerated, and partly discarded to landfills; T2, engineered timber is incinerated for bioenergy; T3, engineered timber is reused as functionally equivalent products, and this removes the incentive for forest regrowth; T4, engineered timber is recycled into lower quality and/or value products, and this removes the incentive for forest regrowth; T5, engineered timber is partly reused, partly recycled into lower quality/value products, and forestry and land management practices are applied to sustain the forest regrowth; T6, engineered timber is discarded to landfills with direct LFGs release; T7, engineered timber is discarded to landfills, and LFGs are treated by enclosed flare; T8, engineered timber is discarded to landfills, and LFGs are captured and burned for energy production.. S22

Supplementary Fig. S3 | Atmospheric CH_4 concentrations between 2025 and 2325 based on future timber and reinforced concrete city scenarios. a BAU-C1, b BAU-C2, c BAU-C3, d LC^3-C1, e LC^3-C2, f LC^3-C3, g EST-T1, h EST-T2, i EST-T3, j EST-T4, k EST-T5, l EST-T6, m EST-T7, n EST-T8. The results are shown for SSP2. Start-of-life scenarios for future urban buildings cover the construction period between 2025 and 2100: BAU, reinforced OPC concrete cities; LC^3, reinforced LC^3 concrete cities; EST, timber cities. End-of-life scenarios for future urban buildings cover the demolition period between 2125 and 2200: C1, concrete is partly recycled, and partly discarded to landfills; C2, concrete is recycled; C3, concrete is discarded to landfills; T1, engineered timber is partly recycled into lower-quality products, partly incinerated, and partly discarded to landfills; T2, engineered timber is incinerated for bioenergy; T3, engineered timber is reused as functionally equivalent products, and this removes the incentive for forest regrowth; T4, engineered timber is recycled into lower quality and/or value products, and this removes the incentive for forest regrowth; T5, engineered timber is partly reused, partly recycled into lower quality/value products, and forestry and land management practices are applied to sustain the forest regrowth; T6, engineered timber is discarded to landfills with direct LFGs release; T7, engineered timber is discarded to landfills, and LFGs are treated by enclosed flare; T8, engineered timber is discarded to landfills, and LFGs are captured and burned for energy production. S24

Supplementary Fig. S4 | Start-of-life emission projections of the representative case study building in this study by 2100. Start-of-life CO_2 emissions based on a REMIND-SSPs-Base, b REMIND-SSPs-NDC, c REMIND-SSPs-PkBudg1150, d REMIND-SSPs-PkBudg500. Start-of-life CH_4 emissions based on e REMIND-SSPs-Base, f REMIND-SSPs-NDC, g REMIND-SSPs-PkBudg1150, h REMIND-SSPs-PkBudg500. Start-of-life N_2O emissions based on i REMIND-SSPs-Base, j REMIND-SSPs-NDC, k REMIND-SSPs-PkBudg1150, l REMIND-SSPs-PkBudg500. For each scenario, the solid line (SSP2), the dashed line (SSP1), and the dotted line (SSP5) represent shared socioeconomic pathways (SSPs), with the shaded area between these lines quantifying uncertainty. Start-of-life scenarios for future urban buildings cover the construction period between 2025 and 2100. REMIND-SSPs-Base represents a current-policies scenario reflecting existing climate policies without full implementation of announced pledges; REMIND-SSPs-NDC represents a climate policy scenario consistent with countries' nationally determined contributions (NDC); REMIND-SSPs-PkBudg1150 represents a climate policy scenario restricting cumulative CO_2 emissions to a budget of 1150 Gt CO_2, consistent with a ~2 °C target; REMIND-SSPs-PkBudg500 represents a climate policy scenario restricting cumulative CO_2 emissions to a budget of 500 Gt CO_2, consistent with a ~1.5 °C target. BAU, reinforced OPC concrete building; LC^3, reinforced LC^3 concrete building; EST, engineered structural timber building. S26

Supplementary Fig. S5 | Absolute global temperature potential (AGTP) of future cities between 2025 and 2325 based on start-of-life and end-of-life urban building scenarios, and various REMIND IAM scenarios. a A current-policies scenario reflecting existing climate policies without full implementation of announced pledges (REMIND-SSPs-Base), b A climate policy scenario consistent with countries' nationally determined contributions (NDC) (REMIND-SSPs-NDC), c A climate policy scenario restricting cumulative CO_2 emissions to a budget of 1150 Gt CO_2, consistent with a ~2 °C target (REMIND-SSPs-PkBudg1150), d A climate policy scenario restricting cumulative CO_2 emissions to a budget of 500 Gt CO_2, consistent with a ~1.5 °C target (REMIND-SSPs-PkBudg500). For each scenario, the solid line (SSP2), the dashed line (SSP1), and the dotted line (SSP5) represent shared socioeconomic pathways (SSPs), with the shaded area between these lines quantifying uncertainty. BAU, reinforced OPC concrete building; LC^3, reinforced LC^3 concrete building; EST, engineered structural timber building; C1, concrete is partly recycled, and partly discarded to landfills; C2, concrete is recycled; C3, concrete is discarded to landfills; T1, engineered timber is partly recycled into lower-quality products, partly incinerated, and partly discarded to landfills; T2, engineered timber is incinerated for bioenergy; T3, engineered timber is reused as functionally equivalent products, and this removes the incentive for forest regrowth; T4, engineered timber is recycled into lower quality and/or value products, and this removes the incentive for forest regrowth; T5, engineered timber is partly reused, partly recycled into lower quality/value products, and forestry and land management practices are applied to sustain the forest regrowth; T6, engineered timber is discarded to landfills with direct LFGs release; T7, engineered timber is discarded to landfills, and LFGs are treated by enclosed flare; T8, engineered timber is discarded to landfills, and LFGs are captured and burned for energy production. S28

Supplementary Fig. S6 | Dynamic and static global warming potential (GWP) results of future cities based on start-of-life and end-of-life urban building scenarios, and various REMIND IAM scenarios for the time horizon impact of 100 years ($GWP100_{dynamic}$ and $GWP100_{static}$). a A current-policies scenario reflecting existing climate policies without full implementation of announced pledges (REMIND-SSPs-Base), b A climate policy scenario consistent with countries' nationally determined contributions (NDC) (REMIND-SSPs-NDC), c A climate policy scenario restricting cumulative CO_2 emissions to a budget of 1150 Gt CO_2, consistent with a ~2 °C target (REMIND-SSPs-PkBudg1150), d A climate policy scenario restricting cumulative CO_2 emissions to a budget of 500 Gt CO_2, consistent with a ~1.5 °C target (REMIND-SSPs-PkBudg500). Uncertainties are based on shared socioeconomic pathways (SSPs). The contribution analysis (i.e., heights of bars for each variable), red dots and texts for each scenario represent SSP2. Red error bars quantify uncertainty based on SSP1 and SSP5. Start-of-life scenarios for future urban buildings cover the construction period between 2025 and 2100: BAU, reinforced OPC concrete cities; LC^3, reinforced LC^3 concrete cities; EST, timber cities. End-of-life scenarios for future urban buildings cover the demolition period between 2125 and 2200: C1, concrete is partly recycled, and partly discarded to landfills; C2, concrete is recycled; C3, concrete is discarded to landfills; T1, engineered timber is partly recycled into lower-quality products, partly incinerated, and partly discarded to landfills; T2, engineered timber is incinerated for bioenergy; T3, engineered timber is reused as functionally equivalent products, and this removes the incentive for forest regrowth; T4, engineered timber is recycled into lower quality and/or value products, and this removes the incentive for forest regrowth; T5, engineered timber is partly reused, partly recycled

into lower quality/value products, and forestry and land management practices are applied to sustain the forest regrowth; T6, engineered timber is discarded to landfills with direct LFGs release; T7, engineered timber is discarded to landfills, and LFGs are treated by enclosed flare; T8, engineered timber is discarded to landfills, and LFGs are captured and burned for energy production. S30

Supplementary Fig. S7 | Absolute global temperature potential (AGTP) of future cities between 2025 and 2325 based on start-of-life and end-of-life urban building scenarios, and various building lifespans. a Urban buildings with an average lifespan of 50 years, b Urban buildings with an average lifespan of 100 years, c Urban buildings with an average lifespan of 150 years For each scenario, the solid line (SSP2), the dashed line (SSP1), and the dotted line (SSP5) represent shared socioeconomic pathways (SSPs), with the shaded area between these lines quantifying uncertainty. BAU, reinforced OPC concrete building; LC^3, reinforced LC^3 concrete building; EST, engineered structural timber building; C1, concrete is partly recycled, and partly discarded to landfills; C2, concrete is recycled; C3, concrete is discarded to landfills; T1, engineered timber is partly recycled into lower-quality products, partly incinerated, and partly discarded to landfills; T2, engineered timber is incinerated for bioenergy; T3, engineered timber is reused as functionally equivalent products, and this removes the incentive for forest regrowth; T4, engineered timber is recycled into lower quality and/or value products, and this removes the incentive for forest regrowth; T5, engineered timber is partly reused, partly recycled into lower quality/value products, and forestry and land management practices are applied to sustain the forest regrowth; T6, engineered timber is discarded to landfills with direct LFGs release; T7, engineered timber is discarded to landfills, and LFGs are treated by enclosed flare; T8, engineered timber is discarded to landfills, and LFGs are captured and burned for energy production. S32

Supplementary Fig. S8 | Dynamic and static global warming potential (GWP) results of future cities based on start-of-life and end-of-life urban building scenarios, and various building lifespans for the time horizon impact of 100 years ($GWP100_{dynamic}$ and $GWP100_{static}$). a Urban buildings with an average lifespan of 50 years, b Urban buildings with an average lifespan of 100 years, c Urban buildings with an average lifespan of 150 years. Uncertainties are based on shared socioeconomic pathways (SSPs). The contribution analysis (i.e., heights of bars for each variable), red dots and texts for each scenario represent SSP2. Red error bars quantify uncertainty based on SSP1 and SSP5. Start-of-life scenarios for future urban buildings cover the construction period between 2025 and 2100: BAU, reinforced OPC concrete cities; LC^3, reinforced LC^3 concrete cities; EST, timber cities. End-of-life scenarios for future urban buildings cover the demolition period between 2125 and 2200: C1, concrete is partly recycled, and partly discarded to landfills; C2, concrete is recycled; C3, concrete is discarded to landfills; T1, engineered timber is partly recycled into lower-quality products, partly incinerated, and partly discarded to landfills; T2, engineered timber is incinerated for bioenergy; T3, engineered timber is reused as functionally equivalent products, and this removes the incentive for forest regrowth; T4, engineered timber is recycled into lower quality and/or value products, and this removes the incentive for forest regrowth; T5, engineered timber is partly reused, partly recycled into lower quality/value products, and forestry and land management practices are applied to sustain the forest regrowth; T6, engineered timber is discarded to landfills with direct LFGs release; T7, engineered timber is discarded to landfills, and LFGs are treated by enclosed flare; T8, engineered timber is discarded to landfills, and LFGs are captured and burned for energy production. S34

Supplementary Fig. S9 | Absolute global temperature potential (AGTP) of future cities between 2025 and 2325 based on start-of-life and end-of-life urban building scenarios, and various floor area per capita. a Urban buildings with an average per capita floor area of 9.2 m^2, b Urban buildings with an average per capita floor area of 30.0 m^2, c Urban buildings with an average per capita floor area of 79.1 m^2. For each scenario, the solid line (SSP2), the dashed line (SSP1), and the dotted line (SSP5) represent shared socioeconomic pathways (SSPs), with the shaded area between these lines quantifying uncertainty. BAU, reinforced OPC concrete building; LC^3, reinforced LC^3 concrete building; EST, engineered structural timber building; C1, concrete is partly recycled, and partly discarded to landfills; C2, concrete is recycled; C3, concrete is discarded to landfills; T1, engineered timber is partly recycled into lower-quality products, partly incinerated, and partly discarded to landfills; T2, engineered timber is incinerated for bioenergy; T3, engineered timber is reused as functionally equivalent products, and this removes the incentive for forest regrowth; T4, engineered timber is recycled into lower quality and/or value products, and this removes the incentive for forest regrowth; T5, engineered timber is partly reused, partly recycled into lower quality/value products, and forestry and land management practices are applied to sustain the forest regrowth; T6, engineered timber is discarded to landfills with direct LFGs release; T7, engineered timber is discarded to

landfills, and LFGs are treated by enclosed flare; T8, engineered timber is discarded to landfills, and LFGs are captured and burned for energy production... S37

Supplementary Fig. S10 | Dynamic and static global warming potential (GWP) results of future cities based on start-of-life and end-of-life urban building scenarios, and various floor area per capita for the time horizon impact of 100 years ($GWP100_{dynamic}$ and $GWP100_{static}$). a Urban buildings with an average per capita floor area of 9.2 m^2, b Urban buildings with an average per capita floor area of 30.0 m^2, c Urban buildings with an average per capita floor area of 79.1 m^2. Uncertainties are based on shared socioeconomic pathways (SSPs). The contribution analysis (i.e., heights of bars for each variable), red dots and texts for each scenario represent SSP2. Red error bars quantify uncertainty based on SSP1 and SSP5. Start-of-life scenarios for future urban buildings cover the construction period between 2025 and 2100: BAU, reinforced OPC concrete cities; LC^3, reinforced LC^3 concrete cities; EST, timber cities. End-of-life scenarios for future urban buildings cover the demolition period between 2125 and 2200: C1, concrete is partly recycled, and partly discarded to landfills; C2, concrete is recycled; C3, concrete is discarded to landfills; T1, engineered timber is partly recycled into lower-quality products, partly incinerated, and partly discarded to landfills; T2, engineered timber is incinerated for bioenergy; T3, engineered timber is reused as functionally equivalent products, and this removes the incentive for forest regrowth; T4, engineered timber is recycled into lower quality and/or value products, and this removes the incentive for forest regrowth; T5, engineered timber is partly reused, partly recycled into lower quality/value products, and forestry and land management practices are applied to sustain the forest regrowth; T6, engineered timber is discarded to landfills with direct LFGs release; T7, engineered timber is discarded to landfills, and LFGs are treated by enclosed flare; T8, engineered timber is discarded to landfills, and LFGs are captured and burned for energy production. S39

Supplementary Fig. S11 | Absolute global temperature potential (AGTP) of future cities between 2025 and 2325 based on start-of-life and end-of-life urban building scenarios, and various forest rotation periods. a An average forest rotation period of 40 years, b An average forest rotation period of 100 years, c An average forest rotation period of 160 years. For each scenario, the solid line (SSP2), the dashed line (SSP1), and the dotted line (SSP5) represent shared socioeconomic pathways (SSPs), with the shaded area between these lines quantifying uncertainty. BAU, reinforced OPC concrete building; LC^3, reinforced LC^3 concrete building; EST, engineered structural timber building; C1, concrete is partly recycled, and partly discarded to landfills; C2, concrete is recycled; C3, concrete is discarded to landfills; T1, engineered timber is partly recycled into lower-quality products, partly incinerated, and partly discarded to landfills; T2, engineered timber is incinerated for bioenergy; T3, engineered timber is reused as functionally equivalent products, and this removes the incentive for forest regrowth; T4, engineered timber is recycled into lower quality and/or value products, and this removes the incentive for forest regrowth; T5, engineered timber is partly reused, partly recycled into lower quality/value products, and forestry and land management practices are applied to sustain the forest regrowth; T6, engineered timber is discarded to landfills with direct LFGs release; T7, engineered timber is discarded to landfills, and LFGs are treated by enclosed flare; T8, engineered timber is discarded to landfills, and LFGs are captured and burned for energy production. S41

Supplementary Fig. S12 | Dynamic and static global warming potential (GWP) results of future cities based on start-of-life and end-of-life urban building scenarios, and various forest rotation periods for the time horizon impact of 100 years ($GWP100_{dynamic}$ and $GWP100_{static}$). a An average forest rotation period of 40 years, b An average forest rotation period of 100 years, c An average forest rotation period of 160 years. Uncertainties are based on shared socioeconomic pathways (SSPs). The contribution analysis (i.e., heights of bars for each variable), red dots and texts for each scenario represent SSP2. Red error bars quantify uncertainty based on SSP1 and SSP5. Start-of-life scenarios for future urban buildings cover the construction period between 2025 and 2100: BAU, reinforced OPC concrete cities; LC^3, reinforced LC^3 concrete cities; EST, timber cities. End-of-life scenarios for future urban buildings cover the demolition period between 2125 and 2200: C1, concrete is partly recycled, and partly discarded to landfills; C2, concrete is recycled; C3, concrete is discarded to landfills; T1, engineered timber is partly recycled into lower-quality products, partly incinerated, and partly discarded to landfills; T2, engineered timber is incinerated for bioenergy; T3, engineered timber is reused as functionally equivalent products, and this removes the incentive for forest regrowth; T4, engineered timber is recycled into lower quality and/or value products, and this removes the incentive for forest regrowth; T5, engineered timber is partly reused, partly recycled into lower quality/value products, and forestry and land management practices are applied to sustain the forest regrowth; T6, engineered timber is discarded to landfills with direct LFGs release; T7, engineered timber is

discarded to landfills, and LFGs are treated by enclosed flare; T8, engineered timber is discarded to landfills, and LFGs are captured and burned for energy production. S43

Supplementary Fig. S13 | Absolute global temperature potential (AGTP) of future cities between 2025 and 2325 based on start-of-life and end-of-life urban building scenarios, and various wood decay rates in landfills. a An average of 0.9% of the discarded wood is subject to decay in landfills, b An average of 10.5% of the discarded wood is subject to decay in landfills, c An average of 23.2% of the discarded wood is subject to decay in landfills, d An average of 50% of the discarded wood is subject to decay in landfills. For each scenario, the solid line (SSP2), the dashed line (SSP1), and the dotted line (SSP5) represent shared socioeconomic pathways (SSPs), with the shaded area between these lines quantifying uncertainty. BAU, reinforced OPC concrete building; LC^3, reinforced LC^3 concrete building; EST, engineered structural timber building; C1, concrete is partly recycled, and partly discarded to landfills; C2, concrete is recycled; C3, concrete is discarded to landfills; T1, engineered timber is partly recycled into lower-quality products, partly incinerated, and partly discarded to landfills; T2, engineered timber is incinerated for bioenergy; T3, engineered timber is reused as functionally equivalent products, and this removes the incentive for forest regrowth; T4, engineered timber is recycled into lower quality and/or value products, and this removes the incentive for forest regrowth; T5, engineered timber is partly reused, partly recycled into lower quality/value products, and forestry and land management practices are applied to sustain the forest regrowth; T6, engineered timber is discarded to landfills with direct LFGs release; T7, engineered timber is discarded to landfills, and LFGs are treated by enclosed flare; T8, engineered timber is discarded to landfills, and LFGs are captured and burned for energy production.. S45

Supplementary Fig. S14 | Dynamic and static global warming potential (GWP) results of future cities based on start-of-life and end-of-life urban building scenarios, and various wood decay rates in landfills for the time horizon impact of 100 years ($GWP100_{dynamic}$ and $GWP100_{static}$). a An average of 0.9% of the discarded wood is subject to decay in landfills, b An average of 10.5% of the discarded wood is subject to decay in landfills, c An average of 23.2% of the discarded wood is subject to decay in landfills, d An average of 50% of the discarded wood is subject to decay in landfills. Uncertainties are based on shared socioeconomic pathways (SSPs). The contribution analysis (i.e., heights of bars for each variable), red dots and texts for each scenario represent SSP2. Red error bars quantify uncertainty based on SSP1 and SSP5. Start-of-life scenarios for future urban buildings cover the construction period between 2025 and 2100: BAU, reinforced OPC concrete cities; LC^3, reinforced LC^3 concrete cities; EST, timber cities. End-of-life scenarios for future urban buildings cover the demolition period between 2125 and 2200: C1, concrete is partly recycled, and partly discarded to landfills; C2, concrete is recycled; C3, concrete is discarded to landfills; T1, engineered timber is partly recycled into lower-quality products, partly incinerated, and partly discarded to landfills; T2, engineered timber is incinerated for bioenergy; T3, engineered timber is reused as functionally equivalent products, and this removes the incentive for forest regrowth; T4, engineered timber is recycled into lower quality and/or value products, and this removes the incentive for forest regrowth; T5, engineered timber is partly reused, partly recycled into lower quality/value products, and forestry and land management practices are applied to sustain the forest regrowth; T6, engineered timber is discarded to landfills with direct LFGs release; T7, engineered timber is discarded to landfills, and LFGs are treated by enclosed flare; T8, engineered timber is discarded to landfills, and LFGs are captured and burned for energy production... S47

Supplementary Fig. S15 | Annual and cumulative future urban population growth and mid-rise residential and commercial urban building area projections. a Annual urban population growth, b Cumulative urban population growth, c Annual new urban building area, d Cumulative new urban building area. Building areas were calculated based on the urban population growth of the countries based on shared socioeconomic pathways (SSPs). SSP2 (solid line) represents the default pathway. SSP1 (dashed line) and SSP5 (dotted line) quantify uncertainty. All new urban population is assumed to live in buildings with a 30 m2/capita average floor area. S49

Supplementary Fig. S16 | Geographic breakdown of absolute global temperature potential (AGTP) of future cities in 2050, 2100, and 2100 based on start-of-life and end-of-life urban building scenarios. a AGTP of United Nations subregions in 2050, b AGTP of United Nations subregions in 2100, c AGTP of United Nations subregions in 2200. Uncertainties are based on shared socioeconomic pathways (SSPs). Large dots based on scenario colour represent SSP2, and error bars quantify uncertainty based on SSP1 and SSP5. Start-of-life scenarios for future urban buildings cover the construction period between 2025 and 2100: BAU, reinforced OPC concrete cities; LC^3, reinforced LC^3 concrete cities; EST, timber cities. End-of-life scenarios for future urban buildings cover the demolition period between 2125 and 2200: C1, concrete is partly recycled, and partly discarded to landfills; C2,

concrete is recycled; C3, concrete is discarded to landfills; T1, engineered timber is partly recycled into lower-quality products, partly incinerated, and partly discarded to landfills; T2, engineered timber is incinerated for bioenergy; T3, engineered timber is reused as functionally equivalent products, and this removes the incentive for forest regrowth; T4, engineered timber is recycled into lower quality and/or value products, and this removes the incentive for forest regrowth; T5, engineered timber is partly reused, partly recycled into lower quality/value products, and forestry and land management practices are applied to sustain the forest regrowth; T6, engineered timber is discarded to landfills with direct landfill gases (LFGs) release; T7, engineered timber is discarded to landfills, and LFGs are treated by enclosed flare; T8, engineered timber is discarded to landfills, and LFGs are captured and burned for energy production. S50

Supplementary Fig. S17 | Geographic breakdown of dynamic global warming potential (GWP) results of future cities based on start-of-life and end-of-life urban building scenarios for time horizon impacts of 20, 100, and 200 years. a $GWP20_{dynamic}$ results of United Nations subregions, b $GWP100_{dynamic}$ results of United Nations subregions, c $GWP200_{dynamic}$ results of United Nations subregions. Uncertainties are based on shared socioeconomic pathways (SSPs). Large dots based on scenario colour represent SSP2, and error bars quantify uncertainty based on SSP1 and SSP5. Start-of-life scenarios for future urban buildings cover the construction period between 2025 and 2100: BAU, reinforced OPC concrete cities; LC^3, reinforced LC^3 concrete cities; EST, timber cities. End-of-life scenarios for future urban buildings cover the demolition period between 2125 and 2200: C1, concrete is partly recycled, and partly discarded to landfills; C2, concrete is recycled; C3, concrete is discarded to landfills; T1, engineered timber is partly recycled into lower-quality products, partly incinerated, and partly discarded to landfills; T2, engineered timber is incinerated for bioenergy; T3, engineered timber is reused as functionally equivalent products, and this removes the incentive for forest regrowth; T4, engineered timber is recycled into lower quality and/or value products, and this removes the incentive for forest regrowth; T5, engineered timber is partly reused, partly recycled into lower quality/value products, and forestry and land management practices are applied to sustain the forest regrowth; T6, engineered timber is discarded to landfills with direct landfill gases (LFGs) release; T7, engineered timber is discarded to landfills, and LFGs are treated by enclosed flare; T8, engineered timber is discarded to landfills, and LFGs are captured and burned for energy production. S51

Supplementary Fig. S18 | Absolute global temperature potential (AGTP) of future cities for Southern Asia between 2025 and 2325 based on start-of-life and end-of-life urban building scenarios. For each scenario, the solid line (SSP2), the dashed line (SSP1), and the dotted line (SSP5) represent shared socioeconomic pathways (SSPs), with the shaded area between these lines quantifying uncertainty. Start-of-life scenarios for future urban buildings cover the construction period between 2025 and 2100: BAU, reinforced OPC concrete cities; LC^3, reinforced LC^3 concrete cities; EST, timber cities. End-of-life scenarios for future urban buildings cover the demolition period between 2125 and 2200: C1, concrete is partly recycled, and partly discarded to landfills; C2, concrete is recycled; C3, concrete is discarded to landfills; T1, engineered timber is partly recycled into lower-quality products, partly incinerated, and partly discarded to landfills; T2, engineered timber is incinerated for bioenergy; T3, engineered timber is reused as functionally equivalent products, and this removes the incentive for forest regrowth; T4, engineered timber is recycled into lower quality and/or value products, and this removes the incentive for forest regrowth; T5, engineered timber is partly reused, partly recycled into lower quality/value products, and forestry and land management practices are applied to sustain the forest regrowth; T6, engineered timber is discarded to landfills with direct landfill gases (LFGs) release; T7, engineered timber is discarded to landfills, and LFGs are treated by enclosed flare; T8, engineered timber is discarded to landfills, and LFGs are captured and burned for energy production. S52

Supplementary Fig. S19 | Dynamic and static global warming potential (GWP) results of future cities for Southern Asia based on start-of-life and end-of-life urban building scenarios for time horizon impacts of 20, 100 and 200 years. a $GWP20_{dynamic}$ and $GWP20_{static}$ results, b $GWP100_{dynamic}$ and $GWP100_{static}$ results, c $GWP200_{dynamic}$ and $GWP200_{static}$ results. Uncertainties are based on shared socioeconomic pathways (SSPs). The contribution analysis (i.e., heights of bars for each variable), red dots and texts for each scenario represent SSP2. Red error bars quantify uncertainty based on SSP1 and SSP5. Start-of-life scenarios for future urban buildings cover the construction period between 2025 and 2100: BAU, reinforced OPC concrete cities; LC^3, reinforced LC^3 concrete cities; EST, timber cities. End-of-life scenarios for future urban buildings cover the demolition period between 2125 and 2200: C1, concrete is partly recycled, and partly discarded to landfills; C2, concrete is recycled; C3, concrete is discarded to landfills; T1, engineered timber is partly recycled

into lower-quality products, partly incinerated, and partly discarded to landfills; T2, engineered timber is incinerated for bioenergy; T3, engineered timber is reused as functionally equivalent products, and this removes the incentive for forest regrowth; T4, engineered timber is recycled into lower quality and/or value products, and this removes the incentive for forest regrowth; T5, engineered timber is partly reused, partly recycled into lower quality/value products, and forestry and land management practices are applied to sustain the forest regrowth; T6, engineered timber is discarded to landfills with direct landfill gases (LFGs) release; T7, engineered timber is discarded to landfills, and LFGs are treated by enclosed flare; T8, engineered timber is discarded to landfills, and LFGs are captured and burned for energy production. S53

Supplementary Fig. S20 | Absolute global temperature potential (AGTP) of future cities for Western Africa between 2025 and 2325 based on start-of-life and end-of-life urban building scenarios. For each scenario, the solid line (SSP2), the dashed line (SSP1), and the dotted line (SSP5) represent shared socioeconomic pathways (SSPs), with the shaded area between these lines quantifying uncertainty. Start-of-life scenarios for future urban buildings cover the construction period between 2025 and 2100: BAU, reinforced OPC concrete cities; LC^3, reinforced LC^3 concrete cities; EST, timber cities. End-of-life scenarios for future urban buildings cover the demolition period between 2125 and 2200: C1, concrete is partly recycled, and partly discarded to landfills; C2, concrete is recycled; C3, concrete is discarded to landfills; T1, engineered timber is partly recycled into lower-quality products, partly incinerated, and partly discarded to landfills; T2, engineered timber is incinerated for bioenergy; T3, engineered timber is reused as functionally equivalent products, and this removes the incentive for forest regrowth; T4, engineered timber is recycled into lower quality and/or value products, and this removes the incentive for forest regrowth; T5, engineered timber is partly reused, partly recycled into lower quality/value products, and forestry and land management practices are applied to sustain the forest regrowth; T6, engineered timber is discarded to landfills with direct landfill gases (LFGs) release; T7, engineered timber is discarded to landfills, and LFGs are treated by enclosed flare; T8, engineered timber is discarded to landfills, and LFGs are captured and burned for energy production. S55

Supplementary Fig. S21 | Dynamic and static global warming potential (GWP) results of future cities for Western Africa based on start-of-life and end-of-life urban building scenarios for time horizon impacts of 20, 100 and 200 years. a $GWP20_{dynamic}$ and $GWP20_{static}$ results, b $GWP100_{dynamic}$ and $GWP100_{static}$ results, c $GWP200_{dynamic}$ and $GWP200_{static}$ results. Uncertainties are based on shared socioeconomic pathways (SSPs). The contribution analysis (i.e., heights of bars for each variable), red dots and texts for each scenario represent SSP2. Red error bars quantify uncertainty based on SSP1 and SSP5. Start-of-life scenarios for future urban buildings cover the construction period between 2025 and 2100: BAU, reinforced OPC concrete cities; LC^3, reinforced LC^3 concrete cities; EST, timber cities. End-of-life scenarios for future urban buildings cover the demolition period between 2125 and 2200: C1, concrete is partly recycled, and partly discarded to landfills; C2, concrete is recycled; C3, concrete is discarded to landfills; T1, engineered timber is partly recycled into lower-quality products, partly incinerated, and partly discarded to landfills; T2, engineered timber is incinerated for bioenergy; T3, engineered timber is reused as functionally equivalent products, and this removes the incentive for forest regrowth; T4, engineered timber is recycled into lower quality and/or value products, and this removes the incentive for forest regrowth; T5, engineered timber is partly reused, partly recycled into lower quality/value products, and forestry and land management practices are applied to sustain the forest regrowth; T6, engineered timber is discarded to landfills with direct landfill gases (LFGs) release; T7, engineered timber is discarded to landfills, and LFGs are treated by enclosed flare; T8, engineered timber is discarded to landfills, and LFGs are captured and burned for energy production. S56

Supplementary Fig. S22 | Absolute global temperature potential (AGTP) of future cities for Eastern Africa between 2025 and 2325 based on start-of-life and end-of-life urban building scenarios. For each scenario, the solid line (SSP2), the dashed line (SSP1), and the dotted line (SSP5) represent shared socioeconomic pathways (SSPs), with the shaded area between these lines quantifying uncertainty. Start-of-life scenarios for future urban buildings cover the construction period between 2025 and 2100: BAU, reinforced OPC concrete cities; LC^3, reinforced LC^3 concrete cities; EST, timber cities. End-of-life scenarios for future urban buildings cover the demolition period between 2125 and 2200: C1, concrete is partly recycled, and partly discarded to landfills; C2, concrete is recycled; C3, concrete is discarded to landfills; T1, engineered timber is partly recycled into lower-quality products, partly incinerated, and partly discarded to landfills; T2, engineered timber is incinerated for bioenergy; T3, engineered timber is reused as functionally equivalent products, and this removes the incentive for forest regrowth; T4, engineered timber is recycled into lower quality and/or value products, and

this removes the incentive for forest regrowth; T5, engineered timber is partly reused, partly recycled into lower quality/value products, and forestry and land management practices are applied to sustain the forest regrowth; T6, engineered timber is discarded to landfills with direct landfill gases (LFGs) release; T7, engineered timber is discarded to landfills, and LFGs are treated by enclosed flare; T8, engineered timber is discarded to landfills, and LFGs are captured and burned for energy production. S58

Supplementary Fig. S23 | Dynamic and static global warming potential (GWP) results of future cities for Eastern Africa based on start-of-life and end-of-life urban building scenarios for time horizon impacts of 20, 100 and 200 years. a $GWP20_{dynamic}$ and $GWP20_{static}$ results, b $GWP100_{dynamic}$ and $GWP100_{static}$ results, c $GWP200_{dynamic}$ and $GWP200_{static}$ results. Uncertainties are based on shared socioeconomic pathways (SSPs). The contribution analysis (i.e., heights of bars for each variable), red dots and texts for each scenario represent SSP2. Red error bars quantify uncertainty based on SSP1 and SSP5. Start-of-life scenarios for future urban buildings cover the construction period between 2025 and 2100: BAU, reinforced OPC concrete cities; LC^3, reinforced LC^3 concrete cities; EST, timber cities. End-of-life scenarios for future urban buildings cover the demolition period between 2125 and 2200: C1, concrete is partly recycled, and partly discarded to landfills; C2, concrete is recycled; C3, concrete is discarded to landfills; T1, engineered timber is partly recycled into lower-quality products, partly incinerated, and partly discarded to landfills; T2, engineered timber is incinerated for bioenergy; T3, engineered timber is reused as functionally equivalent products, and this removes the incentive for forest regrowth; T4, engineered timber is recycled into lower quality and/or value products, and this removes the incentive for forest regrowth; T5, engineered timber is partly reused, partly recycled into lower quality/value products, and forestry and land management practices are applied to sustain the forest regrowth; T6, engineered timber is discarded to landfills with direct landfill gases (LFGs) release; T7, engineered timber is discarded to landfills, and LFGs are treated by enclosed flare; T8, engineered timber is discarded to landfills, and LFGs are captured and burned for energy production. S59

Supplementary Fig. S24 | Geographic breakdown of absolute global temperature potential (AGTP) of future cities in 2050, 2100, and 2100 based on start-of-life and end-of-life urban building scenarios. a The twenty-one countries with the highest AGTP in 2050, b The twenty-one countries with the highest AGTP in 2100, c The twenty-one countries with the highest AGTP in 2200. Uncertainties are based on shared socioeconomic pathways (SSPs). Large dots based on scenario colour represent SSP2, and error bars quantify uncertainty based on SSP1 and SSP5. Start-of-life scenarios for future urban buildings cover the construction period between 2025 and 2100: BAU, reinforced OPC concrete cities; LC^3, reinforced LC^3 concrete cities; EST, timber cities. End-of-life scenarios for future urban buildings cover the demolition period between 2125 and 2200: C1, concrete is partly recycled, and partly discarded to landfills; C2, concrete is recycled; C3, concrete is discarded to landfills; T1, engineered timber is partly recycled into lower-quality products, partly incinerated, and partly discarded to landfills; T2, engineered timber is incinerated for bioenergy; T3, engineered timber is reused as functionally equivalent products, and this removes the incentive for forest regrowth; T4, engineered timber is recycled into lower quality and/or value products, and this removes the incentive for forest regrowth; T5, engineered timber is partly reused, partly recycled into lower quality/value products, and forestry and land management practices are applied to sustain the forest regrowth; T6, engineered timber is discarded to landfills with direct landfill gases (LFGs) release; T7, engineered timber is discarded to landfills, and LFGs are treated by enclosed flare; T8, engineered timber is discarded to landfills, and LFGs are captured and burned for energy production. S61

Supplementary Fig. S25 | Geographic breakdown of dynamic global warming potential (GWP) results of future cities based on start-of-life and end-of-life urban building scenarios for time horizon impacts of 20, 100, and 200 years. a The twenty-one countries with the highest $GWP20_{dynamic}$ results, b The twenty-one countries with the highest $GWP100_{dynamic}$ results, c The twenty-one countries with the highest $GWP200_{dynamic}$ results. Uncertainties are based on shared socioeconomic pathways (SSPs). Large dots based on scenario colour represent SSP2, and error bars quantify uncertainty based on SSP1 and SSP5. Start-of-life scenarios for future urban buildings cover the construction period between 2025 and 2100: BAU, reinforced OPC concrete cities; LC^3, reinforced LC^3 concrete cities; EST, timber cities. End-of-life scenarios for future urban buildings cover the demolition period between 2125 and 2200: C1, concrete is partly recycled, and partly discarded to landfills; C2, concrete is recycled; C3, concrete is discarded to landfills; T1, engineered timber is partly recycled into lower-quality products, partly incinerated, and partly discarded to landfills; T2, engineered timber is incinerated for bioenergy; T3, engineered timber is reused as functionally equivalent products, and this removes the incentive for forest regrowth; T4, engineered timber is recycled into lower quality

and/or value products, and this removes the incentive for forest regrowth; T5, engineered timber is partly reused, partly recycled into lower quality/value products, and forestry and land management practices are applied to sustain the forest regrowth; T6, engineered timber is discarded to landfills with direct landfill gases (LFGs) release; T7, engineered timber is discarded to landfills, and LFGs are treated by enclosed flare; T8, engineered timber is discarded to landfills, and LFGs are captured and burned for energy production. S62

Supplementary Fig. S26 | Absolute global temperature potential (AGTP) of future cities for India between 2025 and 2325 based on start-of-life and end-of-life urban building scenarios. For each scenario, the solid line (SSP2), the dashed line (SSP1), and the dotted line (SSP5) represent shared socioeconomic pathways (SSPs), with the shaded area between these lines quantifying uncertainty. Start-of-life scenarios for future urban buildings cover the construction period between 2025 and 2100: BAU, reinforced OPC concrete cities; LC^3, reinforced LC^3 concrete cities; EST, timber cities. End-of-life scenarios for future urban buildings cover the demolition period between 2125 and 2200: C1, concrete is partly recycled, and partly discarded to landfills; C2, concrete is recycled; C3, concrete is discarded to landfills; T1, engineered timber is partly recycled into lower-quality products, partly incinerated, and partly discarded to landfills; T2, engineered timber is incinerated for bioenergy; T3, engineered timber is reused as functionally equivalent products, and this removes the incentive for forest regrowth; T4, engineered timber is recycled into lower quality and/or value products, and this removes the incentive for forest regrowth; T5, engineered timber is partly reused, partly recycled into lower quality/value products, and forestry and land management practices are applied to sustain the forest regrowth; T6, engineered timber is discarded to landfills with direct landfill gases (LFGs) release; T7, engineered timber is discarded to landfills, and LFGs are treated by enclosed flare; T8, engineered timber is discarded to landfills, and LFGs are captured and burned for energy production. S63

Supplementary Fig. S27 | Dynamic and static global warming potential (GWP) results of future cities for India based on start-of-life and end-of-life urban building scenarios for time horizon impacts of 20, 100 and 200 years. a $GWP20_{dynamic}$ and $GWP20_{static}$ results, b $GWP100_{dynamic}$ and $GWP100_{static}$ results, c $GWP200_{dynamic}$ and $GWP200_{static}$ results. Uncertainties are based on shared socioeconomic pathways (SSPs). The contribution analysis (i.e., heights of bars for each variable), red dots and texts for each scenario represent SSP2. Red error bars quantify uncertainty based on SSP1 and SSP5. Start-of-life scenarios for future urban buildings cover the construction period between 2025 and 2100: BAU, reinforced OPC concrete cities; LC^3, reinforced LC^3 concrete cities; EST, timber cities. End-of-life scenarios for future urban buildings cover the demolition period between 2125 and 2200: C1, concrete is partly recycled, and partly discarded to landfills; C2, concrete is recycled; C3, concrete is discarded to landfills; T1, engineered timber is partly recycled into lower-quality products, partly incinerated, and partly discarded to landfills; T2, engineered timber is incinerated for bioenergy; T3, engineered timber is reused as functionally equivalent products, and this removes the incentive for forest regrowth; T4, engineered timber is recycled into lower quality and/or value products, and this removes the incentive for forest regrowth; T5, engineered timber is partly reused, partly recycled into lower quality/value products, and forestry and land management practices are applied to sustain the forest regrowth; T6, engineered timber is discarded to landfills with direct landfill gases (LFGs) release; T7, engineered timber is discarded to landfills, and LFGs are treated by enclosed flare; T8, engineered timber is discarded to landfills, and LFGs are captured and burned for energy production. S64

Supplementary Fig. S28 | Absolute global temperature potential (AGTP) of future cities for Nigeria between 2025 and 2325 based on start-of-life and end-of-life urban building scenarios. For each scenario, the solid line (SSP2), the dashed line (SSP1), and the dotted line (SSP5) represent shared socioeconomic pathways (SSPs), with the shaded area between these lines quantifying uncertainty. Start-of-life scenarios for future urban buildings cover the construction period between 2025 and 2100: BAU, reinforced OPC concrete cities; LC^3, reinforced LC^3 concrete cities; EST, timber cities. End-of-life scenarios for future urban buildings cover the demolition period between 2125 and 2200: C1, concrete is partly recycled, and partly discarded to landfills; C2, concrete is recycled; C3, concrete is discarded to landfills; T1, engineered timber is partly recycled into lower-quality products, partly incinerated, and partly discarded to landfills; T2, engineered timber is incinerated for bioenergy; T3, engineered timber is reused as functionally equivalent products, and this removes the incentive for forest regrowth; T4, engineered timber is recycled into lower quality and/or value products, and this removes the incentive for forest regrowth; T5, engineered timber is partly reused, partly recycled into lower quality/value products, and forestry and land management practices are applied to sustain the forest regrowth; T6, engineered timber is discarded to landfills with direct landfill gases (LFGs)

release; T7, engineered timber is discarded to landfills, and LFGs are treated by enclosed flare; T8, engineered timber is discarded to landfills, and LFGs are captured and burned for energy production. S66

Supplementary Fig. S29 | Dynamic and static global warming potential (GWP) results of future cities for Nigeria based on start-of-life and end-of-life urban building scenarios for time horizon impacts of 20, 100 and 200 years. a $GWP20_{dynamic}$ and $GWP20_{static}$ results, b $GWP100_{dynamic}$ and $GWP100_{static}$ results, c $GWP200_{dynamic}$ and $GWP200_{static}$ results. Uncertainties are based on shared socioeconomic pathways (SSPs). The contribution analysis (i.e., heights of bars for each variable), red dots and texts for each scenario represent SSP2. Red error bars quantify uncertainty based on SSP1 and SSP5. Start-of-life scenarios for future urban buildings cover the construction period between 2025 and 2100: BAU, reinforced OPC concrete cities; LC^3, reinforced LC^3 concrete cities; EST, timber cities. End-of-life scenarios for future urban buildings cover the demolition period between 2125 and 2200: C1, concrete is partly recycled, and partly discarded to landfills; C2, concrete is recycled; C3, concrete is discarded to landfills; T1, engineered timber is partly recycled into lower-quality products, partly incinerated, and partly discarded to landfills; T2, engineered timber is incinerated for bioenergy; T3, engineered timber is reused as functionally equivalent products, and this removes the incentive for forest regrowth; T4, engineered timber is recycled into lower quality and/or value products, and this removes the incentive for forest regrowth; T5, engineered timber is partly reused, partly recycled into lower quality/value products, and forestry and land management practices are applied to sustain the forest regrowth; T6, engineered timber is discarded to landfills with direct landfill gases (LFGs) release; T7, engineered timber is discarded to landfills, and LFGs are treated by enclosed flare; T8, engineered timber is discarded to landfills, and LFGs are captured and burned for energy production. S67

Supplementary Fig. S30 | Absolute global temperature potential (AGTP) of future cities for the United States of America between 2025 and 2325 based on start-of-life and end-of-life urban building scenarios. For each scenario, the solid line (SSP2), the dashed line (SSP1), and the dotted line (SSP5) represent shared socioeconomic pathways (SSPs), with the shaded area between these lines quantifying uncertainty. Start-of-life scenarios for future urban buildings cover the construction period between 2025 and 2100: BAU, reinforced OPC concrete cities; LC^3, reinforced LC^3 concrete cities; EST, timber cities. End-of-life scenarios for future urban buildings cover the demolition period between 2125 and 2200: C1, concrete is partly recycled, and partly discarded to landfills; C2, concrete is recycled; C3, concrete is discarded to landfills; T1, engineered timber is partly recycled into lower-quality products, partly incinerated, and partly discarded to landfills; T2, engineered timber is incinerated for bioenergy; T3, engineered timber is reused as functionally equivalent products, and this removes the incentive for forest regrowth; T4, engineered timber is recycled into lower quality and/or value products, and this removes the incentive for forest regrowth; T5, engineered timber is partly reused, partly recycled into lower quality/value products, and forestry and land management practices are applied to sustain the forest regrowth; T6, engineered timber is discarded to landfills with direct landfill gases (LFGs) release; T7, engineered timber is discarded to landfills, and LFGs are treated by enclosed flare; T8, engineered timber is discarded to landfills, and LFGs are captured and burned for energy production. S69

Supplementary Fig. S31 | Dynamic and static global warming potential (GWP) results of future cities for the United States of America based on start-of-life and end-of-life urban building scenarios for time horizon impacts of 20, 100 and 200 years. a $GWP20_{dynamic}$ and $GWP20_{static}$ results, b $GWP100_{dynamic}$ and $GWP100_{static}$ results, c $GWP200_{dynamic}$ and $GWP200_{static}$ results. Uncertainties are based on shared socioeconomic pathways (SSPs). The contribution analysis (i.e., heights of bars for each variable), red dots and texts for each scenario represent SSP2. Red error bars quantify uncertainty based on SSP1 and SSP5. Start-of-life scenarios for future urban buildings cover the construction period between 2025 and 2100: BAU, reinforced OPC concrete cities; LC^3, reinforced LC^3 concrete cities; EST, timber cities. End-of-life scenarios for future urban buildings cover the demolition period between 2125 and 2200: C1, concrete is partly recycled, and partly discarded to landfills; C2, concrete is recycled; C3, concrete is discarded to landfills; T1, engineered timber is partly recycled into lower-quality products, partly incinerated, and partly discarded to landfills; T2, engineered timber is incinerated for bioenergy; T3, engineered timber is reused as functionally equivalent products, and this removes the incentive for forest regrowth; T4, engineered timber is recycled into lower quality and/or value products, and this removes the incentive for forest regrowth; T5, engineered timber is partly reused, partly recycled into lower quality/value products, and forestry and land management practices are applied to sustain the forest regrowth; T6, engineered timber is discarded to landfills with direct landfill gases (LFGs) release; T7, engineered timber is discarded to landfills, and LFGs are

treated by enclosed flare; T8, engineered timber is discarded to landfills, and LFGs are captured and burned for energy production. S70

Supplementary Fig. S32 | Absolute global temperature potential (AGTP) of future cities for Pakistan between 2025 and 2325 based on start-of-life and end-of-life urban building scenarios. For each scenario, the solid line (SSP2), the dashed line (SSP1), and the dotted line (SSP5) represent shared socioeconomic pathways (SSPs), with the shaded area between these lines quantifying uncertainty. Start-of-life scenarios for future urban buildings cover the construction period between 2025 and 2100: BAU, reinforced OPC concrete cities; LC^3, reinforced LC^3 concrete cities; EST, timber cities. End-of-life scenarios for future urban buildings cover the demolition period between 2125 and 2200: C1, concrete is partly recycled, and partly discarded to landfills; C2, concrete is recycled; C3, concrete is discarded to landfills; T1, engineered timber is partly recycled into lower-quality products, partly incinerated, and partly discarded to landfills; T2, engineered timber is incinerated for bioenergy; T3, engineered timber is reused as functionally equivalent products, and this removes the incentive for forest regrowth; T4, engineered timber is recycled into lower quality and/or value products, and this removes the incentive for forest regrowth; T5, engineered timber is partly reused, partly recycled into lower quality/value products, and forestry and land management practices are applied to sustain the forest regrowth; T6, engineered timber is discarded to landfills with direct landfill gases (LFGs) release; T7, engineered timber is discarded to landfills, and LFGs are treated by enclosed flare; T8, engineered timber is discarded to landfills, and LFGs are captured and burned for energy production. S72

Supplementary Fig. S33 | Dynamic and static global warming potential (GWP) results of future cities for Pakistan based on start-of-life and end-of-life urban building scenarios for time horizon impacts of 20, 100 and 200 years. a $GWP20_{dynamic}$ and $GWP20_{static}$ results, b $GWP100_{dynamic}$ and $GWP100_{static}$ results, c $GWP200_{dynamic}$ and $GWP200_{static}$ results. Uncertainties are based on shared socioeconomic pathways (SSPs). The contribution analysis (i.e., heights of bars for each variable), red dots and texts for each scenario represent SSP2. Red error bars quantify uncertainty based on SSP1 and SSP5. Start-of-life scenarios for future urban buildings cover the construction period between 2025 and 2100: BAU, reinforced OPC concrete cities; LC^3, reinforced LC^3 concrete cities; EST, timber cities. End-of-life scenarios for future urban buildings cover the demolition period between 2125 and 2200: C1, concrete is partly recycled, and partly discarded to landfills; C2, concrete is recycled; C3, concrete is discarded to landfills; T1, engineered timber is partly recycled into lower-quality products, partly incinerated, and partly discarded to landfills; T2, engineered timber is incinerated for bioenergy; T3, engineered timber is reused as functionally equivalent products, and this removes the incentive for forest regrowth; T4, engineered timber is recycled into lower quality and/or value products, and this removes the incentive for forest regrowth; T5, engineered timber is partly reused, partly recycled into lower quality/value products, and forestry and land management practices are applied to sustain the forest regrowth; T6, engineered timber is discarded to landfills with direct landfill gases (LFGs) release; T7, engineered timber is discarded to landfills, and LFGs are treated by enclosed flare; T8, engineered timber is discarded to landfills, and LFGs are captured and burned for energy production. S73

Supplementary Fig. S34 | Absolute global temperature potential (AGTP) of future cities for the Democratic Republic of the Congo between 2025 and 2325 based on start-of-life and end-of-life urban building scenarios. For each scenario, the solid line (SSP2), the dashed line (SSP1), and the dotted line (SSP5) represent shared socioeconomic pathways (SSPs), with the shaded area between these lines quantifying uncertainty. Start-of-life scenarios for future urban buildings cover the construction period between 2025 and 2100: BAU, reinforced OPC concrete cities; LC^3, reinforced LC^3 concrete cities; EST, timber cities. End-of-life scenarios for future urban buildings cover the demolition period between 2125 and 2200: C1, concrete is partly recycled, and partly discarded to landfills; C2, concrete is recycled; C3, concrete is discarded to landfills; T1, engineered timber is partly recycled into lower-quality products, partly incinerated, and partly discarded to landfills; T2, engineered timber is incinerated for bioenergy; T3, engineered timber is reused as functionally equivalent products, and this removes the incentive for forest regrowth; T4, engineered timber is recycled into lower quality and/or value products, and this removes the incentive for forest regrowth; T5, engineered timber is partly reused, partly recycled into lower quality/value products, and forestry and land management practices are applied to sustain the forest regrowth; T6, engineered timber is discarded to landfills with direct landfill gases (LFGs) release; T7, engineered timber is discarded to landfills, and LFGs are treated by enclosed flare; T8, engineered timber is discarded to landfills, and LFGs are captured and burned for energy production. S75

Supplementary Fig. S35 | Dynamic and static global warming potential (GWP) results of future cities for the Democratic Republic of the Congo based on start-of-life and end-of-life urban building scenarios for time horizon impacts of 20, 100 and 200 years. a $GWP20_{dynamic}$ and $GWP20_{static}$ results, b $GWP100_{dynamic}$ and $GWP100_{static}$ results, c $GWP200_{dynamic}$ and $GWP200_{static}$ results. Uncertainties are based on shared socioeconomic pathways (SSPs). The contribution analysis (i.e., heights of bars for each variable), red dots and texts for each scenario represent SSP2. Red error bars quantify uncertainty based on SSP1 and SSP5. Start-of-life scenarios for future urban buildings cover the construction period between 2025 and 2100: BAU, reinforced OPC concrete cities; LC^3, reinforced LC^3 concrete cities; EST, timber cities. End-of-life scenarios for future urban buildings cover the demolition period between 2125 and 2200: C1, concrete is partly recycled, and partly discarded to landfills; C2, concrete is recycled; C3, concrete is discarded to landfills; T1, engineered timber is partly recycled into lower-quality products, partly incinerated, and partly discarded to landfills; T2, engineered timber is incinerated for bioenergy; T3, engineered timber is reused as functionally equivalent products, and this removes the incentive for forest regrowth; T4, engineered timber is recycled into lower quality and/or value products, and this removes the incentive for forest regrowth; T5, engineered timber is partly reused, partly recycled into lower quality/value products, and forestry and land management practices are applied to sustain the forest regrowth; T6, engineered timber is discarded to landfills with direct landfill gases (LFGs) release; T7, engineered timber is discarded to landfills, and LFGs are treated by enclosed flare; T8, engineered timber is discarded to landfills, and LFGs are captured and burned for energy production. S76

Supplementary Fig. S36 | Absolute global temperature potential (AGTP) of future cities for China between 2025 and 2325 based on start-of-life and end-of-life urban building scenarios. For each scenario, the solid line (SSP2), the dashed line (SSP1), and the dotted line (SSP5) represent shared socioeconomic pathways (SSPs), with the shaded area between these lines quantifying uncertainty. Start-of-life scenarios for future urban buildings cover the construction period between 2025 and 2100: BAU, reinforced OPC concrete cities; LC^3, reinforced LC^3 concrete cities; EST, timber cities. End-of-life scenarios for future urban buildings cover the demolition period between 2125 and 2200: C1, concrete is partly recycled, and partly discarded to landfills; C2, concrete is recycled; C3, concrete is discarded to landfills; T1, engineered timber is partly recycled into lower-quality products, partly incinerated, and partly discarded to landfills; T2, engineered timber is incinerated for bioenergy; T3, engineered timber is reused as functionally equivalent products, and this removes the incentive for forest regrowth; T4, engineered timber is recycled into lower quality and/or value products, and this removes the incentive for forest regrowth; T5, engineered timber is partly reused, partly recycled into lower quality/value products, and forestry and land management practices are applied to sustain the forest regrowth; T6, engineered timber is discarded to landfills with direct landfill gases (LFGs) release; T7, engineered timber is discarded to landfills, and LFGs are treated by enclosed flare; T8, engineered timber is discarded to landfills, and LFGs are captured and burned for energy production. S78

Supplementary Fig. S37 | Dynamic and static global warming potential (GWP) results of future cities for China based on start-of-life and end-of-life urban building scenarios for time horizon impacts of 20, 100 and 200 years. a $GWP20_{dynamic}$ and $GWP20_{static}$ results, b $GWP100_{dynamic}$ and $GWP100_{static}$ results, c $GWP200_{dynamic}$ and $GWP200_{static}$ results. Uncertainties are based on shared socioeconomic pathways (SSPs). The contribution analysis (i.e., heights of bars for each variable), red dots and texts for each scenario represent SSP2. Red error bars quantify uncertainty based on SSP1 and SSP5. Start-of-life scenarios for future urban buildings cover the construction period between 2025 and 2100: BAU, reinforced OPC concrete cities; LC^3, reinforced LC^3 concrete cities; EST, timber cities. End-of-life scenarios for future urban buildings cover the demolition period between 2125 and 2200: C1, concrete is partly recycled, and partly discarded to landfills; C2, concrete is recycled; C3, concrete is discarded to landfills; T1, engineered timber is partly recycled into lower-quality products, partly incinerated, and partly discarded to landfills; T2, engineered timber is incinerated for bioenergy; T3, engineered timber is reused as functionally equivalent products, and this removes the incentive for forest regrowth; T4, engineered timber is recycled into lower quality and/or value products, and this removes the incentive for forest regrowth; T5, engineered timber is partly reused, partly recycled into lower quality/value products, and forestry and land management practices are applied to sustain the forest regrowth; T6, engineered timber is discarded to landfills with direct landfill gases (LFGs) release; T7, engineered timber is discarded to landfills, and LFGs are treated by enclosed flare; T8, engineered timber is discarded to landfills, and LFGs are captured and burned for energy production. S79

Supplementary Fig. S38 | Absolute global temperature potential (AGTP) of future cities for Ethiopia between 2025 and 2325 based on start-of-life and end-of-life urban building scenarios. For each scenario, the solid line (SSP2), the dashed line (SSP1), and the dotted line (SSP5) represent shared socioeconomic pathways (SSPs), with the shaded area between these lines quantifying uncertainty. Start-of-life scenarios for future urban buildings cover the construction period between 2025 and 2100: BAU, reinforced OPC concrete cities; LC^3, reinforced LC^3 concrete cities; EST, timber cities. End-of-life scenarios for future urban buildings cover the demolition period between 2125 and 2200: C1, concrete is partly recycled, and partly discarded to landfills; C2, concrete is recycled; C3, concrete is discarded to landfills; T1, engineered timber is partly recycled into lower-quality products, partly incinerated, and partly discarded to landfills; T2, engineered timber is incinerated for bioenergy; T3, engineered timber is reused as functionally equivalent products, and this removes the incentive for forest regrowth; T4, engineered timber is recycled into lower quality and/or value products, and this removes the incentive for forest regrowth; T5, engineered timber is partly reused, partly recycled into lower quality/value products, and forestry and land management practices are applied to sustain the forest regrowth; T6, engineered timber is discarded to landfills with direct landfill gases (LFGs) release; T7, engineered timber is discarded to landfills, and LFGs are treated by enclosed flare; T8, engineered timber is discarded to landfills, and LFGs are captured and burned for energy production. S81

Supplementary Fig. S39 | Dynamic and static global warming potential (GWP) results of future cities for Ethiopia based on start-of-life and end-of-life urban building scenarios for time horizon impacts of 20, 100 and 200 years. a $GWP20_{dynamic}$ and $GWP20_{static}$ results, b $GWP100_{dynamic}$ and $GWP100_{static}$ results, c $GWP200_{dynamic}$ and $GWP200_{static}$ results. Uncertainties are based on shared socioeconomic pathways (SSPs). The contribution analysis (i.e., heights of bars for each variable), red dots and texts for each scenario represent SSP2. Red error bars quantify uncertainty based on SSP1 and SSP5. Start-of-life scenarios for future urban buildings cover the construction period between 2025 and 2100: BAU, reinforced OPC concrete cities; LC^3, reinforced LC^3 concrete cities; EST, timber cities. End-of-life scenarios for future urban buildings cover the demolition period between 2125 and 2200: C1, concrete is partly recycled, and partly discarded to landfills; C2, concrete is recycled; C3, concrete is discarded to landfills; T1, engineered timber is partly recycled into lower-quality products, partly incinerated, and partly discarded to landfills; T2, engineered timber is incinerated for bioenergy; T3, engineered timber is reused as functionally equivalent products, and this removes the incentive for forest regrowth; T4, engineered timber is recycled into lower quality and/or value products, and this removes the incentive for forest regrowth; T5, engineered timber is partly reused, partly recycled into lower quality/value products, and forestry and land management practices are applied to sustain the forest regrowth; T6, engineered timber is discarded to landfills with direct landfill gases (LFGs) release; T7, engineered timber is discarded to landfills, and LFGs are treated by enclosed flare; T8, engineered timber is discarded to landfills, and LFGs are captured and burned for energy production. S82

Supplementary Fig. S40 | Atmospheric CO_2 concentrations over time based on start-of-life and end-of-life scenarios of the case study building constructed in 2025. a BAU-C1 scenario, b LC^3-C1 scenario, c EST-T2 scenario, d EST-T4 scenario. The case study building has a 1,970 m^2 gross internal area with four storeys, and a lifespan of 100 years. The results are shown for SSP2. BAU, reinforced OPC concrete building; LC^3, reinforced LC^3 concrete building; EST, engineered structural timber building; C1, concrete is partly recycled, and partly discarded to landfills; T2, engineered timber is incinerated for bioenergy; T4, engineered timber is recycled into lower quality and/or value products, and this removes the incentive for forest regrowth. S85

Supplementary Fig. S41 | Atmospheric CH_4 concentrations over time based on start-of-life and end-of-life scenarios of the case study building. a EST-T6 scenario, b EST-T8 scenario. The case study building has a 1,970 m^2 gross internal area with four storeys, and a lifespan of 100 years. The results are shown for SSP2. EST, engineered structural timber building; T6, engineered timber is discarded to landfills with direct landfill gases (LFGs) release; T8, engineered timber is discarded to landfills, and LFGs are captured and burned for energy production. S86

Supplementary Fig. S42 | Absolute global temperature potential (AGTP) of the case study building constructed in 2025 based on start-of-life and end-of-life urban building scenarios. The case study building has a 1,970 m^2 gross internal area with four storeys, and a lifespan of 100 years. For each scenario, the solid line (SSP2), the dashed line (SSP1), and the dotted line (SSP5) represent shared socioeconomic pathways (SSPs), with the shaded area between these lines quantifying uncertainty. BAU, reinforced OPC concrete building; LC^3, reinforced LC^3 concrete building; EST, engineered structural timber building; C1, concrete is partly recycled, and partly discarded to landfills; C2,

concrete is recycled; C3, concrete is discarded to landfills; T1, engineered timber is partly recycled into lower-quality products, partly incinerated, and partly discarded to landfills; T2, engineered timber is incinerated for bioenergy; T3, engineered timber is reused as functionally equivalent products, and this removes the incentive for forest regrowth; T4, engineered timber is recycled into lower quality and/or value products, and this removes the incentive for forest regrowth; T5, engineered timber is partly reused, partly recycled into lower quality/value products, and forestry and land management practices are applied to sustain the forest regrowth; T6, engineered timber is discarded to landfills with direct landfill gases (LFGs) release; T7, engineered timber is discarded to landfills, and LFGs are treated by enclosed flare; T8, engineered timber is discarded to landfills, and LFGs are captured and burned for energy production. S87

Supplementary Fig. S43 | Dynamic and static global warming potential (GWP) results of the case study building constructed in 2025 based on start-of-life and end-of-life urban building scenarios for time horizon impacts of 20, 100 and 200 years. a $GWP20_{dynamic}$ and $GWP20_{static}$ results, b $GWP100_{dynamic}$ and $GWP100_{static}$ results, c $GWP200_{dynamic}$ and $GWP200_{static}$ results. The case study building has a 1,970 m^2 gross internal area with four storeys, and a lifespan of 100 years. Uncertainties are based on shared socioeconomic pathways (SSPs). The contribution analysis (i.e., heights of bars for each variable), red dots and texts for each scenario represent SSP2. Red error bars quantify uncertainty based on SSP1 and SSP5. BAU, reinforced OPC concrete building; LC^3, reinforced LC^3 concrete building; EST, engineered structural timber building; C1, concrete is partly recycled, and partly discarded to landfills; C2, concrete is recycled; C3, concrete is discarded to landfills; T1, engineered timber is partly recycled into lower-quality products, partly incinerated, and partly discarded to landfills; T2, engineered timber is incinerated for bioenergy; T3, engineered timber is reused as functionally equivalent products, and this removes the incentive for forest regrowth; T4, engineered timber is recycled into lower quality and/or value products, and this removes the incentive for forest regrowth; T5, engineered timber is partly reused, partly recycled into lower quality/value products, and forestry and land management practices are applied to sustain the forest regrowth; T6, engineered timber is discarded to landfills with direct landfill gases (LFGs) release; T7, engineered timber is discarded to landfills, and LFGs are treated by enclosed flare; T8, engineered timber is discarded to landfills, and LFGs are captured and burned for energy production. S90

Supplementary Fig. S44 | Absolute global temperature potential (AGTP) of the case study building constructed in 2025 based on start-of-life and end-of-life urban building scenarios. The case study building has a 1,970 m^2 gross internal area with four storeys, and a lifespan of 100 years. The results are shown for SSP2. Solid lines represent that concrete rubble is exposed to air for 3 months after demolition. Dashed lines represent that concrete rubble is exposed to air for 5 years after demolition. The dotted lines represent that concrete rubble is exposed to air for 10 years after demolition. The results are shown for SSP2 greenhouse gas concentrations. BAU, reinforced OPC concrete building; LC^3, reinforced LC^3 concrete building; EST, engineered structural timber building; C1, concrete is partly recycled, and partly discarded to landfills; C2, concrete is recycled; C3, concrete is discarded to landfills; T1, engineered timber is partly recycled into lower-quality products, partly incinerated, and partly discarded to landfills; T2, engineered timber is incinerated for bioenergy; T3, engineered timber is reused as functionally equivalent products, and this removes the incentive for forest regrowth; T4, engineered timber is recycled into lower quality and/or value products, and this removes the incentive for forest regrowth; T5, engineered timber is partly reused, partly recycled into lower quality/value products, and forestry and land management practices are applied to sustain the forest regrowth; T6, engineered timber is discarded to landfills with direct landfill gases (LFGs) release; T7, engineered timber is discarded to landfills, and LFGs are treated by enclosed flare; T8, engineered timber is discarded to landfills, and LFGs are captured and burned for energy production. S92

Supplementary Fig. S45 | Schematic visualisation for the reinforced concrete structural design of the case study building.. S100

Supplementary Fig. S46 | Schematic visualisation for the engineered timber structural design of the case study building.. S101

Supplementary Fig. S47 | Start-of-life emission projections of the representative case study building in this study by 2100. a Start-of-life CO_2 emissions, b Start-of-life CH_4 emissions, c Start-of-life N_2O emissions. The graphs show the results for the REMIND-SSPs-Base scenario which represents a current-policies scenario reflecting existing climate policies without full implementation of announced pledges. The main results of this study (i.e., AGTP, GWPs, Atmospheric GHG level results presented in the main text) uses REMIND-SSPs-Base scenario start-of-life and end-of-life emissions. For each scenario, the solid line (SSP2), the dashed line (SSP1), and the dotted line (SSP5) represent shared

socioeconomic pathways (SSPs), with the shaded area between these lines quantifying uncertainty. Start-of-life scenarios for future urban buildings cover the construction period between 2025 and 2100. BAU, reinforced OPC concrete building; LC^3, reinforced LC^3 concrete building; EST, engineered structural timber building. S105

S1. Supplementary results

S1.1. Global level

S1.1.1. Global warming potential

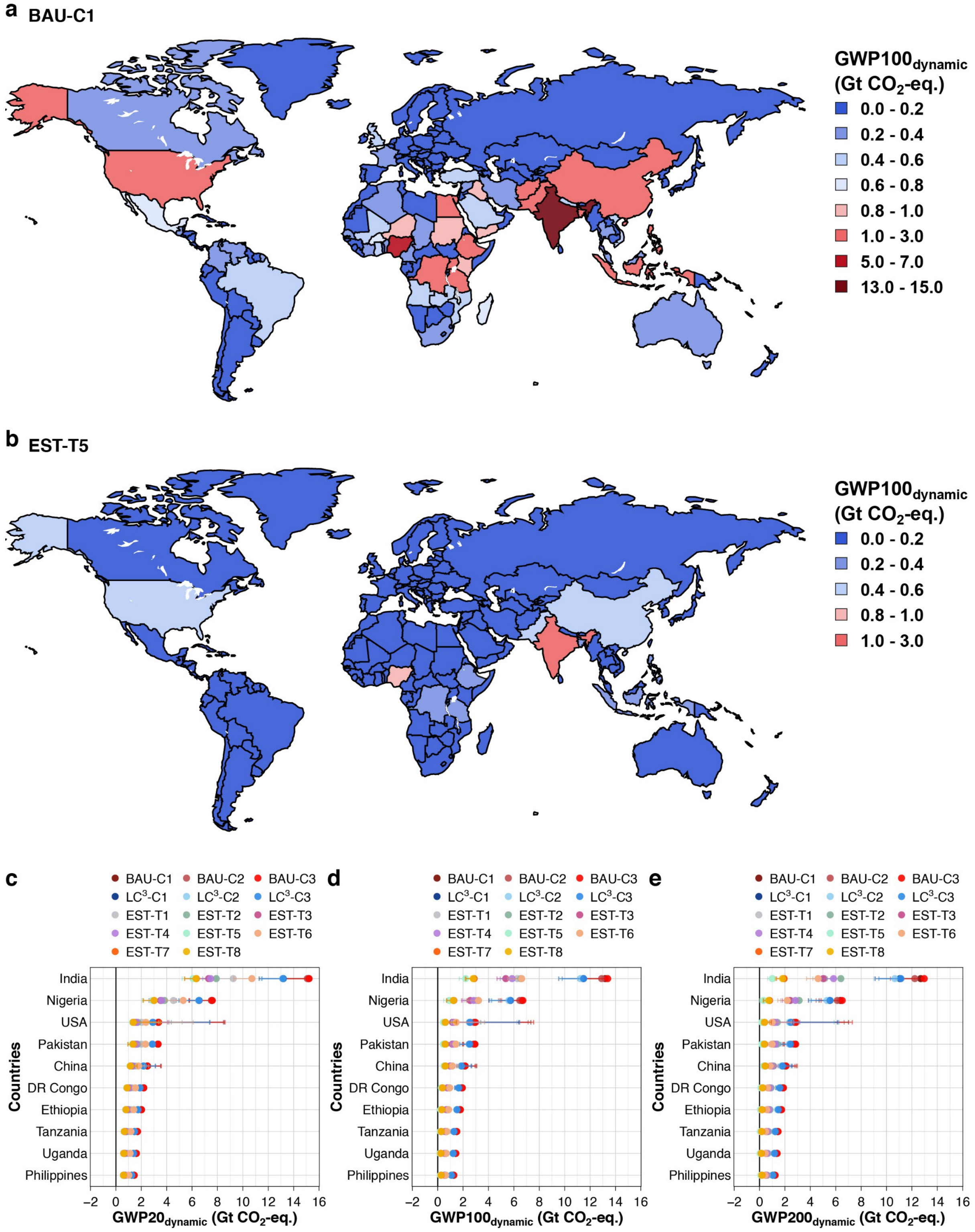

Supplementary Fig. S1 | Geographic breakdown of dynamic global warming potential (GWP) results of future cities based on start-of-life and end-of-life urban building scenarios for time horizon impacts of 20, 100, and 200 years. a GWP100$_{dynamic}$ ranges at a country-scale resolution for the BAU-C3 scenario, b GWP100$_{dynamic}$ ranges at a country-scale resolution for the EST-T6 scenario,

c The ten countries with the highest $GWP20_{dynamic}$ results, d The ten countries with the highest $GWP100_{dynamic}$ results, e The ten countries with the highest $GWP200_{dynamic}$ results. Uncertainties are based on shared socioeconomic pathways (SSPs). For the world maps in a and b, results are shown for SSP2. For graphs in c, d, and e, large dots use SSP2, and error bars quantify uncertainty based on SSP1 and SSP5. Start-of-life scenarios for future urban buildings cover the construction period between 2025 and 2100: BAU, reinforced OPC concrete cities; LC^3, reinforced LC^3 concrete cities; EST, timber cities. End-of-life scenarios for future urban buildings cover the demolition period between 2125 and 2200: C1, concrete is partly recycled, and partly discarded to landfills; C2, concrete is recycled; C3, concrete is discarded to landfills; T1, engineered timber is partly recycled into lower-quality products, partly incinerated, and partly discarded to landfills; T2, engineered timber is incinerated for bioenergy; T3, engineered timber is reused as functionally equivalent products, and this removes the incentive for forest regrowth; T4, engineered timber is recycled into lower quality and/or value products, and this removes the incentive for forest regrowth; T5, engineered timber is partly reused, partly recycled into lower quality/value products, and forestry and land management practices are applied to sustain the forest regrowth; T6, engineered timber is discarded to landfills with direct LFGs release; T7, engineered timber is discarded to landfills, and LFGs are treated by enclosed flare; T8, engineered timber is discarded to landfills, and LFGs are captured and burned for energy production.

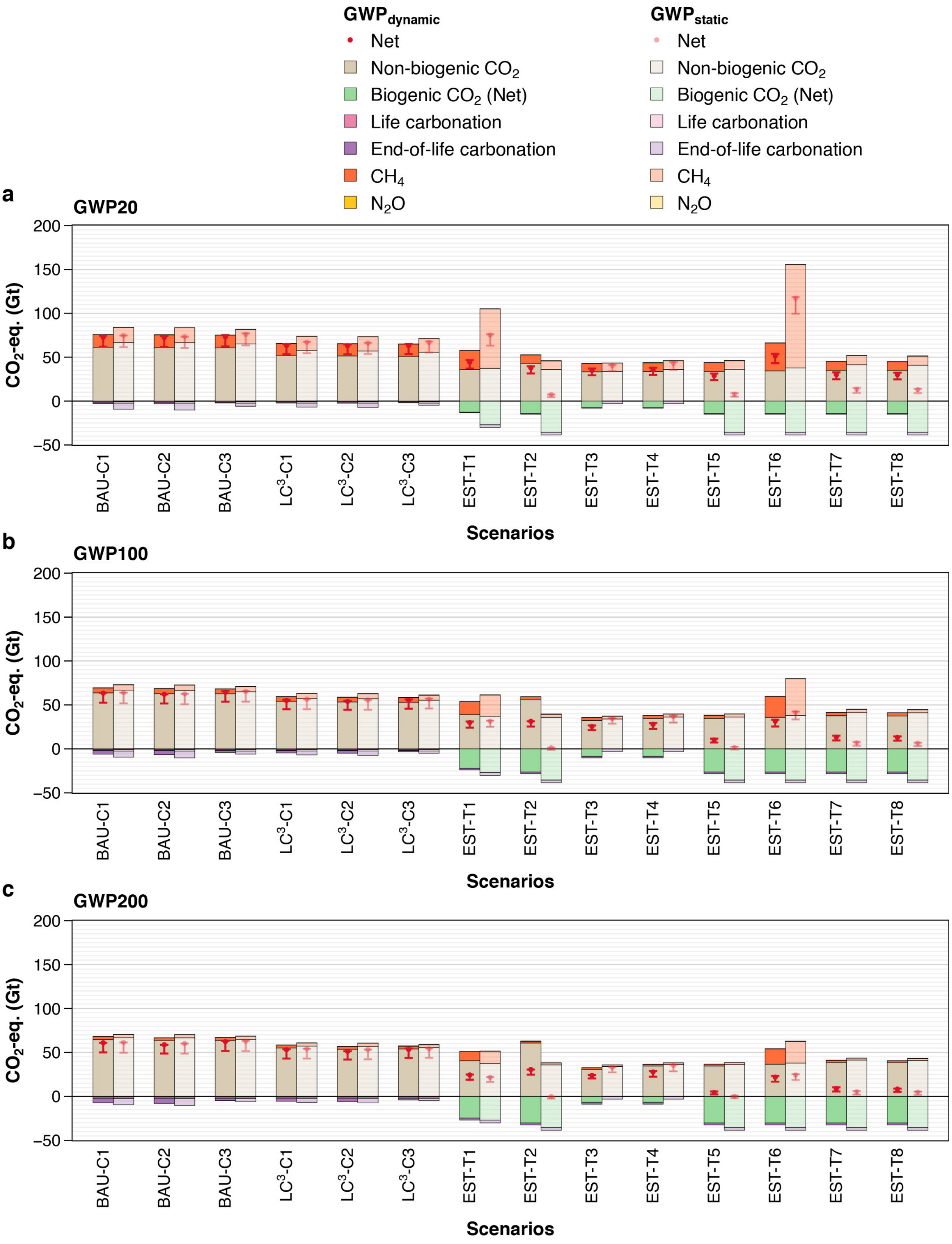

Supplementary Fig. S2 | Dynamic and static global warming potential (GWP) results of future cities based on start-of-life and end-of-life urban building scenarios for time horizon impacts of 20, 100 and 200 years. a GWP20$_{dynamic}$ and GWP20$_{static}$, **b** GWP100$_{dynamic}$ and GWP100$_{static}$, **c** GWP200$_{dynamic}$ and GWP200$_{static}$. Uncertainties are based on shared socioeconomic pathways (SSPs). The contribution analysis (i.e., heights of bars for each variable), red dots and texts for each scenario represent SSP2. Red error bars quantify uncertainty based on SSP1 and SSP5. Start-of-life scenarios for future urban buildings cover the construction period between 2025 and 2100: BAU, reinforced OPC concrete cities; LC^3, reinforced LC^3 concrete cities; EST, timber cities. End-of-life scenarios for future urban buildings cover the demolition period between 2125 and 2200: C1,

concrete is partly recycled, and partly discarded to landfills; C2, concrete is recycled; C3, concrete is discarded to landfills; T1, engineered timber is partly recycled into lower-quality products, partly incinerated, and partly discarded to landfills; T2, engineered timber is incinerated for bioenergy; T3, engineered timber is reused as functionally equivalent products, and this removes the incentive for forest regrowth; T4, engineered timber is recycled into lower quality and/or value products, and this removes the incentive for forest regrowth; T5, engineered timber is partly reused, partly recycled into lower quality/value products, and forestry and land management practices are applied to sustain the forest regrowth; T6, engineered timber is discarded to landfills with direct LFGs release; T7, engineered timber is discarded to landfills, and LFGs are treated by enclosed flare; T8, engineered timber is discarded to landfills, and LFGs are captured and burned for energy production.

S1.1.2. Atmospheric CH_4 concentrations

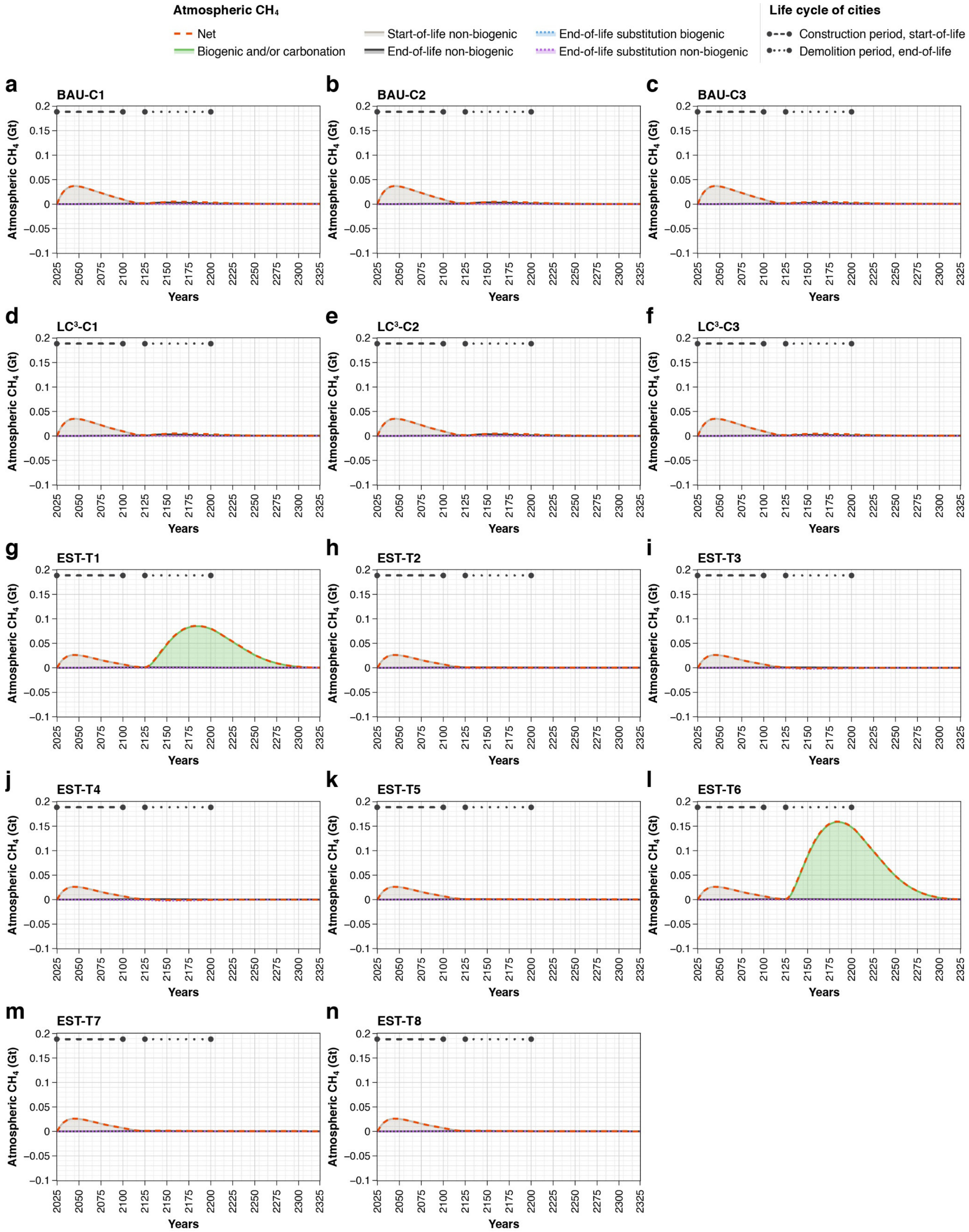

Supplementary Fig. S3 | Atmospheric CH_4 concentrations between 2025 and 2325 based on future timber and reinforced concrete city scenarios. **a** BAU-C1, **b** BAU-C2, **c** BAU-C3, **d** LC^3-C1, **e** LC^3-C2, **f** LC^3-C3, **g** EST-T1, **h** EST-T2, **i** EST-T3, **j** EST-T4, **k** EST-T5, **l** EST-T6, **m** EST-T7, **n** EST-T8. The results are shown for SSP2. Start-of-life scenarios for future urban buildings cover the construction period between 2025 and 2100: BAU, reinforced OPC concrete cities; LC^3, reinforced LC^3 concrete cities; EST, timber cities. End-of-life scenarios for future urban buildings cover the demolition period between 2125 and 2200: C1, concrete is partly recycled, and partly discarded to landfills; C2, concrete is recycled; C3, concrete is discarded to landfills; T1, engineered timber is

partly recycled into lower-quality products, partly incinerated, and partly discarded to landfills; T2, engineered timber is incinerated for bioenergy; T3, engineered timber is reused as functionally equivalent products, and this removes the incentive for forest regrowth; T4, engineered timber is recycled into lower quality and/or value products, and this removes the incentive for forest regrowth; T5, engineered timber is partly reused, partly recycled into lower quality/value products, and forestry and land management practices are applied to sustain the forest regrowth; T6, engineered timber is discarded to landfills with direct LFGs release; T7, engineered timber is discarded to landfills, and LFGs are treated by enclosed flare; T8, engineered timber is discarded to landfills, and LFGs are captured and burned for energy production.

S1.1.3. Sensitivity analysis based on Regionalized Model of Investment and Development (REMIND) integrated assessment model (IAM) scenarios

We have conducted a sensitivity analysis to test the robustness of our results under alternative climate policy assumptions. Specifically, we performed include a total of four Regionalized Model of Investment and Development (REMIND) integrated assessment model (IAM) scenarios: (1) ‘REMIND-SSPs-Base’ represents a current-policies scenario reflecting existing climate policies without full implementation of announced pledges; (2) ‘REMIND-SSPs-NDC’ represents a climate policy scenario consistent with countries’ nationally determined contributions (NDC); (3) ‘REMIND-SSPs-PkBudg1150’ represents a climate policy scenario restricting cumulative CO_2 emissions to a budget of 1150 Gt CO_2, consistent with a ~2 °C target; (4) ‘REMIND-SSPs-PkBudg500’ represents a climate policy scenario restricting cumulative CO_2 emissions to a budget of 500 Gt CO_2, consistent with a ~1.5 °C target. These scenarios span a wide range of mitigation ambition, from relatively pessimistic to highly ambitious pathways.

S1.1.3.1. Start-of-life emission projections by 2100

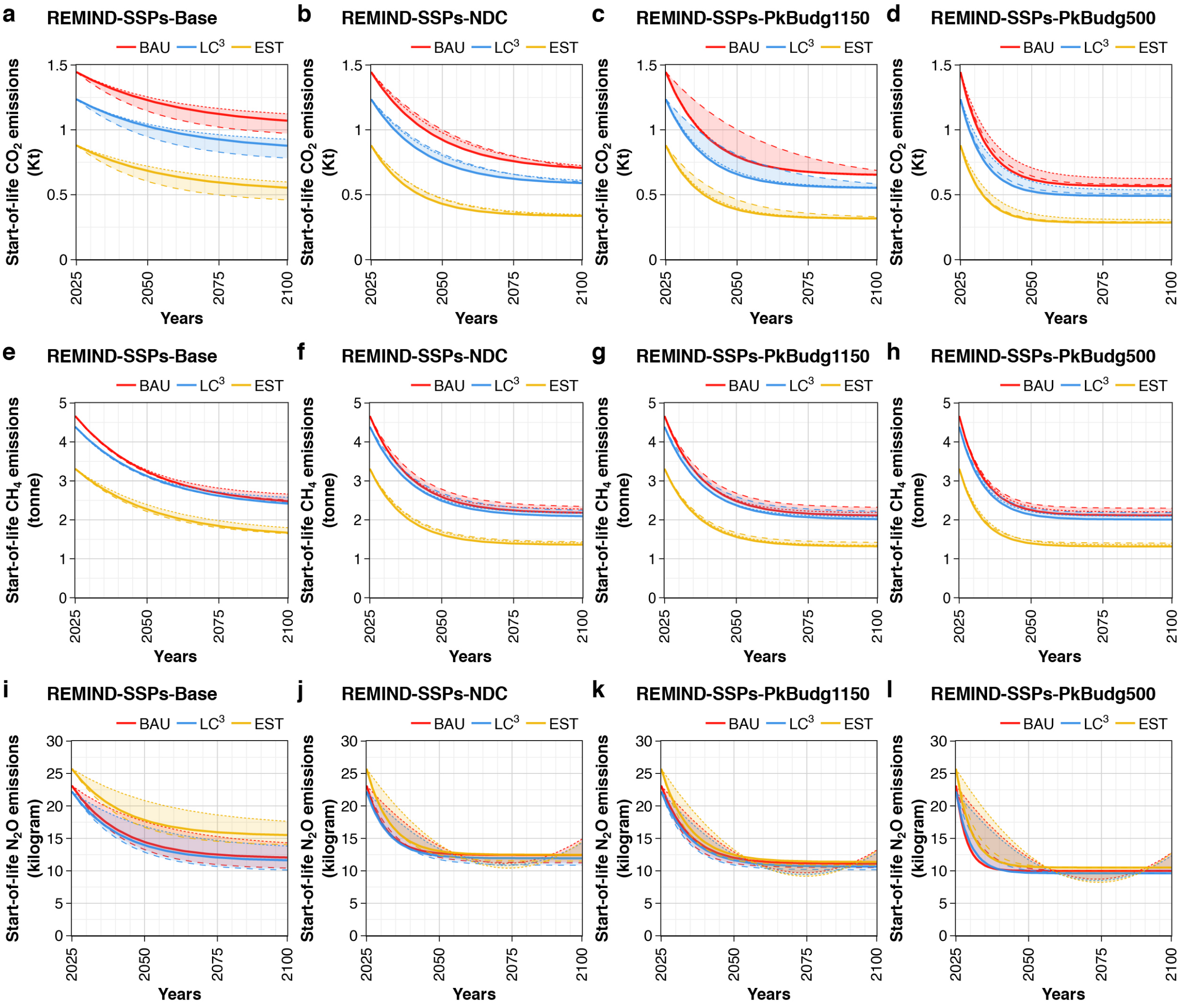

Supplementary Fig. S4 | Start-of-life emission projections of the representative case study building in this study by 2100. Start-of-life CO_2 emissions based on **a** REMIND-SSPs-Base, **b** REMIND-SSPs-NDC, **c** REMIND-SSPs-PkBudg1150, **d** REMIND-SSPs-PkBudg500. Start-of-life CH_4 emissions based on **e** REMIND-SSPs-Base, **f** REMIND-SSPs-NDC, **g** REMIND-SSPs-PkBudg1150, **h** REMIND-SSPs-PkBudg500. Start-of-life N_2O emissions based on **i** REMIND-SSPs-Base, **j** REMIND-SSPs-NDC, **k** REMIND-SSPs-PkBudg1150, **l** REMIND-SSPs-PkBudg500. For each scenario, the solid line (SSP2), the dashed line (SSP1), and the dotted line (SSP5) represent shared socioeconomic pathways (SSPs), with the shaded area between these lines quantifying uncertainty. Start-of-life scenarios for future urban buildings cover the construction period between 2025 and

2100. REMIND-SSPs-Base represents a current-policies scenario reflecting existing climate policies without full implementation of announced pledges; REMIND-SSPs-NDC represents a climate policy scenario consistent with countries' nationally determined contributions (NDC); REMIND-SSPs-PkBudg1150 represents a climate policy scenario restricting cumulative CO_2 emissions to a budget of 1150 Gt CO_2, consistent with a ~2 °C target; REMIND-SSPs-PkBudg500 represents a climate policy scenario restricting cumulative CO_2 emissions to a budget of 500 Gt CO_2, consistent with a ~1.5 °C target. BAU, reinforced OPC concrete building; LC^3, reinforced LC^3 concrete building; EST, engineered structural timber building.

S1.1.3.2. Absolute global temperature potential

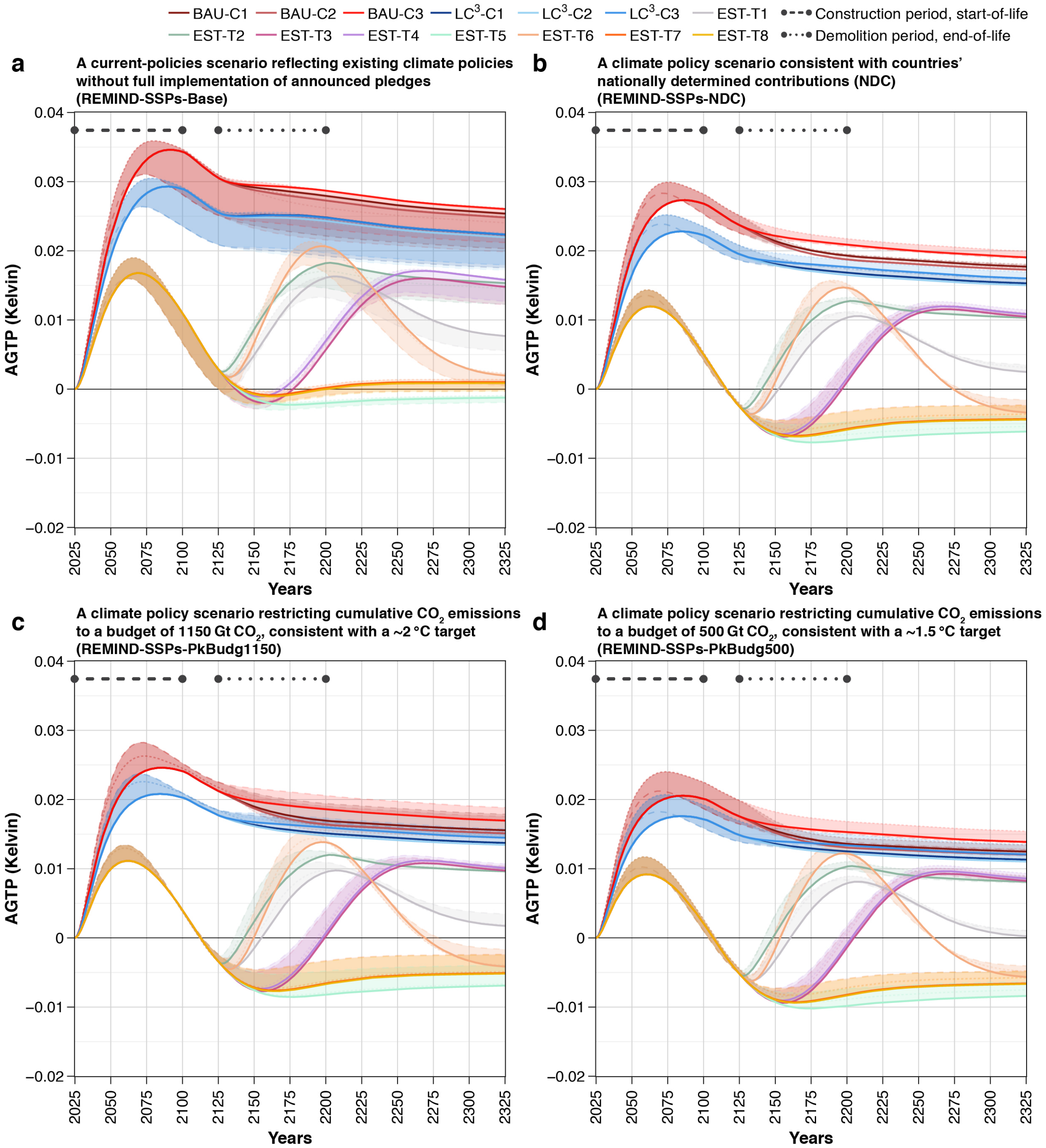

Supplementary Fig. S5 | Absolute global temperature potential (AGTP) of future cities between 2025 and 2325 based on start-of-life and end-of-life urban building scenarios, and various REMIND IAM scenarios. a A current-policies scenario reflecting existing climate policies without full implementation of announced pledges (REMIND-SSPs-Base), **b** A climate policy scenario consistent with countries' nationally determined contributions (NDC) (REMIND-SSPs-NDC), **c** A climate policy scenario restricting cumulative CO_2 emissions to a budget of 1150 Gt CO_2, consistent with a ~2 °C target (REMIND-SSPs-PkBudg1150), **d** A climate policy scenario restricting cumulative CO_2 emissions to a budget of 500 Gt CO_2, consistent with a ~1.5 °C target (REMIND-SSPs-PkBudg500). For each scenario, the solid line (SSP2), the dashed line (SSP1), and the dotted line (SSP5) represent shared socioeconomic pathways (SSPs), with the shaded area between these lines quantifying uncertainty. BAU, reinforced OPC concrete building; LC^3, reinforced LC^3 concrete building; EST, engineered structural timber building; C1, concrete is partly recycled, and partly discarded to landfills; C2, concrete is recycled; C3, concrete is discarded to landfills; T1, engineered timber is partly recycled into lower-quality products, partly incinerated, and partly discarded to landfills; T2, engineered timber is incinerated for bioenergy; T3, engineered timber is reused as

functionally equivalent products, and this removes the incentive for forest regrowth; T4, engineered timber is recycled into lower quality and/or value products, and this removes the incentive for forest regrowth; T5, engineered timber is partly reused, partly recycled into lower quality/value products, and forestry and land management practices are applied to sustain the forest regrowth; T6, engineered timber is discarded to landfills with direct LFGs release; T7, engineered timber is discarded to landfills, and LFGs are treated by enclosed flare; T8, engineered timber is discarded to landfills, and LFGs are captured and burned for energy production.

S1.1.3.3. Global potential warming potential

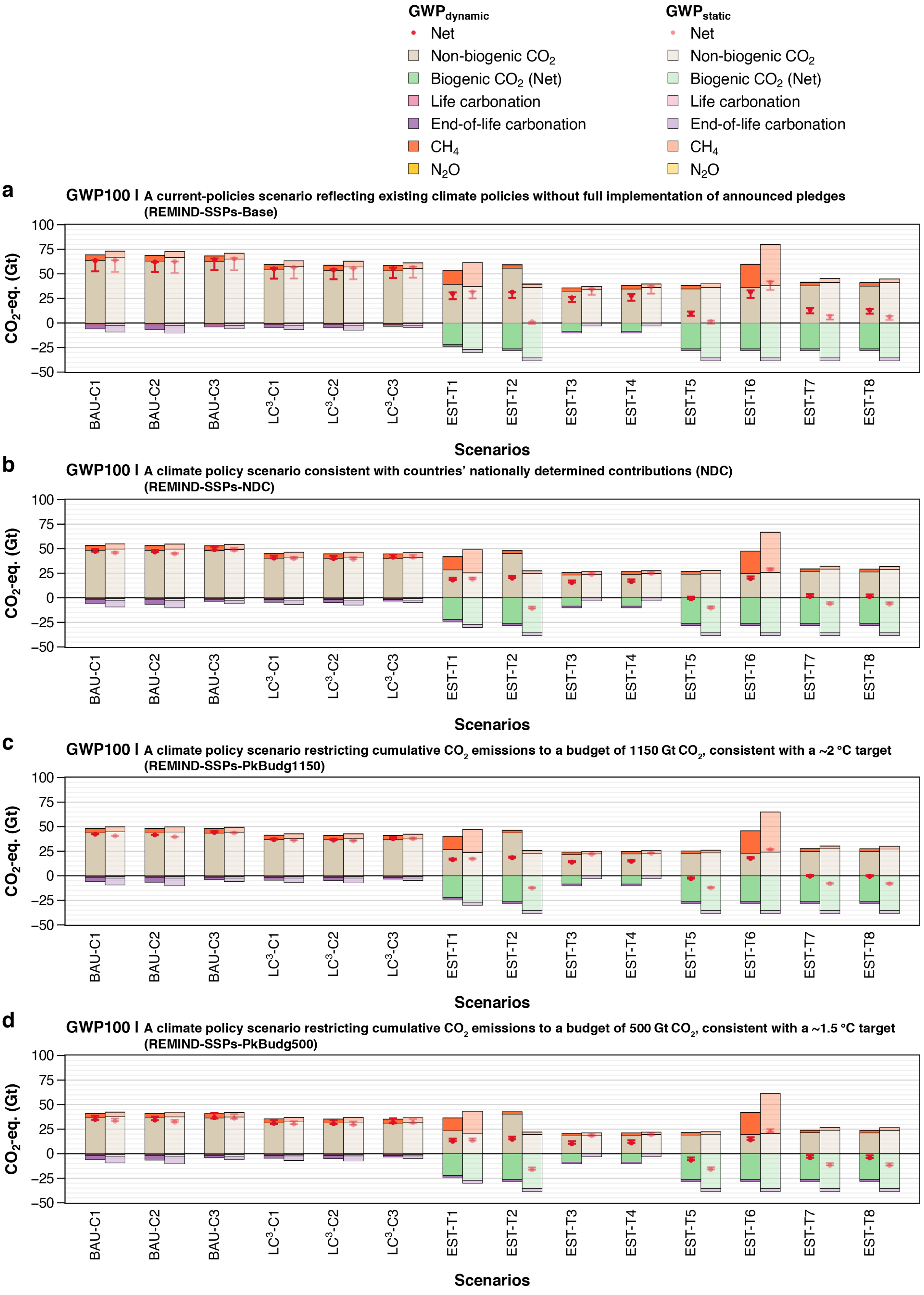

Supplementary Fig. S6 | Dynamic and static global warming potential (GWP) results of future cities based on start-of-life and end-of-life urban building scenarios, and various REMIND IAM scenarios for the time horizon impact of 100 years ($GWP100_{dynamic}$ and $GWP100_{static}$). a A

current-policies scenario reflecting existing climate policies without full implementation of announced pledges (REMIND-SSPs-Base), **b** A climate policy scenario consistent with countries' nationally determined contributions (NDC) (REMIND-SSPs-NDC), **c** A climate policy scenario restricting cumulative CO_2 emissions to a budget of 1150 Gt CO_2, consistent with a ~2 °C target (REMIND-SSPs-PkBudg1150), **d** A climate policy scenario restricting cumulative CO_2 emissions to a budget of 500 Gt CO_2, consistent with a ~1.5 °C target (REMIND-SSPs-PkBudg500). Uncertainties are based on shared socioeconomic pathways (SSPs). The contribution analysis (i.e., heights of bars for each variable), red dots and texts for each scenario represent SSP2. Red error bars quantify uncertainty based on SSP1 and SSP5. Start-of-life scenarios for future urban buildings cover the construction period between 2025 and 2100: BAU, reinforced OPC concrete cities; LC^3, reinforced LC^3 concrete cities; EST, timber cities. End-of-life scenarios for future urban buildings cover the demolition period between 2125 and 2200: C1, concrete is partly recycled, and partly discarded to landfills; C2, concrete is recycled; C3, concrete is discarded to landfills; T1, engineered timber is partly recycled into lower-quality products, partly incinerated, and partly discarded to landfills; T2, engineered timber is incinerated for bioenergy; T3, engineered timber is reused as functionally equivalent products, and this removes the incentive for forest regrowth; T4, engineered timber is recycled into lower quality and/or value products, and this removes the incentive for forest regrowth; T5, engineered timber is partly reused, partly recycled into lower quality/value products, and forestry and land management practices are applied to sustain the forest regrowth; T6, engineered timber is discarded to landfills with direct LFGs release; T7, engineered timber is discarded to landfills, and LFGs are treated by enclosed flare; T8, engineered timber is discarded to landfills, and LFGs are captured and burned for energy production.

S1.1.4. Sensitivity analysis based on building lifespan

S1.1.4.1. Absolute global temperature potential

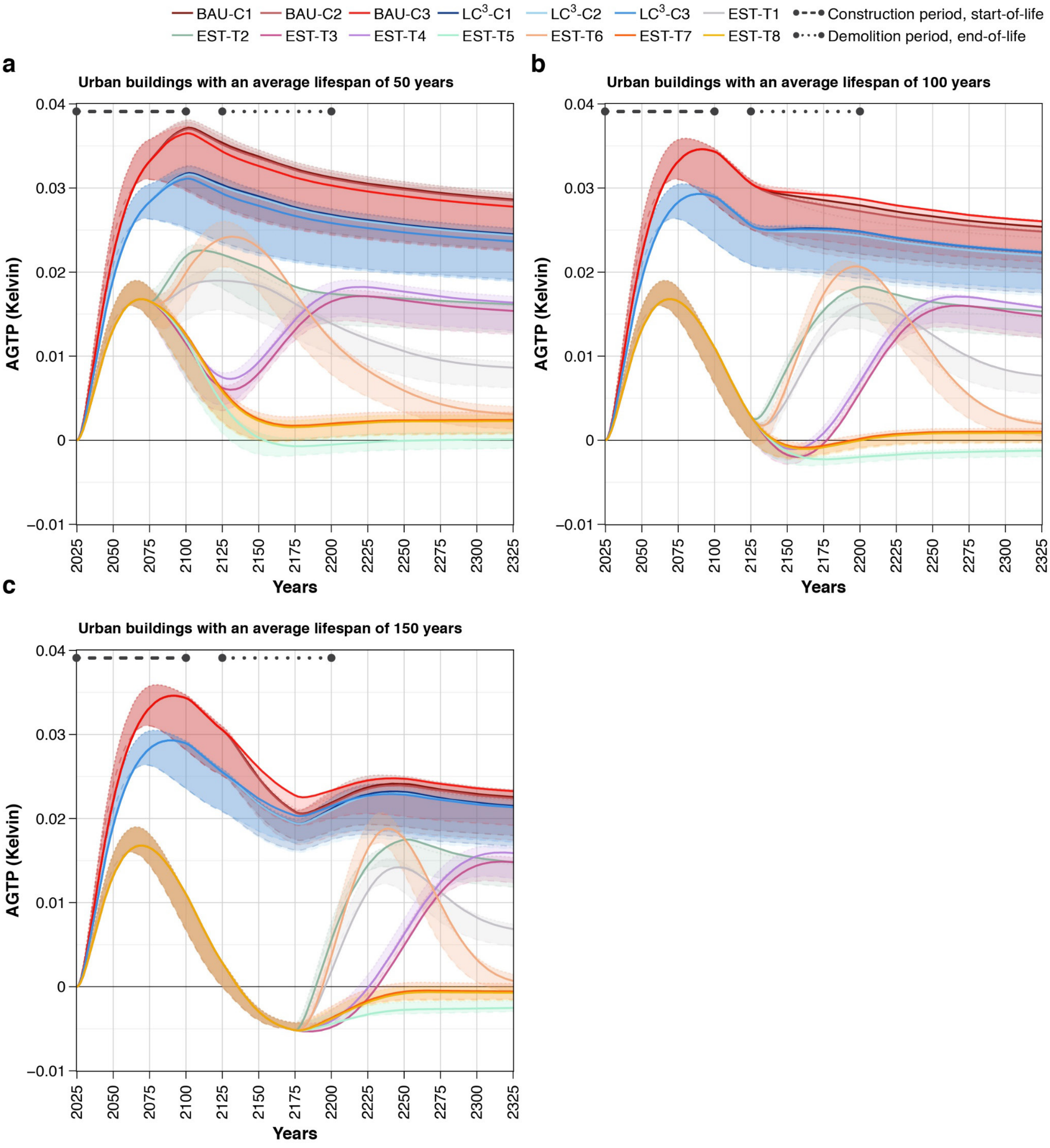

Supplementary Fig. S7 | Absolute global temperature potential (AGTP) of future cities between 2025 and 2325 based on start-of-life and end-of-life urban building scenarios, and various building lifespans. a Urban buildings with an average lifespan of 50 years, **b** Urban buildings with an average lifespan of 100 years, **c** Urban buildings with an average lifespan of 150 years For each scenario, the solid line (SSP2), the dashed line (SSP1), and the dotted line (SSP5) represent shared socioeconomic pathways (SSPs), with the shaded area between these lines quantifying uncertainty. BAU, reinforced OPC concrete building; LC^3, reinforced LC^3 concrete building; EST, engineered structural timber building; C1, concrete is partly recycled, and partly discarded to landfills; C2, concrete is recycled; C3, concrete is discarded to landfills; T1, engineered timber is partly recycled into lower-quality products, partly incinerated, and partly discarded to landfills; T2, engineered timber is incinerated for bioenergy; T3, engineered timber is reused as functionally equivalent products, and this removes the incentive for forest regrowth; T4, engineered timber is recycled into lower quality and/or value products, and this removes the incentive for forest regrowth; T5, engineered timber is

partly reused, partly recycled into lower quality/value products, and forestry and land management practices are applied to sustain the forest regrowth; T6, engineered timber is discarded to landfills with direct LFGs release; T7, engineered timber is discarded to landfills, and LFGs are treated by enclosed flare; T8, engineered timber is discarded to landfills, and LFGs are captured and burned for energy production.

S1.1.4.2. Global warming potential

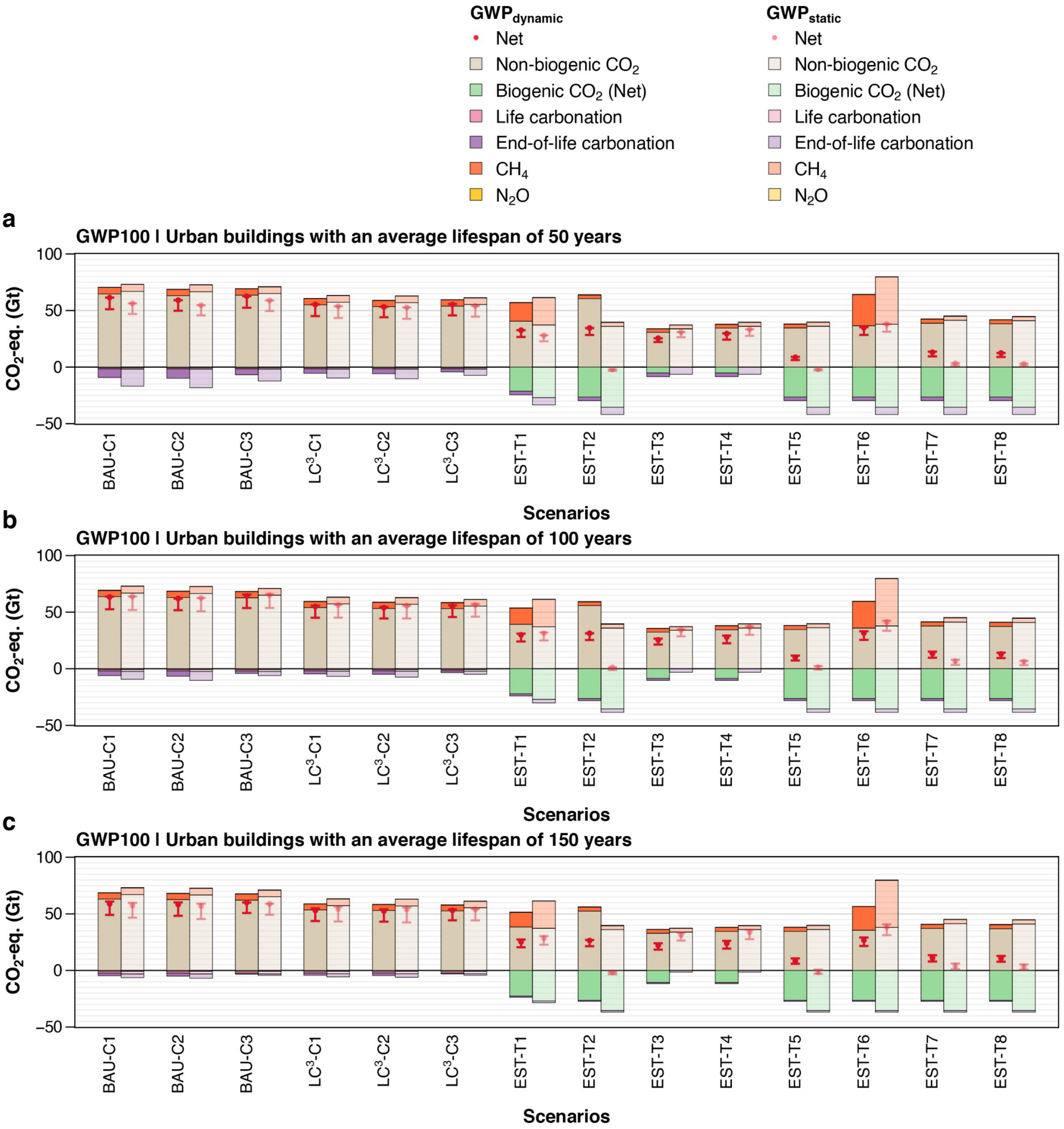

Supplementary Fig. S8 | Dynamic and static global warming potential (GWP) results of future cities based on start-of-life and end-of-life urban building scenarios, and various building lifespans for the time horizon impact of 100 years ($GWP100_{dynamic}$ and $GWP100_{static}$). a Urban buildings with an average lifespan of 50 years, **b** Urban buildings with an average lifespan of 100 years, **c** Urban buildings with an average lifespan of 150 years. Uncertainties are based on shared socioeconomic pathways (SSPs). The contribution analysis (i.e., heights of bars for each variable), red dots and texts for each scenario represent SSP2. Red error bars quantify uncertainty based on SSP1 and SSP5. Start-of-life scenarios for future urban buildings cover the construction period between 2025 and 2100: BAU, reinforced OPC concrete cities; LC^3, reinforced LC^3 concrete cities; EST, timber cities. End-of-life scenarios for future urban buildings cover the demolition period between 2125 and 2200: C1, concrete is partly recycled, and partly discarded to landfills; C2, concrete is recycled; C3, concrete is discarded to landfills; T1, engineered timber is partly recycled into lower-quality products, partly incinerated, and partly discarded to landfills; T2, engineered timber is incinerated for bioenergy; T3, engineered timber is reused as functionally equivalent products, and this removes the incentive for forest regrowth; T4, engineered timber is recycled into lower quality and/or value products, and this removes the incentive for forest regrowth; T5, engineered timber is

partly reused, partly recycled into lower quality/value products, and forestry and land management practices are applied to sustain the forest regrowth; T6, engineered timber is discarded to landfills with direct LFGs release; T7, engineered timber is discarded to landfills, and LFGs are treated by enclosed flare; T8, engineered timber is discarded to landfills, and LFGs are captured and burned for energy production.

S1.1.5. Sensitivity analysis based on floor area per capita

Our results show that floor area per capita is a critical parameter shaping long-term climate change impacts. Higher floor area levels substantially increase construction-related emissions (Supplementary Figs. S9,10). It may also drive other key emission factors, such as land-use and energy demand. At the same time, low floor area provision may negatively affect human well-being, since access to a minimum living space is a basic requirement regardless of regional context. Urban planning and housing guidelines therefore need to carefully balance these competing objectives, accounting for both climate change mitigation and minimum standards of living.

S1.1.5.1. Absolute global temperature potential

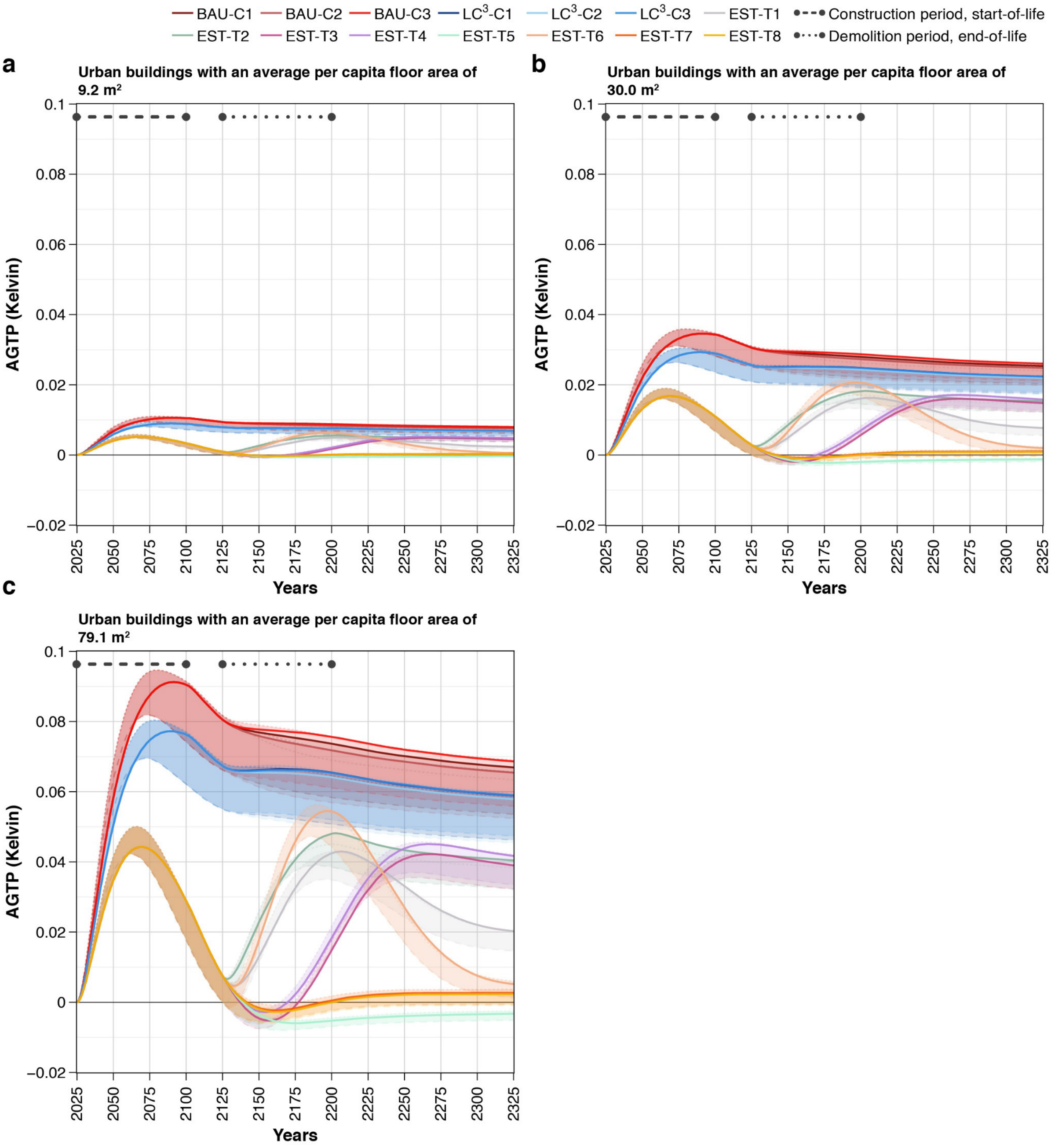

Supplementary Fig. S9 | Absolute global temperature potential (AGTP) of future cities between 2025 and 2325 based on start-of-life and end-of-life urban building scenarios, and various floor area per capita. a Urban buildings with an average per capita floor area of 9.2 m^2, **b** Urban buildings with an average per capita floor area of 30.0 m^2, **c** Urban buildings with an average per capita floor area of 79.1 m^2. For each scenario, the solid line (SSP2), the dashed line (SSP1), and the dotted line (SSP5) represent shared socioeconomic pathways (SSPs), with the shaded area between these lines quantifying uncertainty. BAU, reinforced OPC concrete building; LC^3, reinforced LC^3 concrete building; EST, engineered structural timber building; C1, concrete is partly recycled, and partly discarded to landfills; C2, concrete is recycled; C3, concrete is discarded to landfills; T1, engineered timber is partly recycled into lower-quality products, partly incinerated, and partly discarded to landfills; T2, engineered timber is incinerated for bioenergy; T3, engineered timber is reused as functionally equivalent products, and this removes the incentive for forest regrowth; T4, engineered timber is recycled into lower quality and/or value products, and this removes the incentive for forest regrowth; T5, engineered timber is partly reused, partly recycled into lower quality/value products, and forestry and land management practices are applied to sustain the forest regrowth; T6, engineered

timber is discarded to landfills with direct LFGs release; T7, engineered timber is discarded to landfills, and LFGs are treated by enclosed flare; T8, engineered timber is discarded to landfills, and LFGs are captured and burned for energy production.

S1.1.5.2. Global warming potential

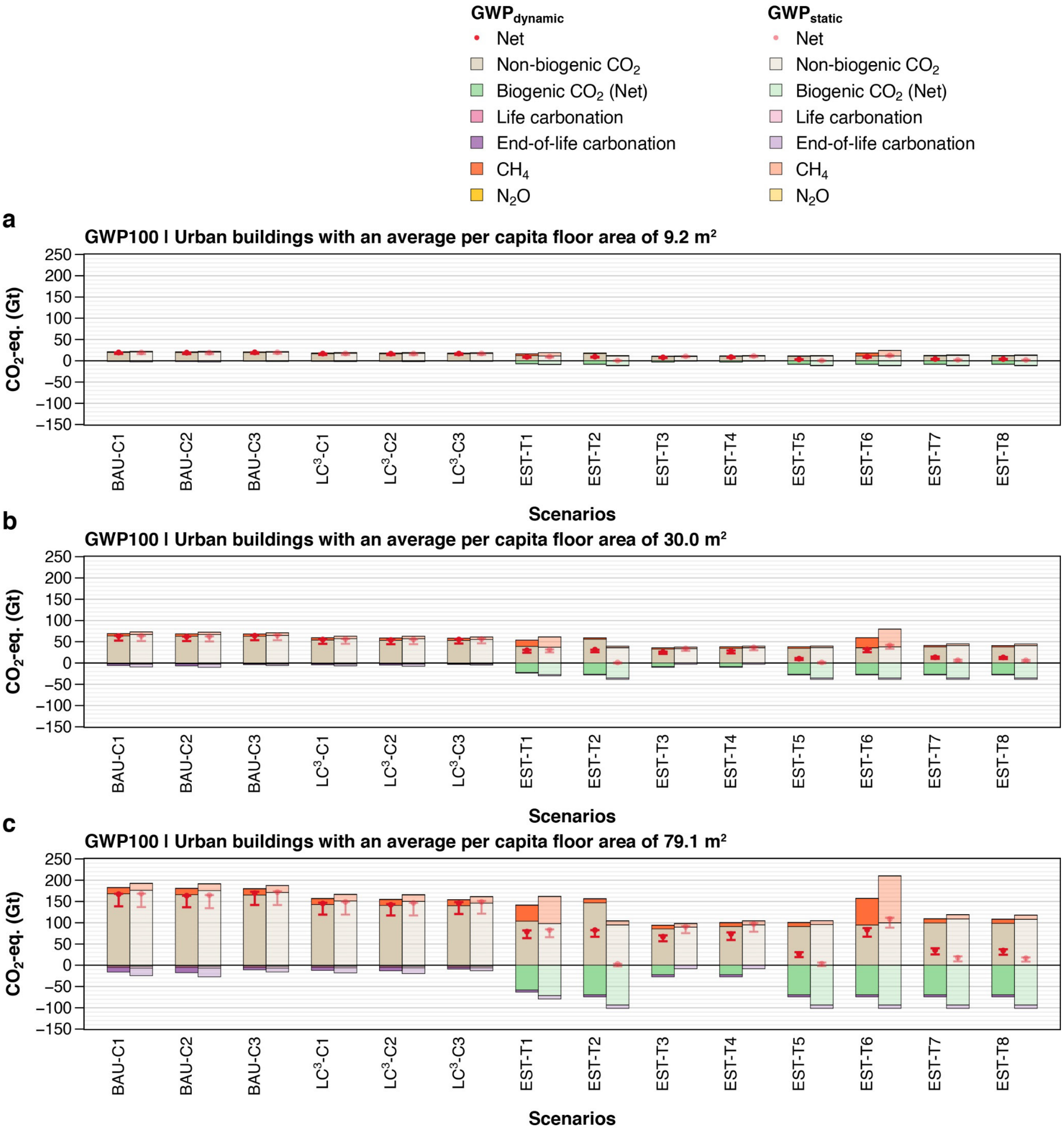

Supplementary Fig. S10 | Dynamic and static global warming potential (GWP) results of future cities based on start-of-life and end-of-life urban building scenarios, and various floor area per capita for the time horizon impact of 100 years ($GWP100_{dynamic}$ and $GWP100_{static}$). a Urban buildings with an average per capita floor area of 9.2 m^2, **b** Urban buildings with an average per capita floor area of 30.0 m^2, **c** Urban buildings with an average per capita floor area of 79.1 m^2. Uncertainties are based on shared socioeconomic pathways (SSPs). The contribution analysis (i.e., heights of bars for each variable), red dots and texts for each scenario represent SSP2. Red error bars quantify uncertainty based on SSP1 and SSP5. Start-of-life scenarios for future urban buildings cover the construction period between 2025 and 2100: BAU, reinforced OPC concrete cities; LC^3, reinforced LC^3 concrete cities; EST, timber cities. End-of-life scenarios for future urban buildings cover the demolition period between 2125 and 2200: C1, concrete is partly recycled, and partly discarded to landfills; C2, concrete is recycled; C3, concrete is discarded to landfills; T1, engineered timber is partly recycled into lower-quality products, partly incinerated, and partly discarded to landfills; T2, engineered timber is incinerated for bioenergy; T3, engineered timber is reused as functionally equivalent products, and this removes the incentive for forest regrowth; T4, engineered timber is recycled into lower quality and/or value products, and this removes the incentive for forest regrowth;

T5, engineered timber is partly reused, partly recycled into lower quality/value products, and forestry and land management practices are applied to sustain the forest regrowth; T6, engineered timber is discarded to landfills with direct LFGs release; T7, engineered timber is discarded to landfills, and LFGs are treated by enclosed flare; T8, engineered timber is discarded to landfills, and LFGs are captured and burned for energy production.

S1.1.6. Sensitivity analysis based on forest rotation period

S1.1.6.1. Absolute global temperature potential

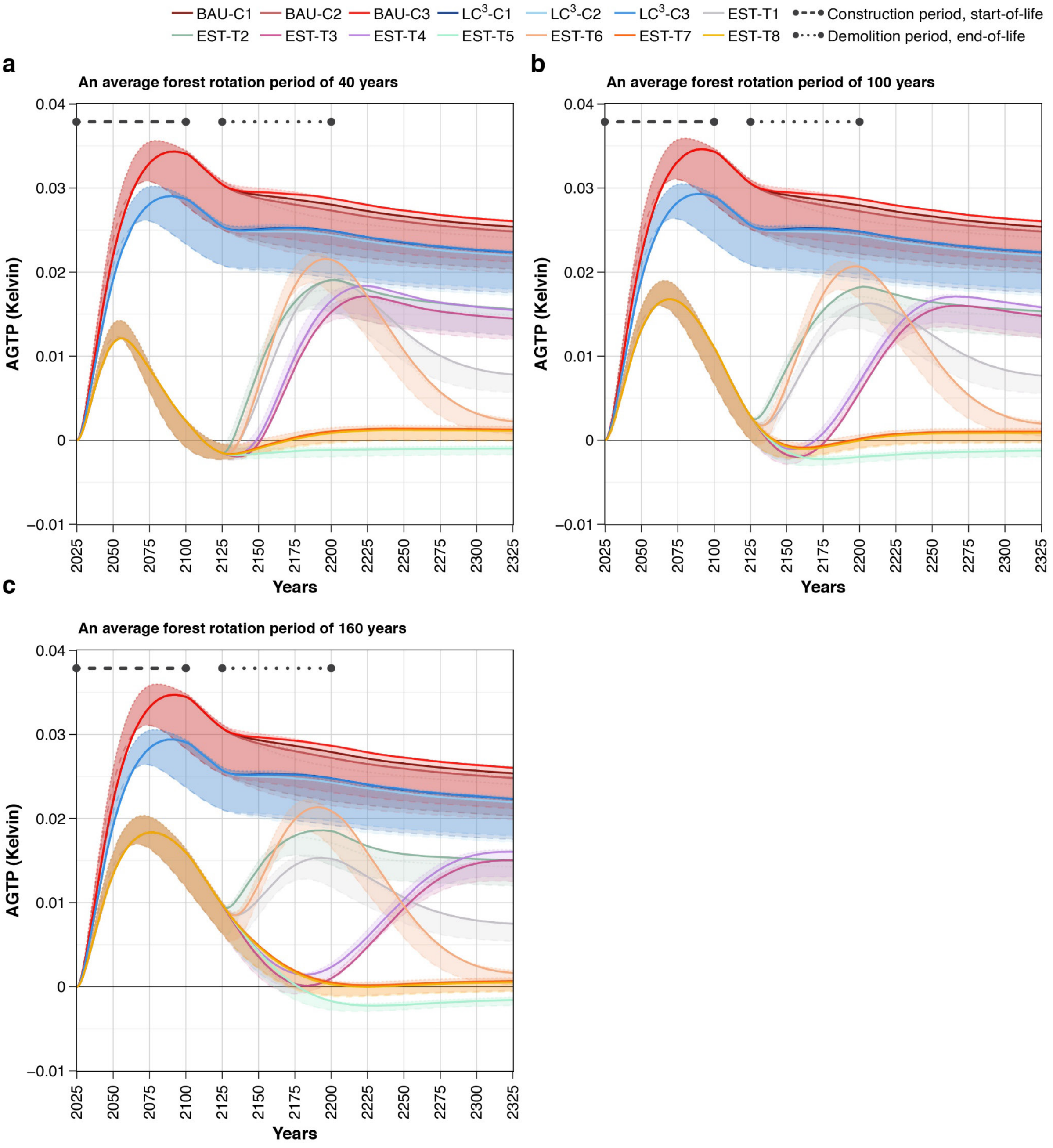

Supplementary Fig. S11 | Absolute global temperature potential (AGTP) of future cities between 2025 and 2325 based on start-of-life and end-of-life urban building scenarios, and various forest rotation periods. a An average forest rotation period of 40 years, **b** An average forest rotation period of 100 years, **c** An average forest rotation period of 160 years. For each scenario, the solid line (SSP2), the dashed line (SSP1), and the dotted line (SSP5) represent shared socioeconomic pathways (SSPs), with the shaded area between these lines quantifying uncertainty. BAU, reinforced OPC concrete building; LC^3, reinforced LC^3 concrete building; EST, engineered structural timber building; C1, concrete is partly recycled, and partly discarded to landfills; C2, concrete is recycled; C3, concrete is discarded to landfills; T1, engineered timber is partly recycled into lower-quality products, partly incinerated, and partly discarded to landfills; T2, engineered timber is incinerated for bioenergy; T3, engineered timber is reused as functionally equivalent products, and this removes the incentive for forest regrowth; T4, engineered timber is recycled into lower quality and/or value products, and this removes the incentive for forest regrowth; T5, engineered timber is

partly reused, partly recycled into lower quality/value products, and forestry and land management practices are applied to sustain the forest regrowth; T6, engineered timber is discarded to landfills with direct LFGs release; T7, engineered timber is discarded to landfills, and LFGs are treated by enclosed flare; T8, engineered timber is discarded to landfills, and LFGs are captured and burned for energy production.

S1.1.6.2. Global warming potential

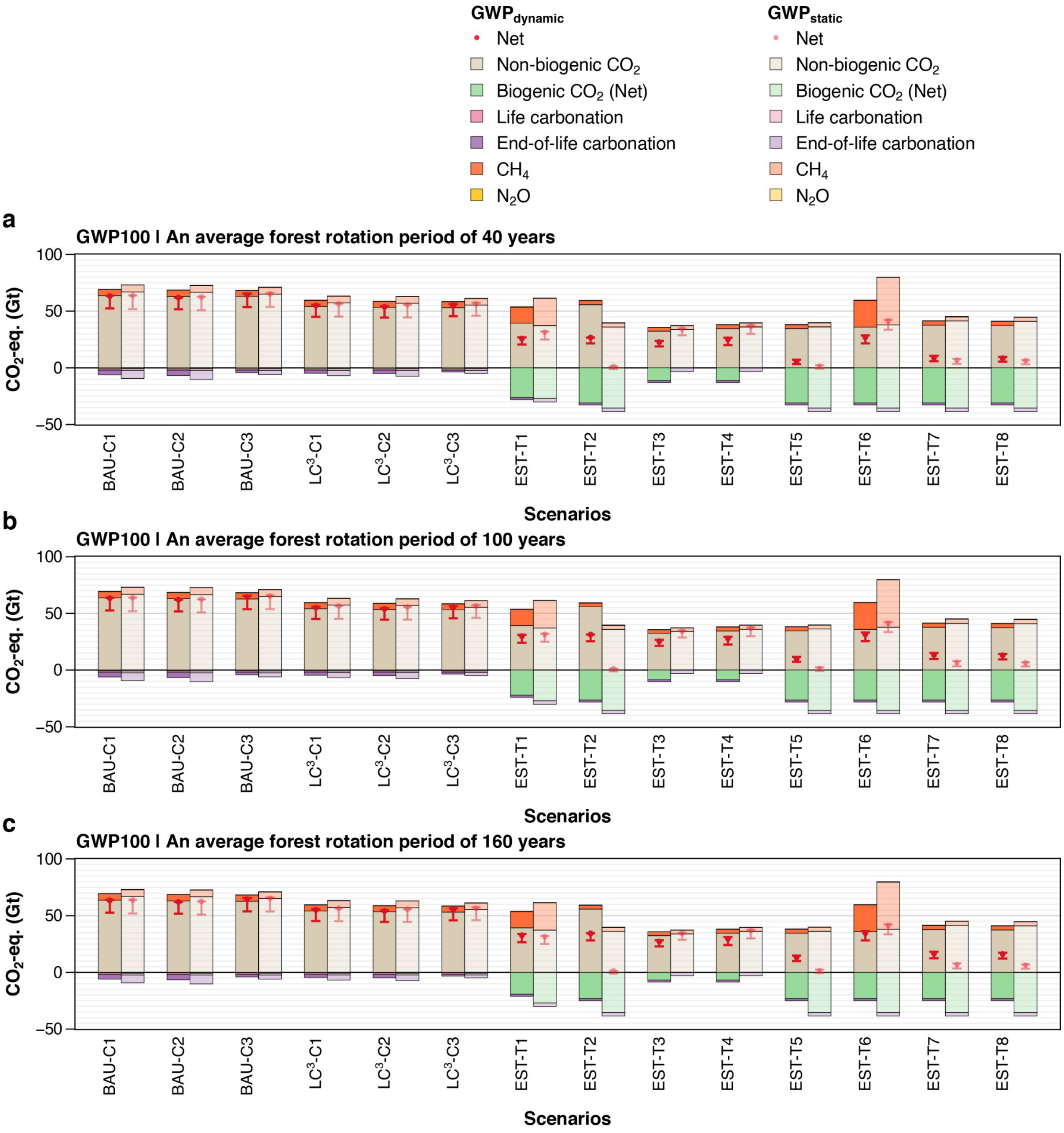

Supplementary Fig. S12 | Dynamic and static global warming potential (GWP) results of future cities based on start-of-life and end-of-life urban building scenarios, and various forest rotation periods for the time horizon impact of 100 years ($GWP100_{dynamic}$ and $GWP100_{static}$). a An average forest rotation period of 40 years, **b** An average forest rotation period of 100 years, **c** An average forest rotation period of 160 years. Uncertainties are based on shared socioeconomic pathways (SSPs). The contribution analysis (i.e., heights of bars for each variable), red dots and texts for each scenario represent SSP2. Red error bars quantify uncertainty based on SSP1 and SSP5. Start-of-life scenarios for future urban buildings cover the construction period between 2025 and 2100: BAU, reinforced OPC concrete cities; LC^3, reinforced LC^3 concrete cities; EST, timber cities. End-of-life scenarios for future urban buildings cover the demolition period between 2125 and 2200: C1, concrete is partly recycled, and partly discarded to landfills; C2, concrete is recycled; C3, concrete is discarded to landfills; T1, engineered timber is partly recycled into lower-quality products, partly incinerated, and partly discarded to landfills; T2, engineered timber is incinerated for bioenergy; T3, engineered timber is reused as functionally equivalent products, and this removes the incentive for forest regrowth; T4, engineered timber is recycled into lower quality and/or value products, and this removes the incentive for forest regrowth; T5, engineered timber is partly reused, partly recycled

into lower quality/value products, and forestry and land management practices are applied to sustain the forest regrowth; T6, engineered timber is discarded to landfills with direct LFGs release; T7, engineered timber is discarded to landfills, and LFGs are treated by enclosed flare; T8, engineered timber is discarded to landfills, and LFGs are captured and burned for energy production.

S1.1.7. Sensitivity analysis based on wood decay rate in landfills

S1.1.7.1. Absolute global temperature potential

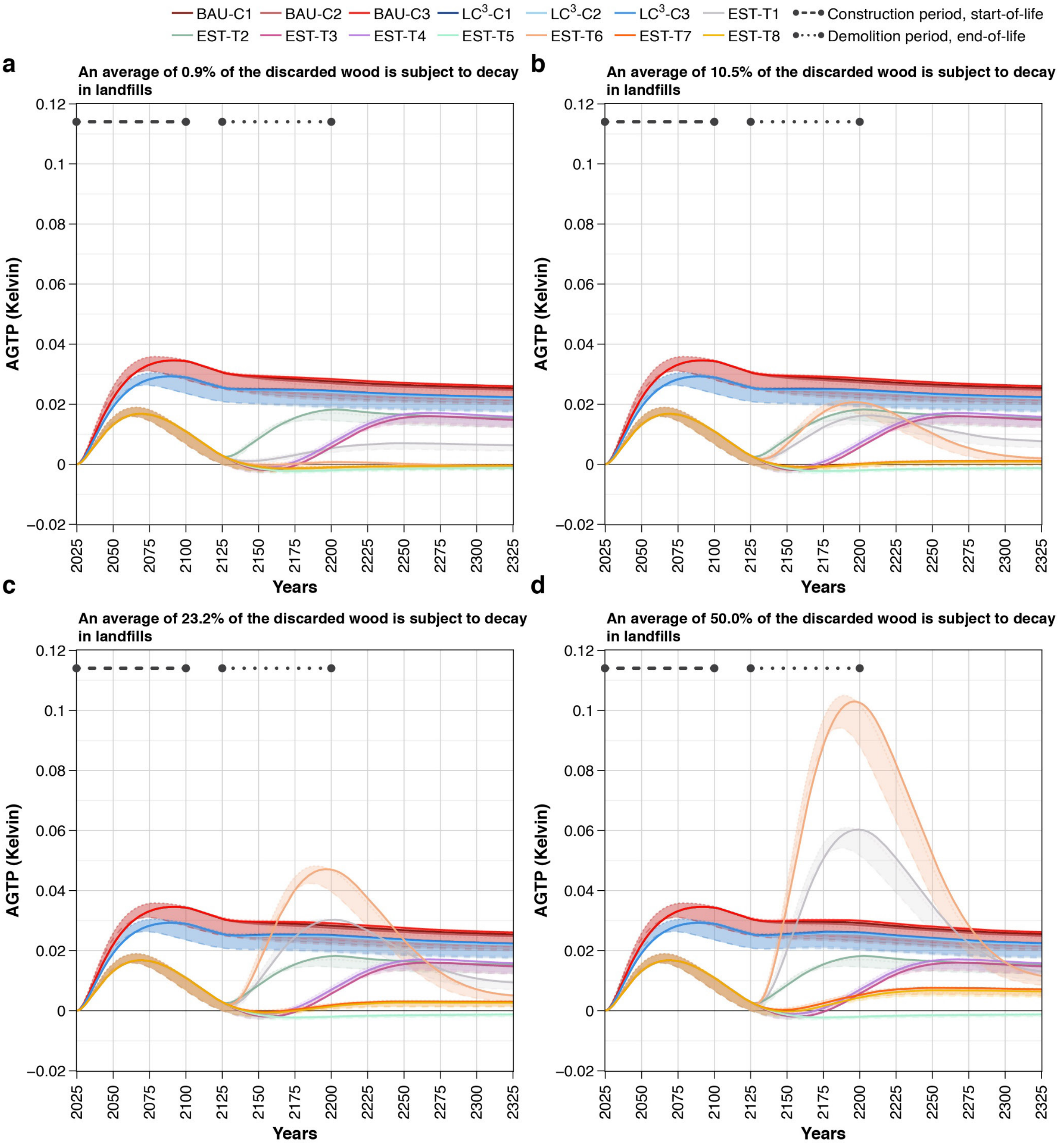

Supplementary Fig. S13 | Absolute global temperature potential (AGTP) of future cities between 2025 and 2325 based on start-of-life and end-of-life urban building scenarios, and various wood decay rates in landfills. a An average of 0.9% of the discarded wood is subject to decay in landfills, **b** An average of 10.5% of the discarded wood is subject to decay in landfills, **c** An average of 23.2% of the discarded wood is subject to decay in landfills, **d** An average of 50% of the discarded wood is subject to decay in landfills. For each scenario, the solid line (SSP2), the dashed line (SSP1), and the dotted line (SSP5) represent shared socioeconomic pathways (SSPs), with the shaded area between these lines quantifying uncertainty. BAU, reinforced OPC concrete building; LC^3, reinforced LC^3 concrete building; EST, engineered structural timber building; C1, concrete is partly recycled, and partly discarded to landfills; C2, concrete is recycled; C3, concrete is discarded to landfills; T1, engineered timber is partly recycled into lower-quality products, partly incinerated, and partly discarded to landfills; T2, engineered timber is incinerated for bioenergy; T3, engineered timber is reused as functionally equivalent products, and this removes the incentive for forest regrowth; T4,

engineered timber is recycled into lower quality and/or value products, and this removes the incentive for forest regrowth; T5, engineered timber is partly reused, partly recycled into lower quality/value products, and forestry and land management practices are applied to sustain the forest regrowth; T6, engineered timber is discarded to landfills with direct LFGs release; T7, engineered timber is discarded to landfills, and LFGs are treated by enclosed flare; T8, engineered timber is discarded to landfills, and LFGs are captured and burned for energy production.

S1.1.7.2. Global warming potential

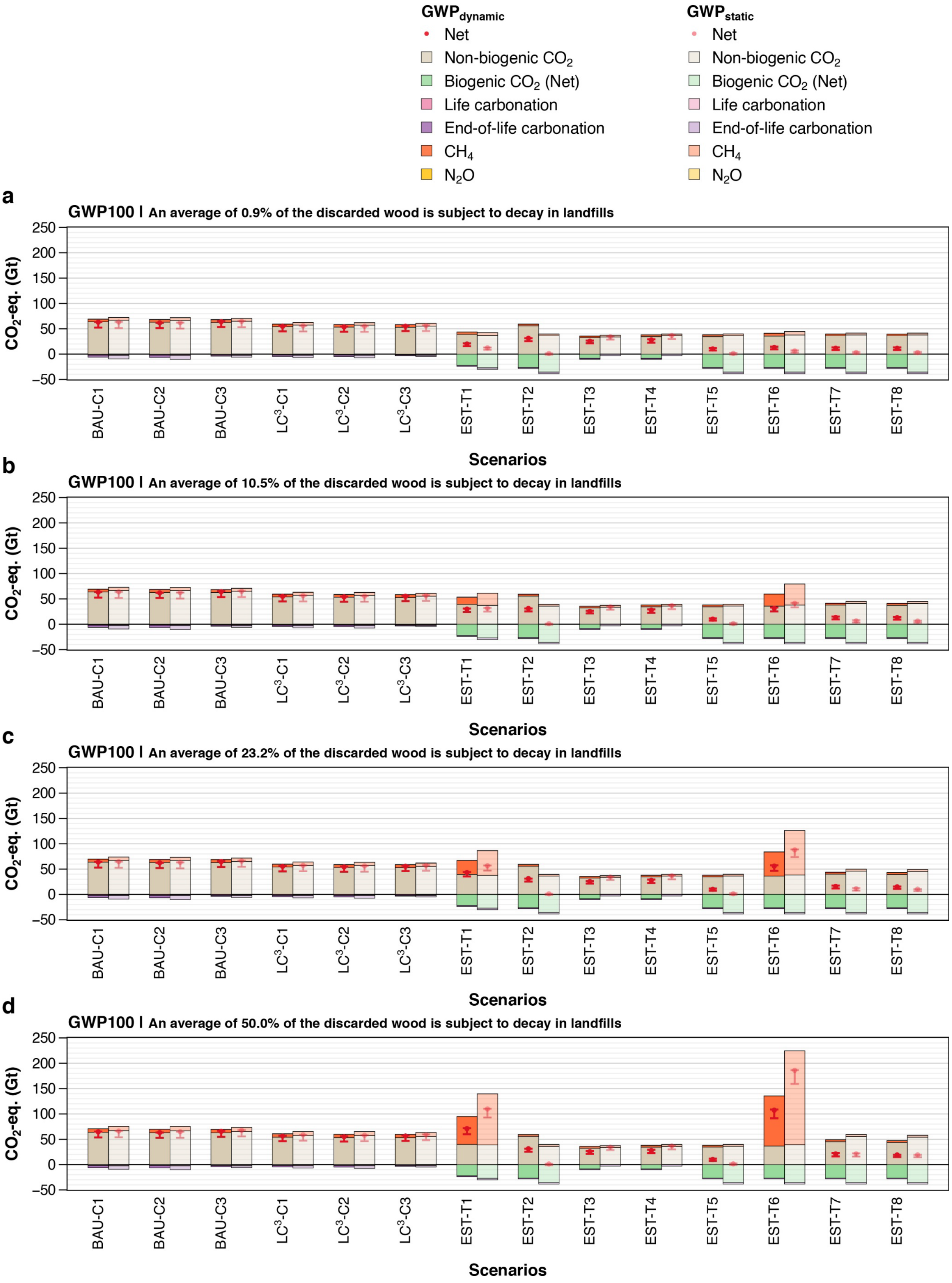

Supplementary Fig. S14 | Dynamic and static global warming potential (GWP) results of future cities based on start-of-life and end-of-life urban building scenarios, and various wood decay rates in landfills for the time horizon impact of 100 years ($GWP100_{dynamic}$ and $GWP100_{static}$). a An average of 0.9% of the discarded wood is subject to decay in landfills, **b** An average of 10.5% of the discarded wood is subject to decay in landfills, **c** An average of 23.2% of the discarded wood is

subject to decay in landfills, **d** An average of 50% of the discarded wood is subject to decay in landfills. Uncertainties are based on shared socioeconomic pathways (SSPs). The contribution analysis (i.e., heights of bars for each variable), red dots and texts for each scenario represent SSP2. Red error bars quantify uncertainty based on SSP1 and SSP5. Start-of-life scenarios for future urban buildings cover the construction period between 2025 and 2100: BAU, reinforced OPC concrete cities; LC^3, reinforced LC^3 concrete cities; EST, timber cities. End-of-life scenarios for future urban buildings cover the demolition period between 2125 and 2200: C1, concrete is partly recycled, and partly discarded to landfills; C2, concrete is recycled; C3, concrete is discarded to landfills; T1, engineered timber is partly recycled into lower-quality products, partly incinerated, and partly discarded to landfills; T2, engineered timber is incinerated for bioenergy; T3, engineered timber is reused as functionally equivalent products, and this removes the incentive for forest regrowth; T4, engineered timber is recycled into lower quality and/or value products, and this removes the incentive for forest regrowth; T5, engineered timber is partly reused, partly recycled into lower quality/value products, and forestry and land management practices are applied to sustain the forest regrowth; T6, engineered timber is discarded to landfills with direct LFGs release; T7, engineered timber is discarded to landfills, and LFGs are treated by enclosed flare; T8, engineered timber is discarded to landfills, and LFGs are captured and burned for energy production.

S1.1.8. Future urban population growth and building area

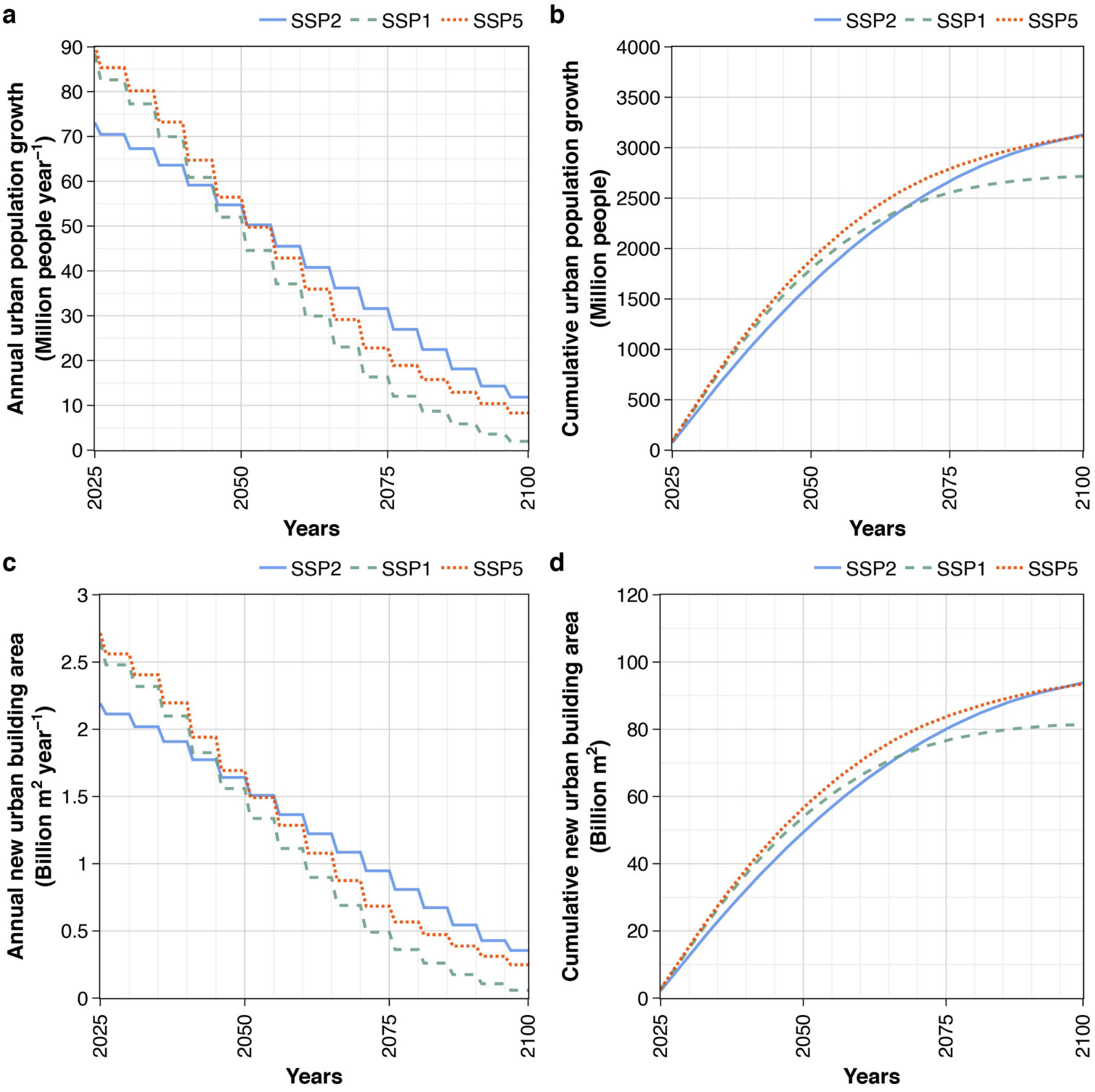

Supplementary Fig. S15 | Annual and cumulative future urban population growth and mid-rise residential and commercial urban building area projections. a Annual urban population growth, **b** Cumulative urban population growth, **c** Annual new urban building area, **d** Cumulative new urban building area. Building areas were calculated based on the urban population growth of the countries based on shared socioeconomic pathways (SSPs). SSP2 (solid line) represents the default pathway. SSP1 (dashed line) and SSP5 (dotted line) quantify uncertainty. All new urban population is assumed to live in buildings with a 30 m2/capita average floor area.

S1.2. Regional level

S1.2.1. Absolute global temperature potential by region

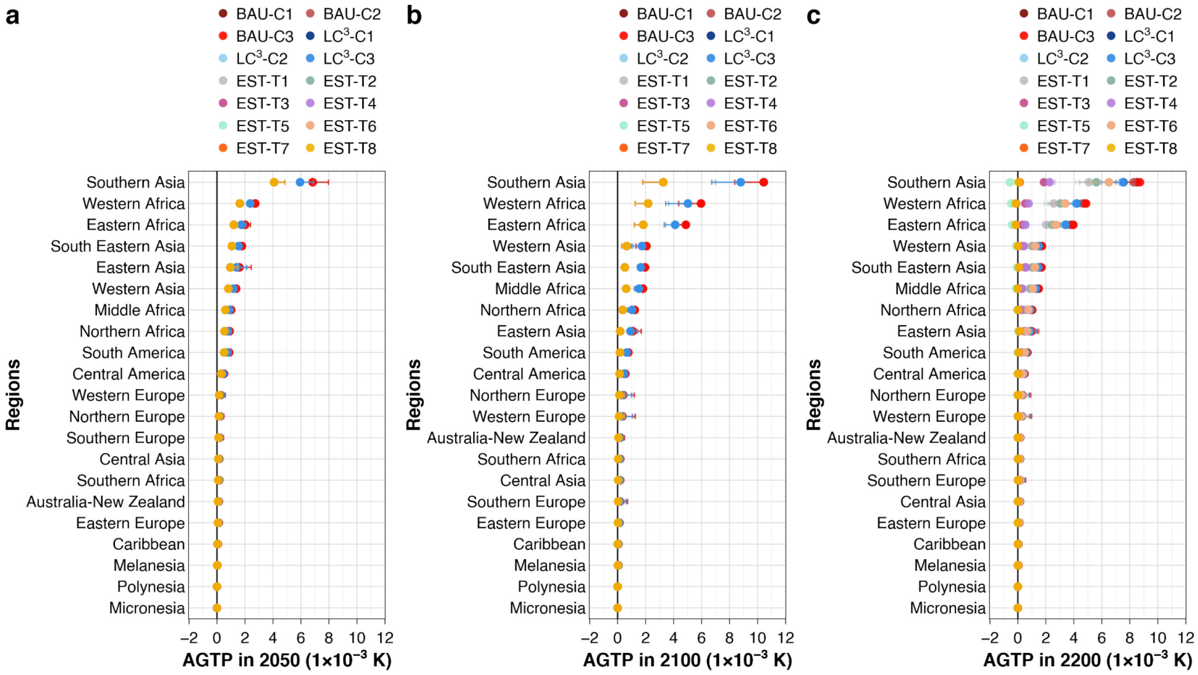

Supplementary Fig. S16 | Geographic breakdown of absolute global temperature potential (AGTP) of future cities in 2050, 2100, and 2100 based on start-of-life and end-of-life urban building scenarios. a AGTP of United Nations subregions in 2050, **b** AGTP of United Nations subregions in 2100, **c** AGTP of United Nations subregions in 2200. Uncertainties are based on shared socioeconomic pathways (SSPs). Large dots based on scenario colour represent SSP2, and error bars quantify uncertainty based on SSP1 and SSP5. Start-of-life scenarios for future urban buildings cover the construction period between 2025 and 2100: BAU, reinforced OPC concrete cities; LC^3, reinforced LC^3 concrete cities; EST, timber cities. End-of-life scenarios for future urban buildings cover the demolition period between 2125 and 2200: C1, concrete is partly recycled, and partly discarded to landfills; C2, concrete is recycled; C3, concrete is discarded to landfills; T1, engineered timber is partly recycled into lower-quality products, partly incinerated, and partly discarded to landfills; T2, engineered timber is incinerated for bioenergy; T3, engineered timber is reused as functionally equivalent products, and this removes the incentive for forest regrowth; T4, engineered timber is recycled into lower quality and/or value products, and this removes the incentive for forest regrowth; T5, engineered timber is partly reused, partly recycled into lower quality/value products, and forestry and land management practices are applied to sustain the forest regrowth; T6, engineered timber is discarded to landfills with direct landfill gases (LFGs) release; T7, engineered timber is discarded to landfills, and LFGs are treated by enclosed flare; T8, engineered timber is discarded to landfills, and LFGs are captured and burned for energy production.

S1.2.2. Global warming potential by region

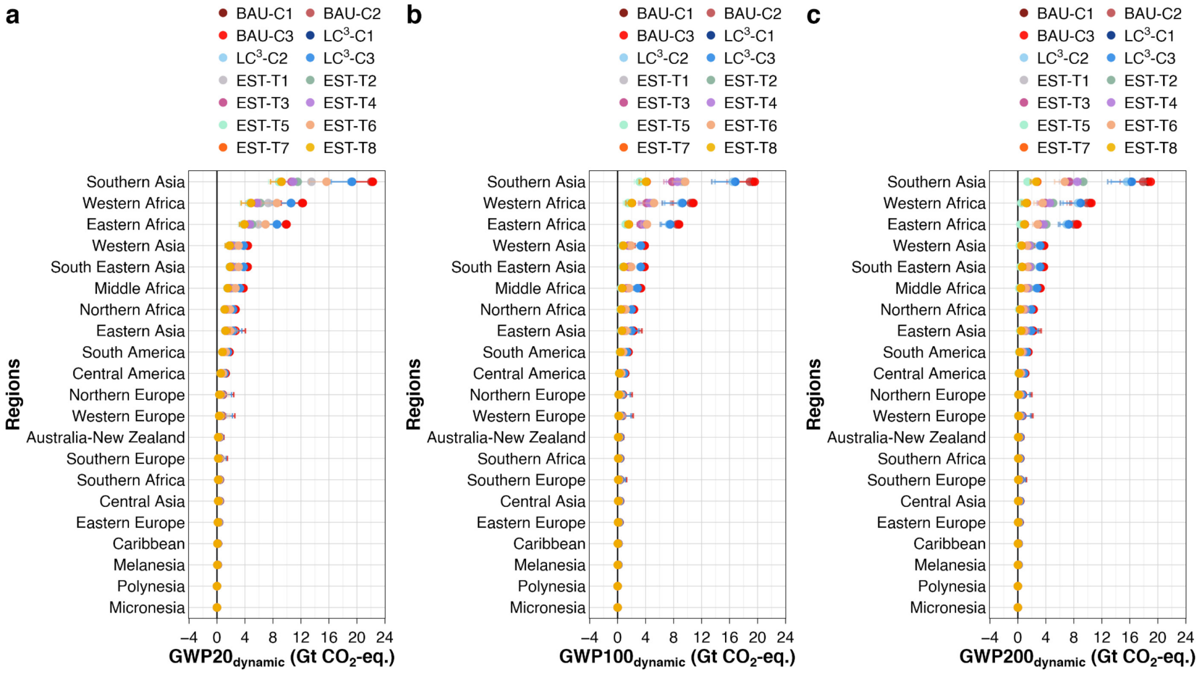

Supplementary Fig. S17 | Geographic breakdown of dynamic global warming potential (GWP) results of future cities based on start-of-life and end-of-life urban building scenarios for time horizon impacts of 20, 100, and 200 years. a $GWP20_{dynamic}$ results of United Nations subregions, **b** $GWP100_{dynamic}$ results of United Nations subregions, **c** $GWP200_{dynamic}$ results of United Nations subregions. Uncertainties are based on shared socioeconomic pathways (SSPs). Large dots based on scenario colour represent SSP2, and error bars quantify uncertainty based on SSP1 and SSP5. Start-of-life scenarios for future urban buildings cover the construction period between 2025 and 2100: BAU, reinforced OPC concrete cities; LC^3, reinforced LC^3 concrete cities; EST, timber cities. End-of-life scenarios for future urban buildings cover the demolition period between 2125 and 2200: C1, concrete is partly recycled, and partly discarded to landfills; C2, concrete is recycled; C3, concrete is discarded to landfills; T1, engineered timber is partly recycled into lower-quality products, partly incinerated, and partly discarded to landfills; T2, engineered timber is incinerated for bioenergy; T3, engineered timber is reused as functionally equivalent products, and this removes the incentive for forest regrowth; T4, engineered timber is recycled into lower quality and/or value products, and this removes the incentive for forest regrowth; T5, engineered timber is partly reused, partly recycled into lower quality/value products, and forestry and land management practices are applied to sustain the forest regrowth; T6, engineered timber is discarded to landfills with direct landfill gases (LFGs) release; T7, engineered timber is discarded to landfills, and LFGs are treated by enclosed flare; T8, engineered timber is discarded to landfills, and LFGs are captured and burned for energy production.

S1.2.3. Southern Asia

S1.2.3.1. Absolute global temperature potential

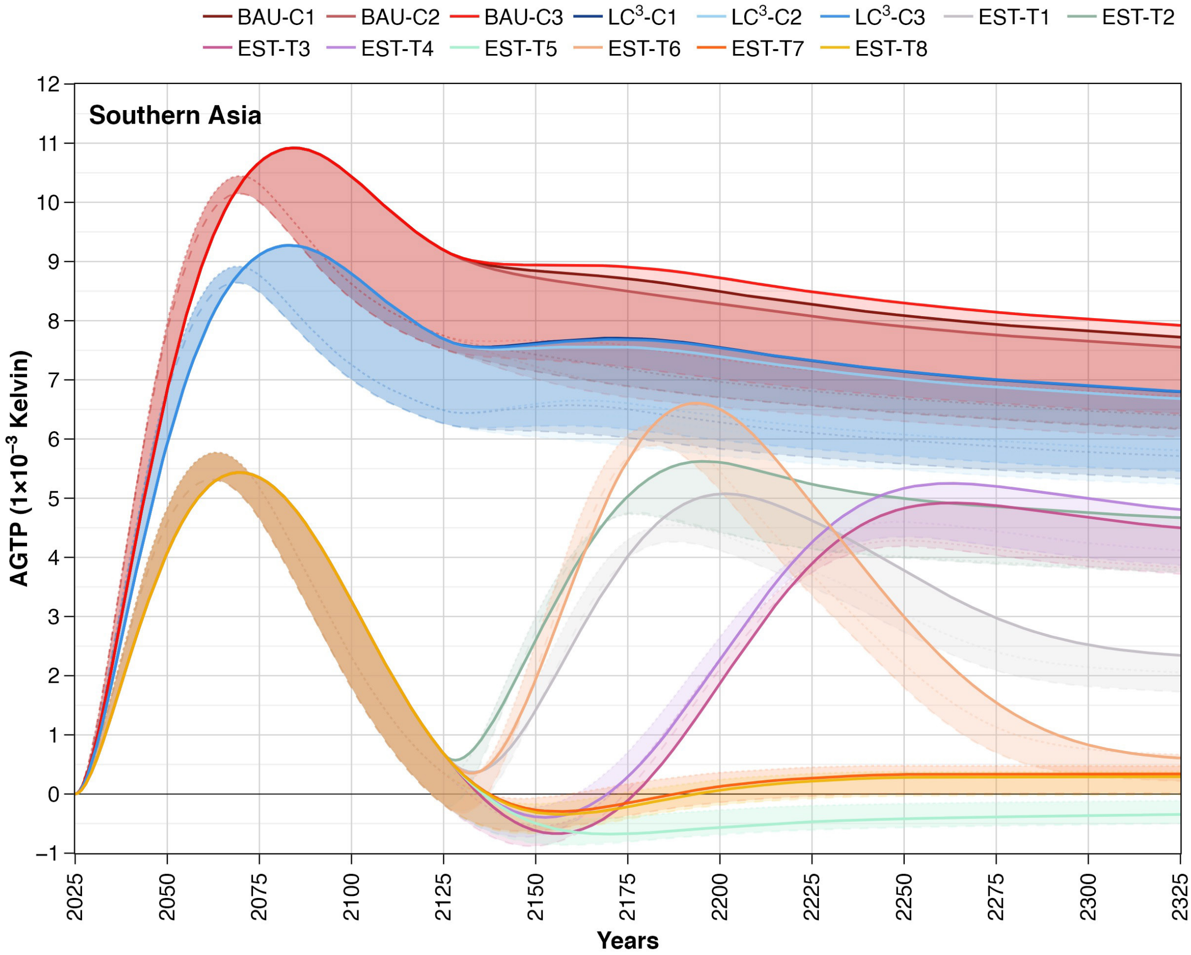

Supplementary Fig. S18 | Absolute global temperature potential (AGTP) of future cities for Southern Asia between 2025 and 2325 based on start-of-life and end-of-life urban building scenarios. For each scenario, the solid line (SSP2), the dashed line (SSP1), and the dotted line (SSP5) represent shared socioeconomic pathways (SSPs), with the shaded area between these lines quantifying uncertainty. Start-of-life scenarios for future urban buildings cover the construction period between 2025 and 2100: BAU, reinforced OPC concrete cities; LC^3, reinforced LC^3 concrete cities; EST, timber cities. End-of-life scenarios for future urban buildings cover the demolition period between 2125 and 2200: C1, concrete is partly recycled, and partly discarded to landfills; C2, concrete is recycled; C3, concrete is discarded to landfills; T1, engineered timber is partly recycled into lower-quality products, partly incinerated, and partly discarded to landfills; T2, engineered timber is incinerated for bioenergy; T3, engineered timber is reused as functionally equivalent products, and this removes the incentive for forest regrowth; T4, engineered timber is recycled into lower quality and/or value products, and this removes the incentive for forest regrowth; T5, engineered timber is partly reused, partly recycled into lower quality/value products, and forestry and land management practices are applied to sustain the forest regrowth; T6, engineered timber is discarded to landfills with direct landfill gases (LFGs) release; T7, engineered timber is discarded to landfills, and LFGs are treated by enclosed flare; T8, engineered timber is discarded to landfills, and LFGs are captured and burned for energy production.

S1.2.3.2. Global warming potential

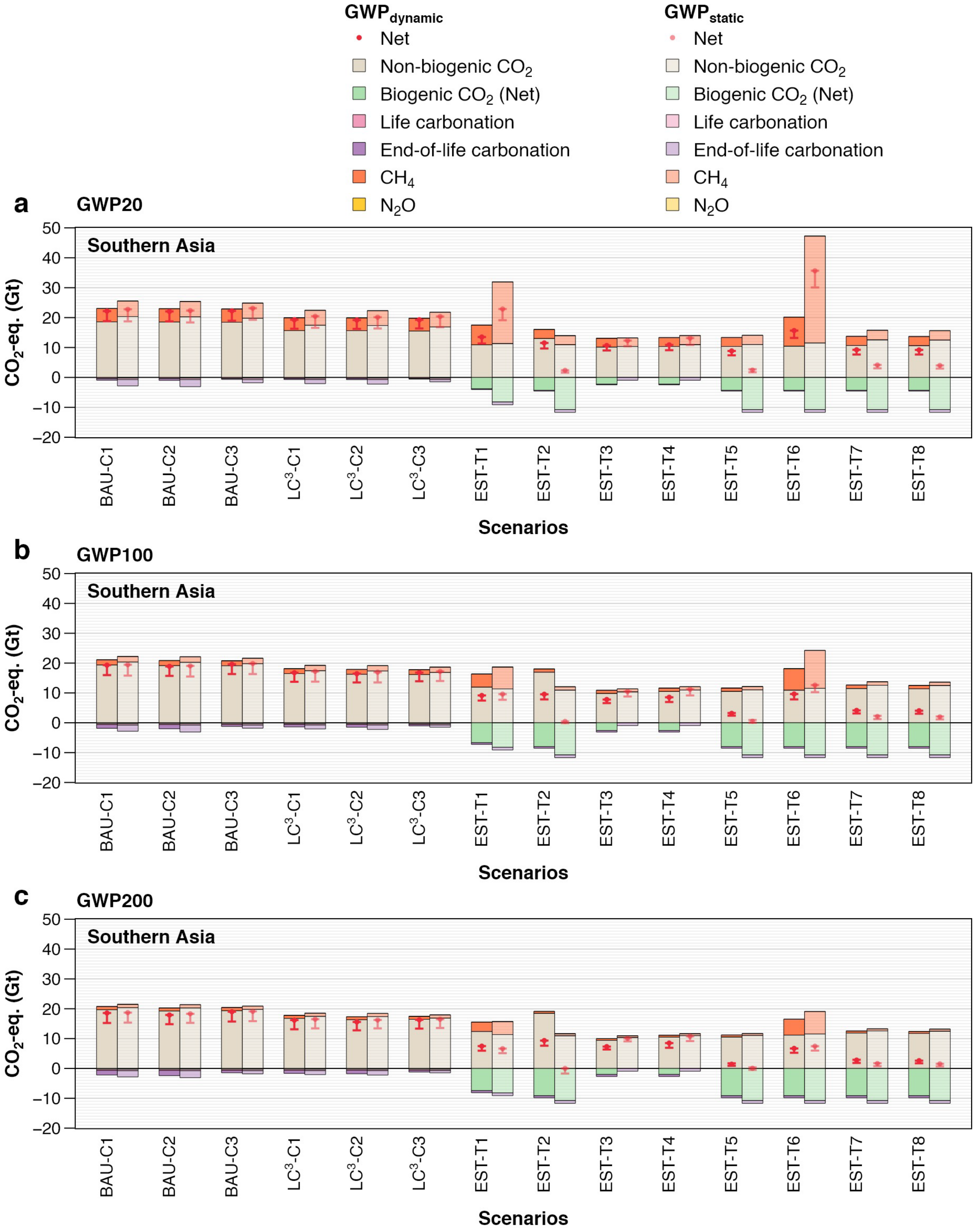

Supplementary Fig. S19 | Dynamic and static global warming potential (GWP) results of future cities for Southern Asia based on start-of-life and end-of-life urban building scenarios for time horizon impacts of 20, 100 and 200 years. a GWP20$_{dynamic}$ and GWP20$_{static}$ results, **b** GWP100$_{dynamic}$ and GWP100$_{static}$ results, **c** GWP200$_{dynamic}$ and GWP200$_{static}$ results. Uncertainties are based on shared socioeconomic pathways (SSPs). The contribution analysis (i.e., heights of bars for each variable), red dots and texts for each scenario represent SSP2. Red error bars quantify uncertainty based on SSP1 and SSP5. Start-of-life scenarios for future urban buildings cover the construction period between 2025 and 2100: BAU, reinforced OPC concrete cities; LC^3, reinforced LC^3 concrete

cities; EST, timber cities. End-of-life scenarios for future urban buildings cover the demolition period between 2125 and 2200: C1, concrete is partly recycled, and partly discarded to landfills; C2, concrete is recycled; C3, concrete is discarded to landfills; T1, engineered timber is partly recycled into lower-quality products, partly incinerated, and partly discarded to landfills; T2, engineered timber is incinerated for bioenergy; T3, engineered timber is reused as functionally equivalent products, and this removes the incentive for forest regrowth; T4, engineered timber is recycled into lower quality and/or value products, and this removes the incentive for forest regrowth; T5, engineered timber is partly reused, partly recycled into lower quality/value products, and forestry and land management practices are applied to sustain the forest regrowth; T6, engineered timber is discarded to landfills with direct landfill gases (LFGs) release; T7, engineered timber is discarded to landfills, and LFGs are treated by enclosed flare; T8, engineered timber is discarded to landfills, and LFGs are captured and burned for energy production.

S1.2.4. Western Africa

S1.2.4.1. Absolute global temperature potential

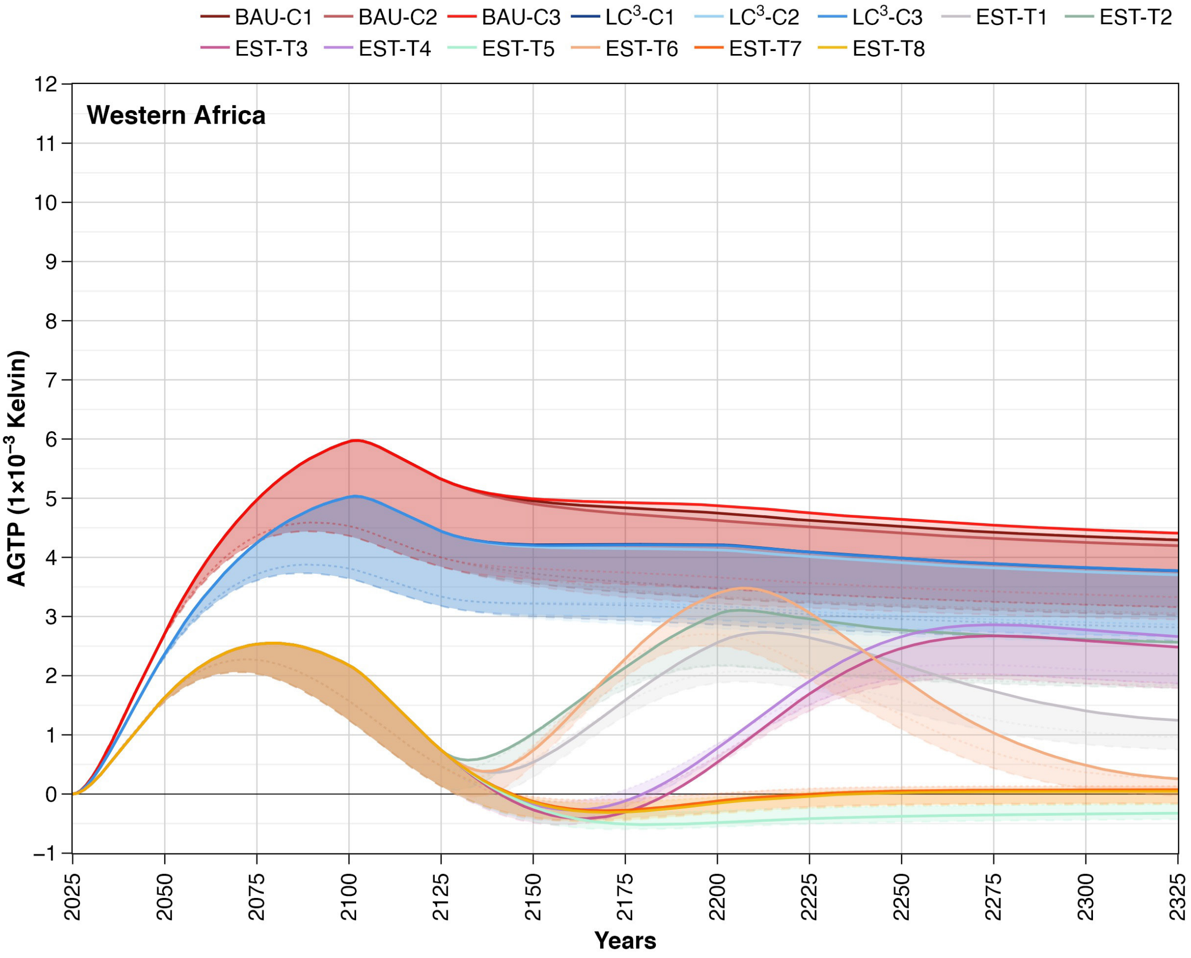

Supplementary Fig. S20 | Absolute global temperature potential (AGTP) of future cities for Western Africa between 2025 and 2325 based on start-of-life and end-of-life urban building scenarios. For each scenario, the solid line (SSP2), the dashed line (SSP1), and the dotted line (SSP5) represent shared socioeconomic pathways (SSPs), with the shaded area between these lines quantifying uncertainty. Start-of-life scenarios for future urban buildings cover the construction period between 2025 and 2100: BAU, reinforced OPC concrete cities; LC^3, reinforced LC^3 concrete cities; EST, timber cities. End-of-life scenarios for future urban buildings cover the demolition period between 2125 and 2200: C1, concrete is partly recycled, and partly discarded to landfills; C2, concrete is recycled; C3, concrete is discarded to landfills; T1, engineered timber is partly recycled into lower-quality products, partly incinerated, and partly discarded to landfills; T2, engineered timber is incinerated for bioenergy; T3, engineered timber is reused as functionally equivalent products, and this removes the incentive for forest regrowth; T4, engineered timber is recycled into lower quality and/or value products, and this removes the incentive for forest regrowth; T5, engineered timber is partly reused, partly recycled into lower quality/value products, and forestry and land management practices are applied to sustain the forest regrowth; T6, engineered timber is discarded to landfills with direct landfill gases (LFGs) release; T7, engineered timber is discarded to landfills, and LFGs are treated by enclosed flare; T8, engineered timber is discarded to landfills, and LFGs are captured and burned for energy production.

S1.2.4.2. Global warming potential

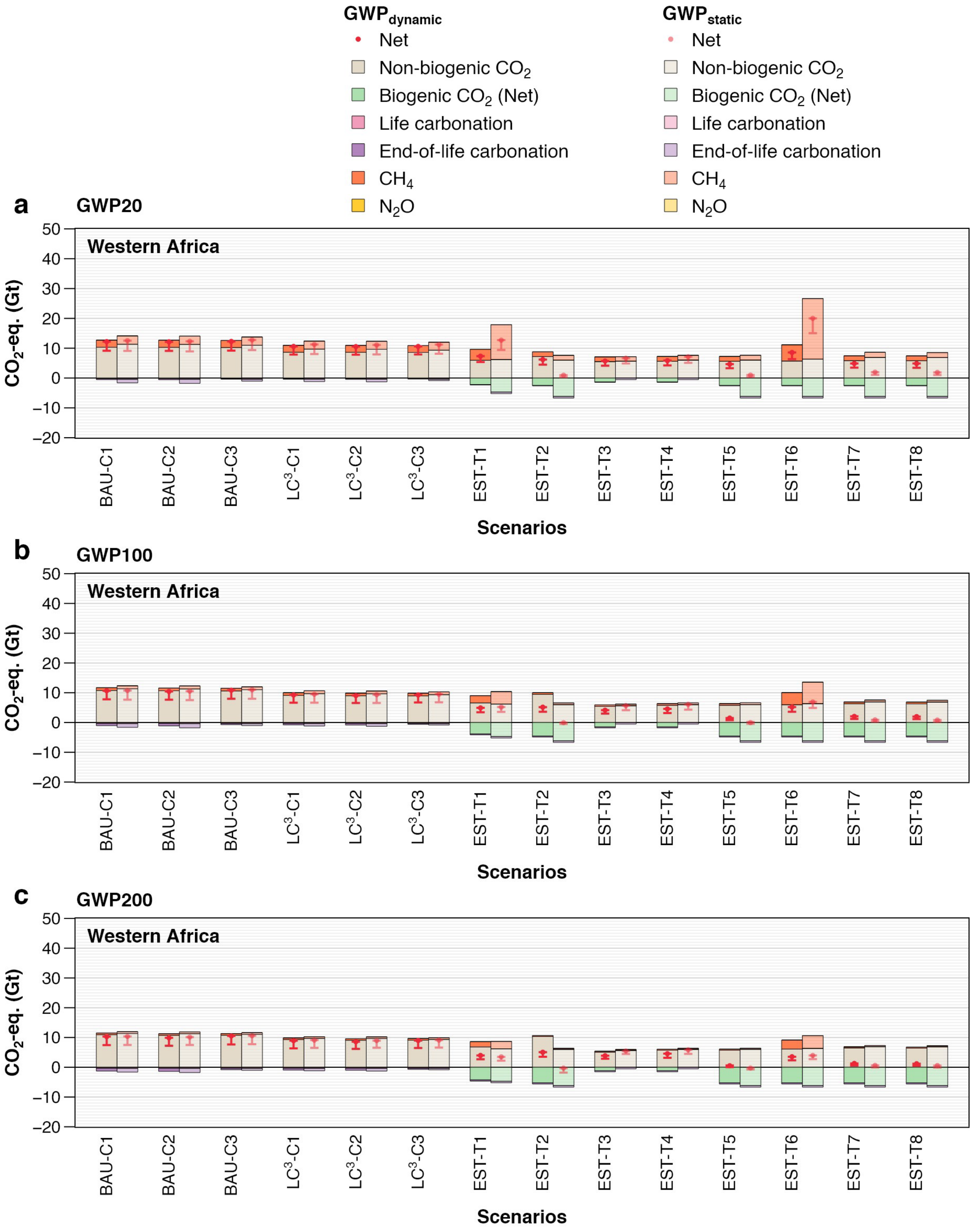

Supplementary Fig. S21 | Dynamic and static global warming potential (GWP) results of future cities for Western Africa based on start-of-life and end-of-life urban building scenarios for time horizon impacts of 20, 100 and 200 years. a $GWP20_{dynamic}$ and $GWP20_{static}$ results, **b** $GWP100_{dynamic}$ and $GWP100_{static}$ results, **c** $GWP200_{dynamic}$ and $GWP200_{static}$ results. Uncertainties are based on shared socioeconomic pathways (SSPs). The contribution analysis (i.e., heights of bars for each variable), red dots and texts for each scenario represent SSP2. Red error bars quantify uncertainty based on SSP1 and SSP5. Start-of-life scenarios for future urban buildings cover the construction period between 2025 and 2100: BAU, reinforced OPC concrete cities; LC^3, reinforced LC^3 concrete

cities; EST, timber cities. End-of-life scenarios for future urban buildings cover the demolition period between 2125 and 2200: C1, concrete is partly recycled, and partly discarded to landfills; C2, concrete is recycled; C3, concrete is discarded to landfills; T1, engineered timber is partly recycled into lower-quality products, partly incinerated, and partly discarded to landfills; T2, engineered timber is incinerated for bioenergy; T3, engineered timber is reused as functionally equivalent products, and this removes the incentive for forest regrowth; T4, engineered timber is recycled into lower quality and/or value products, and this removes the incentive for forest regrowth; T5, engineered timber is partly reused, partly recycled into lower quality/value products, and forestry and land management practices are applied to sustain the forest regrowth; T6, engineered timber is discarded to landfills with direct landfill gases (LFGs) release; T7, engineered timber is discarded to landfills, and LFGs are treated by enclosed flare; T8, engineered timber is discarded to landfills, and LFGs are captured and burned for energy production.

S1.2.5. Eastern Africa

S1.2.5.1. Absolute global temperature potential

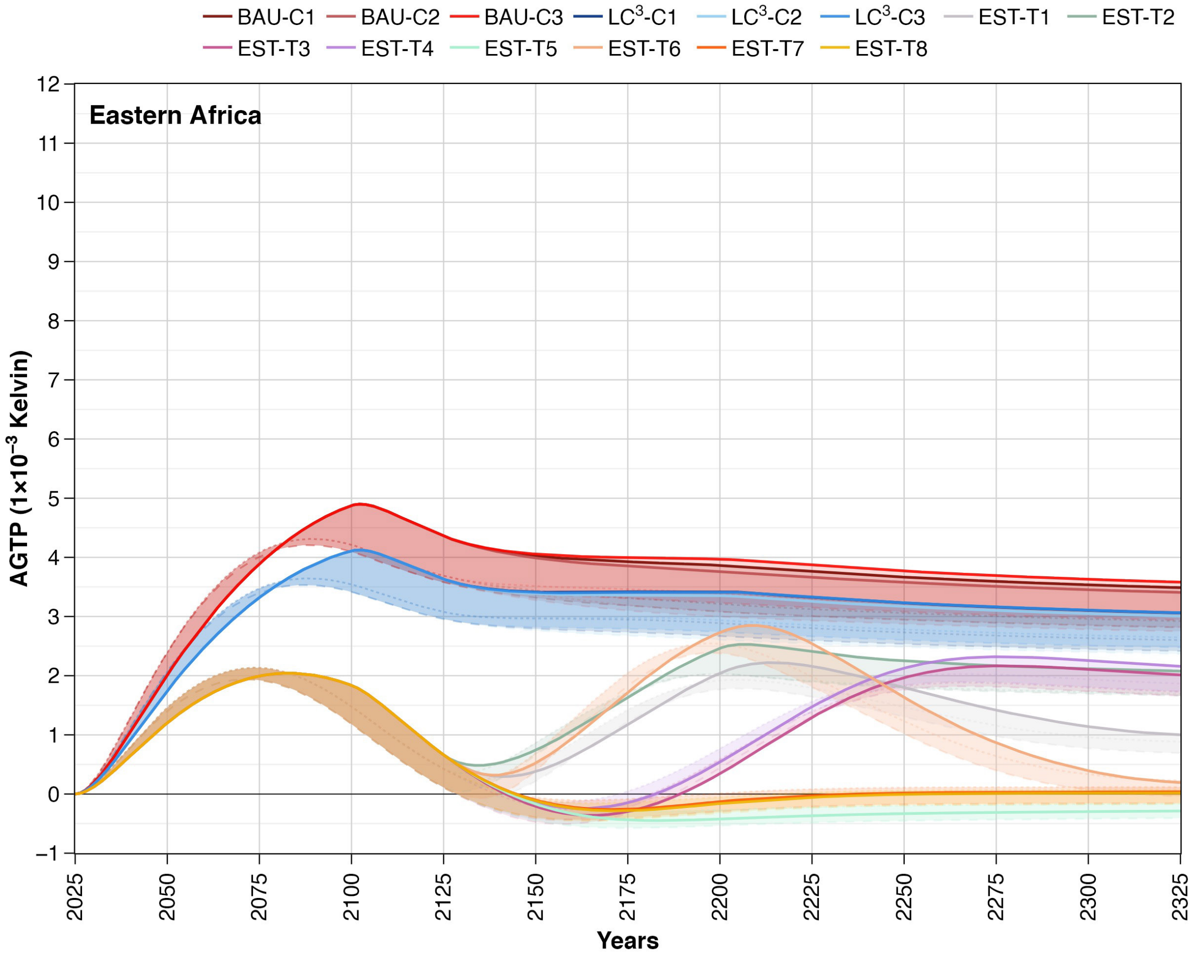

Supplementary Fig. S22 | Absolute global temperature potential (AGTP) of future cities for Eastern Africa between 2025 and 2325 based on start-of-life and end-of-life urban building scenarios. For each scenario, the solid line (SSP2), the dashed line (SSP1), and the dotted line (SSP5) represent shared socioeconomic pathways (SSPs), with the shaded area between these lines quantifying uncertainty. Start-of-life scenarios for future urban buildings cover the construction period between 2025 and 2100: BAU, reinforced OPC concrete cities; LC^3, reinforced LC^3 concrete cities; EST, timber cities. End-of-life scenarios for future urban buildings cover the demolition period between 2125 and 2200: C1, concrete is partly recycled, and partly discarded to landfills; C2, concrete is recycled; C3, concrete is discarded to landfills; T1, engineered timber is partly recycled into lower-quality products, partly incinerated, and partly discarded to landfills; T2, engineered timber is incinerated for bioenergy; T3, engineered timber is reused as functionally equivalent products, and this removes the incentive for forest regrowth; T4, engineered timber is recycled into lower quality and/or value products, and this removes the incentive for forest regrowth; T5, engineered timber is partly reused, partly recycled into lower quality/value products, and forestry and land management practices are applied to sustain the forest regrowth; T6, engineered timber is discarded to landfills with direct landfill gases (LFGs) release; T7, engineered timber is discarded to landfills, and LFGs are treated by enclosed flare; T8, engineered timber is discarded to landfills, and LFGs are captured and burned for energy production.

S1.2.5.2. Global warming potential

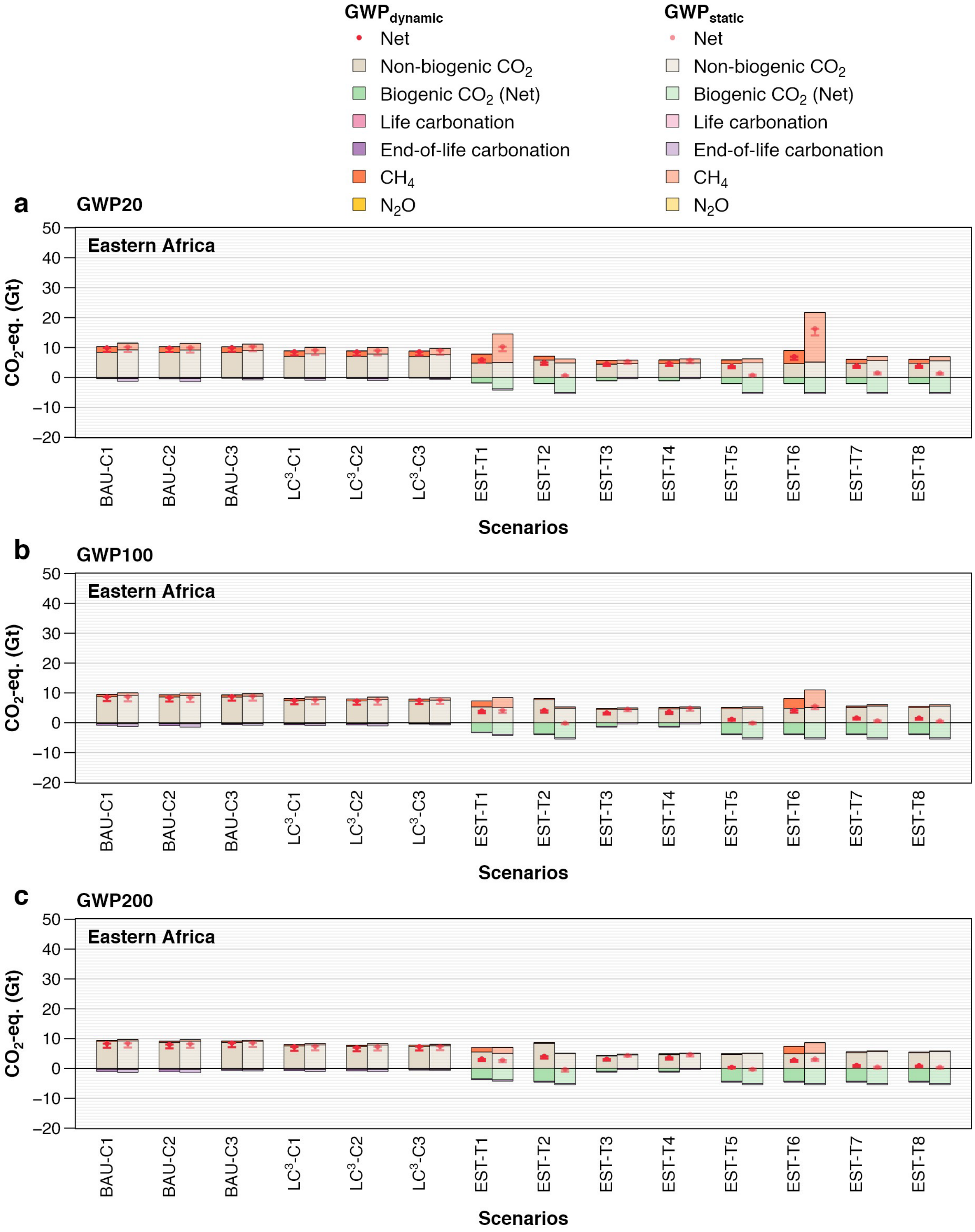

Supplementary Fig. S23 | Dynamic and static global warming potential (GWP) results of future cities for Eastern Africa based on start-of-life and end-of-life urban building scenarios for time horizon impacts of 20, 100 and 200 years. a $GWP20_{dynamic}$ and $GWP20_{static}$ results, **b** $GWP100_{dynamic}$ and $GWP100_{static}$ results, **c** $GWP200_{dynamic}$ and $GWP200_{static}$ results. Uncertainties are based on shared socioeconomic pathways (SSPs). The contribution analysis (i.e., heights of bars for each variable), red dots and texts for each scenario represent SSP2. Red error bars quantify uncertainty based on SSP1 and SSP5. Start-of-life scenarios for future urban buildings cover the construction period between 2025 and 2100: BAU, reinforced OPC concrete cities; LC^3, reinforced LC^3 concrete

cities; EST, timber cities. End-of-life scenarios for future urban buildings cover the demolition period between 2125 and 2200: C1, concrete is partly recycled, and partly discarded to landfills; C2, concrete is recycled; C3, concrete is discarded to landfills; T1, engineered timber is partly recycled into lower-quality products, partly incinerated, and partly discarded to landfills; T2, engineered timber is incinerated for bioenergy; T3, engineered timber is reused as functionally equivalent products, and this removes the incentive for forest regrowth; T4, engineered timber is recycled into lower quality and/or value products, and this removes the incentive for forest regrowth; T5, engineered timber is partly reused, partly recycled into lower quality/value products, and forestry and land management practices are applied to sustain the forest regrowth; T6, engineered timber is discarded to landfills with direct landfill gases (LFGs) release; T7, engineered timber is discarded to landfills, and LFGs are treated by enclosed flare; T8, engineered timber is discarded to landfills, and LFGs are captured and burned for energy production.

S1.3. Country level

S1.3.1. Absolute global temperature potential by country

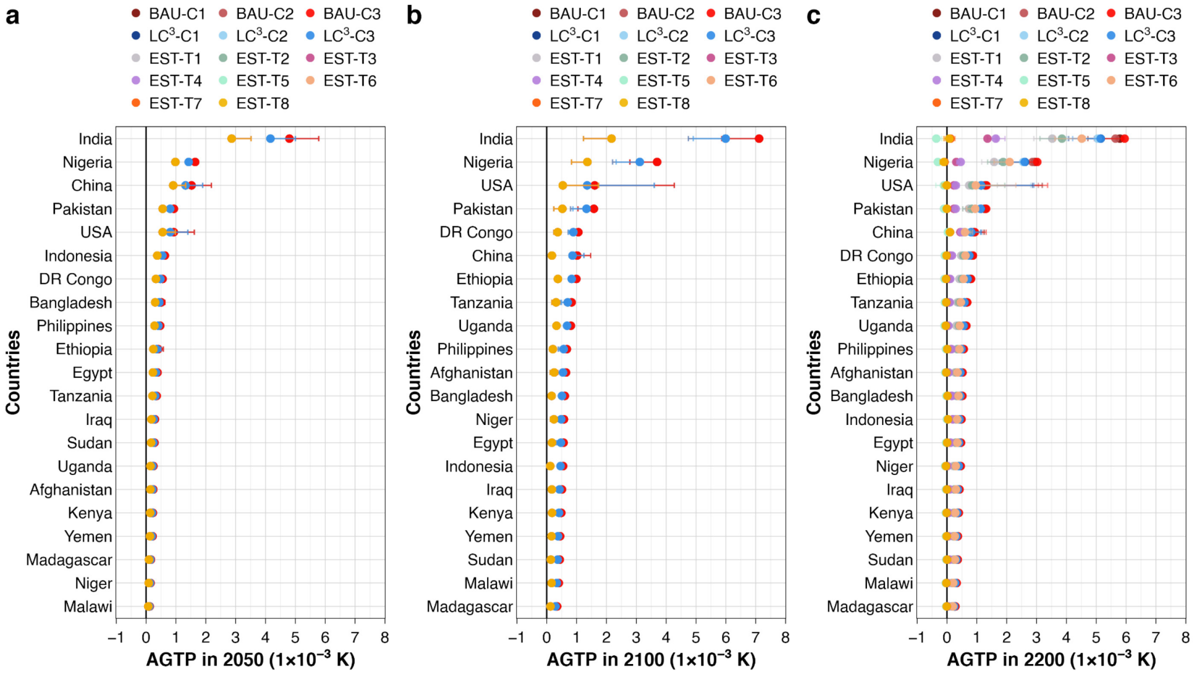

Supplementary Fig. S24 | Geographic breakdown of absolute global temperature potential (AGTP) of future cities in 2050, 2100, and 2100 based on start-of-life and end-of-life urban building scenarios. a The twenty-one countries with the highest AGTP in 2050, **b** The twenty-one countries with the highest AGTP in 2100, **c** The twenty-one countries with the highest AGTP in 2200. Uncertainties are based on shared socioeconomic pathways (SSPs). Large dots based on scenario colour represent SSP2, and error bars quantify uncertainty based on SSP1 and SSP5. Start-of-life scenarios for future urban buildings cover the construction period between 2025 and 2100: BAU, reinforced OPC concrete cities; LC^3, reinforced LC^3 concrete cities; EST, timber cities. End-of-life scenarios for future urban buildings cover the demolition period between 2125 and 2200: C1, concrete is partly recycled, and partly discarded to landfills; C2, concrete is recycled; C3, concrete is discarded to landfills; T1, engineered timber is partly recycled into lower-quality products, partly incinerated, and partly discarded to landfills; T2, engineered timber is incinerated for bioenergy; T3, engineered timber is reused as functionally equivalent products, and this removes the incentive for forest regrowth; T4, engineered timber is recycled into lower quality and/or value products, and this removes the incentive for forest regrowth; T5, engineered timber is partly reused, partly recycled into lower quality/value products, and forestry and land management practices are applied to sustain the forest regrowth; T6, engineered timber is discarded to landfills with direct landfill gases (LFGs) release; T7, engineered timber is discarded to landfills, and LFGs are treated by enclosed flare; T8, engineered timber is discarded to landfills, and LFGs are captured and burned for energy production.

S1.3.2. Global warming potential by country

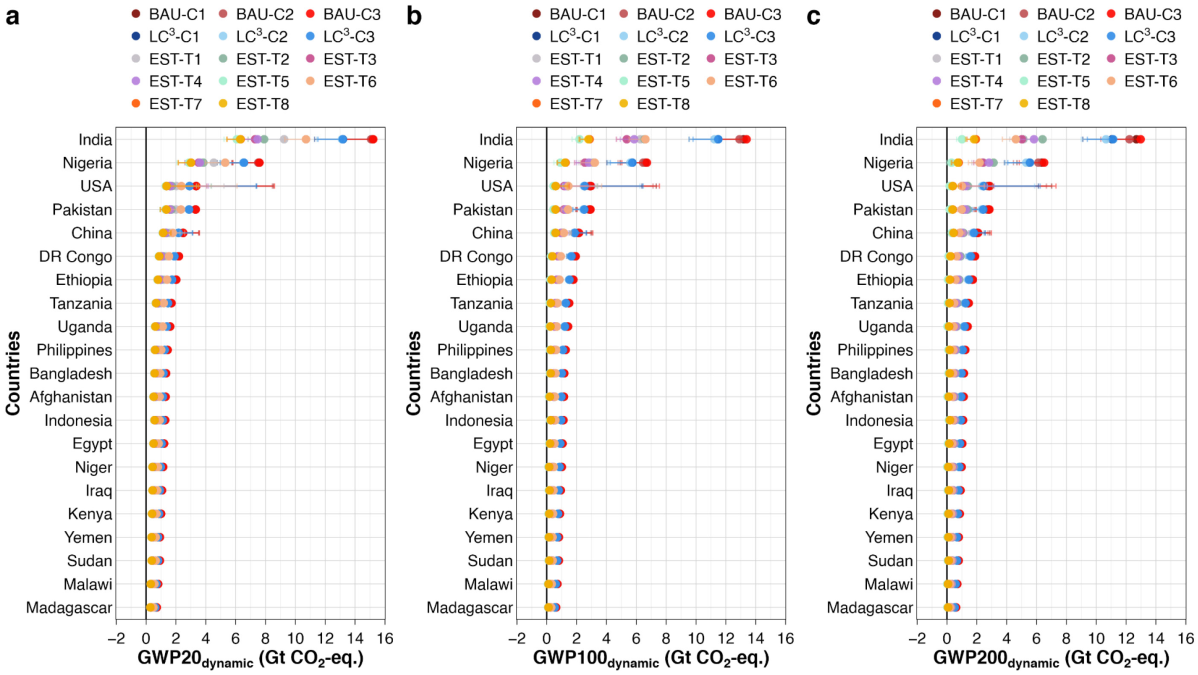

Supplementary Fig. S25 | Geographic breakdown of dynamic global warming potential (GWP) results of future cities based on start-of-life and end-of-life urban building scenarios for time horizon impacts of 20, 100, and 200 years. a The twenty-one countries with the highest $GWP20_{dynamic}$ results, **b** The twenty-one countries with the highest $GWP100_{dynamic}$ results, **c** The twenty-one countries with the highest $GWP200_{dynamic}$ results. Uncertainties are based on shared socioeconomic pathways (SSPs). Large dots based on scenario colour represent SSP2, and error bars quantify uncertainty based on SSP1 and SSP5. Start-of-life scenarios for future urban buildings cover the construction period between 2025 and 2100: BAU, reinforced OPC concrete cities; LC^3, reinforced LC^3 concrete cities; EST, timber cities. End-of-life scenarios for future urban buildings cover the demolition period between 2125 and 2200: C1, concrete is partly recycled, and partly discarded to landfills; C2, concrete is recycled; C3, concrete is discarded to landfills; T1, engineered timber is partly recycled into lower-quality products, partly incinerated, and partly discarded to landfills; T2, engineered timber is incinerated for bioenergy; T3, engineered timber is reused as functionally equivalent products, and this removes the incentive for forest regrowth; T4, engineered timber is recycled into lower quality and/or value products, and this removes the incentive for forest regrowth; T5, engineered timber is partly reused, partly recycled into lower quality/value products, and forestry and land management practices are applied to sustain the forest regrowth; T6, engineered timber is discarded to landfills with direct landfill gases (LFGs) release; T7, engineered timber is discarded to landfills, and LFGs are treated by enclosed flare; T8, engineered timber is discarded to landfills, and LFGs are captured and burned for energy production.

S1.3.3. India

S1.3.3.1. Absolute global temperature potential

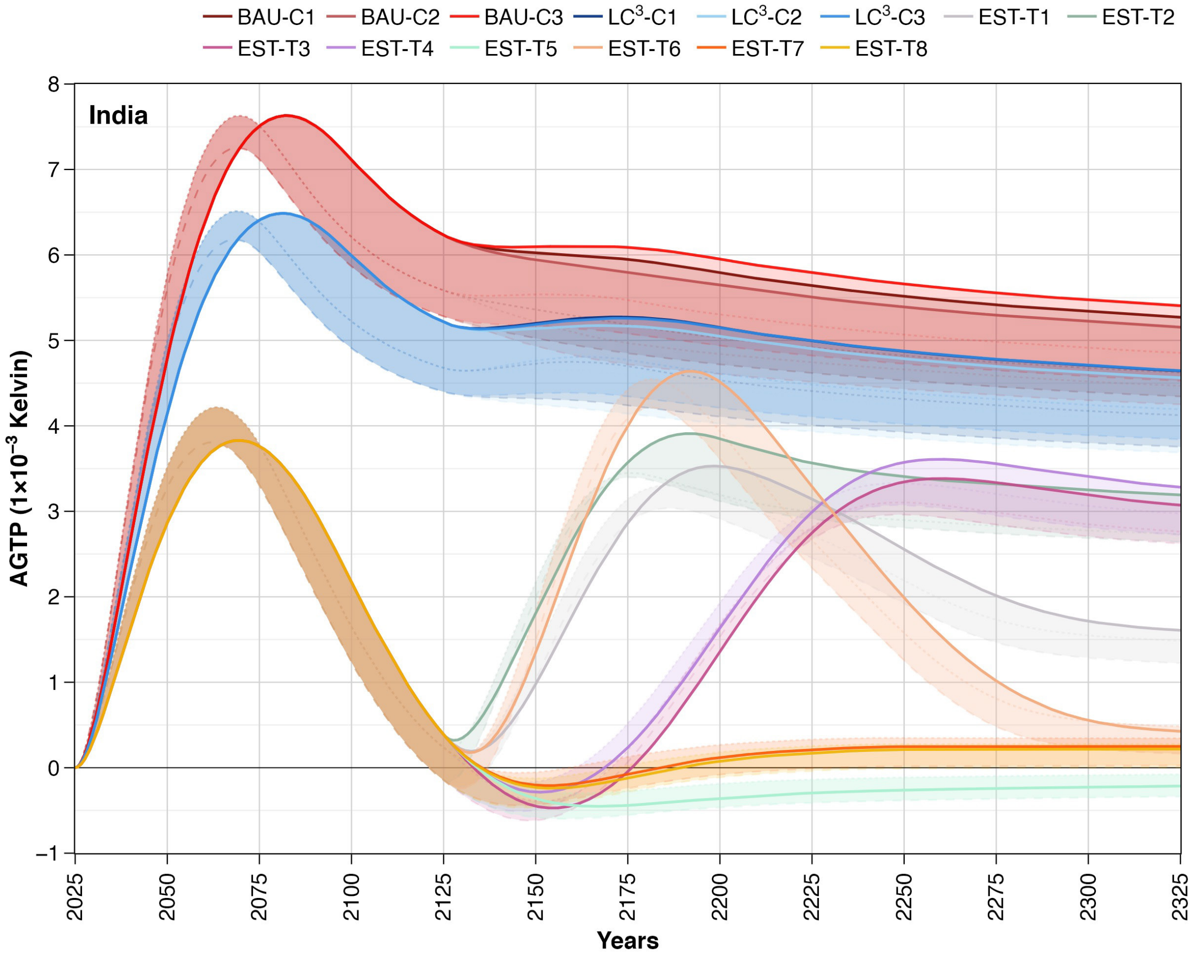

Supplementary Fig. S26 | Absolute global temperature potential (AGTP) of future cities for India between 2025 and 2325 based on start-of-life and end-of-life urban building scenarios. For each scenario, the solid line (SSP2), the dashed line (SSP1), and the dotted line (SSP5) represent shared socioeconomic pathways (SSPs), with the shaded area between these lines quantifying uncertainty. Start-of-life scenarios for future urban buildings cover the construction period between 2025 and 2100: BAU, reinforced OPC concrete cities; LC^3, reinforced LC^3 concrete cities; EST, timber cities. End-of-life scenarios for future urban buildings cover the demolition period between 2125 and 2200: C1, concrete is partly recycled, and partly discarded to landfills; C2, concrete is recycled; C3, concrete is discarded to landfills; T1, engineered timber is partly recycled into lower-quality products, partly incinerated, and partly discarded to landfills; T2, engineered timber is incinerated for bioenergy; T3, engineered timber is reused as functionally equivalent products, and this removes the incentive for forest regrowth; T4, engineered timber is recycled into lower quality and/or value products, and this removes the incentive for forest regrowth; T5, engineered timber is partly reused, partly recycled into lower quality/value products, and forestry and land management practices are applied to sustain the forest regrowth; T6, engineered timber is discarded to landfills with direct landfill gases (LFGs) release; T7, engineered timber is discarded to landfills, and LFGs are treated by enclosed flare; T8, engineered timber is discarded to landfills, and LFGs are captured and burned for energy production.

S1.3.3.2. Global warming potential

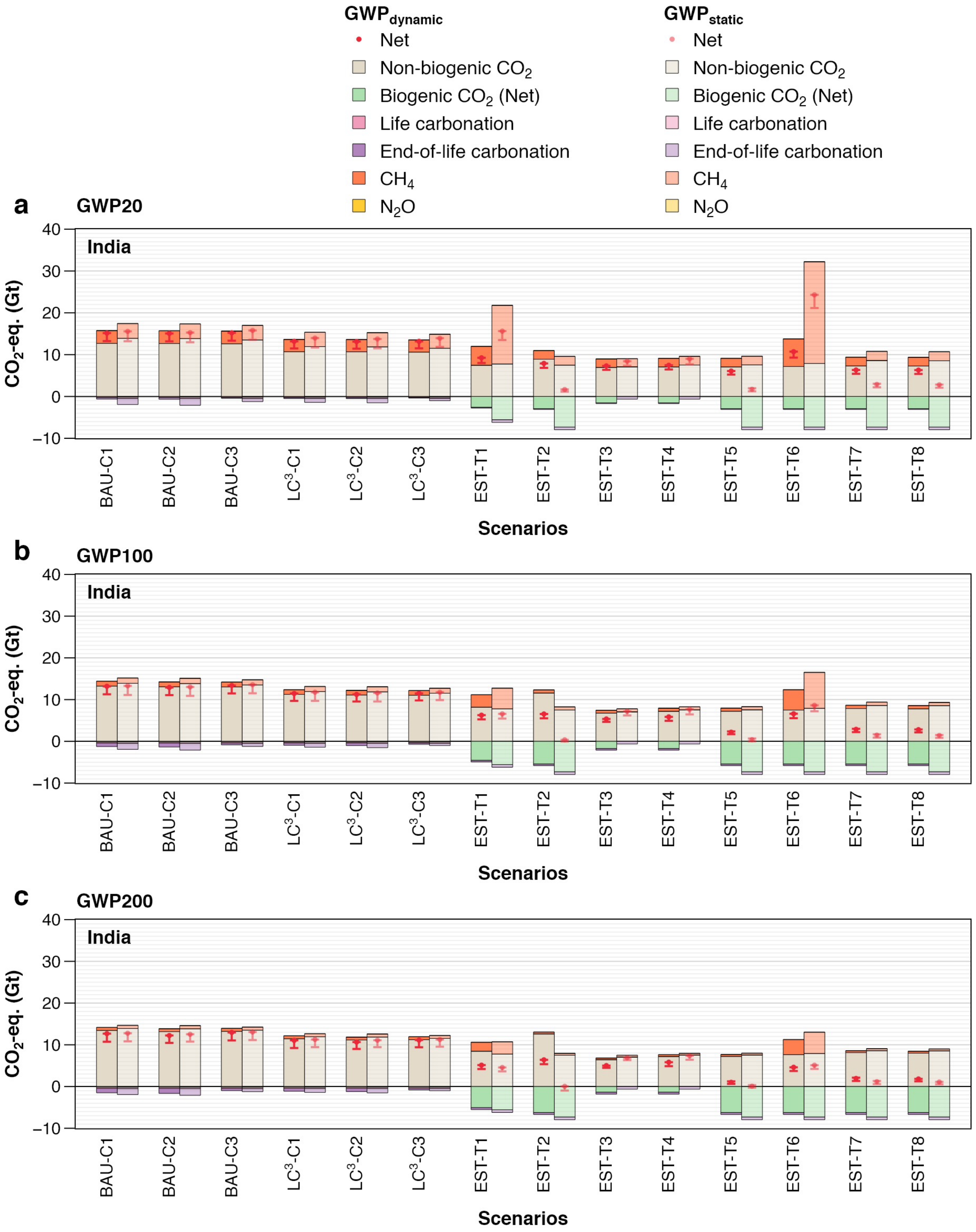

Supplementary Fig. S27 | Dynamic and static global warming potential (GWP) results of future cities for India based on start-of-life and end-of-life urban building scenarios for time horizon impacts of 20, 100 and 200 years. a $GWP20_{dynamic}$ and $GWP20_{static}$ results, **b** $GWP100_{dynamic}$ and $GWP100_{static}$ results, **c** $GWP200_{dynamic}$ and $GWP200_{static}$ results. Uncertainties are based on shared socioeconomic pathways (SSPs). The contribution analysis (i.e., heights of bars for each variable), red dots and texts for each scenario represent SSP2. Red error bars quantify uncertainty based on SSP1 and SSP5. Start-of-life scenarios for future urban buildings cover the construction period between 2025 and 2100: BAU, reinforced OPC concrete cities; LC^3, reinforced LC^3 concrete cities;

EST, timber cities. End-of-life scenarios for future urban buildings cover the demolition period between 2125 and 2200: C1, concrete is partly recycled, and partly discarded to landfills; C2, concrete is recycled; C3, concrete is discarded to landfills; T1, engineered timber is partly recycled into lower-quality products, partly incinerated, and partly discarded to landfills; T2, engineered timber is incinerated for bioenergy; T3, engineered timber is reused as functionally equivalent products, and this removes the incentive for forest regrowth; T4, engineered timber is recycled into lower quality and/or value products, and this removes the incentive for forest regrowth; T5, engineered timber is partly reused, partly recycled into lower quality/value products, and forestry and land management practices are applied to sustain the forest regrowth; T6, engineered timber is discarded to landfills with direct landfill gases (LFGs) release; T7, engineered timber is discarded to landfills, and LFGs are treated by enclosed flare; T8, engineered timber is discarded to landfills, and LFGs are captured and burned for energy production.

S1.3.4. Nigeria

S1.3.4.1. Absolute global temperature potential

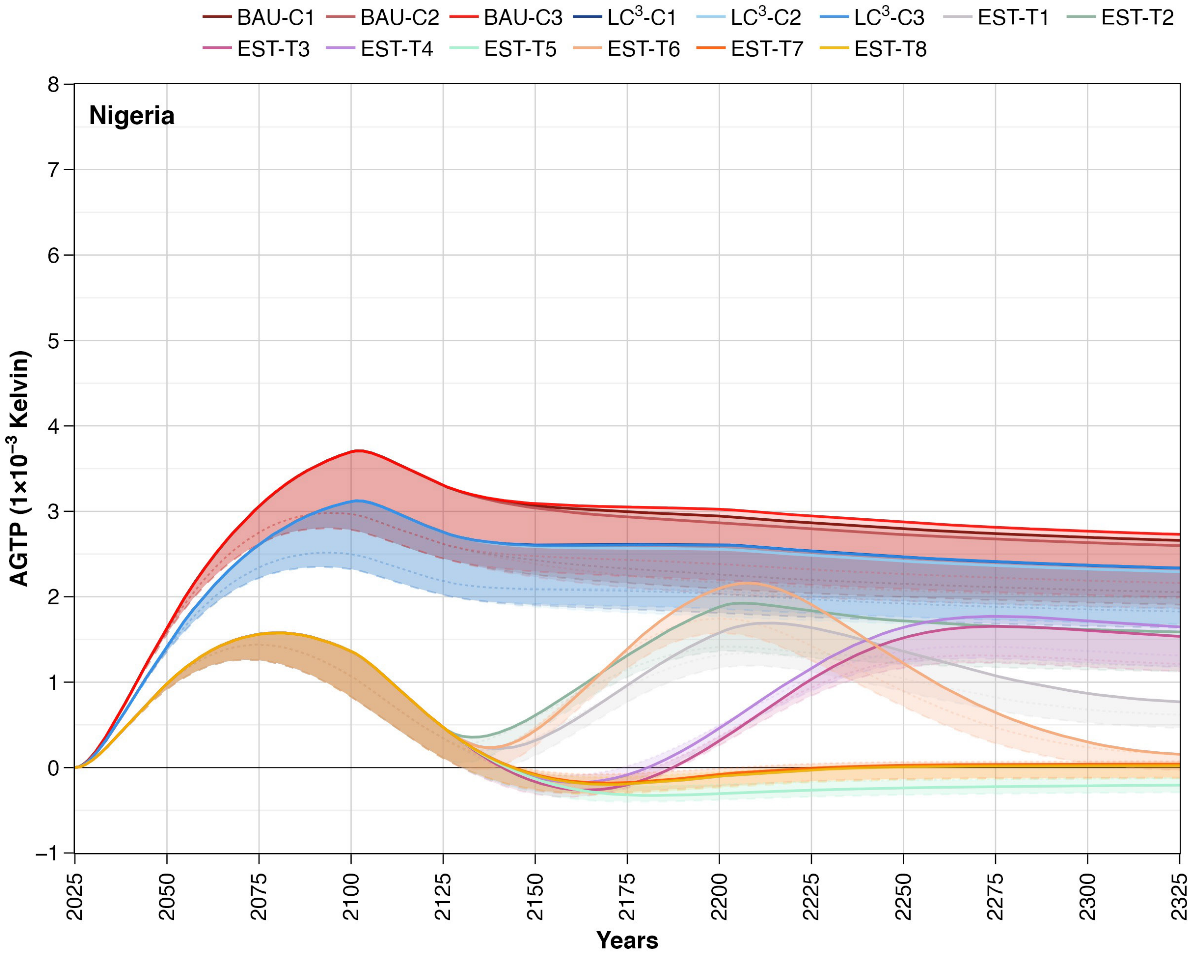

Supplementary Fig. S28 | Absolute global temperature potential (AGTP) of future cities for Nigeria between 2025 and 2325 based on start-of-life and end-of-life urban building scenarios. For each scenario, the solid line (SSP2), the dashed line (SSP1), and the dotted line (SSP5) represent shared socioeconomic pathways (SSPs), with the shaded area between these lines quantifying uncertainty. Start-of-life scenarios for future urban buildings cover the construction period between 2025 and 2100: BAU, reinforced OPC concrete cities; LC^3, reinforced LC^3 concrete cities; EST, timber cities. End-of-life scenarios for future urban buildings cover the demolition period between 2125 and 2200: C1, concrete is partly recycled, and partly discarded to landfills; C2, concrete is recycled; C3, concrete is discarded to landfills; T1, engineered timber is partly recycled into lower-quality products, partly incinerated, and partly discarded to landfills; T2, engineered timber is incinerated for bioenergy; T3, engineered timber is reused as functionally equivalent products, and this removes the incentive for forest regrowth; T4, engineered timber is recycled into lower quality and/or value products, and this removes the incentive for forest regrowth; T5, engineered timber is partly reused, partly recycled into lower quality/value products, and forestry and land management practices are applied to sustain the forest regrowth; T6, engineered timber is discarded to landfills with direct landfill gases (LFGs) release; T7, engineered timber is discarded to landfills, and LFGs are treated by enclosed flare; T8, engineered timber is discarded to landfills, and LFGs are captured and burned for energy production.

S1.3.4.2. Global warming potential

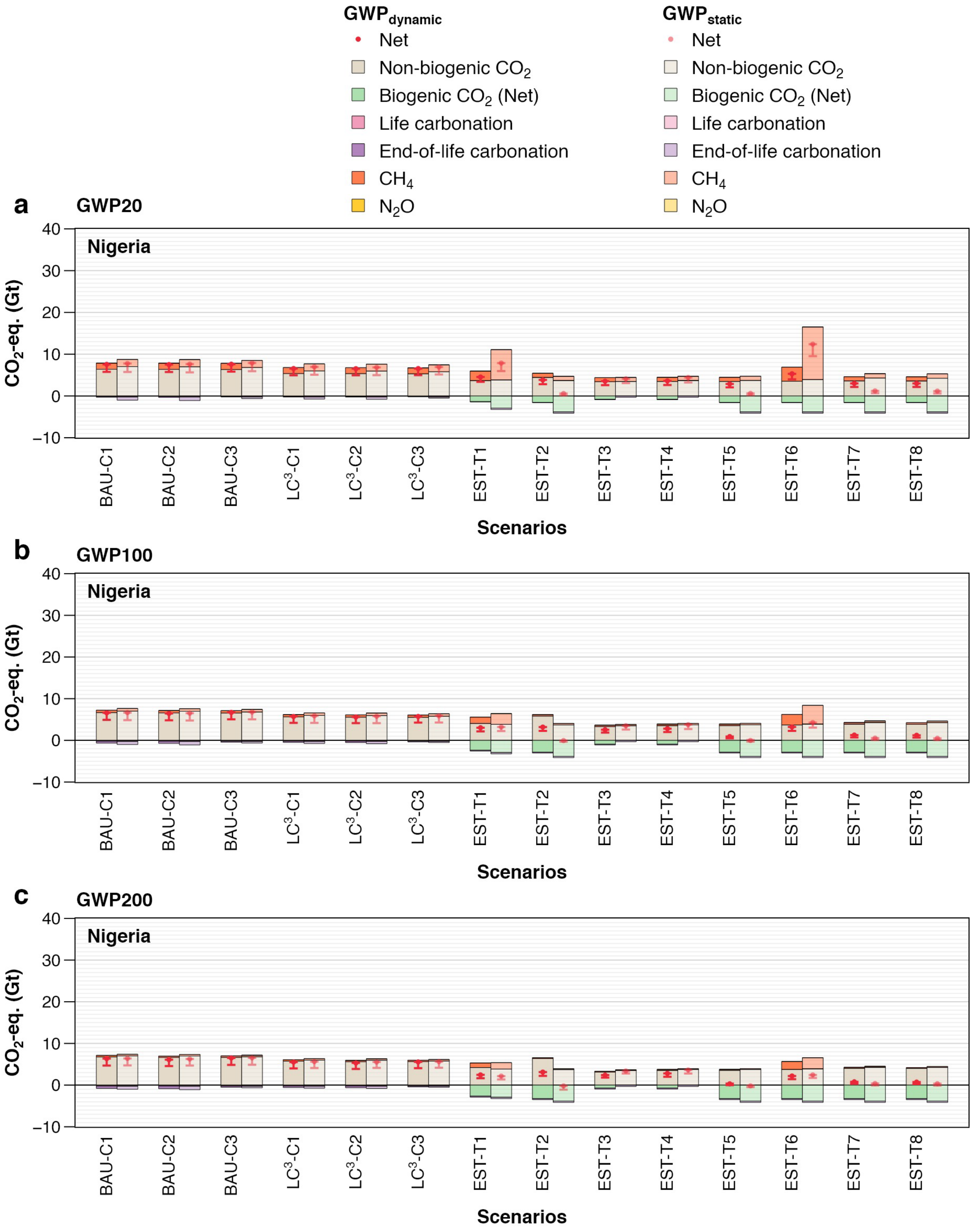

Supplementary Fig. S29 | Dynamic and static global warming potential (GWP) results of future cities for Nigeria based on start-of-life and end-of-life urban building scenarios for time horizon impacts of 20, 100 and 200 years. a GWP20$_{dynamic}$ and GWP20$_{static}$ results, **b** GWP100$_{dynamic}$ and GWP100$_{static}$ results, **c** GWP200$_{dynamic}$ and GWP200$_{static}$ results. Uncertainties are based on shared socioeconomic pathways (SSPs). The contribution analysis (i.e., heights of bars for each variable), red dots and texts for each scenario represent SSP2. Red error bars quantify uncertainty based on SSP1 and SSP5. Start-of-life scenarios for future urban buildings cover the construction period between 2025 and 2100: BAU, reinforced OPC concrete cities; LC^3, reinforced LC^3 concrete

cities; EST, timber cities. End-of-life scenarios for future urban buildings cover the demolition period between 2125 and 2200: C1, concrete is partly recycled, and partly discarded to landfills; C2, concrete is recycled; C3, concrete is discarded to landfills; T1, engineered timber is partly recycled into lower-quality products, partly incinerated, and partly discarded to landfills; T2, engineered timber is incinerated for bioenergy; T3, engineered timber is reused as functionally equivalent products, and this removes the incentive for forest regrowth; T4, engineered timber is recycled into lower quality and/or value products, and this removes the incentive for forest regrowth; T5, engineered timber is partly reused, partly recycled into lower quality/value products, and forestry and land management practices are applied to sustain the forest regrowth; T6, engineered timber is discarded to landfills with direct landfill gases (LFGs) release; T7, engineered timber is discarded to landfills, and LFGs are treated by enclosed flare; T8, engineered timber is discarded to landfills, and LFGs are captured and burned for energy production.

S1.3.5. United States of America

S1.3.5.1. Absolute global temperature potential

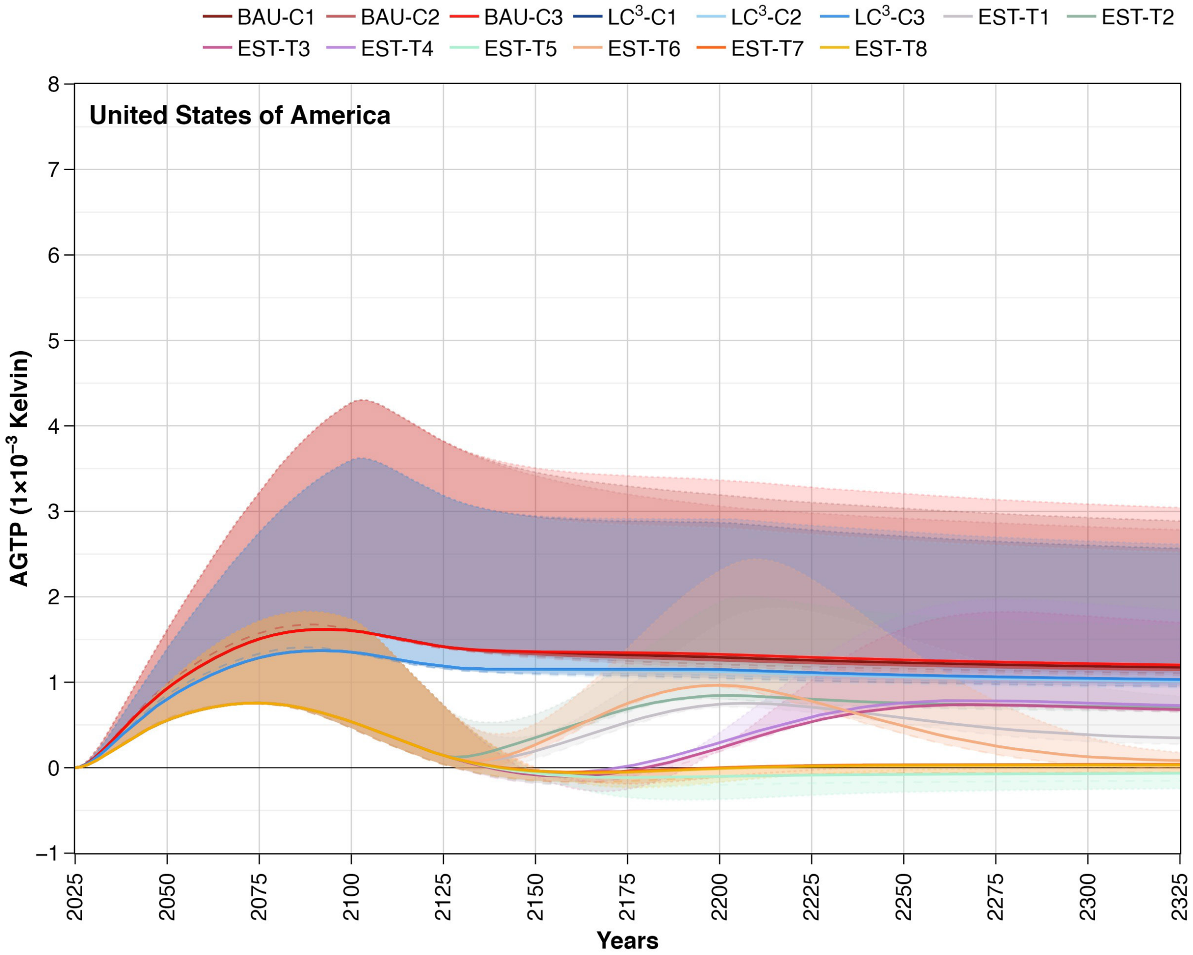

Supplementary Fig. S30 | Absolute global temperature potential (AGTP) of future cities for the United States of America between 2025 and 2325 based on start-of-life and end-of-life urban building scenarios. For each scenario, the solid line (SSP2), the dashed line (SSP1), and the dotted line (SSP5) represent shared socioeconomic pathways (SSPs), with the shaded area between these lines quantifying uncertainty. Start-of-life scenarios for future urban buildings cover the construction period between 2025 and 2100: BAU, reinforced OPC concrete cities; LC^3, reinforced LC^3 concrete cities; EST, timber cities. End-of-life scenarios for future urban buildings cover the demolition period between 2125 and 2200: C1, concrete is partly recycled, and partly discarded to landfills; C2, concrete is recycled; C3, concrete is discarded to landfills; T1, engineered timber is partly recycled into lower-quality products, partly incinerated, and partly discarded to landfills; T2, engineered timber is incinerated for bioenergy; T3, engineered timber is reused as functionally equivalent products, and this removes the incentive for forest regrowth; T4, engineered timber is recycled into lower quality and/or value products, and this removes the incentive for forest regrowth; T5, engineered timber is partly reused, partly recycled into lower quality/value products, and forestry and land management practices are applied to sustain the forest regrowth; T6, engineered timber is discarded to landfills with direct landfill gases (LFGs) release; T7, engineered timber is discarded to landfills, and LFGs are treated by enclosed flare; T8, engineered timber is discarded to landfills, and LFGs are captured and burned for energy production.

S1.3.5.2. Global warming potential

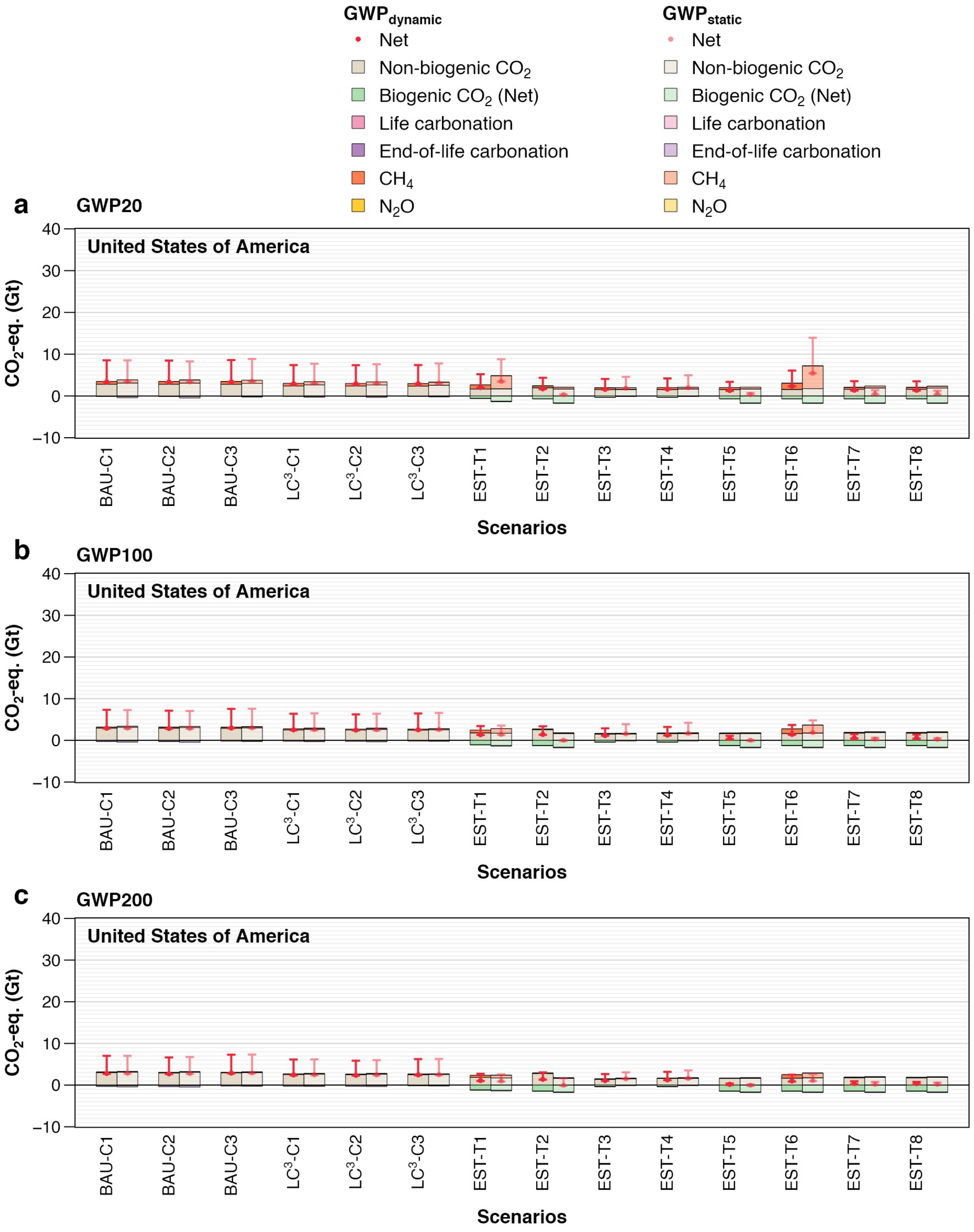

Supplementary Fig. S31 | Dynamic and static global warming potential (GWP) results of future cities for the United States of America based on start-of-life and end-of-life urban building scenarios for time horizon impacts of 20, 100 and 200 years. a GWP20$_{dynamic}$ and GWP20$_{static}$ results, **b** GWP100$_{dynamic}$ and GWP100$_{static}$ results, **c** GWP200$_{dynamic}$ and GWP200$_{static}$ results. Uncertainties are based on shared socioeconomic pathways (SSPs). The contribution analysis (i.e., heights of bars for each variable), red dots and texts for each scenario represent SSP2. Red error bars quantify uncertainty based on SSP1 and SSP5. Start-of-life scenarios for future urban buildings cover the construction period between 2025 and 2100: BAU, reinforced OPC concrete cities; LC^3,

reinforced LC^3 concrete cities; EST, timber cities. End-of-life scenarios for future urban buildings cover the demolition period between 2125 and 2200: C1, concrete is partly recycled, and partly discarded to landfills; C2, concrete is recycled; C3, concrete is discarded to landfills; T1, engineered timber is partly recycled into lower-quality products, partly incinerated, and partly discarded to landfills; T2, engineered timber is incinerated for bioenergy; T3, engineered timber is reused as functionally equivalent products, and this removes the incentive for forest regrowth; T4, engineered timber is recycled into lower quality and/or value products, and this removes the incentive for forest regrowth; T5, engineered timber is partly reused, partly recycled into lower quality/value products, and forestry and land management practices are applied to sustain the forest regrowth; T6, engineered timber is discarded to landfills with direct landfill gases (LFGs) release; T7, engineered timber is discarded to landfills, and LFGs are treated by enclosed flare; T8, engineered timber is discarded to landfills, and LFGs are captured and burned for energy production.

S1.3.6. Pakistan

S1.3.6.1. Absolute global temperature potential

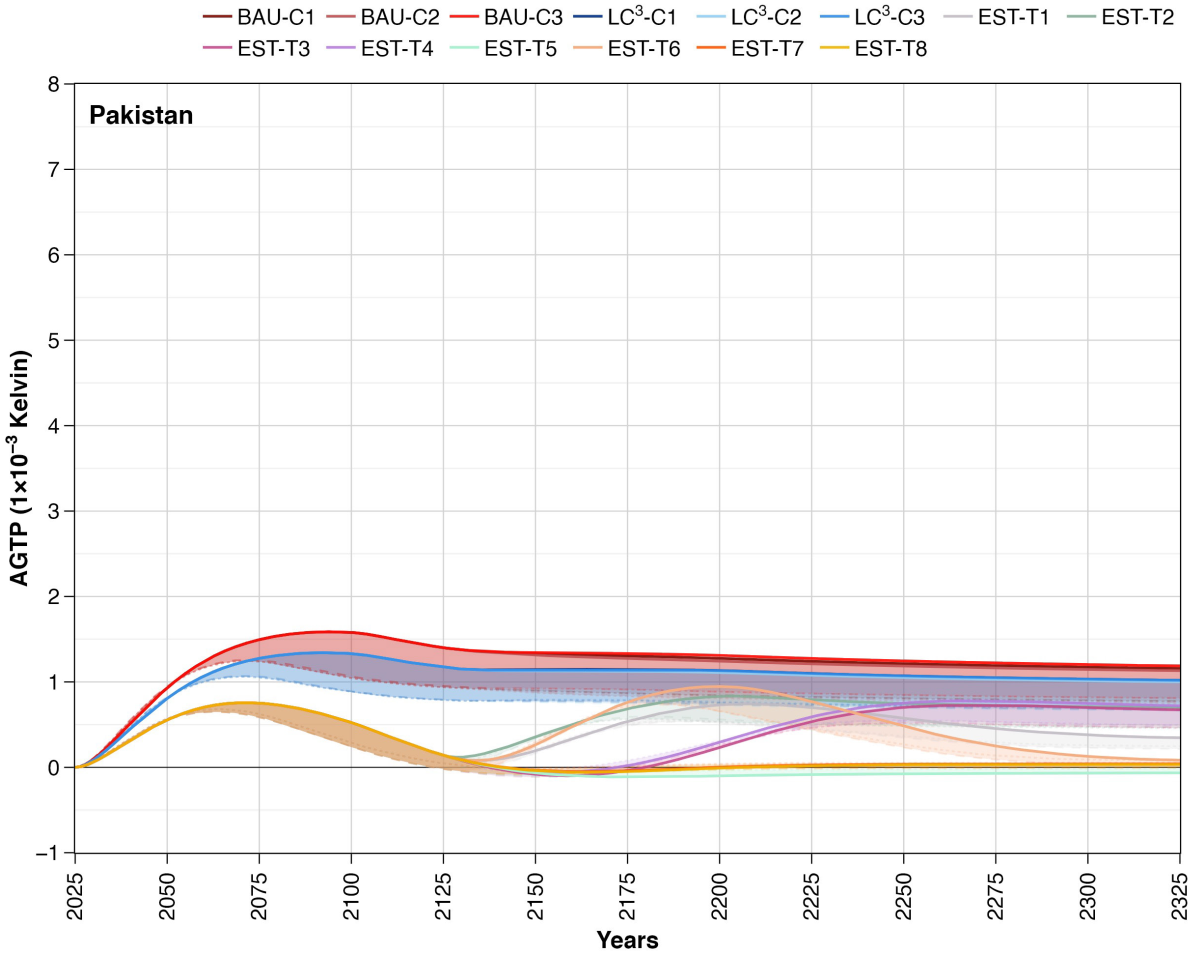

Supplementary Fig. S32 | Absolute global temperature potential (AGTP) of future cities for Pakistan between 2025 and 2325 based on start-of-life and end-of-life urban building scenarios. For each scenario, the solid line (SSP2), the dashed line (SSP1), and the dotted line (SSP5) represent shared socioeconomic pathways (SSPs), with the shaded area between these lines quantifying uncertainty. Start-of-life scenarios for future urban buildings cover the construction period between 2025 and 2100: BAU, reinforced OPC concrete cities; LC^3, reinforced LC^3 concrete cities; EST, timber cities. End-of-life scenarios for future urban buildings cover the demolition period between 2125 and 2200: C1, concrete is partly recycled, and partly discarded to landfills; C2, concrete is recycled; C3, concrete is discarded to landfills; T1, engineered timber is partly recycled into lower-quality products, partly incinerated, and partly discarded to landfills; T2, engineered timber is incinerated for bioenergy; T3, engineered timber is reused as functionally equivalent products, and this removes the incentive for forest regrowth; T4, engineered timber is recycled into lower quality and/or value products, and this removes the incentive for forest regrowth; T5, engineered timber is partly reused, partly recycled into lower quality/value products, and forestry and land management practices are applied to sustain the forest regrowth; T6, engineered timber is discarded to landfills with direct landfill gases (LFGs) release; T7, engineered timber is discarded to landfills, and LFGs are treated by enclosed flare; T8, engineered timber is discarded to landfills, and LFGs are captured and burned for energy production.

S1.3.6.2. Global warming potential

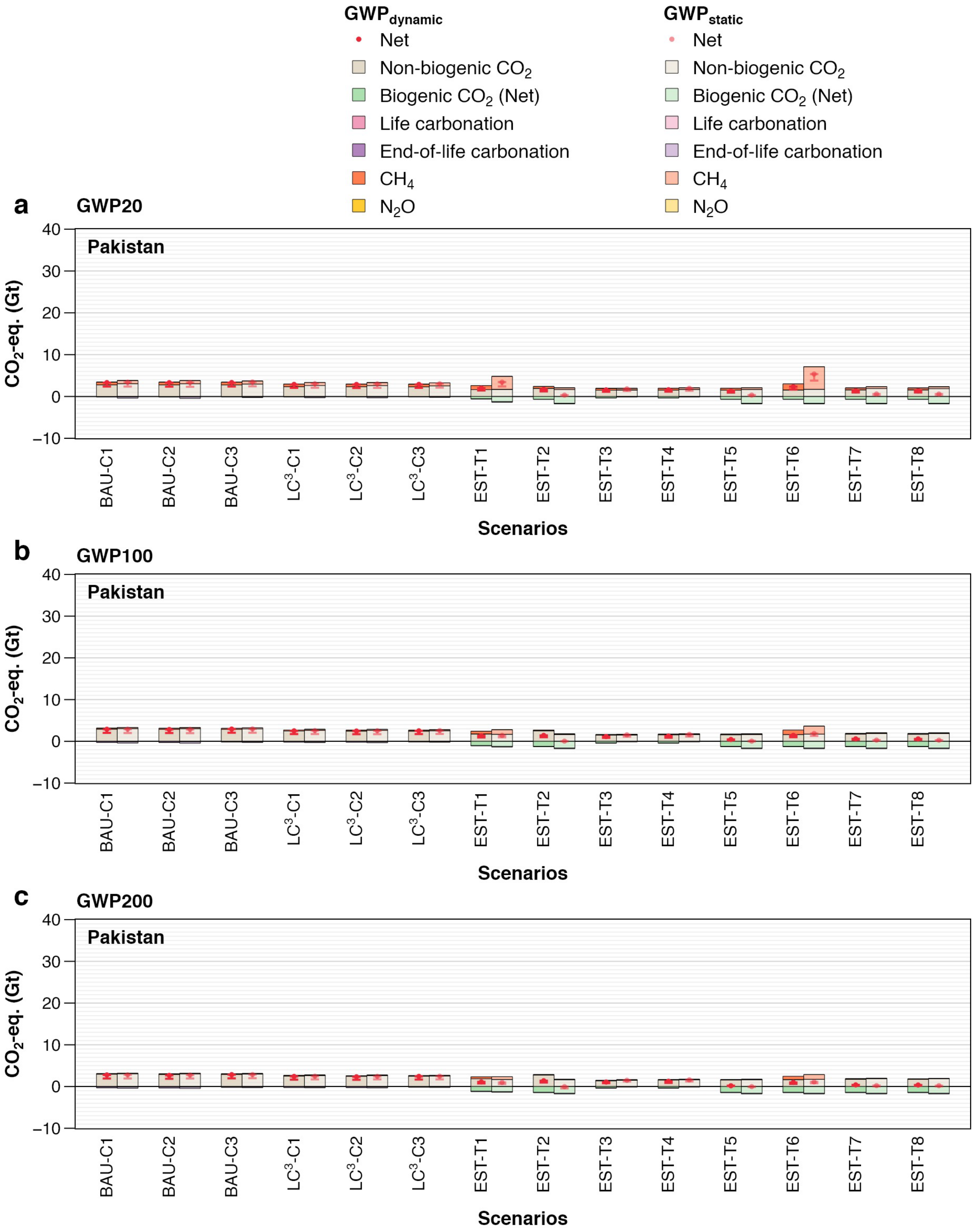

Supplementary Fig. S33 | Dynamic and static global warming potential (GWP) results of future cities for Pakistan based on start-of-life and end-of-life urban building scenarios for time horizon impacts of 20, 100 and 200 years. a $GWP20_{dynamic}$ and $GWP20_{static}$ results, **b** $GWP100_{dynamic}$ and $GWP100_{static}$ results, **c** $GWP200_{dynamic}$ and $GWP200_{static}$ results. Uncertainties are based on shared socioeconomic pathways (SSPs). The contribution analysis (i.e., heights of bars for each variable), red dots and texts for each scenario represent SSP2. Red error bars quantify uncertainty based on SSP1 and SSP5. Start-of-life scenarios for future urban buildings cover the construction period between 2025 and 2100: BAU, reinforced OPC concrete cities; LC^3, reinforced LC^3 concrete

cities; EST, timber cities. End-of-life scenarios for future urban buildings cover the demolition period between 2125 and 2200: C1, concrete is partly recycled, and partly discarded to landfills; C2, concrete is recycled; C3, concrete is discarded to landfills; T1, engineered timber is partly recycled into lower-quality products, partly incinerated, and partly discarded to landfills; T2, engineered timber is incinerated for bioenergy; T3, engineered timber is reused as functionally equivalent products, and this removes the incentive for forest regrowth; T4, engineered timber is recycled into lower quality and/or value products, and this removes the incentive for forest regrowth; T5, engineered timber is partly reused, partly recycled into lower quality/value products, and forestry and land management practices are applied to sustain the forest regrowth; T6, engineered timber is discarded to landfills with direct landfill gases (LFGs) release; T7, engineered timber is discarded to landfills, and LFGs are treated by enclosed flare; T8, engineered timber is discarded to landfills, and LFGs are captured and burned for energy production.

S1.3.7. Democratic Republic of the Congo

S1.3.7.1. Absolute global temperature potential

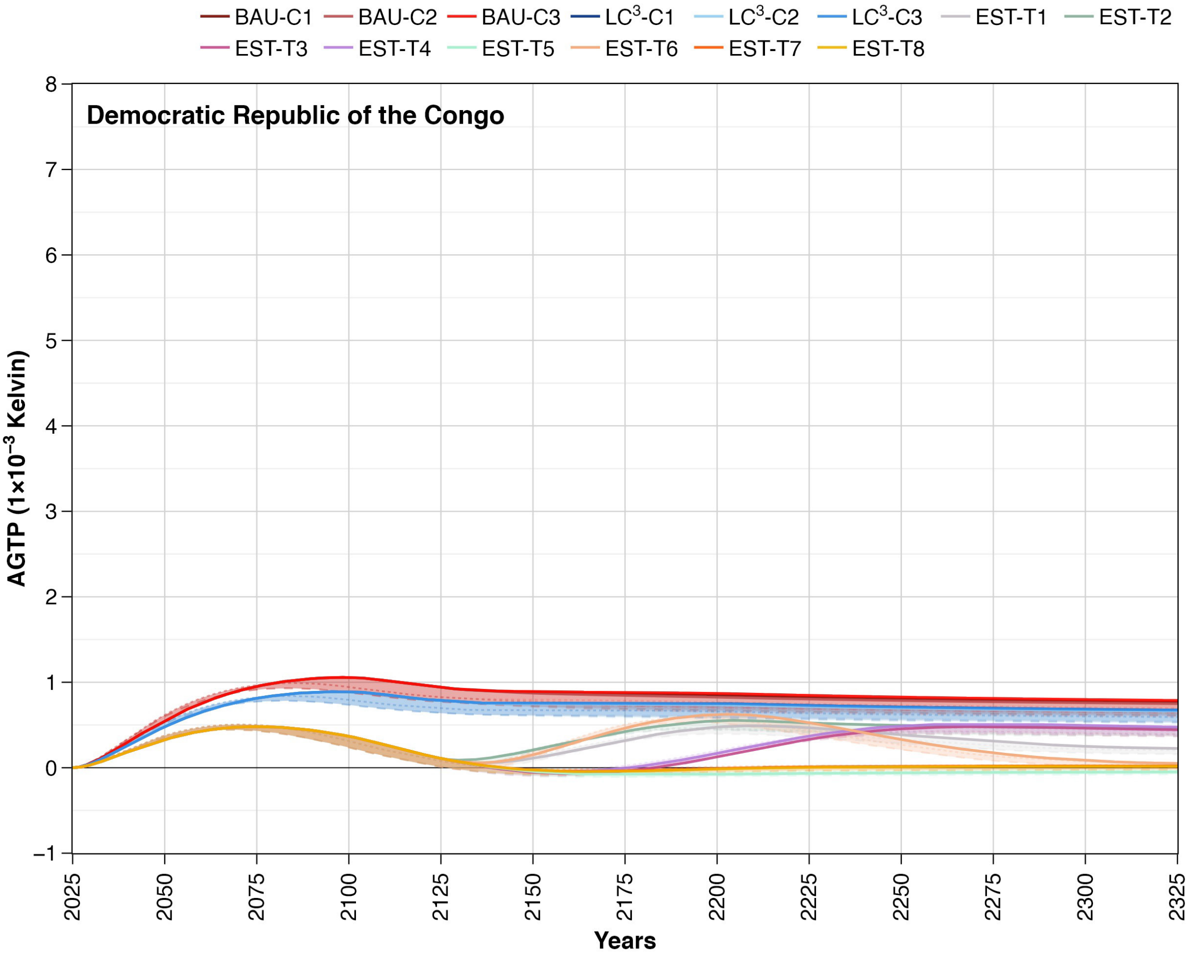

Supplementary Fig. S34 | Absolute global temperature potential (AGTP) of future cities for the Democratic Republic of the Congo between 2025 and 2325 based on start-of-life and end-of-life urban building scenarios. For each scenario, the solid line (SSP2), the dashed line (SSP1), and the dotted line (SSP5) represent shared socioeconomic pathways (SSPs), with the shaded area between these lines quantifying uncertainty. Start-of-life scenarios for future urban buildings cover the construction period between 2025 and 2100: BAU, reinforced OPC concrete cities; LC^3, reinforced LC^3 concrete cities; EST, timber cities. End-of-life scenarios for future urban buildings cover the demolition period between 2125 and 2200: C1, concrete is partly recycled, and partly discarded to landfills; C2, concrete is recycled; C3, concrete is discarded to landfills; T1, engineered timber is partly recycled into lower-quality products, partly incinerated, and partly discarded to landfills; T2, engineered timber is incinerated for bioenergy; T3, engineered timber is reused as functionally equivalent products, and this removes the incentive for forest regrowth; T4, engineered timber is recycled into lower quality and/or value products, and this removes the incentive for forest regrowth; T5, engineered timber is partly reused, partly recycled into lower quality/value products, and forestry and land management practices are applied to sustain the forest regrowth; T6, engineered timber is discarded to landfills with direct landfill gases (LFGs) release; T7, engineered timber is discarded to landfills, and LFGs are treated by enclosed flare; T8, engineered timber is discarded to landfills, and LFGs are captured and burned for energy production.

S1.3.7.2. Global warming potential

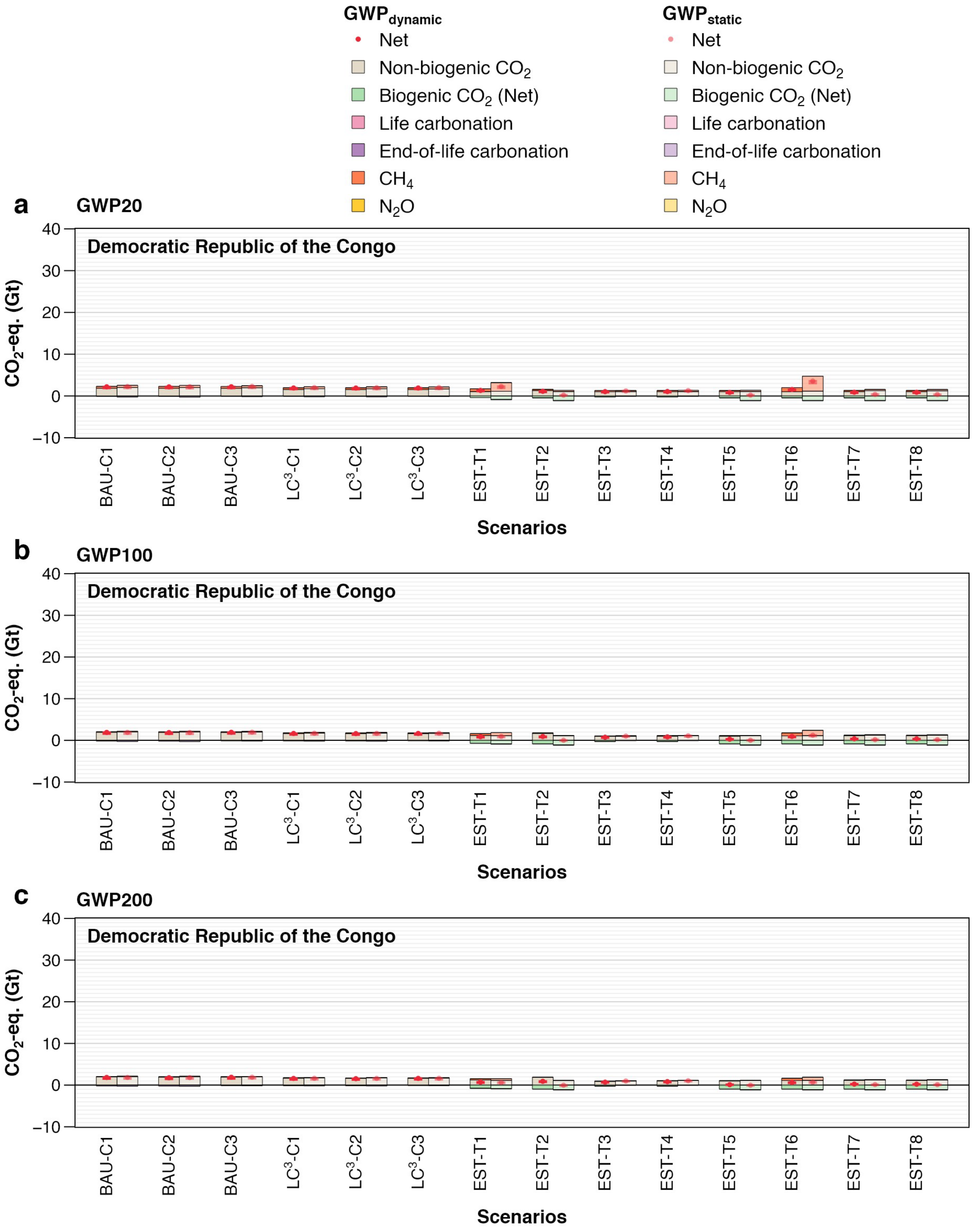

Supplementary Fig. S35 | Dynamic and static global warming potential (GWP) results of future cities for the Democratic Republic of the Congo based on start-of-life and end-of-life urban building scenarios for time horizon impacts of 20, 100 and 200 years. a $GWP20_{dynamic}$ and $GWP20_{static}$ results, **b** $GWP100_{dynamic}$ and $GWP100_{static}$ results, **c** $GWP200_{dynamic}$ and $GWP200_{static}$ results. Uncertainties are based on shared socioeconomic pathways (SSPs). The contribution analysis (i.e., heights of bars for each variable), red dots and texts for each scenario represent SSP2. Red error bars quantify uncertainty based on SSP1 and SSP5. Start-of-life scenarios for future urban buildings cover the construction period between 2025 and 2100: BAU, reinforced OPC concrete cities;

LC^3, reinforced LC^3 concrete cities; EST, timber cities. End-of-life scenarios for future urban buildings cover the demolition period between 2125 and 2200: C1, concrete is partly recycled, and partly discarded to landfills; C2, concrete is recycled; C3, concrete is discarded to landfills; T1, engineered timber is partly recycled into lower-quality products, partly incinerated, and partly discarded to landfills; T2, engineered timber is incinerated for bioenergy; T3, engineered timber is reused as functionally equivalent products, and this removes the incentive for forest regrowth; T4, engineered timber is recycled into lower quality and/or value products, and this removes the incentive for forest regrowth; T5, engineered timber is partly reused, partly recycled into lower quality/value products, and forestry and land management practices are applied to sustain the forest regrowth; T6, engineered timber is discarded to landfills with direct landfill gases (LFGs) release; T7, engineered timber is discarded to landfills, and LFGs are treated by enclosed flare; T8, engineered timber is discarded to landfills, and LFGs are captured and burned for energy production.

S1.3.8. China

S1.3.8.1. Absolute global temperature potential

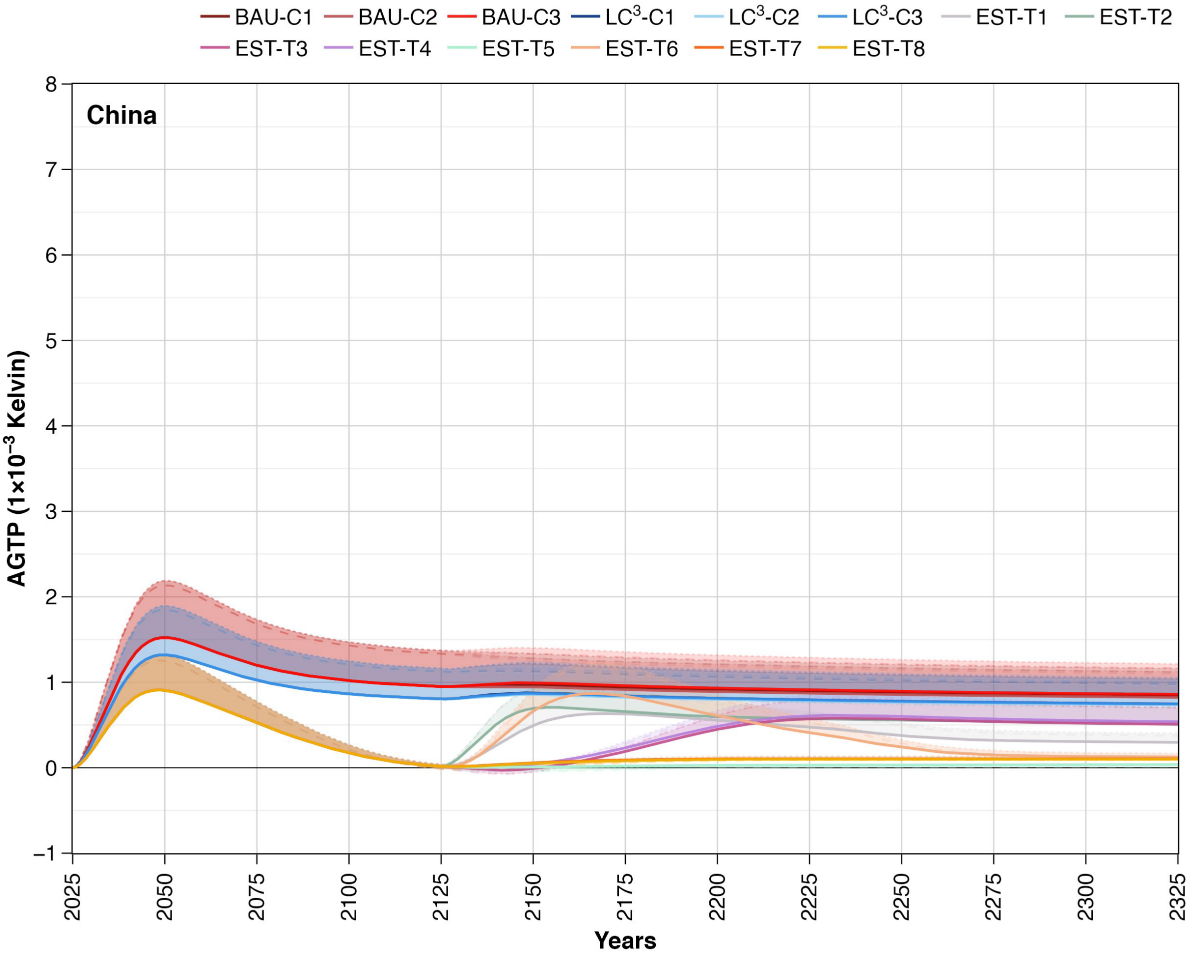

Supplementary Fig. S36 | Absolute global temperature potential (AGTP) of future cities for China between 2025 and 2325 based on start-of-life and end-of-life urban building scenarios. For each scenario, the solid line (SSP2), the dashed line (SSP1), and the dotted line (SSP5) represent shared socioeconomic pathways (SSPs), with the shaded area between these lines quantifying uncertainty. Start-of-life scenarios for future urban buildings cover the construction period between 2025 and 2100: BAU, reinforced OPC concrete cities; LC^3, reinforced LC^3 concrete cities; EST, timber cities. End-of-life scenarios for future urban buildings cover the demolition period between 2125 and 2200: C1, concrete is partly recycled, and partly discarded to landfills; C2, concrete is recycled; C3, concrete is discarded to landfills; T1, engineered timber is partly recycled into lower-quality products, partly incinerated, and partly discarded to landfills; T2, engineered timber is incinerated for bioenergy; T3, engineered timber is reused as functionally equivalent products, and this removes the incentive for forest regrowth; T4, engineered timber is recycled into lower quality and/or value products, and this removes the incentive for forest regrowth; T5, engineered timber is partly reused, partly recycled into lower quality/value products, and forestry and land management practices are applied to sustain the forest regrowth; T6, engineered timber is discarded to landfills with direct landfill gases (LFGs) release; T7, engineered timber is discarded to landfills, and LFGs are treated by enclosed flare; T8, engineered timber is discarded to landfills, and LFGs are captured and burned for energy production.

S1.3.8.2. Global warming potential

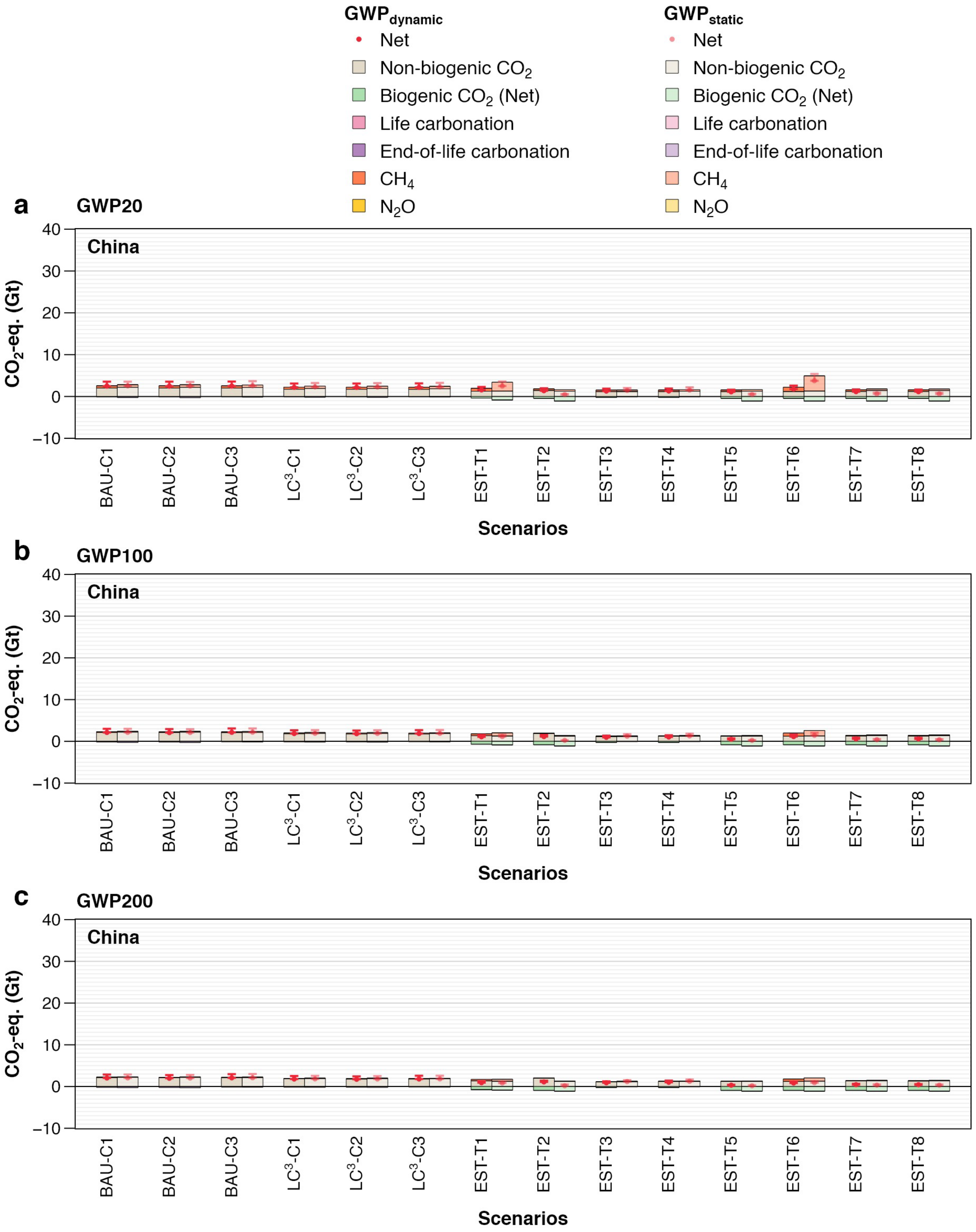

Supplementary Fig. S37 | Dynamic and static global warming potential (GWP) results of future cities for China based on start-of-life and end-of-life urban building scenarios for time horizon impacts of 20, 100 and 200 years. a $GWP20_{dynamic}$ and $GWP20_{static}$ results, **b** $GWP100_{dynamic}$ and $GWP100_{static}$ results, **c** $GWP200_{dynamic}$ and $GWP200_{static}$ results. Uncertainties are based on shared socioeconomic pathways (SSPs). The contribution analysis (i.e., heights of bars for each variable), red dots and texts for each scenario represent SSP2. Red error bars quantify uncertainty based on SSP1 and SSP5. Start-of-life scenarios for future urban buildings cover the construction period between 2025 and 2100: BAU, reinforced OPC concrete cities; LC^3, reinforced LC^3 concrete cities;

EST, timber cities. End-of-life scenarios for future urban buildings cover the demolition period between 2125 and 2200: C1, concrete is partly recycled, and partly discarded to landfills; C2, concrete is recycled; C3, concrete is discarded to landfills; T1, engineered timber is partly recycled into lower-quality products, partly incinerated, and partly discarded to landfills; T2, engineered timber is incinerated for bioenergy; T3, engineered timber is reused as functionally equivalent products, and this removes the incentive for forest regrowth; T4, engineered timber is recycled into lower quality and/or value products, and this removes the incentive for forest regrowth; T5, engineered timber is partly reused, partly recycled into lower quality/value products, and forestry and land management practices are applied to sustain the forest regrowth; T6, engineered timber is discarded to landfills with direct landfill gases (LFGs) release; T7, engineered timber is discarded to landfills, and LFGs are treated by enclosed flare; T8, engineered timber is discarded to landfills, and LFGs are captured and burned for energy production.

S1.3.9. Ethiopia

S1.3.9.1. Absolute global temperature potential

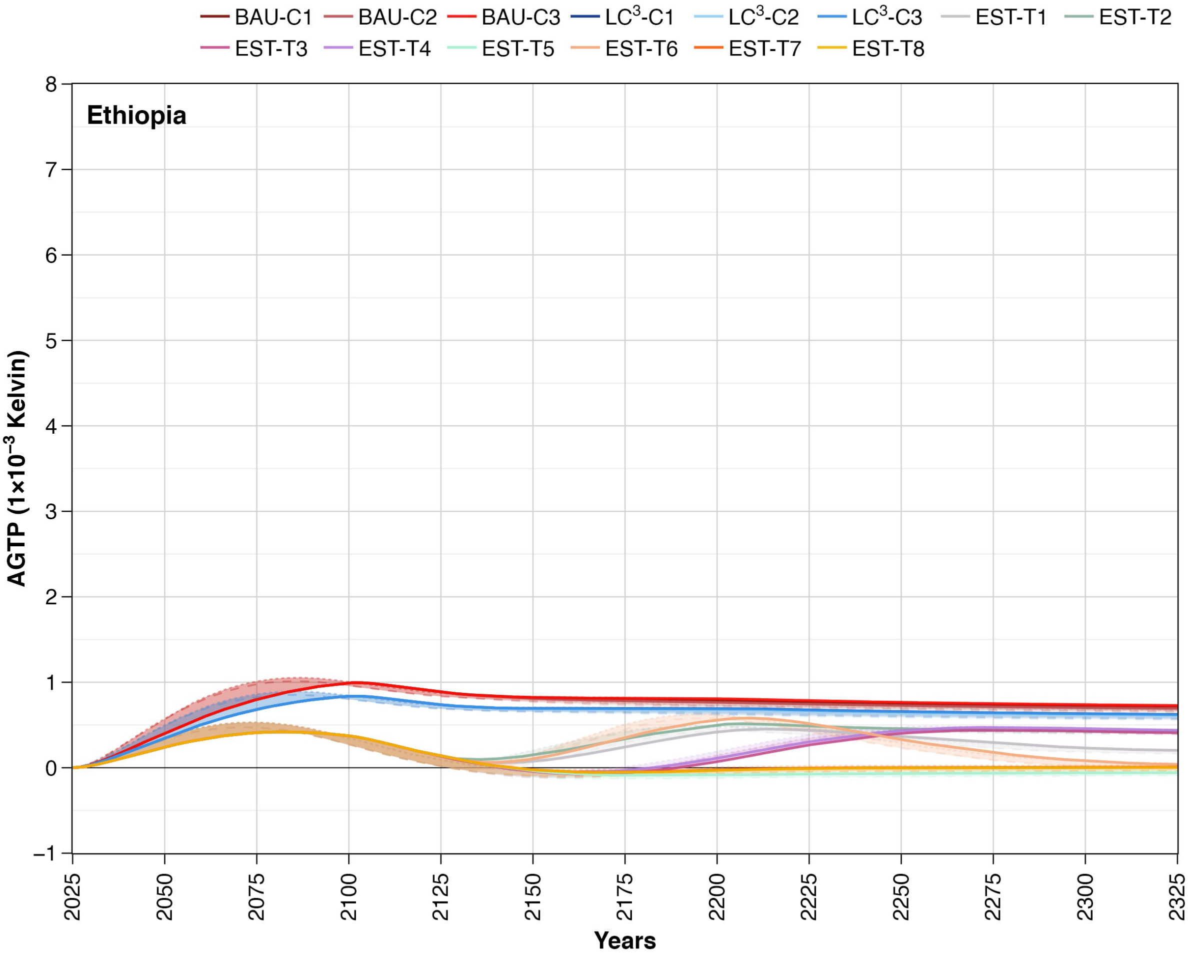

Supplementary Fig. S38 | Absolute global temperature potential (AGTP) of future cities for Ethiopia between 2025 and 2325 based on start-of-life and end-of-life urban building scenarios. For each scenario, the solid line (SSP2), the dashed line (SSP1), and the dotted line (SSP5) represent shared socioeconomic pathways (SSPs), with the shaded area between these lines quantifying uncertainty. Start-of-life scenarios for future urban buildings cover the construction period between 2025 and 2100: BAU, reinforced OPC concrete cities; LC^3, reinforced LC^3 concrete cities; EST, timber cities. End-of-life scenarios for future urban buildings cover the demolition period between 2125 and 2200: C1, concrete is partly recycled, and partly discarded to landfills; C2, concrete is recycled; C3, concrete is discarded to landfills; T1, engineered timber is partly recycled into lower-quality products, partly incinerated, and partly discarded to landfills; T2, engineered timber is incinerated for bioenergy; T3, engineered timber is reused as functionally equivalent products, and this removes the incentive for forest regrowth; T4, engineered timber is recycled into lower quality and/or value products, and this removes the incentive for forest regrowth; T5, engineered timber is partly reused, partly recycled into lower quality/value products, and forestry and land management practices are applied to sustain the forest regrowth; T6, engineered timber is discarded to landfills with direct landfill gases (LFGs) release; T7, engineered timber is discarded to landfills, and LFGs are treated by enclosed flare; T8, engineered timber is discarded to landfills, and LFGs are captured and burned for energy production.

S1.3.9.2. Global warming potential

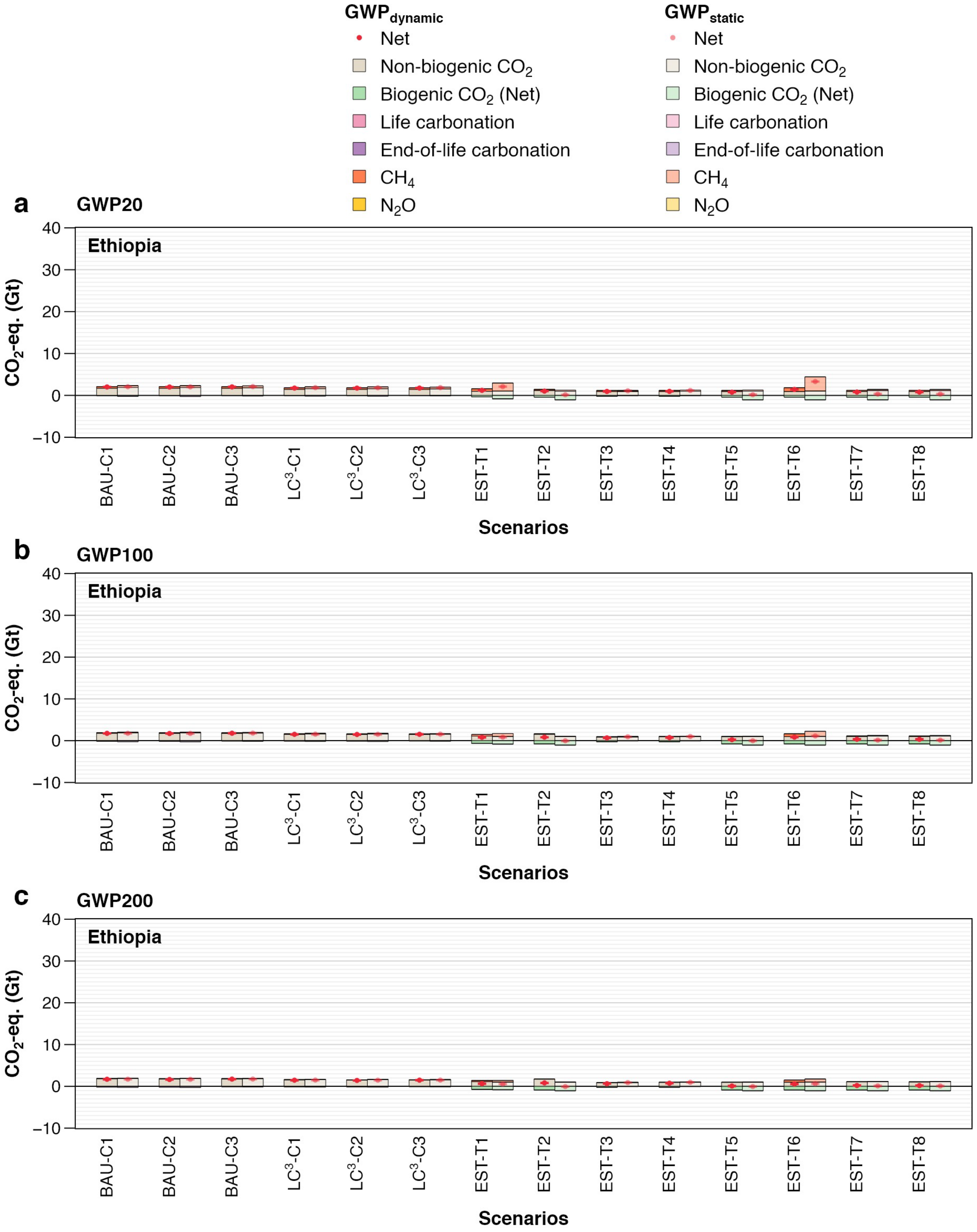

Supplementary Fig. S39 | Dynamic and static global warming potential (GWP) results of future cities for Ethiopia based on start-of-life and end-of-life urban building scenarios for time horizon impacts of 20, 100 and 200 years. a $GWP20_{dynamic}$ and $GWP20_{static}$ results, **b** $GWP100_{dynamic}$ and $GWP100_{static}$ results, **c** $GWP200_{dynamic}$ and $GWP200_{static}$ results. Uncertainties are based on shared socioeconomic pathways (SSPs). The contribution analysis (i.e., heights of bars for each variable), red dots and texts for each scenario represent SSP2. Red error bars quantify uncertainty based on SSP1 and SSP5. Start-of-life scenarios for future urban buildings cover the construction period between 2025 and 2100: BAU, reinforced OPC concrete cities; LC^3, reinforced LC^3 concrete

cities; EST, timber cities. End-of-life scenarios for future urban buildings cover the demolition period between 2125 and 2200: C1, concrete is partly recycled, and partly discarded to landfills; C2, concrete is recycled; C3, concrete is discarded to landfills; T1, engineered timber is partly recycled into lower-quality products, partly incinerated, and partly discarded to landfills; T2, engineered timber is incinerated for bioenergy; T3, engineered timber is reused as functionally equivalent products, and this removes the incentive for forest regrowth; T4, engineered timber is recycled into lower quality and/or value products, and this removes the incentive for forest regrowth; T5, engineered timber is partly reused, partly recycled into lower quality/value products, and forestry and land management practices are applied to sustain the forest regrowth; T6, engineered timber is discarded to landfills with direct landfill gases (LFGs) release; T7, engineered timber is discarded to landfills, and LFGs are treated by enclosed flare; T8, engineered timber is discarded to landfills, and LFGs are captured and burned for energy production.

S1.4. Building level

S1.4.1. Atmospheric greenhouse gas concentration changes

Biogenic sinks from forest regrowth in engineered timber buildings provide a considerably larger CO_2 sink than the carbonation sink in reinforced concrete buildings (Supplementary Fig. S40). Two factors can cause rapid changes in these sinks at the end of the building lifespan. (1) Incineration causes an instantaneous biogenic emission, and (2) crushing of concrete provides an instantaneous CO_2 uptake through carbonation due to the exposure of larger surface areas (Supplementary Fig. S40a,b,c,d).

BAU and LC^3 start-of-life scenarios show similar carbonation dynamics, but LC^3 has a lower carbonation capacity than Portland cement. LC^3 carbonates relatively faster during building life and does not carbonate as much as Portland cement after demolition (Supplementary Fig. S40a,b). When carbonation peaks and stops after year 100, the sink is shown to decay as an emission would (Supplementary Fig. S40a,b). This allows for a fair comparison of emissions and sinks and is consistent with the model by Joos et al.[1]. If left to carbonate longer, the concrete would rapidly reach saturation and carbonation would stop. This would be quicker for LC^3 which carbonates faster and has a reduced capacity to bind CO_2.

Atmospheric GHG concentration changes caused by engineered timber buildings are highly variable depending on the end-of-life utilisation of the timber. Forest regrowth provides continuous CO_2 storage except for the modelled incineration scenario (T2), where the accumulated and stored CO_2 is emitted instantaneously into the atmosphere after 100 years (Supplementary Fig. S40c). In that same scenario, the energy from incineration fossil fuel use which results in a simultaneous negative end-of-life substitution credit. However, when the timber is recovered in EST-T3, and EST-T4 scenarios, a large positive end-of-life substitution biogenic credit occurs due to a lack of new tree growth (Supplementary Fig. S40d).

End-of-life utilisation of concrete has a much lower GHG impact than engineered timber. Recycling concrete almost does not change atmospheric concentrations (Supplementary Fig. S40a). This is because recycled concrete is modelled to replace gravel, which has insignificant associated emissions.

In the case of timber landfilling, the type of landfill significantly affects atmospheric concentrations of CH_4. In the EST-T6 scenario, timber decomposition leads to a peak in atmospheric CH_4 ~20 years after demolition (Supplementary Fig. S41a). However, in the EST-T8 scenario, we observe no CH_4 release since landfill gases are captured and used for energy production (Supplementary Fig. S41b).

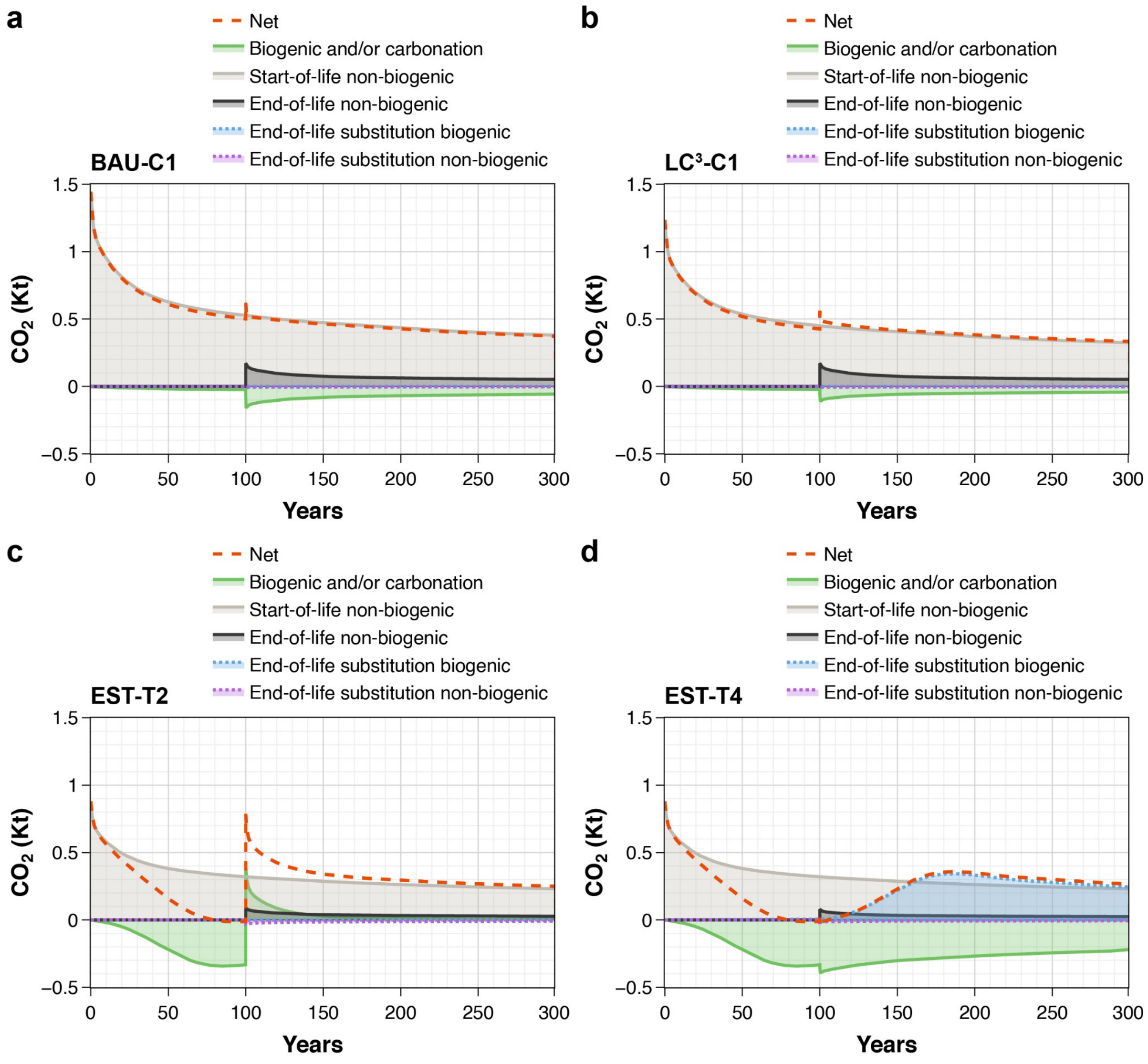

Supplementary Fig. S40 | Atmospheric CO_2 concentrations over time based on start-of-life and end-of-life scenarios of the case study building constructed in 2025. a BAU-C1 scenario, **b** LC^3-C1 scenario, **c** EST-T2 scenario, **d** EST-T4 scenario. The case study building has a 1,970 m^2 gross internal area with four storeys, and a lifespan of 100 years. The results are shown for SSP2. BAU, reinforced OPC concrete building; LC^3, reinforced LC^3 concrete building; EST, engineered structural timber building; C1, concrete is partly recycled, and partly discarded to landfills; T2, engineered timber is incinerated for bioenergy; T4, engineered timber is recycled into lower quality and/or value products, and this removes the incentive for forest regrowth.

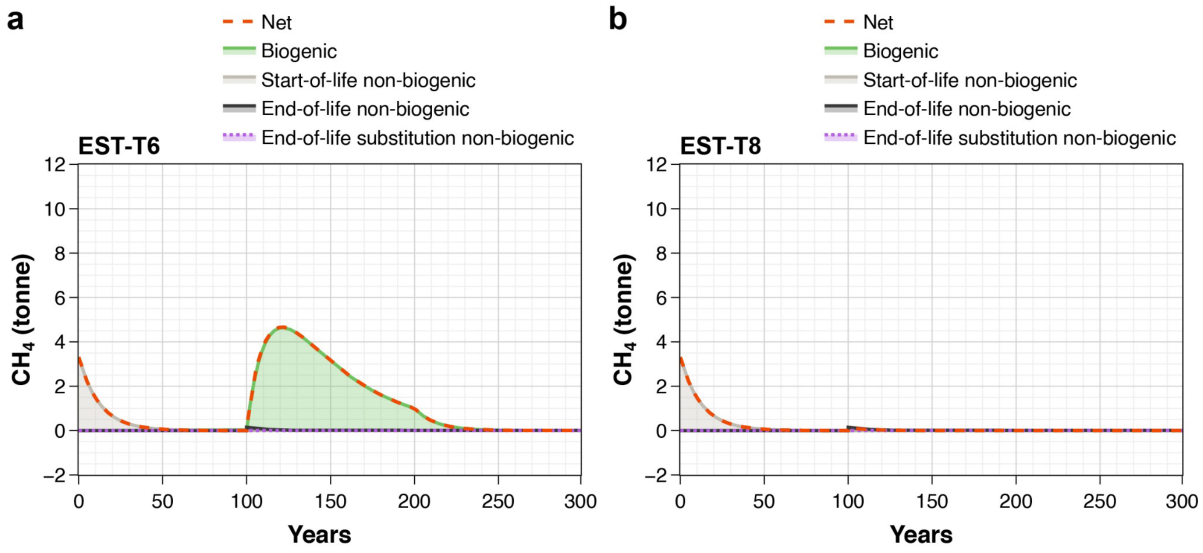

Supplementary Fig. S41 | Atmospheric CH_4 concentrations over time based on start-of-life and end-of-life scenarios of the case study building. a EST-T6 scenario, **b** EST-T8 scenario. The case study building has a 1,970 m^2 gross internal area with four storeys, and a lifespan of 100 years. The results are shown for SSP2. EST, engineered structural timber building; T6, engineered timber is discarded to landfills with direct landfill gases (LFGs) release; T8, engineered timber is discarded to landfills, and LFGs are captured and burned for energy production.

S1.4.2. Absolute global temperature potential

AGTP results are driven by three main parameters. The initial peak is associated with the magnitude of upfront emissions, assumed instantaneous. Their gaseous composition affects the delay of this peak which is related to the inertia of the climatic system, occurring 15 and 18 years after construction for engineered timber and concrete buildings, respectively (Supplementary Fig. S42). It is followed by a natural decay amplified by the sinks associated with each material. Finally, decisions taken at the end of the life stage significantly affect the impacts on the AGTP.

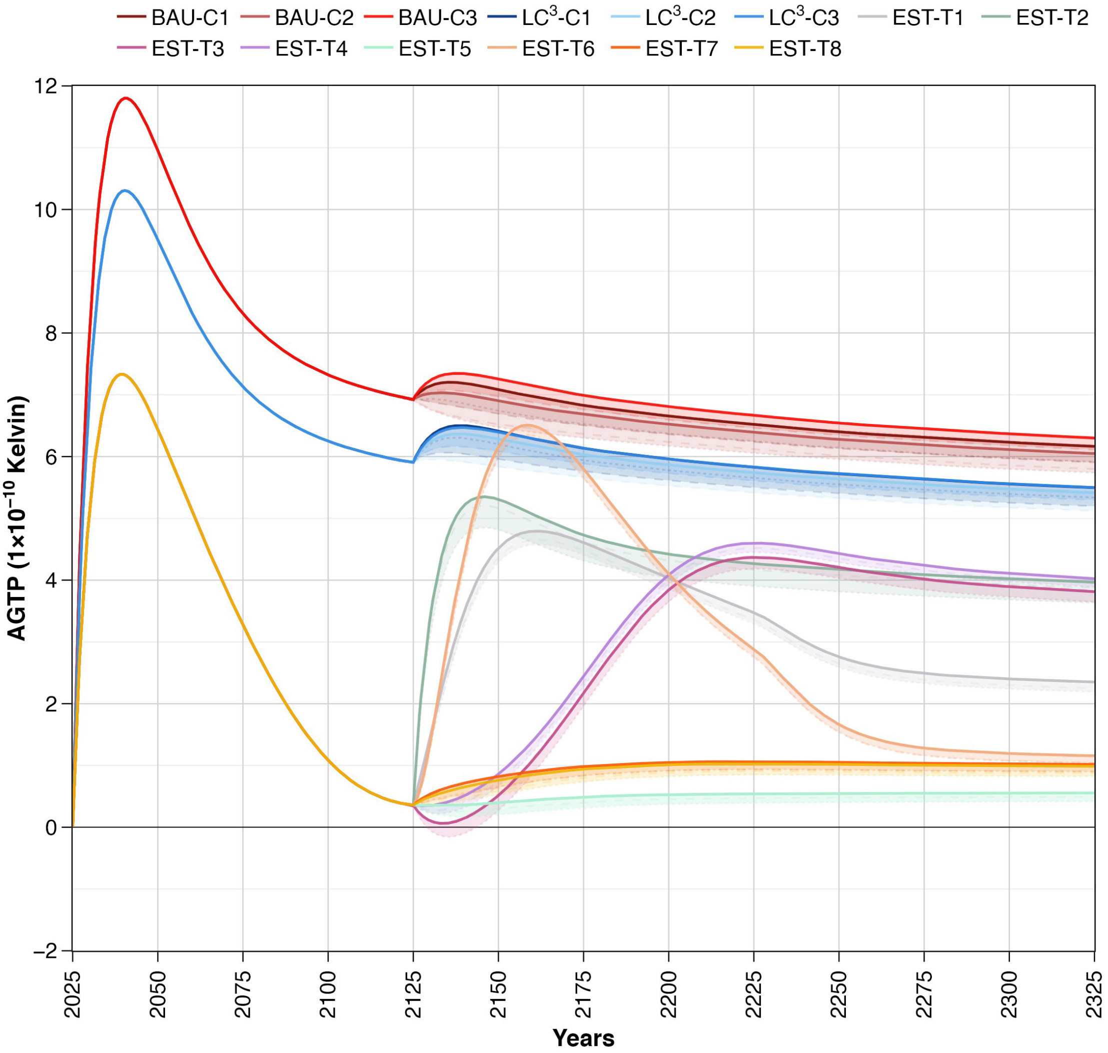

Supplementary Fig. S42 | Absolute global temperature potential (AGTP) of the case study building constructed in 2025 based on start-of-life and end-of-life urban building scenarios. The case study building has a 1,970 m^2 gross internal area with four storeys, and a lifespan of 100 years. For each scenario, the solid line (SSP2), the dashed line (SSP1), and the dotted line (SSP5) represent shared socioeconomic pathways (SSPs), with the shaded area between these lines quantifying uncertainty. BAU, reinforced OPC concrete building; LC^3, reinforced LC^3 concrete building; EST, engineered structural timber building; C1, concrete is partly recycled, and partly discarded to landfills; C2, concrete is recycled; C3, concrete is discarded to landfills; T1, engineered timber is partly recycled into lower-quality products, partly incinerated, and partly discarded to landfills; T2, engineered timber is incinerated for bioenergy; T3, engineered timber is reused as functionally equivalent products, and this removes the incentive for forest regrowth; T4, engineered timber is recycled into lower quality and/or value products, and this removes the incentive for forest regrowth; T5, engineered timber is partly reused, partly recycled into lower quality/value products, and forestry and land management practices are applied to sustain the forest regrowth; T6, engineered timber is discarded to landfills with direct landfill gases (LFGs) release; T7, engineered timber is discarded to

landfills, and LFGs are treated by enclosed flare; T8, engineered timber is discarded to landfills, and LFGs are captured and burned for energy production.

S1.4.3. Global warming potential

Our dynamic LCA results show that buildings with engineered timber structural frames can have up to a 3.7-fold decrease in $GWP100_{dynamic}$ compared to a building with a reinforced concrete structural frame: The EST-T5 scenario has the lowest dynamic GWPs (for $GWP100_{dynamic}$: 0.43 Kt CO_2-eq., 217.2 kg CO_2-eq./m^2), and the BAU-C3 scenario has the highest dynamic GWPs (for $GWP100_{dynamic}$: 1.60 Kt CO_2-eq., 811.3 kg CO_2-eq./m^2) among all scenarios (Supplementary Fig. S43b). Additionally, the EST-T5 scenario has a 3-fold decrease in $GWP100_{dynamic}$ compared to the LC^3-C2 (1.37 Kt CO_2-eq., 694.2 kg CO_2-eq./m^2) scenario which has the lowest value for LC^3 start-of-life scenarios (Supplementary Fig. S43b).

Our GWP100 results are consistent with existing literature[2-5]. However, providing a direct comparison of LCA results is challenging because of varying system boundaries, normalising methods (i.e., net floor area vs gross floor area), building designs, and geographical considerations[6,7]. Supplementary Table S1 presents the $GWP100_{dynamic}$ and $GWP100_{static}$ results per square meter of floor space for the case study building constructed in 2025 based on start-of-life and end-of-life urban building scenarios.

Supplementary Table S1 | $GWP100_{dynamic}$ and $GWP100_{static}$ results per square meter of floor space for the case study building constructed in 2025 based on start-of-life and end-of-life urban building scenarios. The case study building has a 1,970 m^2 gross internal area with four storeys, and a lifespan of 100 years. The results are shown for SSP2.

Scenarios	$GWP100_{dynamic}$ (kg CO_2-eq. per m^2 floor space)	$GWP100_{static}$ (kg CO_2-eq. per m^2 floor space)
BAU-C1	801.1	806.4
BAU-C2	786.8	792.7
BAU-C3	811.3	820.7
LC^3-C1	706.2	721.9
LC^3-C2	694.2	712.2
LC^3-C3	707.5	721.3
EST-T1	427.9	444.4
EST-T2	442.6	122.5
EST-T3	380.8	474.8
EST-T4	406.6	500.5
EST-T5	217.2	123.8
EST-T6	445.6	549.8
EST-T7	252.2	180.7
EST-T8	248.2	176.7

Abbreviations: GWP, global warming potential; CO_2-eq., carbon dioxide equivalents; kg, kilogram; m^2, square meter.
Scenarios: BAU, reinforced OPC concrete building; LC^3, reinforced LC^3 concrete building; EST, engineered structural timber building; C1, concrete is partly recycled, and partly discarded to landfills; C2, concrete is recycled; C3, concrete is discarded to landfills; T1, engineered timber is partly recycled into lower-quality products, partly incinerated, and partly discarded to landfills; T2, engineered timber is incinerated for bioenergy; T3, engineered timber is reused as functionally equivalent products, and this removes the incentive for forest regrowth; T4, engineered timber is recycled into lower quality and/or value products, and this removes the incentive for forest regrowth; T5, engineered timber is partly reused, partly recycled into lower quality/value products, and forestry and land management practices are applied to sustain the forest regrowth; T6, engineered timber is discarded to landfills with direct landfill gases (LFGs) release; T7, engineered timber is discarded to landfills, and LFGs are treated by enclosed flare; T8, engineered timber is discarded to landfills, and LFGs are captured and burned for energy production.

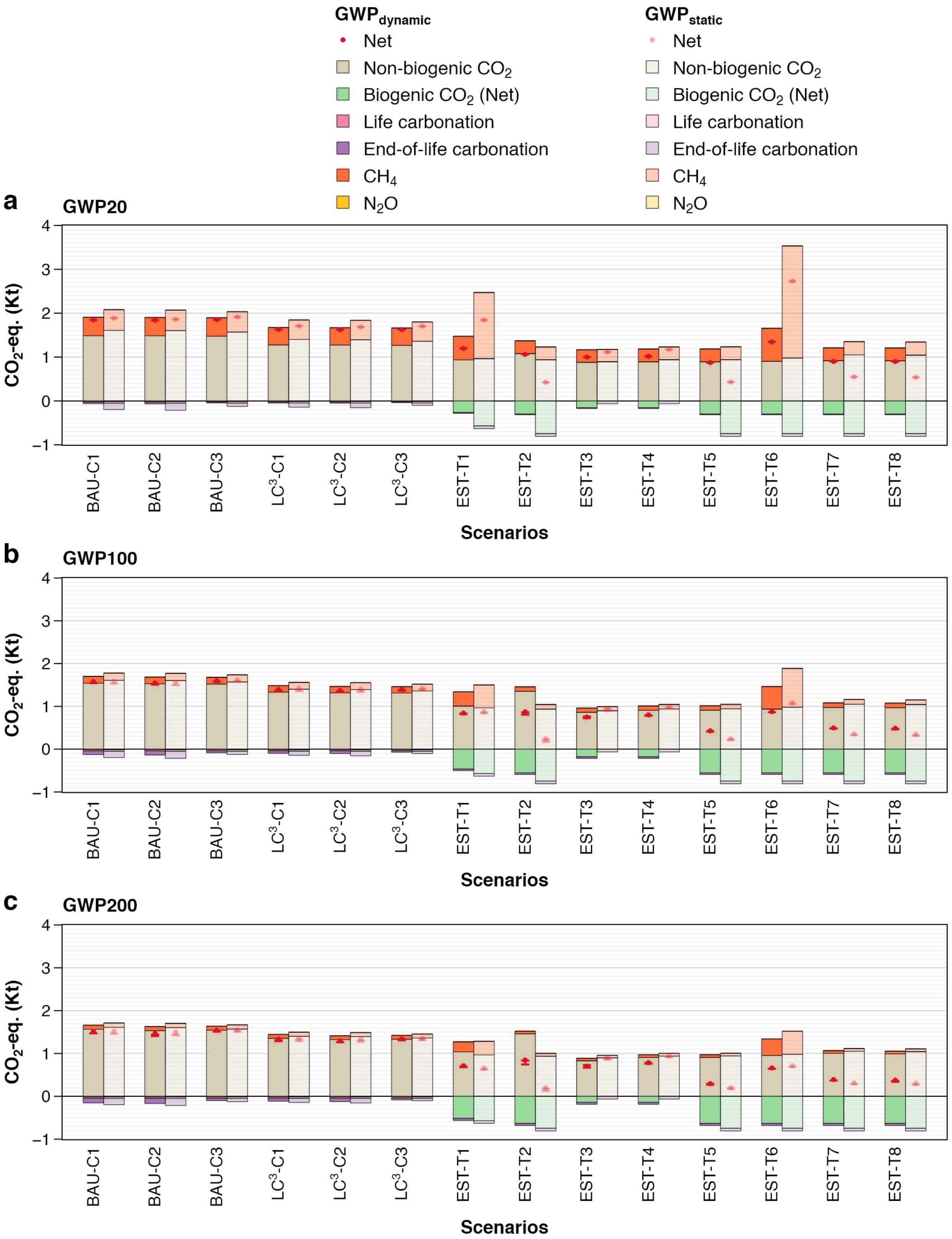

Supplementary Fig. S43 | Dynamic and static global warming potential (GWP) results of the case study building constructed in 2025 based on start-of-life and end-of-life urban building scenarios for time horizon impacts of 20, 100 and 200 years. a GWP20$_{dynamic}$ and GWP20$_{static}$ results, **b** GWP100$_{dynamic}$ and GWP100$_{static}$ results, **c** GWP200$_{dynamic}$ and GWP200$_{static}$ results. The case study building has a 1,970 m^2 gross internal area with four storeys, and a lifespan of 100 years. Uncertainties are based on shared socioeconomic pathways (SSPs). The contribution analysis (i.e., heights of bars for each variable), red dots and texts for each scenario represent SSP2. Red error bars quantify uncertainty based on SSP1 and SSP5. BAU, reinforced OPC concrete building; LC^3, reinforced LC^3 concrete building; EST, engineered structural timber building; C1, concrete is partly

recycled, and partly discarded to landfills; C2, concrete is recycled; C3, concrete is discarded to landfills; T1, engineered timber is partly recycled into lower-quality products, partly incinerated, and partly discarded to landfills; T2, engineered timber is incinerated for bioenergy; T3, engineered timber is reused as functionally equivalent products, and this removes the incentive for forest regrowth; T4, engineered timber is recycled into lower quality and/or value products, and this removes the incentive for forest regrowth; T5, engineered timber is partly reused, partly recycled into lower quality/value products, and forestry and land management practices are applied to sustain the forest regrowth; T6, engineered timber is discarded to landfills with direct landfill gases (LFGs) release; T7, engineered timber is discarded to landfills, and LFGs are treated by enclosed flare; T8, engineered timber is discarded to landfills, and LFGs are captured and burned for energy production.

S1.4.4. Carbonation sensitivity

After building demolition, the concrete is assumed to be crushed into spherical particles of different distributions, and the particles are assumed to be fully exposed to the air. They, thus, have a much larger surface area, increasing the rate of CO_2 uptake at the end of life[8]. The carbonation capacity of concrete, therefore, differs based on exposure time to air while it stays as rubble. Supplementary Fig. S44 presents the AGTP results based on different exposure times. For each scenario, 10 years, and 5 years of exposure time provides ~%8 decrease in AGTP compared to 3 months.

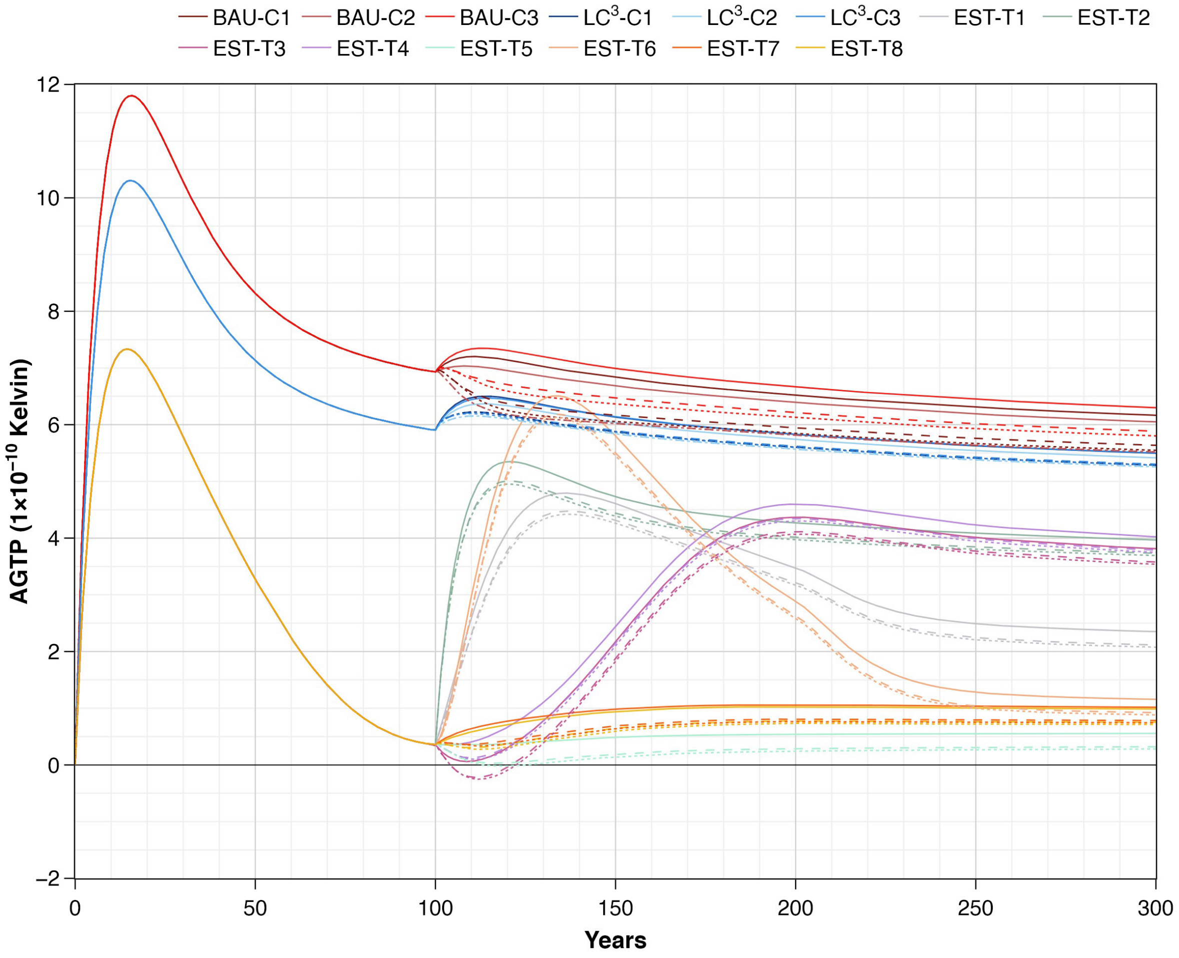

Supplementary Fig. S44 | Absolute global temperature potential (AGTP) of the case study building constructed in 2025 based on start-of-life and end-of-life urban building scenarios. The case study building has a 1,970 m^2 gross internal area with four storeys, and a lifespan of 100 years. The results are shown for SSP2. Solid lines represent that concrete rubble is exposed to air for 3 months after demolition. Dashed lines represent that concrete rubble is exposed to air for 5 years after demolition. The dotted lines represent that concrete rubble is exposed to air for 10 years after demolition. The results are shown for SSP2 greenhouse gas concentrations. BAU, reinforced OPC concrete building; LC^3, reinforced LC^3 concrete building; EST, engineered structural timber building; C1, concrete is partly recycled, and partly discarded to landfills; C2, concrete is recycled; C3, concrete is discarded to landfills; T1, engineered timber is partly recycled into lower-quality products, partly incinerated, and partly discarded to landfills; T2, engineered timber is incinerated for bioenergy; T3, engineered timber is reused as functionally equivalent products, and this removes the incentive for forest regrowth; T4, engineered timber is recycled into lower quality and/or value products, and this removes the incentive for forest regrowth; T5, engineered timber is partly reused, partly recycled into lower quality/value products, and forestry and land management practices are applied to sustain the forest regrowth; T6, engineered timber is discarded to landfills with direct landfill gases (LFGs) release; T7, engineered timber is discarded to landfills, and LFGs are treated by enclosed flare; T8, engineered timber is discarded to landfills, and LFGs are captured and burned for energy production.

S2. Supplementary discussion

The rapid transition to timber cities requires the rapid globalisation of policy practices related to timber building architecture and structural design, which only exist in a few countries/municipalities. For example, the city of Amsterdam mandated that 25% of all new residential buildings must be constructed with biobased materials including timber from 2025[9]. The current largest CLT building in the world, the Dalston Works project, was completed in 2017[10]. This 10-storey building is reported to weigh a fifth of a concrete building of the same size and reduced the number of deliveries during construction by 80% (e.g., lighter components, fewer trucks). Hybrid systems combining timber with concrete and/or steel can enable high-rise timber buildings such as the Mjøstårnet project in Norway (standing at 85.4 meters, glulam and CLT structure, timber floor in storeys 2-11, reinforced concrete slabs in storeys 12-18)[11], and HoHo Tower in Austria (standing at 84 meters, reinforced concrete core with CLT structure)[12]. Vancouver allows new mass timber buildings to be built with 2 (in zones permitting 8–11 storeys) or 3 (in zones permitting 12 or more storeys) additional storeys relative to other new building designs[13].

Investment in local mass timber supply chains reduces transport emissions and drives local economies. Public procurement of timber construction drives demand and supports the scaling of local mass timber production. Expansion of structural standards permits increased use of local tree species and reuse of existing timber stocks. Speedy upskilling of engineers, architects, and contractors increases confidence in delivery, especially in places without a lasting tradition of timber construction. Costly design or installation mistakes affect how timber construction is perceived by industry and society. Better knowledge of fire engineering and a clearer understanding of risk among insurers strengthen confidence in timber construction.

Regulation of whole life carbon at the project level and city-wide sectoral carbon budgets drive uptake of timber construction. Increasing trust in the measurement of the benefits of timber cities ensures appropriate measurement and inclusion of sequestration not only at the economy level but also at the project level, motivating designers, developers, and investors to use timber. Improved pricing of negative externalities of construction material use is achieved through effective carbon pricing or global taxation of non-renewable resource extraction or consumption.

Accurate quantification of end-of-life climate change impacts of timber buildings, and better decision-making on climate-sensitive design options requires a systemic multi-sector approach[14], which can be complex. Evidence for developing policies for large-scale transitions (e.g., national-level[15]) in the built environment should utilise dynamic LCA methods and indicators such as the AGTP. These national-level analyses can also consider the existing building stock restoration/demolition, local building typologies and material intensity, and future improvements in material production (e.g.,[16,17]). Application areas where simpler dynamic LCA methodology such as time factors[18] are appropriate include feasibility studies and early-stage design comparisons for smaller-scale projects. The time factor method is a simplified approach in dynamic LCA that applies time-based weighting factors to emissions based on when they occur over the product or building lifecycle, without requiring complex data or scenarios.

S3. Supplementary limitations

Our modelling framework assumes only one construction and demolition phase. This is because (1) we sought to keep our model as simple as possible, and (2) the existing SSP databases do not include population and emission projections beyond 2100. Future work can include more construction and demolition phases when more reliable data is available.

Determining a country/region-specific case study building, and conducting a detailed regional analysis for new urban buildings would require knowledge of the parameters of newly constructed buildings in each region, which would require a separate and extensive data collection and/or field investigation study. Considering these limitations, using a single modern building as a reference is arguably a more appropriate approach than relying on current heterogeneous regional material intensity data. We also expect that using a representative building and calculating a detailed bill of quantities provides more reliable results than using simplified global material intensity assumptions, as was done in previous large-scale timber city studies[19-21], since this ensures validity of both the building model and its corresponding material intensity.

The types of insulation materials used and their properties (e.g., thickness) can vary by region depending on climatic conditions. However, consistent with the literature, our results show that emissions from insulation materials account for only ~1% of the start-of-life emissions of a building. This indicates that variations in insulation materials do not significantly affect the generalisability of the overall results.

S4. Supplementary methods

S4.1. Goal and scope

Our dynamic LCA study assessed the life cycle impacts of buildings from cradle to grave, including both biogenic carbon and concrete carbonation. Life cycle stages are based on the International Organization for Standardization (ISO) standards (ISO 14040), and European Standards (EN 15978)[22]: product stage (e.g., raw material supply, manufacturing), construction process stage, use stage, end-of-life stage (e.g., demolition), and potential benefits and loads beyond the system boundary (e.g., recovery, reuse).

Based on the architectural plan of the case study building, two comparable preliminary structural designs were developed: (i) reinforced OPC and LC^3 concrete structural frames, and (ii) an engineered timber structural frame for the use of glulam (for columns and beams) and nail-laminated timber (NLT, for floors and roof) (for schematic visualisation see Supplementary Fig. S45, Supplementary Fig. S46, for detailed structural design reports see Supplementary Information S2[23]). For non-structural elements, all dimensions, materials, and layer structure of the elements (e.g., wall, insulation) were customized for both types of buildings. This is due to reinforced concrete buildings and engineered timber buildings typically using different materials for different building elements (e.g., concrete masonry block interior walls vs. CLT interior walls). We considered requirements in the International Building Code 2021 (IBC 2021) and the International Energy Conservation Code 2021 (IECC 2021).

We took into account structural materials, insulation materials (i.e., for water insulation, acoustic insulation, and fire protection), and other main materials necessary for a building (e.g., facade and wall materials including internal finishes such as cement screed), leaving out operational energy use (e.g., heating and electricity since building designs were developed to achieve the same operational performance), and interior materials (e.g., furniture), focussing on embodied carbon. These emissions typically account for ~96% of the total whole life embodied CO_2-eq. emissions of newly constructed residential buildings (46% superstructure, 21% substructure, 16% internal finishes, and 13% facade)[22]. The maintenance of buildings was also excluded from the scope. Hence, only biogenic carbon and concrete carbonation are considered in the use stage of the building, which are both linked to embodied emissions.

S4.2. Scenarios

Our study does not consider the demand for construction materials required for maintaining and renovating existing buildings or for reconstructing buildings after demolition. The exclusion of old building stock dynamics is based on two key considerations. First, although maintenance and retrofitting require timber products, the scale of this demand is currently relatively small compared to the material demand for new construction[24]. Second, although the scale of this demand is expected to increase in the future, incorporating existing building dynamics requires a different and complex methodological framework[25]. Estimating wood demand from renovation and reconstruction requires detailed data on existing buildings, including age, typology, material composition, and physical condition. It also depends on future renovation rates, which vary by region, policy, economic conditions, and user behaviour. Existing buildings vary widely in design, age, and construction materials, affecting renovation approaches and material requirements. The suitability of engineered structural timber products in these different renovation approaches is not well known[26]. Accurate data on the condition and renovation needs of existing structures are often scarce, leading to uncertainties in demand projections. The modelling of such demand involves building stock models, lifecycle analysis of existing structures, and scenario assumptions that differ from those used for new construction. We estimate that these models will carry high uncertainty due to limited data availability, and variable assumptions about future use, durability, and policy direction. Third, recent studies show that extending the life of buildings with major structural refurbishments, and repurposing buildings (e.g., old factories to residentials) may result in lower climate impacts compared to constructing new buildings, even those made of timber[27,28]. Therefore, including the dynamics of the existing building stock requires a separate study with detailed comparative analyses. In line with previous studies[19,20], we consider these aspects to be beyond the scope of our work.

S4.2.1. Start-of-life scenarios

Our study explores three start-of-life scenarios: (i) 'BAU' (business as usual) represents reinforced concrete cities, and buildings with reinforced concrete structure made with OPC; (ii) 'LC^3' represents reinforced concrete cities, and buildings with reinforced concrete structure made with LC^3; and (iii) 'EST' represents timber cities and buildings with engineered structural timber structure: glulam is used for columns and beams, and NLT is used for floors and roof.

S4.2.2. End-of-life scenarios

We considered a total of ten end-of-life scenarios based on the structural frame materials (i.e., start-of-life scenarios). For reinforced concrete frame buildings (i.e., 'BAU', and 'LC^3' start-of-life scenarios), there are three scenarios: (i) 'C1', concrete is partly recycled by crushing and reusing after demolition either as recycled concrete aggregate or as unbounded aggregate in groundwork or road construction (21%), and partly discarded to landfills (79%), representing current end-of-life concrete treatment share (see Supplementary Information S2 for our end-of-life construction waste treatment share analysis based on the waste database by Kaza et. al.[29]); (ii) 'C2', concrete is recycled; (iii) 'C3', concrete rubble is discarded to landfills.

For the engineered timber structural frame building (i.e., 'EST' start-of-life scenario), we considered eight scenarios: (i) 'T1', engineered timber is partly recycled into lower-quality products (24%), partly incinerated (18%), and partly discarded to landfills, where LFGs are released directly (54%), treated by an enclosed flare (2%), and captured and burned for energy production (2%), representing current end-of-life timber treatment share (see Supplementary Information S2 for our end-of-life construction waste treatment share analysis based on the waste database by Kaza et. al.[29]); (ii) 'T2', engineered timber is incinerated for bioenergy so that 100% of its stored CO_2 is emitted into the atmosphere at once; (iii) 'T3', engineered timber is reused in a new project as functionally equivalent products; (iv) 'T4', engineered timber is recycled by chipping, and lower quality and/or value products are produced; (v) 'T5', engineered timber is partly reused in new projects in functionally equivalent products (50%), and is partly recycled by chipping and lower quality and/or value products are produced (50%); (vi) 'T6', engineered timber is discarded to landfills, and LFGs are directly emitted into atmosphere; (vii) 'T7', engineered timber is discarded to landfills, and LFGs are treated by enclosed flare; (viii) 'T8', engineered timber is discarded to landfills, and LFGs are burned for energy production.

For wood use in reinforced concrete buildings, we assumed that these woods are treated based on the T1 scenario. For concrete use in engineered timber buildings, we assumed that these concretes are treated based on the C1 scenario.

Combinations of start-of-life and end-of-life scenarios represent the whole life cycle of each building. For example, EST-T8 indicates timber cities, and buildings with engineered timber structural frame. After the demolition of buildings, the engineered timber will be sent to controlled landfills where LFGs are captured, converted, and burned to produce energy.

Our end-of-life scenarios also explore the most ideal solutions. For example, in the T3, T4, and T5 scenarios, our assumption of 100% recovery of timber waste can be theoretically possible but is not achievable in practice (e.g. due to yield losses in manufacturing). These assumptions are made to highlight differences with other pathways, and to find the best possible solutions. Our study shows that the AGTP, GWP, and GHG concentration results for possible scenario variations (e.g., 50% recycling, 20% incineration, 30% landfilling for end-of-life timber) will vary between the presented scenario results such as in the EST-T1 scenario.

S4.2.3. End-of-life substitution credits

When concrete and engineered timber are recovered, it may affect raw material extraction (i.e., aggregate production, and wood harvesting), and the energy system (i.e., bioenergy and fossil fuel supply). We modelled these effects as end-of-life substitution credits in our dynamic LCA: In the 'C1', and 'C2' end-of-life scenarios, recovered concrete reduces virgin aggregate production and associated emissions.

In ‘T1’ and ‘T2’ end-of-life scenarios, timber incineration supplies bioenergy which avoids other energy sources and associated emissions. This substitution is modelled based on the future global electricity production emissions projected by Regionalized Model of Investment and Development (REMIND) integrated assessment models (IAM) scenarios. In the ‘T3’ scenario, reusing engineered timber as functionally equivalent products avoids new engineered timber production and associated emissions in line with ref.[30].

In the ‘T3’ and ‘T4‘ scenarios, recovered engineered timber reduces new wood harvesting, and it is assumed that this removes the incentive to grow new trees and associated future CO_2 sequestration (in ‘T3’, new harvesting for construction use, and in ‘T4’, new harvesting for paper and wood-based panel production are assumed to reduce). This assumption is consistent with a limited timber market. In the ‘T5’ scenario, recovered engineered timber reduces virgin timber demand; however, proactive forestry and land management practices such as preventing existing and possible future deforestation, encouraging regeneration of natural forest, planned harvesting and reafforestation (e.g., including wood vaulting) are carried out to sustain the forest carbon sink. Policies promoting wood use for other societal benefits (e.g., furniture) could also help sustain timber demand and the forest carbon sink.

In the ‘T8’ scenario, captured and burned LFGs supply energy which avoids other energy sources and associated emissions. This substitution is also modelled based on the future global electricity production emissions projected by REMIND-IAM scenarios.

In scenarios where wood is landfilled (T6-8), the discarded material does not re-enter the market and therefore does not contribute to meeting demand for new wood-based products. As a result, market demand continues to be supplied by virgin wood, maintaining current incentives for forestry activities and forest regrowth. In contrast, scenarios with high levels of wood recycling (T3-T5) offset demand for virgin wood, which can reduce harvesting incentives and affect forest dynamics.

Supplementary Table S2 | The urban building end-of-life scenarios and substitution credits modelled in this study.

End-of-life scenarios (2125-2200) Waste treatment types and descriptions				End-of-life substitution credits (2125-2200)
1	C1	Mixed	Concrete is partly recycled (21%), and partly discarded to landfills (79%) (C1).	Recovered concrete reduces virgin aggregate production (50% gravel and 50% sand) and associated emissions.
2	C2	Recycling	Concrete is recycled (C2).	Recovered concrete reduces virgin aggregate production (50% gravel and 50% sand) and associated emissions.
3	C3	Landfilling	Concrete is discarded to landfills (C3).	No substitution credits.
4	T1	Mixed	Engineered timber is partly recycled into lower-quality products (24%), partly incinerated (18%), and partly discarded to landfills, where LFGs are released directly (54%), treated by an enclosed flare (2%), and captured and burned for energy production (2%) (T1).	Timber incineration supplies bioenergy which avoids other energy sources and associated emissions. This substitution is modelled based on the future global electricity production emissions projected by REMIND-IAM scenarios. Recycled engineered timber reduces the demand for virgin wood harvesting, and removes the incentive to forest regrowth.
5	T2	Incineration	Engineered timber is incinerated for bioenergy (T2)	Timber incineration supplies bioenergy which avoids other energy sources and associated emissions. This substitution is modelled based on the future global electricity production emissions projected by REMIND-IAM scenarios.
6	T3	Recycling	Engineered timber is reused in new projects in functionally equivalent products (T3).	Reusing engineered timber as functionally equivalent products (e.g., CLT) avoids new engineered timber production and associated emissions. Reused engineered timber reduces the demand for virgin wood harvesting, and removes the incentive to forest regrowth.
7	T4	Recycling	Engineered timber is recycled by chipping, and lower quality and/or value products are produced. This reduces the demand for virgin wood harvesting, and removes the incentive to forest regrowth (T4).	Recycled engineered timber as lower quality/value products (e.g., particle boards) avoids new lower/value quality timber production and associated emissions. Recycled engineered timber reduces the demand for virgin wood harvesting, and removes the incentive to forest regrowth.
8	T5	Recycling	Engineered timber is partly reused in new projects in functionally equivalent products (50%), and is partly recycled by chipping, and lower quality and/or value products are produced (50%).	Reusing engineered timber as functionally equivalent and lower quality/value products avoids new engineered timber and lower quality/value timber production and associated emissions. Proactive forestry and land management practices are applied to sustain the carbon sink.
9	T6	Landfilling	Engineered timber is discarded to landfills, and LFGs are directly emitted into the atmosphere (T6).	No substitution credits.
10	T7	Landfilling	Engineered timber is discarded to landfills, and LFGs are treated by enclosed flare (T7).	No substitution credits.
11	T8	Landfilling	Engineered timber is discarded to landfills, and LFGs are captured and burned for energy production (T8).	Captured and burned methane supplies energy which avoids other energy sources and associated emissions. This substitution is modelled based on the future global electricity production emissions projected by REMIND-IAM scenarios.

Abbreviations: REMIND-IAM, Regionalized Model of Investment and Development-Integrated Assessment Models; LFGs, landfill gases; CLT, cross-laminated timber.

S4.3. Case study building

We used a mid-rise urban residential/commercial building case study to compare the life cycle impacts of residential buildings with different structural frame materials including various end-of-life scenarios using dynamic LCA. These materials are (1) reinforced concrete with ordinary Portland cement, (2) reinforced concrete with LC^3, and (3) engineered timbers of glue-laminated timber (glulam), and nail-laminated timber (NLT). This case study uses a 1,970 m^2 gross internal area building as its functional unit, with four storeys, and a lifespan of 100 years. The case study building is located in New Haven, Connecticut, United States of America (USA).

Based on the architectural plan of the case study building, two comparable preliminary structural designs are developed: (1) reinforced concrete structural frame for the use of ordinary Portland cement, and LC^3, and (2) engineered timber structural frame for the use of glulam, and NLT (Supplementary Fig. S45, Supplementary Fig. S46) (for structural analysis, See Supplementary Information S2[23]). For non-structural elements, all dimensions, materials, and layer structure of the elements (e.g., wall, insulation) are customized for both types of buildings, as reinforced concrete buildings and engineered timber buildings typically use different materials for different building elements. For this, we consider requirements in the International Building Code 2021 (IBC 2021) and the International Energy Conservation Code 2021 (IECC 2021).

Supplementary Fig. S45 and Supplementary Fig. S46 present the schematic visualisation for the reinforced concrete, and engineered timber structural designs of the case study building, respectively. Based on these structural designs, Supplementary Table S3, and Supplementary Table S4 present the insulation material design, and U-values of buildings' envelopes for reinforced concrete, and engineered timber buildings, respectively. Detailed quantities of all materials based on these building designs are provided in Supplementary Information S2[23].

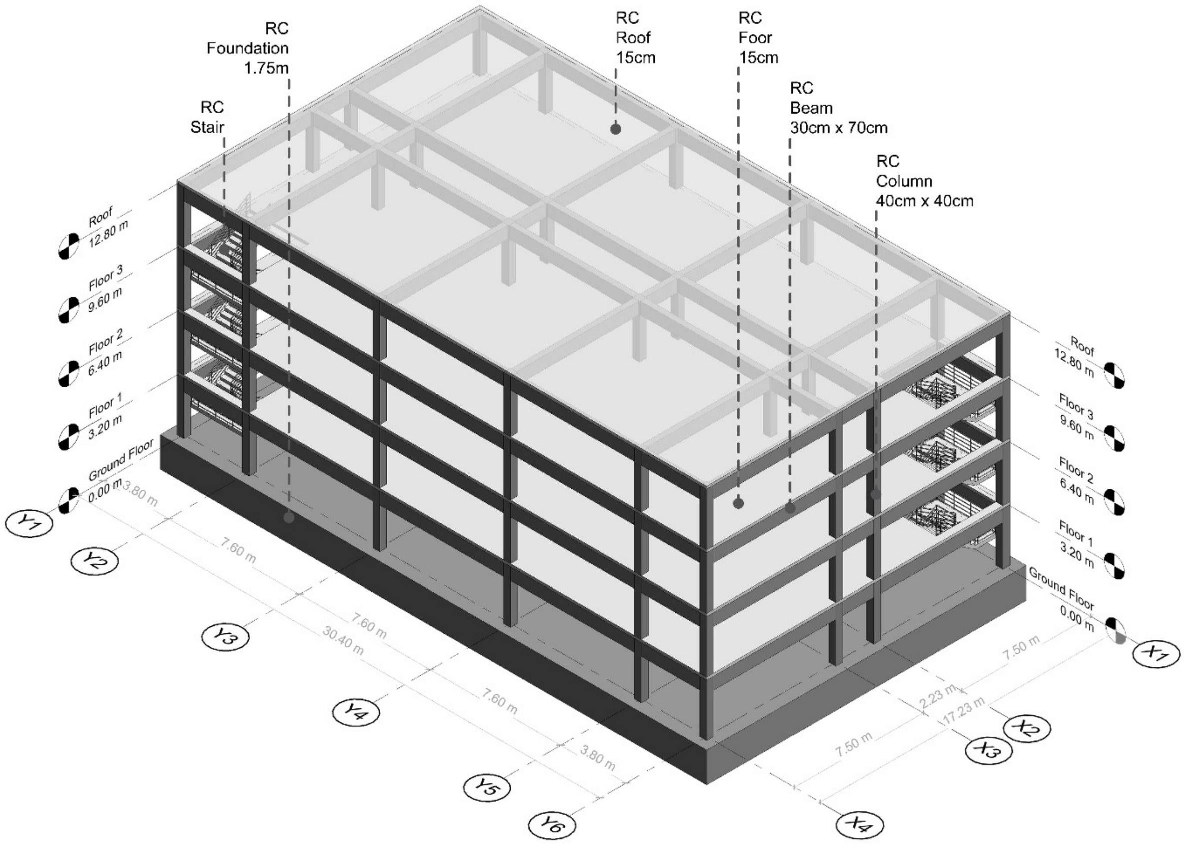

Supplementary Fig. S45 | Schematic visualisation for the reinforced concrete structural design of the case study building.

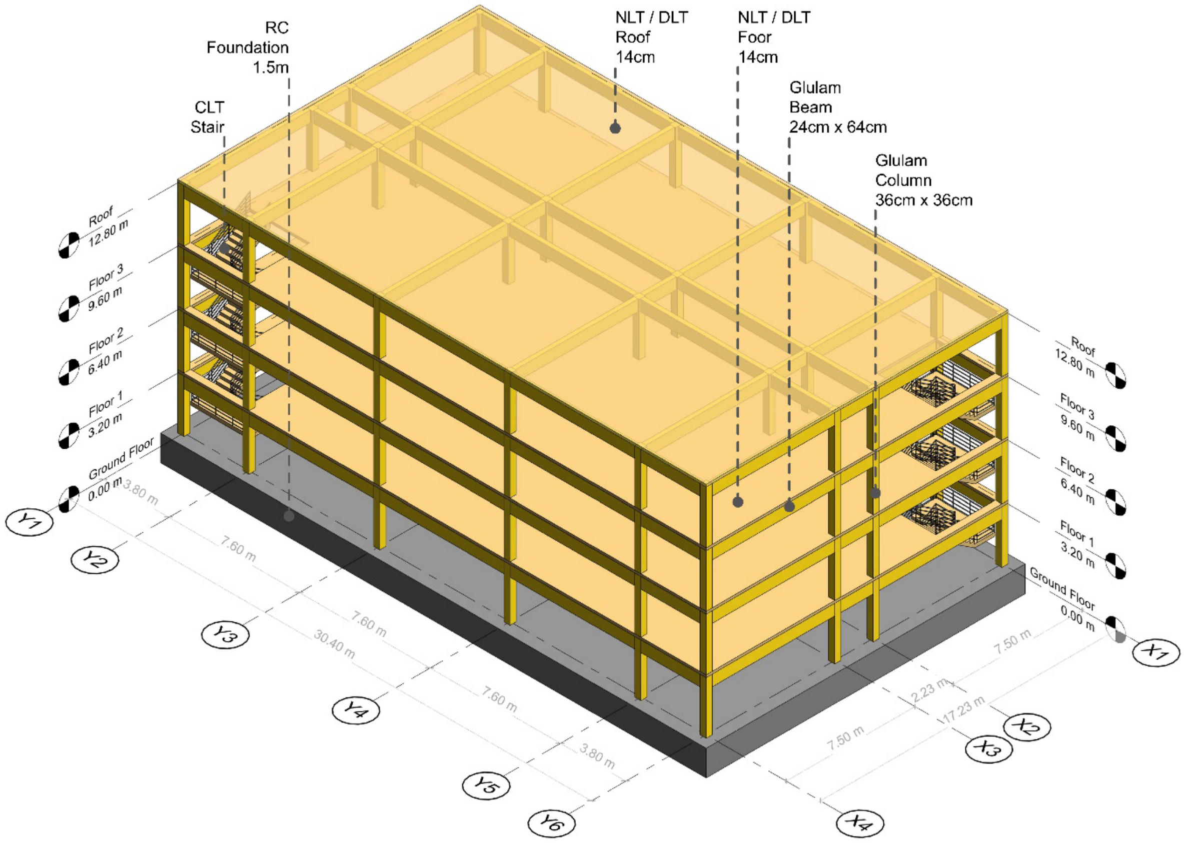

Supplementary Fig. S46 | Schematic visualisation for the engineered timber structural design of the case study building.

Supplementary Table S3 | Insulation materials for the reinforced concrete structural design of the case study building, and U-values of building envelope.

Building Element	Material (Inside to outside)	Thickness (mm)	Thermal conductivity (W/m·K)	Raw density (kg/m^3)	Heat capacity (J/kg·K)	U-values of a building envelope (W/m^2·K)
Foundation	Cement screed	60	1.40	2000	1000	0.246
	Foil, EPDM	1	0.25	1200	1000	
	EPS	120	0.04	20	1500	
	Waterproofing membrane	5	0.17	1050	1000	
	Reinforced concrete	1750	2.30	2300	880	
	Foil, EPDM	1	0.25	1200	1000	
	Gravel	100	2.00	2200	1000	
Intermediate Floor	Acoustic insulation	12	0.045	15	1400	-
	Reinforced concrete	150	2.30	2300	880	
Interior Wall	Concrete masonry units	100	0.09	350	1000	-
Exterior Wall	Concrete masonry units	100	0.09	350	1000	0.210
	Vapor retarder	1	0.22	260	1700	
	EPS	120	0.04	20	1500	
	Vapor retarder	1	0.22	260	1700	
	Air layer and cladding frame	40	0.23	1.2	1000	
	Wood Cladding	20	0.09	375	1600	
Flat Roof	Reinforced concrete	150	2.30	2300	880	0.129
	Waterproofing membrane	1	0.17	1050	1000	
	Vapor retarder	1	0.22	260	1700	
	EPS	300	0.04	20	1500	
	Foil, EPDM	1	0.25	1200	1000	
	Gravel	50	2.00	2200	1000	

Supplementary Table S4 | Insulation materials for the engineered timber structural design of the case study building, and U-values of building envelope.

Building Element	Material (Inside to outside)	Thickness (mm)	Thermal conductivity (W/m·K)	Long-wave radiation emissivity	Diffusion resistance factor	Raw density (kg/m^3)	U-values of building envelope (W/m^2·K)
	Cement screed	60	1.40	0.90	15/35	2000	
	Foil, EPDM	1	0.25	0.90	6000/32000	1200	
	EPS	120	0.04	0.90	20/100	20	
Foundation	Waterproofing membrane	5	0.17	0.90	10000/80000	1050	0.253
	Reinforced concrete	1500	2.30	0.97	80/130	2300	
	Foil, EPDM	1	0.25	0.90	6000/32000	1200	
	Gravel	100	2.00	0.90	50	2200	
Intermediate Floor	OSB acoustic insulation	12	0.13	0.9	30/300	650	-
	NLT	160	0.13	0.90	40/200	500	
Interior Wall	CLT	60	0.13	0.90	40/200	500	-
	CLT	90	0.13	0.90	40/200	500	
	Vapor retarder	1	0.22	0.9	4600	260	
Exterior Wall	EPS	130	0.04	0.9	20/100	20	0.217
	Vapor retarder	1	0.22	0.9	4600	260	
	Air layer and cladding frame	40	0.23	0.9	1	1.2	
	Wood Cladding	20	0.09	0.9	20/50	375	
	NLT	100	0.13	0.90	40/200	500	
	Waterproofing membrane	1	0.17	0.90	10000/80000	1050	
Flat Roof	Vapor retarder	1	0.22	0.9	4600	260	0.133
	EPS	260	0.04	0.9	20/100	20	
	Foil, EPDM	1	0.25	0.90	6000/32000	1200	
	Gravel	50	2.00	0.90	50	2200	

S4.4. Life cycle inventory

S4.4.1. Material quantities

For the quantities of materials, we modelled the case study building in 3D using Revit software, creating detailed representations for each start-of-life scenario. These models are based on the architectural plan of the case study building. For structural frame elements (e.g., columns, beams) of buildings, we incorporate a preliminary structural analysis conducted specifically for this study. This is to obtain the most accurate material quantities for both reinforced concrete and engineered timber structural frames instead of using simple material intensity values (see Supplementary Information S2[23] for detailed structural analysis reports).

We also took into account the calculations of thermal transmittance of related building elements (e.g., for insulation materials), and other material needs (e.g., wall and façade materials) within the scope of the study (Supplementary Tables S3,4) (see Supplementary Information S2[23] for detailed bill of quantities).

Environmental product declarations (EPDs) were used to obtain material density information where such data were not available in the ecoinvent database. For example, some start-of-life processes require volume-based inputs (e.g., CLT), whereas end-of-life processes (e.g., end-of-life timber waste) require mass-based inputs.

S4.4.2. Start-of-life and end-of-life emissions

For start-of-life emissions, we modelled the life cycle inventory for 2025 using the ecoinvent[31] database (v3.10.1, cut-off system model), and life cycle inventories for 2050 and 2100 using IAM-integrated datasets generated from the same ecoinvent database through the *premise* framework by Sacchi et al.[32]. For intermediate years, we projected start-of-life emissions using (Equation S1), which implies a fast initial reduction in emissions followed by a progressively slower decline. Supplementary Fig. S47 presents the results for annual start-of-life emission projections by 2100. For end-of-life scenarios, emission values for 2100 were derived separately for SSP1, SSP2, and SSP5 using the premise framework, and the same inventory was applied to all demolition and waste management activities occurring after 2100.

For start-of-life scenarios, detailed unit processes were identified to represent material production including raw material supply, transport, and construction of buildings (i.e., product stage, and construction stage). For end-of-life scenarios, detailed unit processes were identified to represent building demolition, and specified waste treatment methods in the scenarios (i.e., end-of-life stage, and benefits and loads beyond the system boundary). All unit processes also included average transportation distance and energy needs (see Supplementary Information S2[23] for detailed unit processes).

$$E(t)=C+A\cdot e^{k(t-y_1)} \quad \text{(S1)}$$

Where $E(t)$ is the start-of-life emission at time t (years). C represents the minimum emission level reached in the long term. A defines the emission difference between the initial year and the minimum level. The parameter k controls the rate of emission reduction, with higher values indicating a faster initial decline. y_1 denotes the reference year from which the emission reduction trajectory starts. This functional form captures a rapid early decrease in emissions followed by a progressively slower reduction over time.

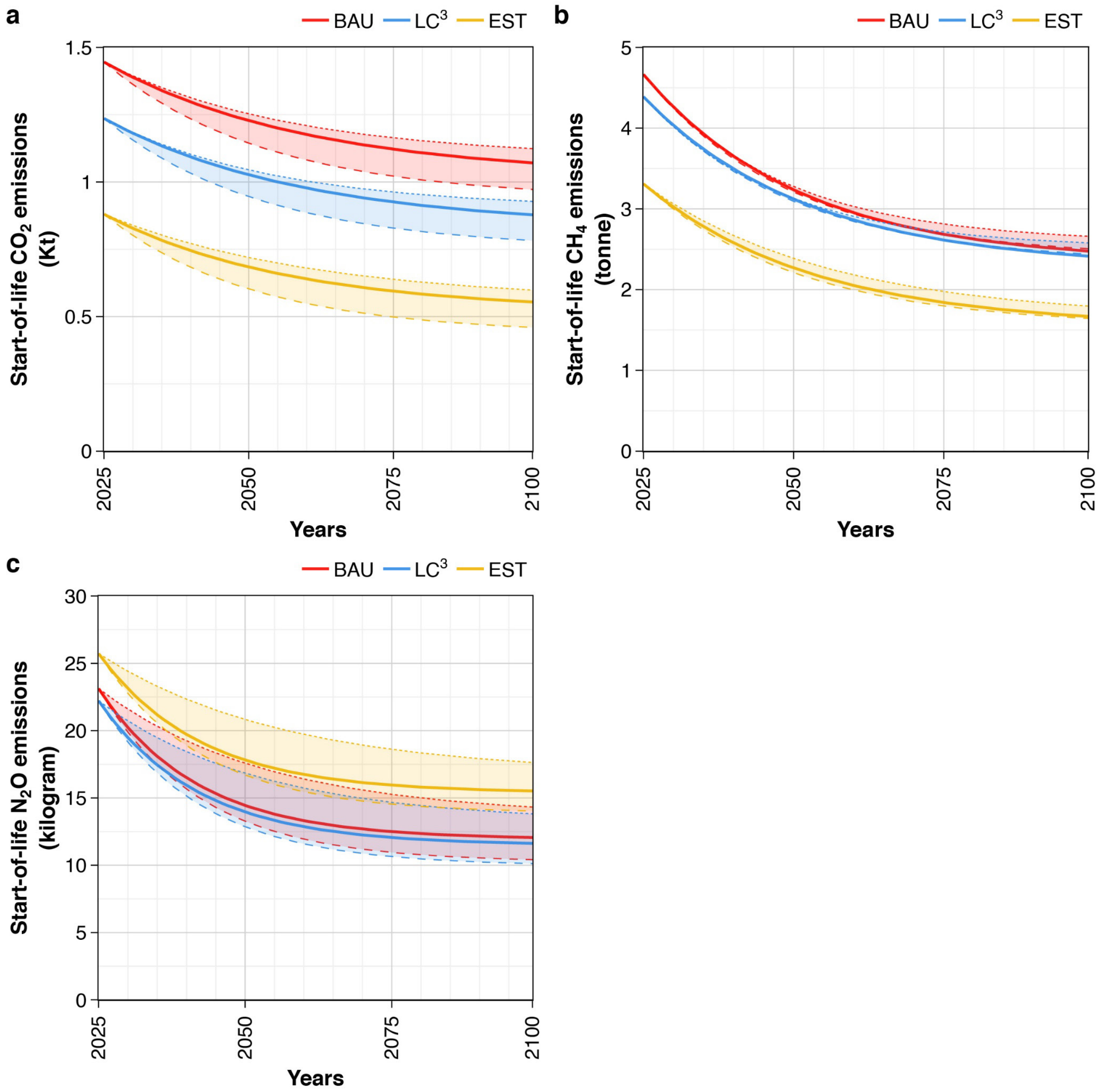

Supplementary Fig. S47 | Start-of-life emission projections of the representative case study building in this study by 2100. a Start-of-life CO_2 emissions, **b** Start-of-life CH_4 emissions, **c** Start-of-life N_2O emissions. The graphs show the results for the REMIND-SSPs-Base scenario which represents a current-policies scenario reflecting existing climate policies without full implementation of announced pledges. The main results of this study (i.e., AGTP, GWPs, Atmospheric GHG level results presented in the main text) uses REMIND-SSPs-Base scenario start-of-life and end-of-life emissions. For each scenario, the solid line (SSP2), the dashed line (SSP1), and the dotted line (SSP5) represent shared socioeconomic pathways (SSPs), with the shaded area between these lines quantifying uncertainty. Start-of-life scenarios for future urban buildings cover the construction period between 2025 and 2100. BAU, reinforced OPC concrete building; LC^3, reinforced LC^3 concrete building; EST, engineered structural timber building.

S4.5. Dynamic life cycle assessment

S4.5.1. Biogenic carbon sequestration

For carbon sequestration through biomass growth, two alternative approaches can be used in dynamic LCA: the 'backward-looking' or the 'forward-looking' approach. The backward-looking approach models sequestration over the period leading up to the harvest of timber, following the carbon physically present in the timber product. The forward-looking approach follows carbon sequestration in the newly planted trees which replace those harvested. In this study, we applied the forward-looking approach, assuming that atmospheric carbon starts to be sequestered during biomass regrowth after wood harvest.

Biomass regrowth is modelled as a Gaussian distribution, expressed as a function of biomass growth rotation period[33] (Equation S2). Forest management that is designed to mitigate climate change may lead to increased rotation periods to enhance the carbon storage capacity of forests[34]. We assumed a rotation period of 100 years, based on an average of currently recommended rotation lengths in Nordic, Continental and Mediterranean climates[35].

$$g(t)=\frac{1}{\sqrt{2\pi\sigma^2}}\cdot e^{\frac{-(t-\mu)^2}{2\sigma^2}} \qquad \text{(S2)}$$

Where *g(t)* is the forest regrowth rate, and *t* (years) is the time. We assume 100 years for the rotation period, *r*. The parameters μ (mean) and σ (variance) is assumed to be *r*/2, and *r*/4, respectively[33].

It is assumed that as much biomass will regrow as the timber we use in engineered wood buildings. A carbon-to-wood ratio (*CW*) of 0.5 is assumed for all tree species. This means that 1 kg of wood sequesters 0.5 kg of carbon (C) or 1.83 kg of CO_2 after the appropriate rotation period. Here, we only model one rotation period, and sequestration takes place between 0 and 100 years. Emissions from loss of carbon pools other than the forest itself, like soil and litter, are not considered.

S4.5.2. Degradable organic carbon in landfills

The degradation of carbon in landfills is modelled using the first-order decay method, a method used by the IPCC 2006 guidelines[36] (Equation S3):

$$DDOCm = DDOCm_0\cdot e^{-kt} \qquad \text{(S3)}$$

where *DDOCm* is the mass of degradable organic carbon that will decompose at time *t* (years), $DDOCm_0$ is the mass of degradable organic carbon at time 0, and *k* ($years^{-1}$) is the decay rate constant. According to the EPA[37], the average half-life of decaying wood is 29 years, and thus; *k* is found as ln2/29 $years^{-1}$.

S4.5.3. Carbonation

As hydration products in Portland cement paste react with CO_2 dissolved in the pore solution, there is a drop of pH below 9. The main hydration product driving the reaction is portlandite, as shown in (Equation S4)[38]:

$$Ca(OH)_2 + CO_2 \xrightarrow{H_2O} CaCO_3 + H_2O \qquad \text{(S4)}$$

We use the calculation method by Lagerblad[39], and by Pomer and Pade[40] considering appropriate parameters (e.g., supplementary cementitious material coefficient) for both types of concrete with ordinary Portland cement, and LC^3. This is an adaptation of Fick's first law of diffusion, and this approach quantifies the progression of a one-dimensional (1D) carbonation front from the concrete surface. It assumes a linear decrease of the CO_2 concentration from the surface to the carbonation front at a certain distance where the concentration is negligible[41]. The concrete behind the front is considered fully carbonated which allows for the reaction kinetics to be neglected[42]. Although reaction

kinetics are not modelled explicitly, they are implicitly accounted for by treating carbonation as a diffusion-controlled process, in which the temporal progression of the carbonation front governs CO_2 uptake over time. (Equation S5 is used to find carbonation depth in compliance with the EN 16757 (2017)[43]):

$$x(t) = k_1 \cdot k_2 \cdot k_3 \cdot \sqrt{t} \qquad \text{(S5)}$$

where x (mm) is the carbonation depth at time t, t (years) is the time of exposure, k_1 is a coefficient related to strength and environmental classes, k_2 is a correction for surface treatment, and k_3 includes the effect of supplementary cementitious material (SCM). The product of these three coefficients is the carbonation rate expressed in length per square root of exposure time (mm years$^{-1/2}$).

Once the depth of the carbonation layer is found, the amount of captured CO_2, C_u, can be found as (Equation S6):

$$C_u(t) = x(t) \cdot A \cdot f_{concrete}^{CO_2} \qquad \text{(S6)}$$

where, C_u (kg) is the carbon uptake at time t (years), x (m) is the carbonation depth at time t, A (m^2) is the exposed surface area, and $f_{concrete}^{CO_2}$ (kg m^{-3}) is the mass of CO_2 captured per cubic meter of concrete. It is assumed that all the CO_2 is bound by reaction with CaO. $f_{concrete}^{CO_2}$ is hence defined as (Equation S7):

$$f_{concrete}^{CO_2} = c \cdot C_{clinker} \cdot f_{CaO} \cdot \gamma \cdot \frac{M_{CO_2}}{M_{CaO}} \qquad \text{(S7)}$$

where, c (kg m^{-3}) is the cement content in the concrete depending on the mix. $C_{clinker}$ (wt.%) clinker content in cement, f_{CaO} (wt.%) CaO content in clinker, γ is a factor representing the assumed proportion of CaO that will convert to $CaCO_3$, and $\frac{M_{CO_2}}{M_{CaO}}$ is the ratio of the molar mass of CO_2 and CaO, a constant equal to 0.7848. In much of the literature, γ is assumed to be 0.80 (ranging from 0.50 to 1.00)[44-46]. Here, a value of 1.00 is taken, since with enough time, all of the portlandite will react with CO_2.

After building demolition, the concrete is assumed to be crushed into spherical particles of different distributions, and the particles are assumed to be fully exposed to the air. They, thus, have a much larger surface area, increasing the rate of CO_2 uptake at end of life[8]. For all scenarios, we assumed a three-month exposure time after building demolition (for sensitivity based on exposure time, see Section S1.4.4). This presents an upper boundary for carbon uptake at the end-of-life stage since crushed concrete is expected to have irregular shapes and be kept in piles with uneven access to atmospheric CO_2.

The internal carbonation rate of concrete is directly proportional to the square root of the molar concentration of CO_2 in the gas phase of the pore expressed in mass units per volume. As human activities keep emitting CO_2 into the atmosphere, we expect this concentration to rise throughout the life of the building. We calculated carbonation for various CO_2 concentration values calculated by Meinshausen, et al.[47] based on SSPs[48]. SSP2-4.5 (2.1-3.5 °C warming by 2081-2100 relative to 1850-1950) is considered the base scenario as it describes the 'middle of the road' (O'Neill et al. 2014). SSP1-1.9 (1.0-1.8 °C warming by 2081-2100 relative to 1850-1950) and SSP5-8.5 (3.3-5.7 °C warming by 2081-2100 relative to 1850-1950) give a range of plausible carbonation values throughout the life of the building.

Due to its lower CaO content and different properties, we expect LC^3 concrete to carbonate at a different rate from Portland cement concrete. Shah et al.[49] show that pores in LC^3 concrete increase in size during carbonation contrary to Portland cement concrete. We would then expect carbonation to accelerate with time. This has been modelled in the literature by von Greve-Dierfeld et al.[41] and You et al.[50] for other types of SCMs by a carbonation depth proportional to the exposure time raised the power of an empirical exponent (n). For LC^3, this is corroborated by Gettu et al.[51] who find a value of 0.9 for the empirical exponent value. However, the study only provides three data points to reach this

conclusion. Shah and Bishnoi[52] and Rathnarajan et al.[53] find a good fit between their experimental carbonation data and the empirical exponent with a value of 0.5 despite the increase in porosity. This is consistent with Lagerblad[39]. Results from Rathnarajan et al.[53] also appear to show that the carbonation coefficient of LC^3 is higher than that of concrete with 30% fly ash and that of concrete with no SCM. The average ratio of carbonation coefficient for concrete with fly ash and with Portland cement is 1.1[53] which is consistent with Lagerblad[39]. Taking the same ratio for LC^3, a value of 1.7 is found. Here, we model the carbonation of LC^3 concrete with the Lagerblad model using the value of 1.7 for k_3.

In this study, Equation S7 is also used to calculate the amount of CO_2 bound per cubic meter of carbonated LC^3 concrete. Based on Gettu et al.[51], the value of CaO in the LC^3 cement (f_{CaO}) was taken to be 39.7%. For γ, the same value (1.00) was used for the aspirational and BAU scenarios.

S4.5.4. Atmospheric decay of emissions

Once greenhouse gases are emitted into the atmosphere, their concentration initially rises but gradually decreases as they are removed by the ocean and biosphere uptake. First, a fraction of the GHG released is quickly stored in the ocean's upper layer. The transport of this GHG to the deep ocean layers is slower. When the uptake by the GHG sink increases, the GHG initially stored in the ocean's upper layer will be released back into the atmosphere at a low rate to compensate for the initial over-absorption (out-gassing).

This atmospheric decay of emissions can be modelled by a single or a sum of exponentials known as impulse response function (IRF). For most gases such as methane (CH_4) or dinitrogen monoxide (N_2O), IRF is based on a simple exponential decay (Equation S8):

$$y_x(t) = e^{\left(\frac{-t}{\tau_x}\right)} \qquad \text{(S8)}$$

Where, $y_x(t)$ represents the time-dependent abundance of gas x caused by the additional emission of one kg of gas x at time 0, t (years) is the time, and τ_x (years) is the perturbation lifetime. τ_{CH_4} for CH_4, and τ_{N_2O} for N_2O are 12.4 and 121 years, respectively[36].

For CO_2, the atmospheric response time cannot be represented by a simple exponential. Instead, it is usually approximated by a sum of exponentials and is widely known as the Bern 2.5 carbon cycle model[1]. This IRF has been estimated from a 40 Gt C impulse CO_2 emission, assuming background CO_2 atmospheric concentrations of 378 ppm (Equation S9).

$$y_{CO_2}(t) = A_0 + A_1 \cdot e^{\left(\frac{-t}{\tau_1}\right)} + A_2 \cdot e^{\left(\frac{-t}{\tau_2}\right)} + A_3 \cdot e^{\left(\frac{-t}{\tau_3}\right)} \qquad \text{(S9)}$$

where, A_0, A_1, A_2, and A_3, unitless coefficients represent a fraction that is associated with certain perturbation lifetimes of T_1, T_2, T_3 and (years). A_0 represents the asymptotic airborne fraction of CO_2 which remains in the atmosphere indefinitely (approximately 22%). For coefficients, and perturbation lifetime values, we relied on Joos et al.[1] (Table 5, A_0: 0.2173, A_1: 0.2240, A_2: 0.2824, A_3: 0.2763, T_1: 394.4 years, T_2: 36.54 years, T_3: 4.304 years).

It is important to note that reversibility is assumed between a GHG pulse and a GHG sink. This is a simplification but is widely accepted, and has a physical interpretation. This assumption is not entirely accurate as the climatic system does not behave in a perfectly symmetrical way[54] yet it is commonly accepted in the literature. In the long term, an equilibrium is found between any atmospheric source and sink[55]. For yearly time-distributed GHG emissions and removals, keeping track of atmospheric concentrations implies a combination of these fluxes with their respective IRFs. Thus, each year, the net atmospheric change considers the emissions for that year as well as the previous emissions as they are decaying. In mathematical terms, this is modelled as a convolution between the emission and removal functions with the GHG decay from the air[55] (Equation S10):

$$f(t) = \underbrace{C_0 \int_0^t e(t')y(t-t')\mathrm{d}t'}_{Emissions} - \underbrace{C_0^* \int_0^t g(t')y(t-t')\mathrm{d}t'}_{Sequestrations} \quad \text{(S10)}$$

where, t' (years) is the integration variable from year 0, t (years) is the time, C_0 (kg) is the intensity of the emission, C_0^* (kg) is the intensity of the removal, $e(t')$ (kg years^{-1}) is the emission function, $g(t')$ (kg years^{-1}) is the removal rate from the atmosphere (due to biomass growth or carbonation), $y(t)$ is the IRF for the GHG considered. Here we can see that the emissions and sinks are considered to behave in a perfect mirror of one another.

In this dynamic LCA study, OpenLCA is used as a tool to estimate the magnitudes of each anthropogenic emission, C_0, and C_0^*. Then, the net flows for CO_2, CH_4, and N_2O are computed in Python. This method has been introduced to the LCA methodology for timber products by Cherubini et al.[33]. Here we adapt it to take into account the carbonation of concrete products.

S4.5.5. Life cycle impact assessment

S4.5.5.1. Static global warming potential

The standard metric for climate change is global warming potential (GWP), expressed in units of kg CO_2-eq. For static analysis, all emissions are assumed to happen at the same time (year 0), and GWP_{static} is computed as (Equation S11) (IPCC 2013):

$$GWP_{static} = \sum_x \left(\frac{a_x \int_0^{THI} C_x(t)\mathrm{d}t}{a_{CO_2} \int_0^{THI} C_{CO_2}(t)\mathrm{d}t} \right) \quad \text{(S11)}$$

where, x represents the index for each GHG, THI (years) is the time horizon impact (years), a_x (W m^2 kg^{-1}) is the specific radiative efficiency of related GHG, a_{CO_2} (W m^2 kg^{-1}) is the specific radiative efficiency of CO_2, $C_x(t)$ (kg) is the degradation curve of a unit pulse emission of related GHG, and $C_{CO_2}(t)$ (kg) is the degradation curve of a unit pulse emission of CO_2.

To assess progressive sinks using GWP_{static} the cumulative mass of GHG sequestered at the end of the observation period was considered as a negative emission in year 0.

S4.5.5.2. Dynamic global warming potential

The current and common version of the dynamic GWP[56] could lead to an overestimation of the possible benefits of temporary carbon storage. It should also be noted that dynamic GWP indicators have increasingly been discussed as a form of discounting, rooted in economic considerations rather than climate physics. This reflects the fact that GWP-based metrics were originally developed to compare the climate impacts of different greenhouse gases, rather than to assess the temporal evolution of the warming influence of a single gas[57].

Here, we used the new expression of dynamic GWP by Ventura et al.[58], which takes into account the totality of the flows and the entireness of their impact according to the time horizon of the Impact (THI) chosen. The THI is used to calculate the GWP and, according to LCA principles, must be the same for all GHGs contributing to the climate change impact category[58]. Whenever a substance is emitted, the GWP must be calculated on the totality of the THI. Hence, the dynamic indicator $GWP_{dynamic}$ was calculated using full THI integration time for all emissions at the numerator, regardless of the emission time. The time integration at the denominator was extended from zero to the sum of THI and emission time for each substance as shown in (Equation S12):

$$GWP_{dynamic} = \sum_x \sum_i \left(\frac{a_x \int_0^{t_i + THI} C_x(t - t_i)\mathrm{d}t}{a_{CO_2} \int_0^{t_i + THI} C_{CO_2}(t)\mathrm{d}t} \right) \quad \text{(S12)}$$

where, x represents the index for each GHG, i represents the index for the emission time of related GHG during the time considered, t_i (years) is the emission time, *THI* (years) is the time horizon impact, a_x (W m^2 kg^{-1}) is the specific radiative efficiency of related GHG, a_{CO_2} (W m^2 kg^{-1}) is the specific radiative efficiency of CO_2, $C_x(t-t_i)$ (kg) is the degradation curve of a unit pulse emission of related GHG, and $C_{CO_2}(t)$ (kg) is the degradation curve of a unit pulse emission of CO_2.

Three important durations are distinguished[58]: (1) time horizon impact, THI; (2) life cycle duration, LCD, and (3) total observation duration, TOD. The first is the duration over which the decay of emissions is considered. Here, THI values of 20,100, and 200 years will be considered. LCD is the time between the first and last emission. Here, this is equal to the time between the construction of the building and the end of the exposure period at the end of the building lifespan. As this last period is investigated for a length of up to 100 years, the maximum LCD is 200 years. TOD has to be superior to or equal to the sum of the THI and LCD[58], here it will hence be 300 years. This method allows for each emission to be considered with the same decay period and for late emissions to be considered even when they occur at a date later than the THI. To treat the carbonation process with this method, each mass increment in carbon dioxide sequestered is considered as a pulse sink.

S4.5.5.3. Absolute global temperature potential

Absolute global temperature potential (AGTP) is defined as the global change in surface temperature at a THI, induced by an instantaneous emission, and has a unit of Kelvin (K). AGTP and instantaneous radiative forcing (IRF) are closely related indicators, as AGTP is directly derived from the radiative forcing response through the climate impulse response function. IRF can also be used to track dynamic climate change impacts caused by an emission. Here, we prefer to use AGTP because it expresses climate impacts in terms of temperature change, which may be more intuitive and easier to interpret for a broad audience, including decision-makers.

Firstly, an instantaneous radiative forcing (RF), which is the perturbation of the Earth's energy balance at the top of the atmosphere by a climate change mechanism, is calculated. This is directly proportional to the atmospheric decay of the GHG, as shown in (Equation S13):

$$\Delta RF_x(t) = a_x \cdot f_x(t) \tag{S13}$$

Where RF is the instantaneous radiative forcing, x represents the index for each GHG, α_x is the specific radiative forcing for the GHG being considered[55], and $f_x(t)$ is the atmospheric concentration of the related GHG. Similarly to atmospheric decay, the temperature impulse response function to instantaneous changes in RF can be written as (Equation S14):

$$\delta T(t) = \sum_i \left(\frac{c_i}{d_i} \cdot e^{\frac{-t}{d_i}} \right) \tag{S14}$$

where $\delta T(t)$ is the climate response to a unit forcing, c_i (K W^{-1} m^2) parameters are the components of the climate sensitivity and d_i (years) are response times, representing the climate's shorter time scale (d_1) and longer time scale responses (d_2), and t (years) is time. For climate sensitivity and response time parameters, we relied on the IPCC Anthropogenic and Natural Radiative Forcing report by Myhre et al.[59] (Anthropogenic and Natural Radiative Forcing Supplementary Material 8SM, Table 8.SM.9, c_1: 0.631 K W^{-1} m^2, c_2: 0.429 K W^{-1} m^2, d_1: 8.4 years, d_2: 409.5 years).

For a time-dependent change in RF, AGTP (Kelvin, K) is then computed as a convolution of $\Delta RF_x(t)$ and $\delta T(t)$ (Equation S15):

$$AGTP = \int_0^{TOD} \Delta RF_x(t) \cdot \delta(TOD - t) \mathrm{d}t \tag{S15}$$

where TOD (years) is the total observation duration, t (years) is the time, RF is the instantaneous radiative forcing, and $\delta T(t)$ is the climate response to a unit forcing.

References in this Supplementary Information S1 file

1 Joos, F., Roth, R., Fuglestvedt, J. S., Peters, G. P., Enting, I. G., von Bloh, W., Brovkin, V., Burke, E. J., Eby, M., Edwards, N. R., Friedrich, T., Frölicher, T. L., Halloran, P. R., Holden, P. B., Jones, C., Kleinen, T., Mackenzie, F. T., Matsumoto, K., Meinshausen, M., Plattner, G. K., Reisinger, A., Segschneider, J., Shaffer, G., Steinacher, M., Strassmann, K., Tanaka, K., Timmermann, A. & Weaver, A. J. Carbon dioxide and climate impulse response functions for the computation of greenhouse gas metrics: a multi-model analysis. *Atmos Chem Phys* **13**, 2793-2825 (2013). https://doi.org/10.5194/acp-13-2793-2013

2 Andersen, J. H., Rasmussen, N. L. & Ryberg, M. W. Comparative life cycle assessment of cross laminated timber building and concrete building with special focus on biogenic carbon. *Energ Buildings* **254** (2022). https://doi.org/10.1016/j.enbuild.2021.111604

3 Gustavsson, L., Pingoud, K. & Sathre, R. Carbon dioxide balance of wood substitution: comparing concrete-and wood-framed buildings. *Mitig Adapt Strat Gl* **11**, 667-691 (2006). https://doi.org/10.1007/s11027-006-7207-1

4 Hawkins, W., Cooper, S., Allen, S., Roynon, J. & Ibell, T. Embodied carbon assessment using a dynamic climate model: Case-study comparison of a concrete, steel and timber building structure. *Structures* **33**, 90-98 (2021). https://doi.org/10.1016/j.istruc.2020.12.013

5 Sinha, R., Lennartsson, M. & Frostell, B. Environmental footprint assessment of building structures: A comparative study. *Building and Environment* **104**, 162-171 (2016).

6 De Wolf, C., Pomponi, F. & Moncaster, A. Measuring embodied carbon dioxide equivalent of buildings: A review and critique of current industry practice. *Energ Buildings* **140**, 68-80 (2017). https://doi.org/10.1016/j.enbuild.2017.01.075

7 Zieger, V., Lecompte, T. & de Menibus, A. H. Impact of GHGs temporal dynamics on the GWP assessment of building materials: A case study on bio-based and non-bio-based walls. *Building and Environment* **185** (2020). https://doi.org/10.1016/j.buildenv.2020.107210

8 Xi, F. M., Davis, S. J., Ciais, P., Crawford-Brown, D., Guan, D. B., Pade, C., Shi, T. M., Syddall, M., Lv, J., Ji, L. Z., Bing, L. F., Wang, J. Y., Wei, W., Yang, K. H., Lagerblad, B., Galan, I., Andrade, C., Zhang, Y. & Liu, Z. Substantial global carbon uptake by cement carbonation. *Nat Geosci* **9**, 880-+ (2016). https://doi.org/10.1038/Ngeo2840

9 Crook, L. *Twenty per cent of new homes in Amsterdam to be constructed from timber*. (2021) <https://www.dezeen.com/2021/11/02/amsterdam-new-buildings-20-per-cent-timber/> (accessed 2025-09-02).

10 Schuler, T. A. *Dalston Works, the Largest CLT Building in the World*. (2018) <https://www.architectmagazine.com/technology/architectural-detail/dalston-works-the-largest-clt-building-in-the-world_o> (accessed 2025-09-02).

11 Pintos, P. *Mjøstårnet The Tower of Lake Mjøsa / Voll Arkitekter*. (2020) <https://www.archdaily.com/934374/mjostarnet-the-tower-of-lake-mjosa-voll-arkitekter> (accessed 2025-09-02).

12 Triple Wood. *HoHo Wien*. (2025) <https://www.triplewood.eu/en/projects/hoho-wien> (accessed 2025-09-02).

13 City of Vancouver. *Tools and Incentives to Encourage Mass Timber Construction.* (2025) <https://vancouver.ca/green-vancouver/mass-timber-buildings.aspx> (accessed 2025-09-02).

14 UNEP. Building Materials and the Climate: Constructing a New Future. (2023). <https://wedocs.unep.org/20.500.11822/43293> (accessed 2025-09-02).

15 Dai, M., Sun, M., Chen, B., Shi, L., Jin, M., Man, Y., Liang, Z., de Almeida, C. M. V. B., Li, J. & Zhang, P. Country-specific net-zero strategies of the pulp and paper industry. *Nature* **626**, 327-334 (2024). https://doi.org/10.1038/s41586-023-06962-0

16 Cavalett, O., Watanabe, M. D., Voldsund, M., Roussanaly, S. & Cherubini, F. Paving the way for sustainable decarbonization of the European cement industry. *Nat Sustain* **7**, 568-580 (2024). https://doi.org/10.1038/s41893-024-01320-y

17 Dunant, C. F., Joseph, S., Prajapati, R. & Allwood, J. M. Electric recycling of Portland cement at scale. *Nature* **629**, 1055–1061 (2024). https://doi.org/10.1038/s41586-024-07338-8

18 ARUP. *The time-value of carbon*. (2024) <https://www.arup.com/insights/the-time-value-of-carbon/> (accessed 2025-09-02).

19 Mishra, A., Humpenoder, F., Churkina, G., Reyer, C. P. O., Beier, F., Bodirsky, B. L., Schellnhuber, H. J., Lotze-Campen, H. & Popp, A. Land use change and carbon emissions of a transformation to timber cities. *Nat Commun* **13** (2022). https://doi.org/10.1038/s41467-022-32244-w

20 Churkina, G., Organschi, A., Reyer, C. P. O., Ruff, A., Vinke, K., Liu, Z., Reck, B. K., Graedel, T. E. & Schellnhuber, H. J. Buildings as a global carbon sink. *Nat Sustain* **3**, 269–276 (2020). https://doi.org/10.1038/s41893-019-0462-4

21 Yayla, A., Mason, A. R., Wang, J., van Ewijk, S. & Myers, R. J. Global wood harvest is sufficient for climate-friendly transitions to timber cities. *Nat Sustain* **8**, 1013–1025 (2025). https://doi.org/10.1038/s41893-025-01605-w

22 EN 15978: 2011. Sustainability of construction works. Assessment of environmental performance of buildings. Calculation method. (2011).

23 Yayla, A., Danneaux, A., Schurer, E., Gao, M., Demirci, C., Rose, C., van Ewijk, S. & Myers, R. J. Data repository for Mitigating hidden climate change impacts of timber cities critically depends on proactive forest and waste management. *Zenodo* (2025). https://doi.org/10.5281/zenodo.13886867

24 David B. McKeever, J. E. *Wood Products and Other Building Materials Used in New Residential Construction in the United States: with comparison to previous studies*. (2015) <https://research.fs.usda.gov/treesearch/48690> (accessed 2025-09-02).

25 Zhang, S., Ma, M., Zhou, N., Yan, J., Feng, W., Yan, R., You, K., Zhang, J. & Ke, J. Estimation of global building stocks by 2070: Unlocking renovation potential. *Nexus* **1**, 100019 (2024). https://doi.org/10.1016/j.ynexs.2024.100019

26 Meireis, C., Serino, F. S., Maia, C., Fontes, A. C. & Branco, J. M. Current Practice and Potential Associated with Timber-Based Solutions for Buildings Retrofitting. *Infrastructures* **7**, 25 (2022). https://doi.org/10.3390/infrastructures7020025

27 Gursel, A. P., Shehabi, A. & Horvath, A. What are the energy and greenhouse gas benefits of repurposing non-residential buildings into apartments? *Resources, Conservation and Recycling* **198**, 107143 (2023). https://doi.org/10.1016/j.resconrec.2023.107143

28 World Economic Forum. *Deep retrofits: how repurposing old buildings can mitigate climate change*. (2024) <https://www.weforum.org/stories/2024/02/deep-retrofit-buildings-carbon-emissions-climate-change/> (accessed 2025-09-02).

29 Kaza, S., Yao, L., Bhada-Tata, P. & Van Woerden, F. What a waste 2.0: a global snapshot of solid waste management to 2050. (2018). <http://hdl.handle.net/10986/30317> (accessed 2025-09-02).

30 Papakosta, A. & Sturgis, S. Whole life carbon assessment for the built environment. *RICS Professional Standards and Guidance; Royal Institution of Chartered Surveyors: London, UK* (2017).

31 Ecoinvent. *Ecoinvent Database*. (2024) <https://ecoinvent.org/> (accessed 2025-09-02).

32 Sacchi, R., Terlouw, T., Siala, K., Dirnaichner, A., Bauer, C., Cox, B., Mutel, C., Daioglou, V. & Luderer, G. PRospective EnvironMental Impact asSEment (premise): A streamlined approach to producing databases for prospective life cycle assessment using integrated assessment models. *Renewable and sustainable energy reviews* **160**, 112311 (2022). https://doi.org/10.1016/j.rser.2022.112311

33 Cherubini, F., Peters, G. P., Berntsen, T., Stromman, A. H. & Hertwich, E. CO_2 emissions from biomass combustion for bioenergy: atmospheric decay and contribution to global warming. *Gcb Bioenergy* **3**, 413-426 (2011). https://doi.org/10.1111/j.1757-1707.2011.01102.x

34 Liski, J., Pussinen, A., Pingoud, K., MSkipSS, R. & Karjalainen, T. Which rotation length is favourable to carbon sequestration? *Can J Forest Res* **31**, 2004-2013 (2001). https://doi.org/10.1139/cjfr-31-11-2004

35 Kaipainen, T., Liski, J., Pussinen, A. & Karjalainen, T. Managing carbon sinks by changing rotation length in European forests. *Environ Sci Policy* **7**, 205-219 (2004). https://doi.org/10.1016/j.envsci.2004.03.001

36 Eggleston, H., Buendia, L., Miwa, K., Ngara, T. & Tanabe, K. 2006 IPCC guidelines for national greenhouse gas inventories. (2006).

37 EPA. Advancing Sustainable Materials Management: 2018 Fact Sheet. (United States Environmental Protection Agency, 2020).

38 Papadakis, V. G., Vayenas, C. G. & Fardis, M. N. Fundamental Modeling and Experimental Investigation of Concrete Carbonation. *Aci Mater J* **88**, 363-373 (1991). https://doi.org/10.14359/1863

39 Lagerblad, B. *Carbon dioxide uptake during concrete life cycle: State of the art*. (Swedish Cement and Concrete Research Institute Stockholm, 2005).

40 Pomer, K. & Pade, C. Guidelines–Uptake of carbon dioxide in the life cycle inventory of concrete. (2005).

<https://www.dti.dk/_/media/21046_769418_Task%204_%20Guidelines_final%20report_DTI_%2031-01-2006.pdf> (accessed 2025-09-02).
41 von Greve-Dierfeld, S., Lothenbach, B., Vollpracht, A., Wu, B., Huet, B., Andrade, C., Medina, C., Thiel, C., Gruyaert, E., Vanoutrive, H., del Bosque, I. F. S., Ignjatovic, I., Elsen, J., Provis, J. L., Scrivener, K., Thienel, K. C., Sideris, K., Zajac, M., Alderete, N., Cizer, Ö., Van den Heede, P., Hooton, R. D., Kamali-Bernard, S., Bernal, S. A., Zhao, Z. F., Shi, Z. G. & De Belie, N. Understanding the carbonation of concrete with supplementary cementitious materials: a critical review by RILEM TC 281-CCC. *Mater Struct* **53** (2020). https://doi.org/10.1617/s11527-020-01558-w
42 Guo, R., Wang, J., Bing, L., Tong, D., Ciais, P., Davis, S. J., Andrew, R. M., Xi, F. & Liu, Z. Global CO2 uptake by cement from 1930 to 2019. *Earth Syst Sci Data* **13**, 1791–1805 (2021). https://doi.org/10.5194/essd-13-1791-2021
43 EN 16757:2017. Sustainability of construction works. Environmental product declarations. Product Category Rules for concrete and concrete elements. (2017).
44 Chang, C.-F. & Chen, J.-W. The experimental investigation of concrete carbonation depth. *Cement and Concrete Research* **36**, 1760-1767 (2006). https://doi.org/10.1016/j.cemconres.2004.07.025
45 Dodoo, A., Gustavsson, L. & Sathre, R. Carbon implications of end-of-life management of building materials. *Resources, conservation and recycling* **53**, 276-286 (2009). https://doi.org/10.1016/j.resconrec.2008.12.007
46 Takano, H. & Matsunaga, T. CO2 fixation by artificial weathering of waste concrete and coccolithophorid algae cultures. *Energy conversion and management* **36**, 697-700 (1995). https://doi.org/10.1016/0196-8904(95)00101-I
47 Meinshausen, M., Nicholls, Z. R., Lewis, J., Gidden, M. J., Vogel, E., Freund, M., Beyerle, U., Gessner, C., Nauels, A. & Bauer, N. The shared socio-economic pathway (SSP) greenhouse gas concentrations and their extensions to 2500. *Geosci Model Dev* **13**, 3571-3605 (2020). https://doi.org/10.5194/gmd-13-3571-2020
48 O'Neill, B. C., Kriegler, E., Riahi, K., Ebi, K. L., Hallegatte, S., Carter, T. R., Mathur, R. & van Vuuren, D. P. A new scenario framework for climate change research: the concept of shared socioeconomic pathways. *Climatic Change* **122**, 387-400 (2014). https://doi.org/10.1007/s10584-013-0905-2
49 Shah, V., Scrivener, K., Bhattacharjee, B. & Bishnoi, S. Changes in microstructure characteristics of cement paste on carbonation. *Cement and Concrete Research* **109**, 184-197 (2018). https://doi.org/10.1016/j.cemconres.2018.04.016
50 You, X. J., Hu, X., He, P. P., Liu, J. H. & Shi, C. J. A review on the modelling of carbonation of hardened and fresh cement-based materials. *Cement Concrete Comp* **125** (2022). https://doi.org/10.1016/j.cemconcomp.2021.104315
51 Gettu, R., Pillai, R. G., Santhanam, M., Rathnarajan, S., Basavaraj, A., Raju, S. & Dhandapani, Y. Service life and life-cycle assessment of reinforced concrete with fly ash and limestone calcined clay cement. (2019).
52 Shah, V. & Bishnoi, S. Carbonation resistance of cements containing supplementary cementitious materials and its relation to various parameters of concrete. *Constr Build Mater* **178**, 219-232 (2018). https://doi.org/10.1016/j.conbuildmat.2018.05.162
53 Rathnarajan, S., Dhanya, B. S., Pillai, R. G., Gettu, R. & Santhanam, M. Carbonation model for concretes with fly ash, slag, and limestone calcined clay-using accelerated and five - year natural exposure data. *Cement Concrete Comp* **126** (2022). https://doi.org/10.1016/j.cemconcomp.2021.104329
54 Zickfeld, K., Azevedo, D., Mathesius, S. & Matthews, H. D. Asymmetry in the climate-carbon cycle response to positive and negative CO2 emissions. *Nat Clim Change* **11**, 613-+ (2021). https://doi.org/10.1038/s41558-021-01061-2
55 Cherubini, F., Guest, G. & Stromman, A. H. Application of probability distributions to the modeling of biogenic CO2 fluxes in life cycle assessment. *Gcb Bioenergy* **4**, 784-798 (2012). https://doi.org/10.1111/j.1757-1707.2011.01156.x
56 Levasseur, A., Lesage, P., Margni, M., Deschenes, L. & Samson, R. Considering Time in LCA: Dynamic LCA and Its Application to Global Warming Impact Assessments. *Environ Sci Technol* **44**, 3169-3174 (2010). https://doi.org/10.1021/es9030003
57 Boucher, O. & Reddy, M. Climate trade-off between black carbon and carbon dioxide emissions. *Energy Policy* **36**, 193-200 (2008). https://doi.org/10.1016/j.enpol.2007.08.039
58 Ventura, A. Conceptual issue of the dynamic GWP indicator and solution. *Int J Life Cycle Ass* **28**, 788-799 (2023). https://doi.org/10.1007/s11367-022-02028-x

59 Myhre, G., D. Shindell, F.-M. Bréon, W. Collins, J. Fuglestvedt, J. Huang, D. Koch, J.-F. Lamarque, D. Lee, B. Mendoza, T. Nakajima, A. Robock, G. Stephens, T. Takemura and H. Zhang. Anthropogenic and Natural Radiative Forcing. In: Climate Change 2013: The Physical Science Basis. Contribution of Working Group I to the Fifth Assessment Report of the Intergovernmental Panel on Climate Change. (2013).
